# Non-uniform impact of extracellular osmotic variations at subcellular level

**DOI:** 10.1038/s41420-025-02703-6

**Published:** 2025-08-27

**Authors:** Pragya Singh, Aditya Mittal

**Affiliations:** Kusuma School of Biological Sciences, Indian Institute of Technology Delhi, Hauz Khas, Delhi 110016 India

**Keywords:** Organelles, Cellular imaging

## Abstract

Osmotic perturbations, towards understanding basic cellular architectures and to alter cellular mechanics for various purposes, are widely utilized in cell biology. While osmotic perturbations are known to alter whole-cell morphology, their subcellular-level impacts remain poorly characterized. Here, we employ a novel quantitative imaging workflow to demonstrate that extracellular osmolarity induces organelle-specific redistribution patterns in adherent RAW264.7 macrophages, independent of whole-cell morphological changes. At the whole-cell level, we report a decrease in cellular pleomorphism (pixel-intensity-distribution-based heterogeneity) under non-isotonic conditions, with cell membrane and lysosomal pleomorphism decreasing as osmolarity decreases. Remarkably, osmolarity-induced variations observed at whole-cell level are translated to actin and tubulin variations only while nucleus, mitochondria, and endoplasmic reticulum are independent of the whole cell morphology alterations. However, there appears to be ‘counterbalancing’ of lateral polarity in the distributions of nucleus and endoplasmic reticulum in hypo-osmotic conditions. This work promises to be a key contribution towards understanding cellular architectures.

## Introduction

The existence of a dynamic balance of interplay between diverse elements and their concentrations is required for cell survival. Such homeostasis is achieved by different transport mechanisms, including osmosis. Osmotic disturbances stand as one of the most frequent morphological perturbations for cells after temperature. Many scientific and physiological setups employ osmotic variations for various purposes, like osmotic loading, establishing cellular homeostasis responses to varying osmotic pressures, and reducing inflammation of tissues [1–3].

Investigations into different facets of osmosis and its implications on cellular phenotype were commenced in the late 1740s. It was observed that hyperosmotic environments resulted in a decrease in the cell size and an increased pleomorphy of the cell population, while the reverse was observed when cells were placed in a hyposmotic environment [4]. Apart from the whole cell morphological changes, reports also investigate the impact of osmotic alterations in the surrounding environment on different organelles [5–7]. Nucleus assumes a convoluted shape, and the nuclear lamina becomes more compressed when the cells are subjected to a hyperosmotic environment [8, 9]. Hyperosmolar treatments of intestinal epithelial cells affect mitochondrial oxygen consumption, resulting in a decrease in intracellular ATP [5]. However, alterations in earlier findings were observed with a few cells showing an increase in cell size/volume in hyperosmotic conditions, which led to futher detailed investigations into volume regulatory mechanisms/pathways of a cell in response to different osmotic conditions [6, 10–13]. Only recently, some studies involving quantification of osmotic effects on different cellular phenotypes were reported [11, 13–15]. Extensive research on osmolarity’s effects on cellular functions, such as osmotic fragility, membrane dynamics including membrane fusion, and hemolysis of erythrocytes, has been carried out [7, 13, 16–18]. However, comprehensive investigations into the quantitative 3D spatial changes in cells, including geometric organeller distributions and cytoskeletal arrangements, during osmotic perturbations, accompanying and/or resulting-in altered pleomorphism of the cell populations are missing/scarce in literature.

Differential spatial distribution of the organelles varies across different cell types as well as within the cells of the same type, giving rise to cell-to-cell variability and has several functional implications. An analytical tool developed to study the spatial distribution of organelle proteins using rapid multiplexed immunofluorescence (RapMIF) was able to establish differential patterns of organelle distribution of umbilical cord mesenchymal stem cells (UC MSCs) and bone marrow cells [19]. The organelle phenotype, which includes their spatial distribution, can impact various cellular processes, including differentiation, cell signaling, and cellular function [20–22]. For example, the spatial distribution of organelles such as the endoplasmic reticulum, mitochondria, and lysosomes can affect Calcium ion (Ca^2+^) signaling, cell shape regulation, and intracellular transport [23]. Additionally, the spatial distribution of organelles is linked to cellular functions such as cell polarization, growth, and energy production [24, 25]. Cell shape, cytoskeletal organization, intracellular transport mechanisms, such as molecular motors, and extracellular environment are some of the factors reported to contribute to the spatial distribution of organelles and in turn the overall cell morphology while any variations [23–26]. Changes in extracellular osmolarity can significantly impact the morphology of the whole cell as well as its internal architecture.

While investigations have been carried out studying the organelle distributions that incorporate cell-to-cell variability at interphase of pluripotent stem cells emphasizing the cellular organization to be a major read-out and driver of cellular behavior, we have built upon existing approaches in, this research, to employ our pipeline to investigate the osmolarity induced variations quantitatively in RAW264.7 macrophages [27, 28]. While previous studies have extensively documented the effects of osmotic pressure on whole-cell morphology, our study focuses on a novel quantitative approach to analyze subcellular morphological changes in response to osmotic stress [29, 30]. By employing high-resolution confocal microscopy and rigorous image analysis, we provide a detailed characterization of how osmotic stress affects the spatial distribution of organelles and cytoskeletal elements, uncovering organelle-specific responses that have not been previously explored. This study seeks to fill the existing gaps in our understanding of how distinct population morphological subtypes exhibit varied responses, in terms of morphological changes, to the same extracellular osmotic conditions, both at the holistic cellular level and within subcellular compartments including the cytoskeletal elements. We employed the use of RAW264.7 cells which have 2 distinct morphological subtypes: round/spherical cells as well as asymmetrical/irregularly shaped cells. The choice of RAW264.7 macrophages, with their intrinsic morphological heterogeneity, allowed us to dissect how pre-existing cellular architecture shapes osmotic adaptation [31]. Unlike homogeneous populations, these cells model the natural diversity encountered in vivo, where subpopulations employ distinct survival strategies (e.g., cytoskeletal polarization in asymmetric cells vs. structural uniformity in symmetric cells). This heterogeneity mirrors bet-hedging mechanisms observed in dynamic environments, where population-level resilience arises from functional diversification [32]. The cells were subjected to mild changes in extracellular osmolarity by modulating Sodium Chloride (NaCl) concentration in the commonly used buffer for biological systems, Phosphate Buffered Saline (PBS), followed by rigorous image analyses at the whole cell and subcellular component level using Z-stacks captured using Confocal microscopy.

## Results

**Fig. 1:**
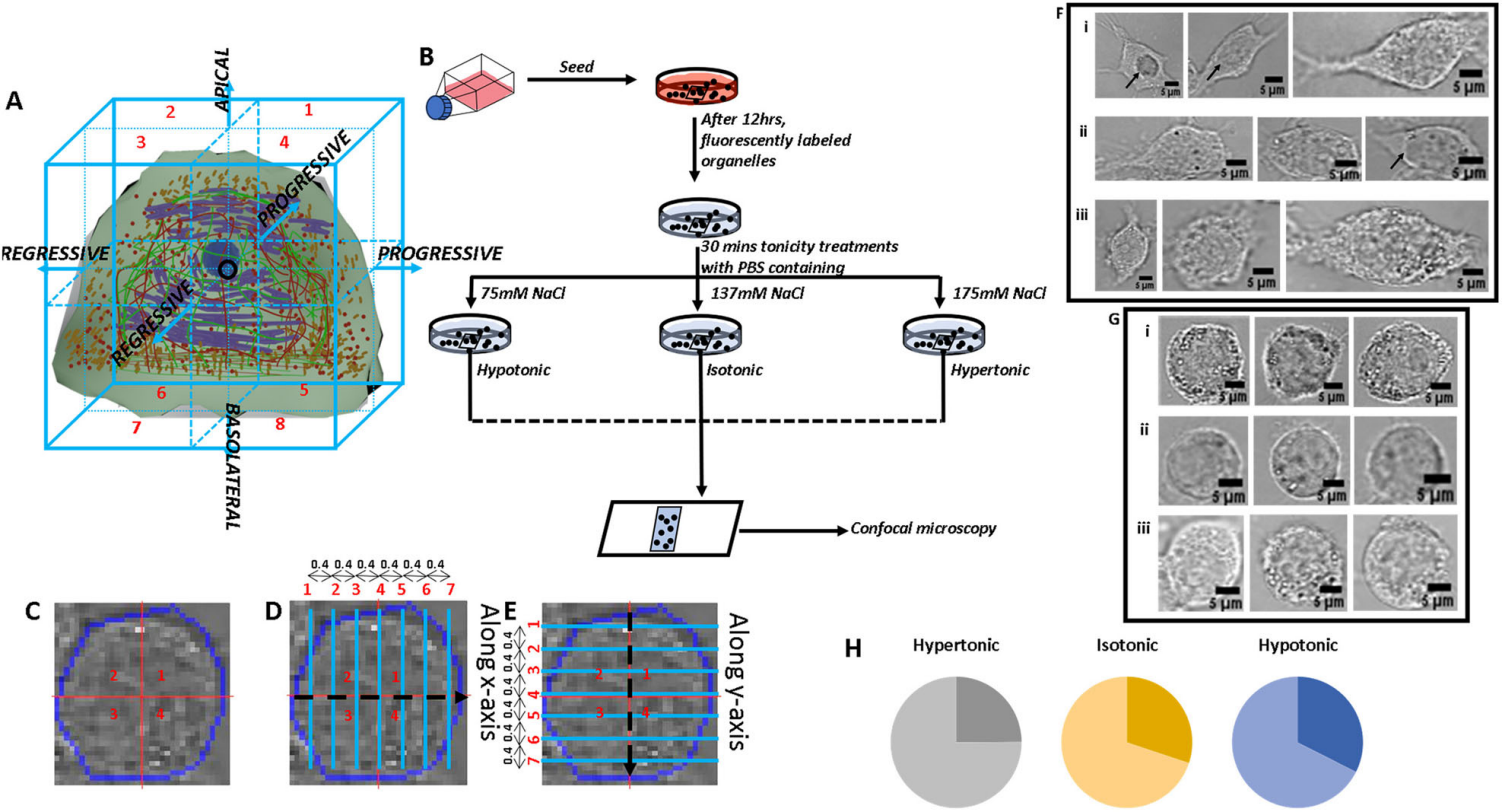
Experimental workflow and representative cell morphologies in different extracellular osmolarities. **A** Shows the division of 3D space of an artificial cell into octants around the centroid (marked in red) and the sides associated with octants along the 3 axes. **B** Flowchart showing the experimental methodology prior to image acquisition. Cells were cultured in a T25 flask till passage 4, followed by the seeding of approximately 50000 cells in a 35 mm Petri dish containing a 19 mm square cover slip. Cells were allowed to adhere to the surface of the coverslip and arrive in the log phase in 12 h. After 12 h, organelles were fluorescently labeled, and tonicity treatments were carried out for 30 min followed by slide preparation in the treatment buffer as mountant and image acquisition. Both DIC and fluorescent images were captured for each field. **C** Shows a representative asymmetrical cell, and the overlaying ROI (blue boundary). **D**, **E** Show the representative frame reading pattern along the *X* and *Y* axes, respectively. Imaging was carried out at in UPlanSApo 60x oil objective (N.A. 1.35) in Olympus FV1200 Confocal Microscope. **F**, **G** Show Three representative images of cells from the same section of single fields in the three environmental conditions, hypertonic (i), isotonic (ii) and hypotonic (iii) in asymmetric (**F**) and symmetric cells (**G**) of RAW264.7 cells. (**H**) show Pie chart representing the distribution of number of asymmetric (darker shades) and symmetric (lighter shades) cell subsets in RAW264.7 cell population in three different environmental conditions.

Images of a total of 1635 RAW264.7 cells were analyzed after treatment with PBS solutions varying in NaCl concentrations. The image data used in the analysis is a combined dataset of image data captured from different samples at different random time points. Images were acquired from multiple fields of view across replicate samples within a 30–45 min window post-treatment to ensure temporal consistency. This timeframe corresponds to the stabilization phase of osmotic adaptation, where cytoskeletal and organelle distributions reach equilibrium [15]. Isotonic PBS has 137 mM NaCl with a theoretical osmolarity of ~313 mOsm/L. Consequently, the concentration of NaCl in hypotonic and hypertonic PBS solutions was kept at 75 mM and 175 mM, respectively with the corresponding theoretical osmolarities as ~190 mOsm/L and ~390 mOsm/L. The cells were treated with NaCl-modulated PBS solutions for 30 min at room temperature. The number of cells in different treatment sets was 575, 548, and 512 in hypertonic, isotonic, and hypotonic treatments, respectively. The variation in the number of cells analyzed across treatment groups arises from technical and biological variability inherent in live-cell experiments, rather than intentional experimental design. The cells were further categorized as symmetric/asymmetric based on their respective circularity values determined using the mid-section image from the Z-stack. The cells with circularity value equal to or greater than 0.8 were categorized as symmetric and the ones with value less than 0.8 with categorized as asymmetric. This categorization was done based on a 1971 study in which cells with circularity value greater than 0.79 were classified as circular [33]. The distribution of asymmetric and symmetric cells in the three treatment sets is shown in Fig. 1H through pie charts. It is observed, from Fig. 1H, that hypertonic treatment significantly increased the proportion of symmetric (round) cells compared to isotonic conditions. The cells imaged were fluorescently labeled for different organelles. The shape-based division of the number of cells for each organelle in the three treatment conditions is mentioned in Table 1. The *p*-values from the statistical tests conducted between different sets of populations and treatments on the average-pixel-values (APVs) and total-pixel-numbers (TPNs) are mentioned in Tables S1–S5.

**Table 1:** Number of Cells imaged for each organelle in RAW264.7 cell population in Hypertonic, Isotonic, and Hypotonic conditions.

S.No.	Organelle	Asymmetric cells	Symmetric cells	Total
***Hypertonic***				
1.	Nucleus	23 (21%)	86 (79%)	109
2.	Cell membrane	34 (32%)	72 (68%)	106
3.	Mitochondria	23 (21%)	86 (79%)	109
4.	Endoplasmic Reticulum	34 (32%)	72 (68%)	106
5.	Lysosome	42 (34%)	80 (66%)	122
6.	Actin	31 (26%)	87 (74%)	118
7.	Tubulin	13 (12%)	97 (88%)	110
***Isotonic***				
1.	Nucleus	36 (30%)	85 (70%)	121
2.	Cell membrane	41 (41%)	58 (59%)	99
3.	Mitochondria	36 (30%)	85 (70%)	121
4.	Endoplasmic Reticulum	41 (41%)	58 (59%)	99
5.	Lysosome	41 (34%)	81 (66%)	122
6.	Actin	20 (19%)	85 (81%)	105
7.	Tubulin	27 (27%)	74 (73%)	101
***Hypotonic***				
1.	Nucleus	32 (29%)	77 (71%)	109
2.	Cell membrane	32 (30%)	72 (70%)	104
3.	Mitochondria	32 (29%)	77 (71%)	109
4.	Endoplasmic Reticulum	32 (30%)	72 (70%)	104
5.	Lysosome	33 (36%)	58 (64%)	91
6.	Actin	32 (30%)	72 (70%)	104
7.	Tubulin	37 (36%)	67 (64%)	104

The cells were stained for multiple organelles at a time. Hence, some of the numbers are repeated twice.

### Osmolarity-driven morphological alterations

The asymmetric subset of cells treated with the hypertonic PBS shows a middle roundish shape (Fig. 1F-i, marked by black arrow). However, only a few cells in isotonic condition show this inner round dense body, and none in hypotonic-treated cells (Fig. 1F-ii and iii). The symmetric cell subset of hypotonically-treated cells appears to be larger in size than hypertonic/isotonic cells (Fig. 1G-iii). Post-treatment imaging (Fig. 1F-i, G-i) confirmed that cells remained adherent during hypertonic exposure. No detachment or floating cells were observed, suggesting adhesion integrity was maintained. A careful visual examination of these cells show variations in cell sizes as well as variations in pixel intensity distribution patterns amongst these cells even though they belong the same Z-section of a Z-stack hinting towards the presence of innate morphological heterogeneity/pleomorphism in the population. The differences in apparent cell size between Fig. 1F, G are due to variations in zoom levels and image field sizes, although all quantifications are based on consistent analytical criteria of single cells. Additionally, these differences reflect the inherent variability in shape-dependent responses to osmotic conditions, which is a key focus of this study. These figures demonstrate the variability among cells of the same morphological subtype under different osmotic treatments. We vary the osmotic conditions of the extracellular environment of the cells to see and quantify how this morphological heterogeneity varies in the cell population for various parameters.

**Fig. 2:**
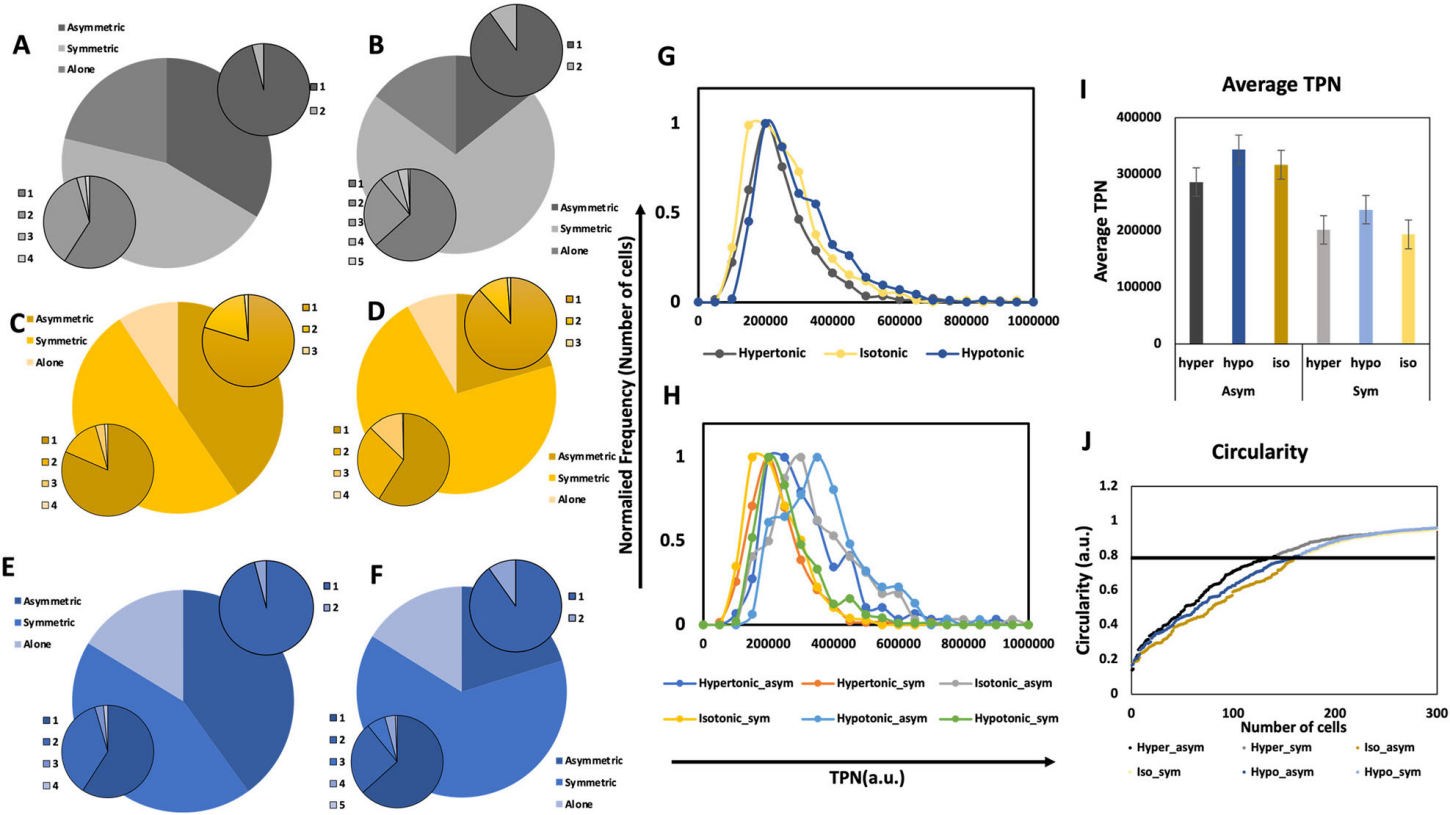
Osmolarity-dependent changes in cellular neighborhood and size distribution. **A**–**F** Pie charts depicting the distribution of neighbor types for asymmetric (**A**, **C**, **E**) and symmetric (**B**, **D**, **F**) cells under hypertonic (**A**, **B**), isotonic (**C**, **D**), and hypotonic (**E**, **F**) conditions. The large pie charts represent the proportion of asymmetric neighbors (blue), symmetric neighbors (orange), and cells without neighbors (gray) within each treatment subset. The small pie charts provide a more detailed breakdown: the top-right chart quantifies the number of asymmetric neighbors, while the bottom-left chart quantifies the number of symmetric neighbors for a given treatment. The numbers (1-4) indicate the specific count of neighboring cells in each subset. This classification helps visualize how osmotic conditions influence cellular interactions and clustering patterns. **G**, **H** Show the distribution of normalized frequency of number of cells against TPN values in the three treatment conditions (G-without shape segregation and H-with shape segregation) (**I**) shows the bar graphs of average TPN values (mean ± SE) in hypertonic (*n* = 143 asymmetric, *n* = 432 symmetric), hypotonic (*n* = 166 asymmetric, *n* = 346 symmetric), and isotonic conditions (*n* = 165 asymmetric, *n* = 383 symmetric) (**J**) shows the circularity value plot of the two shape subsets in the three treatment conditions.

Figure 2A–F illustrates the distribution of neighboring cell types and their respective counts surrounding a particular cell type under different osmotic conditions. The large pie charts represent the proportion of asymmetric neighbors (blue), symmetric neighbors (orange), and cells without any neighbors (gray). The small pie charts provide a more detailed breakdown: the top-right small pie chart quantifies the number of asymmetric neighbors, while the bottom-left small pie chartquantifies the number of symmetric neighbors for a given treatment. The numbers 1, 2, 3, 4, 5 in Fig. 2A-F indicate specific neighbor counts in each subset, offering insights into cell clustering patterns. Various studies have demonstrated the influence of cell-to-cell interactions on various processes in RAW264.7 cells. While in our previous study we found the absence of any correlation between the neighbor type and number, in the present study we aimed to study whether osmolarity alterations affect the neighboring feature of the cell population. Asymmetric subsets show an increasing number of cells with asymmetric neighbors as the concentration of NaCl decreases. It is observed that the number of asymmetric neighbors of both the asymmetric and symmetric subsets is limited to a maximum number of 2 in hypertonic and hypotonic conditions; while in the isotonic condition, the maximum number of asymmetric neighbors reaches 3. Similarly, a maximum of 5 symmetric cells can be found to surround a symmetric cell in both hypertonic and hypotonic conditions as opposed to a number of 4 cells in the isotonic PBS. The maximum number of symmetric neighbors for asymmetric cells was found to be 4 in all three conditions.

### Quantitative DIC analysis

Figure 1C shows a single cell defined by ROI (Region Of Interest) and division of the cellular space into 1–4 quandrants. Figure 1D, E further depict the frame reading patterns along the *x* and *y* axes, respectively, with consecutive planes being 0.4 µm apart. The total pixel number (TPN) served as the indicator of cell size and was calculated for each cell analyzed. The cells are expected to shrink in a hypertonic solution and expand in a hypotonic solution. While we do observe this behavior in the case of the asymmetric cells’ subset, the symmetric cells subset shows a peculiar behavior. The symmetric cells in hypertonic solution show a 4% larger size than the cells in isotonic solution while the cells treated with hypotonic solution showed a 22% larger cell size (Fig. 2I). After shape-based segregation of the cell population, it was observed that the asymmetric cells showed a 9.6% smaller cell size in hypertonic PBS than the cells in isotonic PBS, while cells in hypotonic PBS showed an 8.5% larger cell size (Fig. 2I). Figure 2 G, H shows the frequency distributions of the cell size (defined by TPN) in the murine macrophages treated with modulated PBS solutions before and after shape-based population segregation (carried out based on circularity values - ≥0.8 as symmetric and <0.8 as asymmetric, as mentioned earlier) respectively. Figure 2J depicts the circularity value distribution of RAW264.7 cell population in different osmotic treatments.

**Fig. 3:**
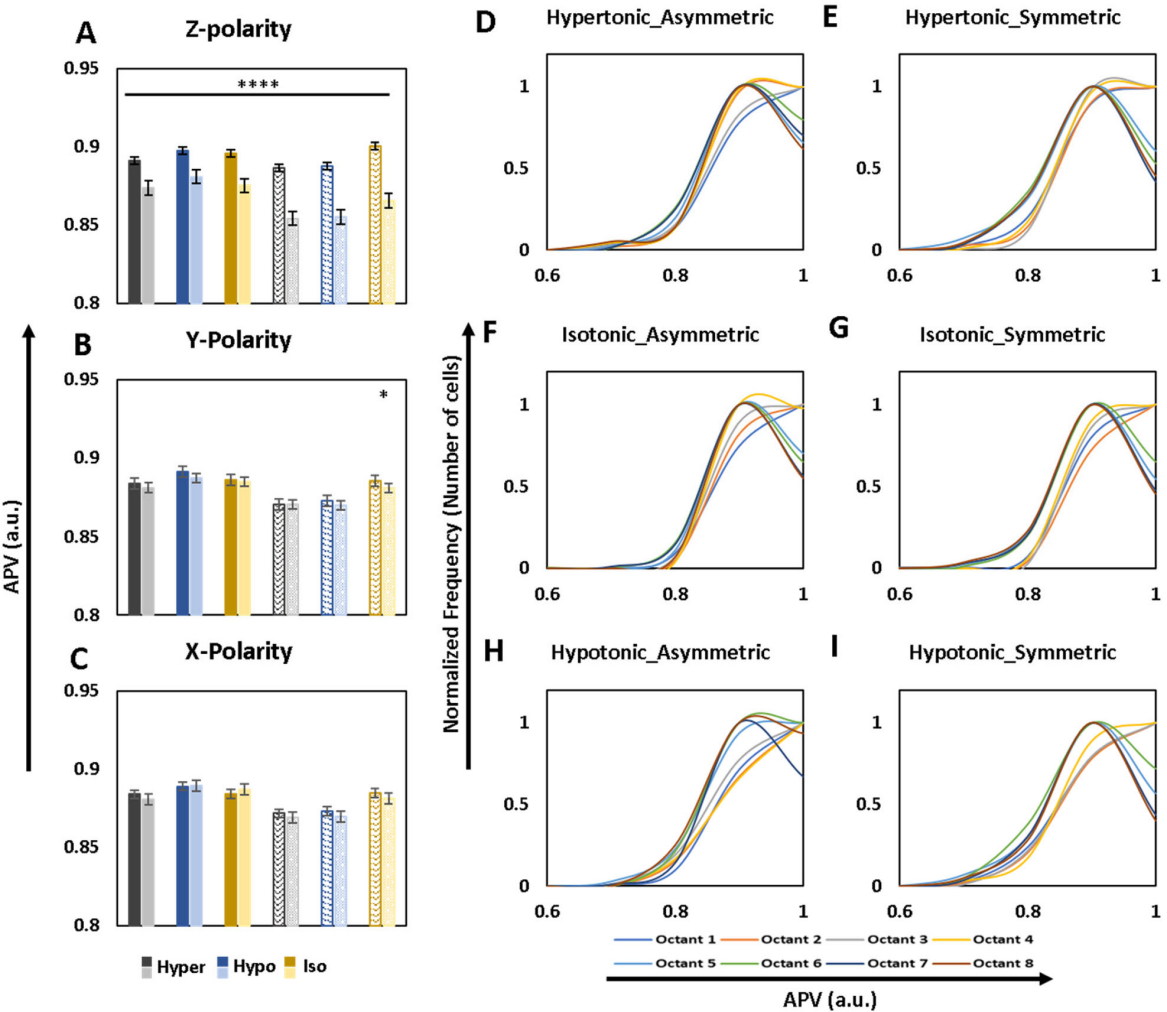
Quantitative spatial analysis of intracellular density reveals osmolarity-induced apico-basal polarity and uniformity. **A**–**C** shows bar graphs depicting average APVs of DIC images of in asymmetric (solid bars) and symmetric (patterned bars) RAW264.7 cells along the *z* (**A**), *y* (**B**), and *x* (**C**) axes in hypertonic (black and gray bars, *n* = 143 asymmetric representing *n* = 432 symmetric, respectively), hypotonic (dark and light blue bars representing *n* = 166 asymmetric, *n* = 346 symmetric, respectively), and isotonic (dark and light-yellow bars representing *n* = 165 asymmetric, *n* = 383 symmetric, respectively) conditions (mean ± SE). **D**–**I** Show the distribution normalized frequency of the number of cells along APV values for each octant (color coding below Fig. **H** and **I**) in asymmetric (**D**, **F**, **H**) and symmetric (**E**, **G**, **I**) RAW264.7 cells in hypertonic (**D**, **E**), isotonic (**F**, **G**) and hypotonic (**H**, **I**) conditions.

The average pixel values (APVs) were tabulated for each cell and normalized using the highest APV of the field. The raw APVs are given in Tables S6–S11. On analyzing the normalized pixel intensities in the 3D space of cells using DIC images, apico-basal polarity is observed with denser basolateral sections (Fig. 3A–C). The apico-basal trend is observed in both the asymmetric and symmetric cell subsets in all three conditions, i.e., hypertonic, isotonic, and hypotonic, with the basolateral sections being 1.9%, 2.2%, and 1.8% denser than apical sections respectively in asymmetric cell subset while in the symmetric cells the differences are 3.6% in both hypertonic and hypotonically treated cells and 3.9% in the cells in isotonic solution. The frequency distributions of cell numbers against APVs for each octant show varying trends, with the uniformity in distribution increasing from hypertonic to hypotonic PBS treatments (Fig. 3D–I). It is also observed that octants 7 and 8 show the same frequency distributions of APVs in both shape-subsets in all treatment conditions baring asymmetric cells in hypotonic conditions, indicating towards the presence of a constant structural feature (possibly molecular entities) residing simultaneously in octants 7 and 8. Isotonic symmetric cells also show y-polarity with a 0.5% denser progressive side (Fig. 3B).

### Quantification of subcellular distribution variations

**Fig. 4:**
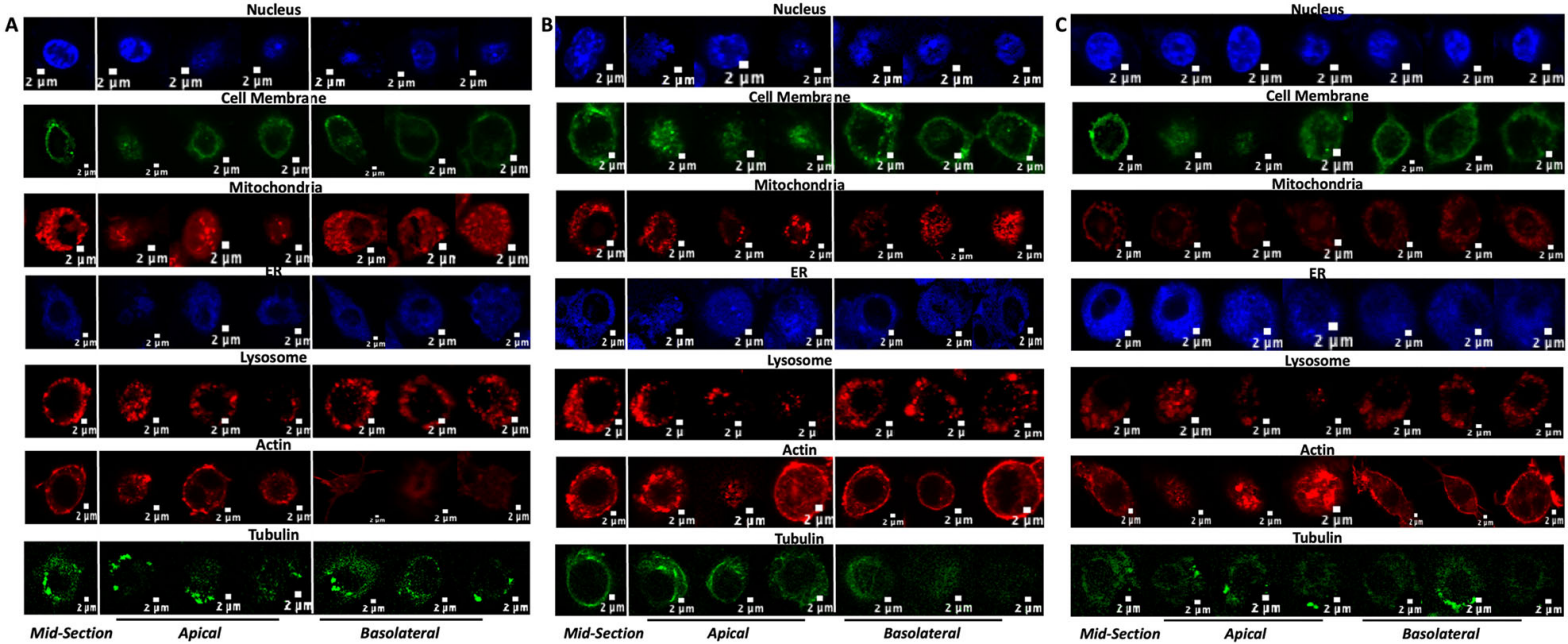
Representative organelle-specific architectural changes in response to different extracellular osmolarities. **A**–**C** Images above show 7 by 7 panels of fluorescent images of different organelles in a single cell in all the three treatment conditions, hypertonic (**A**), isotonic (**B**) and hypotonic (**C**). The first image of each panel is a mid-section image of a single-cell organelle fluorescence from a Z-stack. The mid-section image is followed by representative images of one of the apical sections of a Z-stack of 3 single cells followed by their corresponding basolateral section images. Imaging was carried out at in UPlanSApo 60x oil objective (N.A. 1.35) in Olympus FV1200 Confocal Microscope.

In the hypertonic condition, the heterogeneity in the distribution of fluorescence in the apical and basolateral section (along the *z*-axis) is clearly visible (Fig. 4A). Pixel-intensity-based heterogeneity decreased under hypertonic conditions at the whole cell level (Fig. 4A), supporting reduced irregularity. This heterogeneity exists in isotonic condition as well but is not profound in the case of a few organelles like mitochondria, endoplasmic reticulum, and nucleus (Fig. 4B). Uniformity in the fluorescence distribution increases in hypotonic treatment (Fig. 4C).

**Fig. 5:**
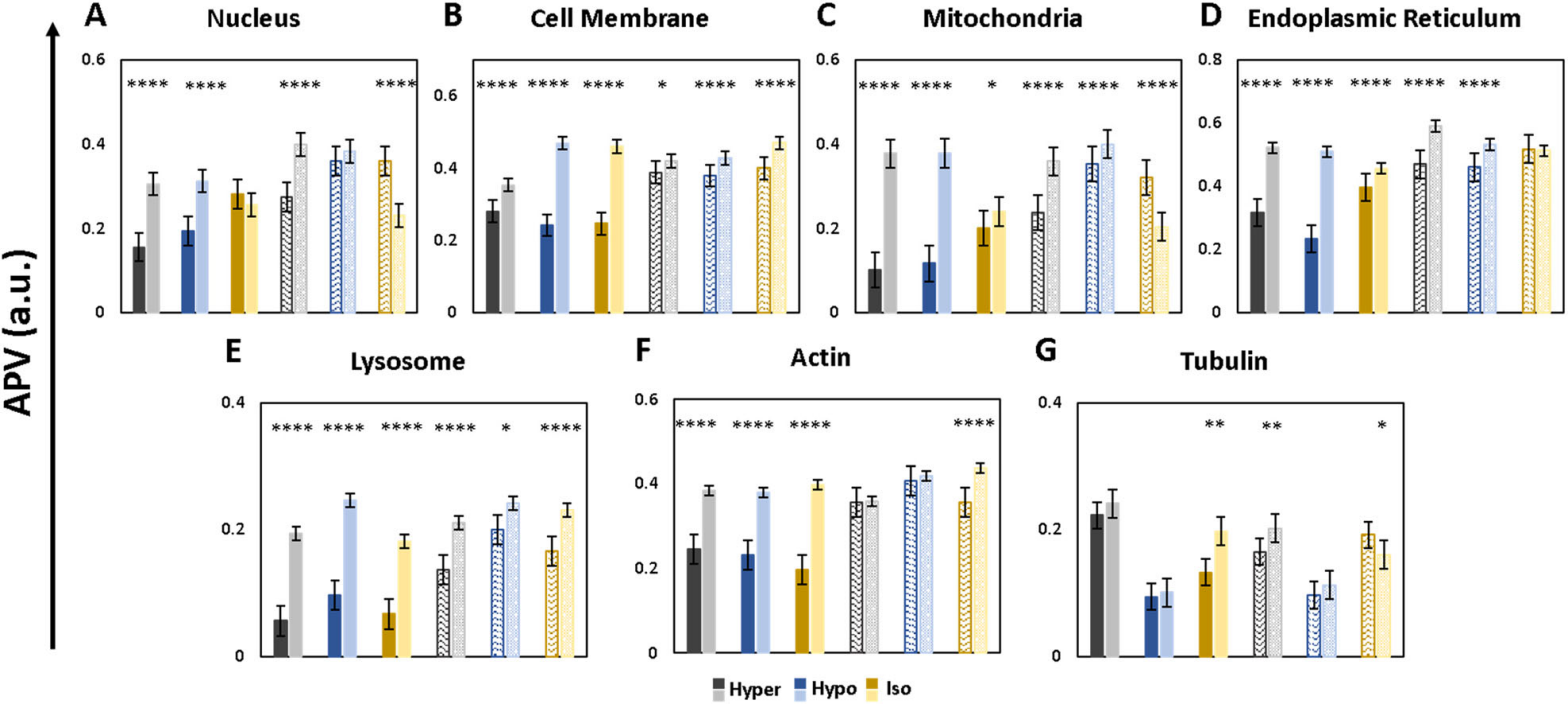
Organelle-specific spatial distribution and polarity are differentially regulated by extracellular osmolarity. **A**–**G** Shows bar graphs depicting average APVs (along the *z*-axis) of fluorescent images of nuclei (**A**), Cell membrane (**B**), Mitochondria (**C**), Endoplasmic Reticulum (**D**), Lysosome (**E**), Actin (**F**), and Tubulin (**G**) in asymmetric (dark colored solid bars) and symmetric (light colored patterned bars) RAW264.7 cells along the *z* axis direction in hypertonic (black and gray bars representing asymmetric and symmetric cells, respectively), hypotonic (dark and light blue bars representing asymmetric and symmetric cells, respectively), and isotonic (dark and light-yellow bars representing asymmetric and symmetric cells respectively) conditions (mean ± SE). Individual n values are mentioned in Table 1.

The APV distribution analysis of fluorescence signals of the seven organelles imaged shows an apico-basal polarity with predominantly basolateral preference in all the organelles, the trend varying in different tonicity conditions in the fluorescence signals of five organelles, namely, nucleus, mitochondria, endoplasmic reticulum, actin, and tubulin (Fig. 5A, C, D, F, G). Cell membrane and lysosome, on the other hand, do not show altering trends on varying NaCl concentration in PBS buffer (Fig. 5B, E). The lateral polarities are observed in the nucleus, mitochondria, and endoplasmic reticulum (Fig. S1). However, the observation is made only on certain shape subsets in only one of the tonicity conditions.

Nuclear fluorescence signals predominantly show apico-basal polarity with the basolateral preference of approximately 97% (indicating a 2-fold change) and 45% in asymmetric and symmetric cell subsets, respectively, in hypertonic conditions. However, as the concentration of NaCl in the treatment buffer decreases, the polarity of the fluorescence signals changes to apical (~57% over basolateral) in symmetric cells in isotonic condition to the absence of polarity altogether in hypotonic treatment (Fig. 5A). The peculiar observation of the existence of lateral polarity, Y-regressive, is seen only in the case of hypotonic treatment irrespective of the shape subset (32% and 14.5% in asymmetric and symmetric cell subset, respectively) while the apico-basal polarity of the fluorescence signals with basolateral preference (61% over apical APV) is seen in case of asymmetric cell subset in hypotonic condition (Fig. S1A).

Cell membrane fluorescence signals exclusively show basolateral preference of apico-basal polarity irrespective of the treatment or shape subset. However, the difference between the apical and basolateral signals is more pronounced in the isotonic (87% and 8% in asymmetric and symmetric cells, respectively) and hypotonic (94% and 3% in asymmetric and symmetric cell subset, respectively) treatment sets than in hypertonic (26% and 7% in asymmetric and symmetric cell subset, respectively) treatment (Fig. 5B).

Asymmetric cells’ mitochondrial fluorescence distributions are basolateral in nature with hypertonic and hypotonic treatments showing a more pronounced difference (272%, and 225% respectively) than isotonic apico-basal distribution (at 20% higher basal fluorescence than apical) (Fig. 5C). Symmetric cells show 51% and 13% higher basal mitochondrial fluorescence in hypertonic and hypotonic treatments respectively while the isotonic treatment showed a lateral (regressive in x-direction - 13% over progressive) apical preference (57% over basolateral APV) (Fig. 5C). Endoplasmic reticulum showed a basolateral preference of fluorescence distribution in all the treatment conditions irrespective of the shape subsets except symmetric cells in isotonic condition showing lack of any polarity (Fig. 5D). Asymmetric cells show 64%, 15% and 118% higher basolateral fluorescence signals than apical signals in hypertonic, isotonic and hypotonic conditions respectively while symmetric cells show only 26% and 16% higher basolateral fluorescence signals than apical in hypertonic and hypotonic conditions, respectively (Fig. 5D). A lateral polarity along y-direction in hypotonic treatment (15% higher progressive preference of fluorescence distribution than regressive) correlates well with our observation of nuclear fluorescence distribution showing a y-regressive preference (Fig. S1A). Lysosome fluorescence distributions, like cell membrane, show a basolateral preference, independent of the shape of the cell with asymmetric cells showing higher fold changes (3.45, 2.72, and 2.53 fold change in basolateral fluorescence than apical fluorescence in hypertonic, isotonic and hypotonic treatments respectively) than symmetric cells (which show 1.53, 1.4, and 1.21 in hypertonic, isotonic and hypotonic treatments respectively) (Fig. 5E).

The spatial fluorescence distributions of cytoskeletal elements, actin and tubulin, were observed to be most dynamic with polarities being apical, basolateral and some sets also showing complete lack of polarity (Table 2). Similar to earlier observations, asymmetric cells showed higher degree of spatial fluorescence signal distribution differences (57%, 103%, and 64% for actin fluorescence in hypertonic, isotonic, and hypotonic treatments and 48% in isotonic tubulin fluorescence) than symmetric cells (22% higher basal actin fluorescence in isotonic treatment and 22% more basal and 20% more apical tubulin fluorescence in hypertonic and isotonic treatments, respectively) (Fig. 5F, G).

**Table 2:** Type of Polarity in APV distribution in the asymmetric and symmetric cells in 3D geometric space of cells in RAW264.7 population.

	MEDIA [28]	HYPERTONIC	ISOTONIC	HYPOTONIC
***Asymmetric***				
*WHOLE CELL*	Basolateral	Basolateral	Basolateral	Basolateral
*NUCLEUS*	Basolateral	Basolateral	Nil	Basolateral, Y-Regressive
*CELL MEMBRANE*	Basolateral	Basolateral	Basolateral	Basolateral
*MITOCHONDRIA*	Basolateral	Basolateral	Basolateral	Basolateral
*ENDOPLASMIC RETICULUM*	Basolateral	Basolateral	Basolateral	Basolateral, Y-Progressive
*LYSOSOME*	Basolateral	Basolateral	Basolateral	Basolateral
*ACTIN*	Basolateral	Basolateral	Basolateral	Basolateral
*TUBULIN*	Nil	Nil	Basolateral	Nil
***Symmetric***				
*WHOLE CELL*	Basolateral	Basolateral	Basolateral, Y-Progressive	Basolateral
*NUCLEUS*	Basolateral, X-Regressive	Basolateral	Apical	Y-Regressive
*CELL MEMBRANE*	Basolateral	Basolateral	Basolateral	Basolateral
*MITOCHONDRIA*	Nil	Basolateral	Apical, X-Regressive	Basolateral
*ENDOPLASMIC RETICULUM*	Apical	Basolateral	Nil	Basolateral
*LYSOSOME*	Basolateral, Y-Regressive	Basolateral	Basolateral	Basolateral
*ACTIN*	Basolateral	Nil	Basolateral	Nil
*TUBULIN*	Basolateral	Basolateral	Apical	Nil

The octants contributing towards the observations of pixel intensity distributions are listed in Table 3 after a careful analysis of the normalized frequency distributions along APVs for each octant (Fig. S2).

**Table 3:** Contributing Octants towards Polarity type in APV distribution in the asymmetric and symmetric cells in 3D geometric space of cells in RAW264.7 population.

	MEDIA [28]	HYPERTONIC	ISOTONIC	HYPOTONIC
***Asymmetric***				
*WHOLE CELL*	5–8	5–8	6,7	6–8
*NUCLEUS*	6,8	8	–	5,6
*CELL MEMBRANE*	5,6,8	6	5,6	7,8
*MITOCHONDRIA*	5,6,8	7,8	7	8
*ENDOPLASMIC RETICULUM*	5,8	7	5	8
*LYSOSOME*	7,8	8	6	6
*ACTIN*	5–8	8	6	8
*TUBULIN*	–	–	7	–
***Symmetric***				
*WHOLE CELL*	5–8	5–8	5–8	5–8
*NUCLEUS*	7,8	8	1,4	1,2
*CELL MEMBRANE*	5–8	8	8	5,6
*MITOCHONDRIA*	–	8	3	6,8
*ENDOPLASMIC RETICULUM*	1–4	7	—	8
*LYSOSOME*	6,7	5	5	8
*ACTIN*	5–8	–	7	–
*TUBULIN*	5–8	5	3	–

## Discussion

**Fig. 6:**
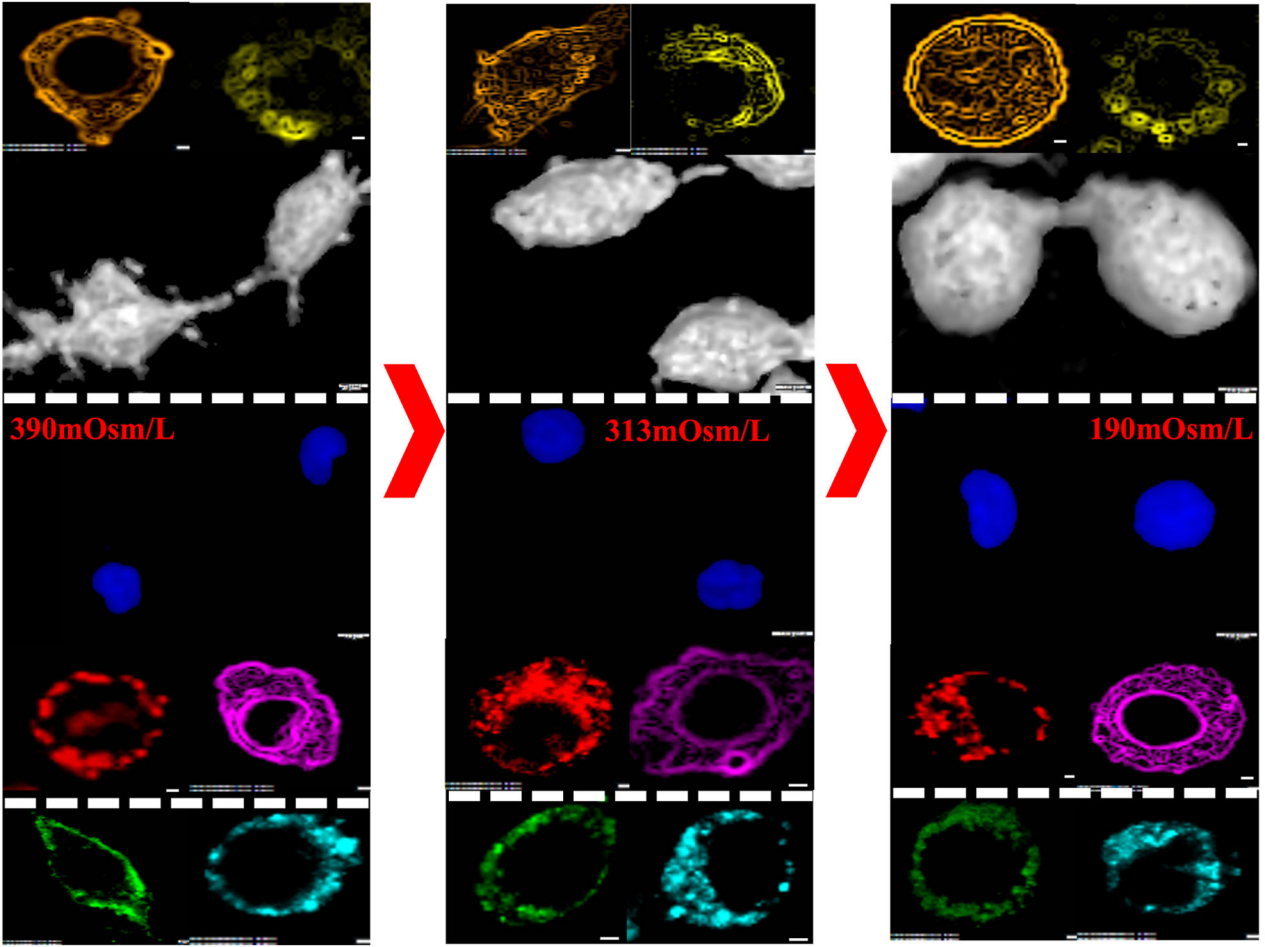
Schematic summary of responses of subcellular compartments to different extracellular osmolarities. Schematic showing the representative images of whole cell (3D Blending Projections - White) and organelles – Nucleus (3D Mean Intensity Projections - Blue), Cell Membrane (Green), Mitochondria (Red), Endoplasmic Reticulum (Purple), Lysosome (Cyan), Actin (Orange), and Tubulin (Yellow) in three different Osmolarities. Dotted white line depicts segregation of organelles into 3 categories, (1) Actin and Tubulin following whole cell parametric variation trends, (2) Nucleus, Mitochondria and Endoplasmic Reticulum being independent of the whole cell parametric variation trends, and (3) Plasma Membrane and Lysosome showing the ‘expected’ variations with the increasing osmolarity.

Even though the knowledge of osmosis in living cells has been in existence for over 2 centuries, the large amount of literature fails to provide a quantitative systemic outlook of the morphological changes resulting from osmotic shocks/variations at the whole cell and subcellular component level in adherent cells. Our study is one of the first attempts to develop a systematic and rigorous methodology for quantitatively investigating morphological changes occurring under varying extracellular osmolarities, at subcellular levels. The cells were subjected to mild variations in osmolarity with respect to NaCl concentrations in PBS to conclusively determine the effect of concentration variations of a single salt. The osmolarities tested in our study (190–390 mOsm/L) align with ranges commonly encountered in biological systems. For example, mammalian cells typically tolerate osmolarities between ~250–350 mOsm/L, and deviations beyond this range often lead to pathological outcomes such as cell lysis (hypotonic) or apoptosis (hypertonic) [3, 34]. By focusing on mild osmotic perturbations, we aimed to study subcellular responses under conditions where cells retain viability and regulatory mechanisms (e.g., volume regulatory ion channels) remain functional [10]. While distinct morphological variations were visible upon treatment of cells with NaCl-modulated PBS, a more rigorous image analysis provides a quantitative estimate of the variations occurring at the whole cell level as well as the organelle level. Figure 6 illustrates a schematic representing the comprehensive outcomes of the study.

Earlier studies showed 2 major types of cell size changes when the cells are subjected to osmotic stress, with studies showing both increase and decrease in cell size when placed under hypertonic stress [6, 11]. An avian-specific differential behavior of erythrocytes to different osmotic conditions was also observed in contrast to their mammalian counterparts [13]. However, prior work studying the effects of different osmotic conditions on various cell types may not apply to RAW264.7 macrophages, which exhibit unique cytoskeletal dynamics and adhesion mechanisms. RAW264.7 cells prioritize rapid volume regulation (via ion channels) over adhesion remodeling under mild osmotic stress, leading to transient rounding [10, 29, 35]. We observed that in RAW264.7 cell population, which have two distinct morphological subsets, the cell size variations on hypertonic treatments are shape dependent with pleomorphic shapes showing an increase in cell size and symmetric shapes showing the decrease in cell size upon hypertonic PBS treatment hinting towards the shape-based differential cell responses and hence volume regulatory mechanisms existing in the same population. We expected the morphological pleomorphism to decrease with a decrease in osmolarity as the cells become more round when placed in a hypotonic solution. Contrary to the expected behavior, we observed an increase in shape-based pleomorphism of the cell population with a decrease in osmolarity, which, on visual examination, we attribute to the peculiar shapes of asymmetric shape subsets in hypotonic conditions. The differences in morphological heterogeneity under varying osmotic conditions correspond to distinct subcellular spatial organization patterns, suggesting that osmotic stress induces structural adaptations/responses at both the whole-cell and organelle levels [22, 23, 36–38]. Organelle redistribution, including counterbalancing nucleus-ER polarity, compensates for volume changes, preserving structural integrity [22, 23]. These immediate-adaptations/responses influence immune function, phagocytosis, and tissue interactions, particularly in macrophages responding to osmotic stress [3, 36]. Physiologically, osmolarity-driven changes impact cell homeostasis, inflammation, and disease states [3, 37–39], with implications for tissue engineering, where controlling osmolarity could enhance organelle positioning for improved cell survival [40, 41]. Our findings provide insights into how cells dynamically adapt to osmotic stress, which is crucial for biomedical research, disease modeling, and therapeutic strategies [3, 37, 38, 41].

The osmotic-driven variation in neighbor numbers (Fig. 2A–F) likely reflects dynamic adjustments in cell adhesion and motility. Hypertonic stress may reduce cadherin-mediated adhesion, limiting stable neighbor interactions, while hypotonic swelling could transiently enhance motility and alter cell clustering [37, 40, 42]. These trends align with cytoskeletal reorganization (Fig. 5F, G), where actin polarization stabilizes rounded morphologies in hypertonic conditions, and diffuse tubulin distributions permit membrane plasticity during swelling. While neighbor number changes suggest altered adhesion/migration, this study did not directly quantify adhesion molecules (e.g., cadherins) or migration dynamics. Future studies incorporating biochemical assays (e.g., cadherin immunostaining) or live-cell tracking could elucidate the molecular drivers of these population-level changes.

A quantitative spatial analysis of average pixel values distributions at the whole cell level, accomplished using the DIC images, showed a consistent basolateral preference. However, population frequency distributions of octants against APVs shed light on the increase in uniformity of pixel distributions in 3D space of the cell as the osmolarity varies from isotonic condition (Table 4). The band of difference starting at an APV of 0.7 existing between the basolateral and apical octants in isotonic and hypertonic treatment symmetric cell subsets disappears in hypotonic solution and the difference between basolateral and apical octants start appearing at 0.8 APV leading us to conclude that there is an increase in uniformity of distribution of pixel intensities lower than 0.7 between the 2 sections along z-axis upon decrease in osmolarity of the extracellular solution along with an increase in the common area under the curves of population frequency distributions of octant APVs.

**Table 4:**
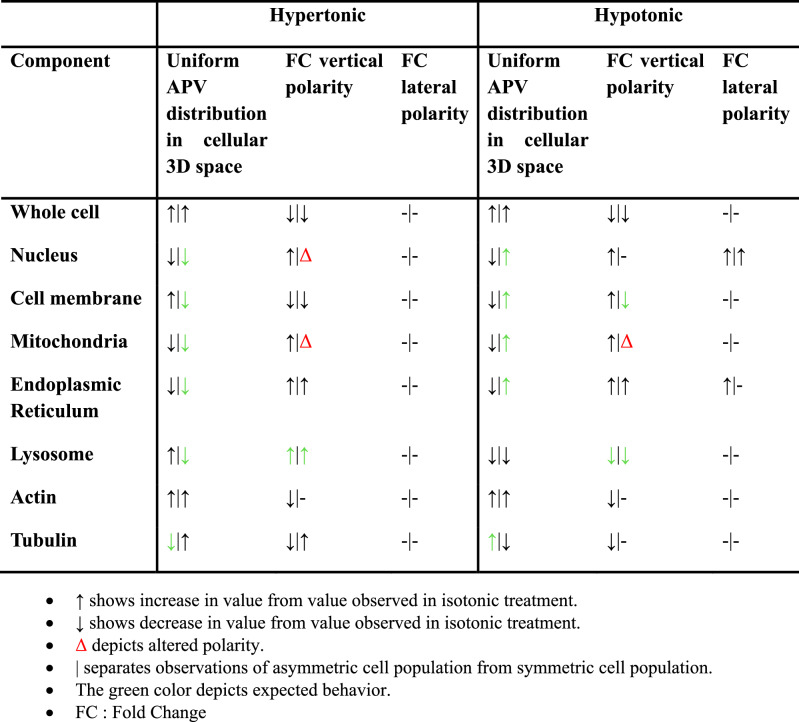
Trends in Parameter variations observed in Hypertonic and Hypotonic Osmotic shocks with respect to Isotonic treatment.

While a lateral polarity is observed in isotonic symmetric cells at the whole cell level, at the organelle level, the lateral polarities exist mainly in asymmetric cells in hypotonic conditions. The independent observations of existence of opposing lateral polarities in nuclear and endoplasmic reticulum fluorescence distributions along y direction upon treatment with hypotonic PBS solution hints towards this shape-dependent phenomenon being a constant occurrence when cells are subjected to osmotic stress. The existence of lateral polarity at the whole cell level in isotonic condition remains requires further in-depth investigations.

Organelle fluorescence distributions showed predominantly apico-basal polarity with a basolateral preference of distribution. However, alterations in polarity, such as apical preference and lack of polarity, are observed when cells are placed in different media. The polarities observed in PBS also vary significantly from the polarities observed in cells placed in culture media. The incomplete DMEM has a NaCl concentration of 81 mM, closest to the hypertonic PBS with 75 mM NaCl. Yet the presence of other components in DMEM makes the polarity patterns at the organelle level significantly different from the PBS treatments, giving us concrete proof that the minute variations in the extracellular environment play a major role in determining the subcellular architecture.

The cellular architecture is controlled by the cytoskeletal elements. The cytoskeletal elements in the present study, actin and tubulin, show the most dynamic variations in distribution in different treatments. The contributing octant analysis provides a strong background to speculate on the role of actin and tubulin in the distribution of various organelles. Both tubulin and mitochondria show the 3rd octant to result in observed polarities, respectively, on the basis of which we infer that tubulin might be one of the major players in the spatial distribution of mitochondria. Similarly, the existence of apical polarity in both tubulin and the nucleus in isotonic conditions offers further evidence of cytoskeletal elements playing a role in organelle distribution. The role of actin in thhe cellular cortex has been studied in-depth [43, 44]. The basolateral polarity of actin correlates well with the basolateral polarity of the cell membrane in asymmetric cells while the lack of polarity in actin distribution upon hypertonic and hypotonic treatments leads us to infer that actin plays an active in determining the overall cell structure when the cell encounter the less than favorable environment.

While mitochondria, nucleus, and ER exhibited unchanged spatial distributions under osmotic stress (Fig. 5A, C, D), their functional states likely adapt to meet cellular demands. Mitochondria may elevate ATP production to fuel ion transport [10], while the nucleus activates osmoprotective transcription programs (e.g., NFAT5) [45]. The ER, though spatially stable, could initiate stress-responsive pathways (e.g., UPR) to maintain proteostasis [46]. These functional adjustments underscore that organelle resilience involves both structural integrity and biochemical plasticity, ensuring survival under osmotic challenge.

Table 4 serves as a concluding interpretation medium for understanding how various parameters change with mild osmotic variations in the extracellular environment of RAW264.7 cells in the 2 shape subsets. We observe that while cell membrane and lysosome distributions show the ‘expected’ behavior with varying osmolarities, cytoskeletal elements, actin and tubulin, play an active role in determining the cell morphologies to the changing NaCl concentrations with the whole cell trends observed for the majority of the parameters being translated for cytoskeletal elements, actin and tubulin. While our data indicate uniformity in the distribution trend (existence of basolateral polarity) of membrane-associated glycoproteins, this observation is distinct from the mechanical stress experienced by the lipid bilayer during osmotic swelling/shrinkage. The fluidity and lateral mobility of membrane components likely enable homogeneous redistribution of glycoproteins despite transient tension [47, 48]. Future studies combining tension-sensitive probes (e.g., fluorescent lipid tension sensors) with osmotic stress could resolve localized mechanical changes undetected by static glycoprotein markers [49]. Nucleus, mitochondria and endoplasmic reticulum appear to be independent of the morphological changes appearing at the whole cell level. Our study shifts the focus from whole-cell osmotic responses to subcellular organelle dynamics, revealing that nucleus, mitochondria, and ER exhibit osmolarity-driven redistribution patterns independent of global morphology (Fig. 6). These findings challenge the assumption that organelle positioning passively follows whole-cell shape changes, instead highlighting active, organelle-specific adaptation mechanisms.

Our findings on reduced cell membrane and lysosomal pleomorphism with increasing osmolarity are specific to the tested range of 190–390 mOsm/L, which represents mild osmotic stress. Extreme conditions (e.g., <150 mOsm/L or >500 mOsm/L) may induce non-linear responses, such as membrane rupture or apoptosis, which could alter or reverse these trends [4, 14, 34, 50]. This study focused on osmolarities that permit cell survival and regulatory adaptation. While our results provide insights into subcellular responses under mild stress, extrapolation to extreme osmotic conditions should be approached cautiously, as pathological outcomes (e.g., lysis, apoptosis) may dominate in such scenarios.

While responses of several organelles (including nucleus) to osmotic stress are well-documented, our study uncovers *organelle-specific redistribution patterns* that are independent of global morphology. For example, mitochondria exhibit basolateral polarization in hypertonic conditions (Fig. 5C), likely optimizing ATP supply for ion transport, while ER-nucleus counterbalancing in hypotonic stress (Fig. S1) reflects mechanical trade-offs during swelling. These findings redefine osmotic adaptation as a *subcellular orchestration* rather than a passive whole-cell response. Our octant-based spatial analysis provides a quantitative framework to dissect organelle heterogeneity, advancing beyond traditional qualitative or 2D approaches. This methodology, combined with shape-based subpopulation analysis, reveals how cells balance structural integrity with functional demands under stress.

Cellular morphologies are transient features, meaning that the changes in morphological features are highly dynamic and vary over short intervals of time. We have accumulated the image data at random time points post a 30-minute treatment up to 45 min and proceeded further based on the assumption that the average of the ensemble will account for all the variations occurring with the window of observation points [51]. Further we acknowledge the limitation of resolution with the present technique which does not allow the accurate measurement of size of the organelles. While our study provides a detailed quantitative analysis of subcellular morphological changes in response to osmotic stress, we acknowledge that the accuracy of our results could be influenced by various image acquisition parameters, such as laser intensity, temperature, and photobleaching. To mitigate these effects, we normalized the average pixel values (APVs) for each field and maintained consistent imaging conditions across all experiments. However, since this is a pioneering study trying to study a biological phenomenon from a different perspective in cells in their native hydrated condition, similar analyses can be conducted using better high throughput techniques.

**Fig. 7:**
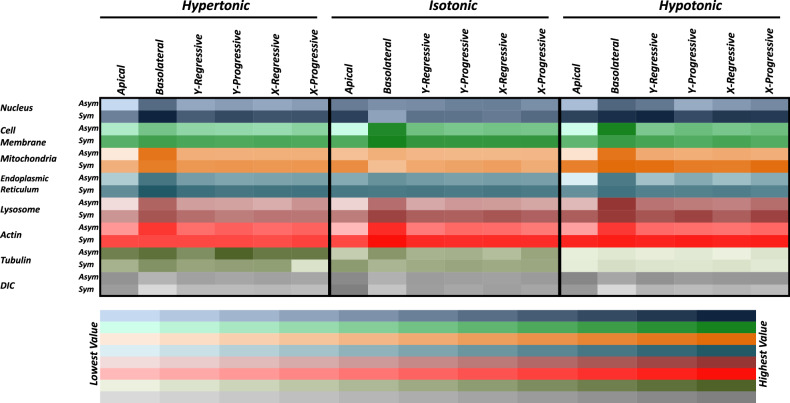
Heatmap of spatial parameter variations across osmotic conditions and organelles. Heatmap showing the variations in average pixel values between segments along 3 directions, namely, *z*- (apical and basolateral), *y*- (regressive and progressive), and *x*-axes (regressive and progressive) of asymmetric (Asym) and symmetric (Sym) of whole cell (DIC) and 7 organelles imaged (nucleus, cell membrane, mitochondria, endoplasmic reticulum, lysosome, actin and tubulin) in hypertonic, isotonic, and hypotonic conditions.

Our work bridges biological discovery and methodological innovation: while uncovering organelle-specific osmotic adaptations, we provide a reproducible pipeline for quantifying subcellular heterogeneity, applicable to diverse environmental stressors. The rigorous image analysis employed in this study to understand the effects of mild osmotic conditions on the cellular and intracellular architecture quantitatively will further assist in gaining a better understanding of this phenomenon which can then be extended to the field of tissue engineering to enhance cell survival and proliferation [41]. While our study captures spatial distribution changes at steady-state (30–45 min post-treatment), cellular responses to osmotic stress are dynamic and phase-dependent. Immediate volume changes (seconds to minutes) are followed by cytoskeletal reorganization and organelle repositioning, eventually stabilizing into the patterns observed here [10, 30, 44]. For example, the basolateral actin polarity in asymmetric cells (Fig. 5F) likely reflects cytoskeletal reinforcement during volume recovery, a process initiated within minutes of stress [23]. Similarly, the transient clustering of lysosomes observed in hypotonic conditions (Fig. 5E) may resolve over time as cells equilibrate. Future studies could employ more advanced imaging techniques, such as super-resolution microscopy or live-cell imaging, to further validate our findings and provide additional insights into the real-time dynamic responses of cells and organelle positioning and morphology to osmotic stress. Further studies could explicitly link adhesion strength (e.g., traction force microscopy) to morphological changes under osmotic stress. A collation of advanced real-time imaging techniques which allow rapid three-dimensional imaging and the present image analysis methodology along with application of machine learning to automate the cell-detection process will further enhance the precision and accuracy of the results giving the researchers a four-dimensional understanding of cellular architectural dynamics in response to various environmental stimuli. Further the image analysis methodology developed to quantify cellular and subcellular morphological changes, can be applied to a wide range of research areas such as studying the cellular responses to other types of environmental stimuli or drug treatments. Furthermore, the findings from this study may have implications for drug development and toxicity testing. Understanding how not only the whole cell but also the various components of the cells respond to osmotic stress can help researchers assess the safety and efficacy of pharmaceutical compounds and predict their effects on cellular morphology and function with higher precision. This work not only provides a new outlook to study mild variations in extracellular osmotic environments but also puts forward an image analysis method that can be used to develop the spatial distribution patterns as shown in Fig. 7 to study minute variations in the cellular architecture owing to various treatments. In conclusion, our work unravels the intricate interplay of extracellular environment and morphological diversity at both the cellular and subcellular levels. As we still continue to contribute to the knowledge of the balancing phenomenon of osmosis, this study provides a stepping stone for further in-depth research in understanding the molecular intricacies that govern cellular responses to different stimuli.

## Method

### Cell culture

Murine macrophage-like RAW264.7 cells were cultured in T25 Nest cell culture flasks in complete media comprising of Dulbecco’s Modified Eagle Medium (Gibco, Cat. No.: 12430-054), 5% Fetal Bovine Serum (Gibco, Cat. No.: 10270106), and 1% Penicillin-Streptomycin (5000U/ml) (Gibco, Cat. No.: 15070063) in 5% CO_2_ at 37 °C. The cells were passed using 0.25% trypsin (Gibco, Cat. No.: 25200072). At passage 4, the cells were counted on a hemocytometer after trypsinization, and 2 ml suspension (~50,000 cells + complete media) was placed in a 19 mm coverslip-containing 35 mm petri dish. Cells were allowed to attach to the coverslip and grow at 37 °C in 5% CO_2_ for 12 h. This timeframe ensures robust adhesion while avoiding over-confluence, as RAW264.7 macrophages typically exhibit firm attachment within 4–6 h under standard culture conditions.

### Organelle-specific fluorescence staining

The staining of the seven sub-cellular components was carried out using well-established commercially available organelle-specific fluorescent dyes. Nuclei of the cells were stained with 15 µg/ml Hoechst 33342 (ThermoFisher Scientific, Cat. No.: H21492) for 15 min at room temperature in 1x PBS. Cell membrane staining was carried out using 5 µg/ml Wheat germ agglutinin fluorescein conjugate (ThermoFisher Scientific, Cat. No.: W834) for 30 min at room temperature in 1x HBSS buffer. For mitochondrial staining, cells were incubated at room temperature in serum-free DMEM containing 50 nM MitoTracker Orange CMTMRos (ThermoFisher Scientific, Cat. No.: M7510). For staining endoplasmic reticulum, cells were kept for 30 min at 37 °C in 1x HBSS solution containing 100 nM ERTracker Blue/White DPX (ThermoFisher Scientific, Cat. No.: E12353). Cells were incubated with 1x PBS containing1µM LysoTracker Deep Red (ThermoFisher Scientific, Cat. No.: L12492) for 30 min at 37 °C to label lysosomes. For labeling actin, cells were stained using BODIPY Phalloidin (ThermoFisher Scientific, Cat. No.: B3475) (30U) for 30 min in PBS after fixation with pre-chilled absolute ethanol (pre-chilled at −20 °C) for 5 min at −20 °C (EMSURE® ACS,ISO,Reag. Ph Eur, Cat. No.: 1.00983). Cells were stained using 1 µM Paclitaxel Oregon-Green 488 (ThermoFisher Scientific, Cat. No.: P22310) for 90 min in serum-free media at 37 °C to fluorescently label tubulin.

### Tonicity Treatment

The tonicity treatments were performed after fluorescently labeling different organelles so that the staining media such as PBS and HBSS would not alter the effects of the tonicity treatment. To accurately analysis the cause-effect relation in the treatments, we chose to alter the concentration of only one salt, NaCl, in the most widely used buffer for biological systems, Phosphate Buffer Saline. An isotonic PBS has 137 mM NaCl. We prepared mildly hypertonic PBS with 175 mM NaCl concentration and mildly hypotonic PBS with 75 mM NaCl concentration so as not to create drastic morphology changes. Since the treatment buffers were only mildly tonic, the treatment time is set to be 30 min at room temperature away from light to avoid the loss of fluorescence of the fluorescently labeled organelles (Fig. 1B). All treatments were performed on cells in the log phase, ensuring comparable adhesion states across conditions. The circularity threshold ( ≥ 0.8 for symmetric cells) was applied post-treatment to categorize morphology after osmotic stress.

### Image data acquisition

Cells were treated with osmotic PBS solutions for 30min at room temperature, after which the coverslip-adherent fluorescently labeled cells were inverted over the slide, having 10µl respective treatment buffers as the mountant, and imaged. Image acquisition commenced within 5 min post-treatment and was continued till 45 min post-treatment, capturing steady-state morphological adaptations. The imaging of the samples was done in an FV1200 Olympus Confocal microscope in UPlanSApo 60x oil objective (N.A. 1.35), and images were captured using Photometrics® evolveTM Delta camera. The fluorescence was obtained using 405 nm, 473 nm, and 548 nm laser at 30%, 44%, and 48% transmissivity and 2µs/Pixel frame rate. Unstained cells were used as a negative control to set imaging parameters. Cells were imaged across multiple fields of view and experimental replicates to avoid selection bias. Some fields had uneven cell densities due to stochastic distribution during seeding. The image data was exported in .tif format for further image processing and analysis. To minimize the effects of photobleaching, laser intensity was kept low (30%, 44%, and 48% transmissivity for 405 nm, 473 nm, and 548 nm lasers, respectively), and images were captured immediately after staining. All imaging was performed at room temperature (25 °C) to avoid temperature fluctuations that could affect cell morphology.

### Image analysis and quantification

The image data comprised of different fields of duplicate treatment samples. For analysis, the .tif image files were imported into MATLAB 2021a, and the middle image of the Z-section was used to define ROI manually based on the assumption that the best-focused image in the Z-section will be the center image. If *N* is the number of DIC images in a Z-section, then the *N*/2nd image is the middle image if *N* is even and (*N* + 1)/2nd image when *N* is odd. ROI was defined around the cell boundary, the array was saved, and the centroid and circularity value of each ROI were calculated. The circularity value of 0.8 was set as the threshold value. The cells with circularity values ≥ 0.8 were categorized as symmetric and those with values <0.8 were categorized as asymmetric. This categorization was based on a 1971 study which reported that cells with circularity value greater than 0.79 had circular shape [33]. Cells categorization as *symmetric* (circularity ≥0.8) or *asymmetric*(circularity <0.8) is based on morphology and do not refer to genetically or functionally distinct cell types. This classification reflects transient shape changes under osmotic stress, not inherent biological differences. Pixel coordinates and corresponding pixel values were determined. The segregation was done in all three directions with 0.1 µm step size between *XY* planes along the *z*-axis and 0.4 µm distance between *YZ* and *XZ* planes along the *x* and *y* axes, respectively, and into the eight octants around the centroid of single-cells Z-stack. Data was normalized for each field to remove any technical variations, such as transmissivity power of the laser, offset, gain, PMT (Photomultiplier Tube) voltage, and other parameters, including handling errors during the staining process [52]. The highest APV from the octant data for a field was equated to one, and the other values were calculated accordingly using the formula

$$Normalized\,Average\,Pixel\,Value=\frac{Average\,Pixel\,Value}{Highest\,Average\,Pixel\,Value}$$

Subsequently the distribution of APVs along the 3 axes was plotted after segregating the 3D space into apical (Octants 1–4) and basal (Octants 5–8) sections, Progressive and Regressive sections along x and y axes (distribution of space shown in Fig. 1A). The population distribution for APVs in each octants were plotted after frequency normalization to determine the APV distribution uniformity in a population in different osmotic conditions. Quantitatively, it was determined by calculating a common area under the frequency distribution curve using Python code (Supplementary Information). Since cell counts varied between treatment groups this variability in numbers was accounted through normalization and statistical validation.

The quantification of cell neighbors was carried out by labeling individual cells as either asymmetric or symmetric based on circularity index and counting the number of cells (along with subset category) with a particular number and type of neighbor. A cell was deemed a neighbor of the cell under observation when their boundaries were observed to be in contact.

### Statistical analysis

All data is shown in Mean ± SE. A 2-tailed unpaired Student’s *t* test was conducted on the processed data to derive the significance of the differences seen in the dataset in Microsoft Excel. Only the *p*-values less than 0.05 were considered significant.

## Supplementary information

Supplementary material

## Data Availability

All the data and codes used for analysis are provided in Supplementary Information.

## Code availability

All the data and codes used for analysis are provided in Supplementary Information.
