# Supplementary material for: Non-uniform impact of extracellular osmotic variations at subcellular level
Source: Cell Death Discov. 2025 Aug 27;11:410. doi: 10.1038/s41420-025-02703-6 (PMC12381040; doi:10.1038/s41420-025-02703-6)
Supplement: Supplementary file 1 — Supplementary material [file 41420_2025_2703_MOESM1_ESM.pdf]

Supplementary Information

Non-uniform impact of extracellular osmotic variations at subcellular level

Pragya Singh ‡, Aditya Mittal ‡,*

Affiliations: ‡ Kusuma School of Biological Sciences, Indian Institute of Technology Delhi, Hauz Khas, Delhi, India, 110016

*corresponding author (amittal@bioschool.iitd.ac.in)

Table of Contents

S.No.	Content	Page No.
1.	Tables S1-S5- p-values of Student's T-test	3-7
2.	Table S6 – Raw APVs of fluorescent images in hypertonically treated RAW264.7 cell population	8-27
3.	Table S7 - Raw APVs of DIC images corresponding to fluorescent images of organelles in hypertonically treated RAW264.7 cell population	27-41
4.	Table S8 - Raw APVs of fluorescent images in isotonically treated RAW264.7 cell population	41-60
5.	Table S9 - Raw APVs of DIC images corresponding to fluorescent images of organelles in isotonically treated RAW264.7 cell population	60-73
6.	Table S10 - Raw APVs of fluorescent images in hypotonically treated RAW264.7 cell population	73-91
7.	Table S11 - Raw APVs of DIC images corresponding to fluorescent images of organelles in hypotonically treated RAW264.7 cell population	91-104
8.	Fig. S1 – Lateral distributions of APVs	105
9.	Fig. S2 – Frequency distributions of APVs in Octants	106
10.	Python Code for calculating area under the curves from Figure S2	107

Table S1: p-value of Student's T-test of apical and basolateral, and regressive and progressive sections around central planes in z-stacks of different organelles in hypertonic RAW264.7 population

	Z- Polarity		Y-Polarity		X-Polarity	
	Asymmetric	Symmetric	Asymmetric	Symmetric	Asymmetric	Symmetric
DIC	2E-05	3E-47	0.5	1	0.5	0.2
Nucleus	8E-10	2E-16	0.6	0.09	0.6	0.9
Cell Membrane	5E-05	0.02	0.8	0.7	0.3	0.6
Mitochondria	1E-20	5E-15	0.9	0.7	1	0.9
Endoplasmic Reticulum	2E-23	7E-18	0.4	0.3	0.7	0.8
Lysosome	1E-15	8E-08	0.8	0.2	0.1	0.9
Actin	2E-11	0.9	0.4	0.9	0.5	0.2
Tubulin	0.6	0.003	0.2	0.6	1	0.6

Table S2: p-value of Student's T-test of apical and basolateral, and regressive and progressive sections around central planes in z-stacks of different organelles in isotonic RAW264.7 population

	Z- Polarity		Y-Polarity		X-Polarity	
	Asymmetric	Symmetric	Asymmetric	Symmetric	Asymmetric	Symmetric
**DIC**	3E-10	4E-69	0.7	0.02	0.4	0.09
**Nucleus**	0.2	1E-21	0.5	0.9	0.3	0.1
**Cell Membrane**	9E-29	3E-05	0.5	0.9	0.8	0.9
**Mitochondria**	0.05	2E-16	1	0.4	1	0.03
**Endoplasmic Reticulum**	8E-04	0.7	0.5	0.5	0.8	0.4
**Lysosome**	9E-17	6E-06	0.2	0.9	0.4	0.8
**Actin**	3E-14	2E-10	0.4	0.7	0.5	0.7
**Tubulin**	0.003	0.04	0.9	0.7	0.2	0.9

Table S3: p-value of Student's T-test of apical and basolateral, and regressive and progressive sections around central planes in z-stacks of different organelles in hypotonic RAW264.7 population

	Z- Polarity		Y-Polarity		X-Polarity	
	Asymmetric	Symmetric	Asymmetric	Symmetric	Asymmetric	Symmetric
**DIC**	1.00E-06	1.00E-36	0.2	0.3	0.9	0.2
**Nucleus**	1.00E-06	0.2	0.005	0.003	0.4	0.8
**Cell Membrane**	2.00E-26	9.00E-04	0.4	0.3	0.5	0.3
**Mitochondria**	1.00E-22	0.01	0.8	0.08	0.7	0.06
**Endoplasmic Reticulum**	1.00E-38	1.00E-06	0.03	0.8	0.06	0.3
**Lysosome**	4.00E-11	0.02	0.7	0.3	0.3	0.2
**Actin**	2.00E-14	0.5	0.7	0.6	0.5	0.8
**Tubulin**	0.7	0.2	0.7	0.9	0.3	0.5

Table S4: p-value of Student's T-test of whole set, and asymmetric and symmetric shape RAW264.7 population subset between Hypertonic-Isotonic (He-I), Hypotonic-Isotonic (Ho-I) and Hypertonic-Hypotonic (He-Ho) treatment sets

	Whole set			Asymmetric			Symmetric		
	**He-I**	**Ho-I**	**He-Ho**	**He-I**	**Ho-I**	**He-Ho**	**He-I**	**Ho-I**	**He-Ho**
**DIC**	4.1E-15	1.2E-06	8.5E-03	0.23	0.11	9E-03	2.9E-16	1.7E-12	0.56
**Nucleus**	0.00	0.00	0.03	0.03	0.36	0.22	0.00	0.00	0.00
**Cell Membrane**	0.00	0.18	0.13	0.01	0.88	0.01	0.00	0.00	0.96
**Mitochondria**	9E-05	5E-19	1E-06	0.28	0.12	0.73	8E-04	4E-22	1E-10
**Endoplasmic Reticulum**	0.07	0.02	0.00	0.6	0.00	0.6	0.17	0.06	0.00
**Lysosome**	0.05	0.00	0.00	0.96	0.00	0.00	0.01	0.05	0.00
**Actin**	0.00	0.8	0.00	0.35	0.63	0.57	0.00	0.11	0.00
**Tubulin**	0.06	0.00	0.00	0.00	0.00	0.00	0.43	0.00	0.00
**Total Pixel Number**	0.00	0.00	0.00	0.00	0.00	0.00	0.00	0.00	0.00

Table S5: p-value of Student's T-test of average pixel values and total pixel numbers in asymmetric and symmetric shape subsets of RAW264.7 population in hypertonically, isotonically and hypotonically treated RAW264.7 population

Treatment Condition	p-values		
	Hypertonic	Isotonic	Hypotonic
Nucleus	9.3E-11	0.04	6.7E-14
Cell Membrane	1.6E-13	1.7E-09	3.9E-04
Mitochondria	8.7E-04	1.2E-03	9.4E-14
Endoplasmic Reticulum	1.4E-16	1.5E-14	1.9E-18
Lysosome	3.6E-05	1.5E-10	8.7E-04
Actin	1.1E-03	4.1E-11	2.0E-14
Tubulin	9.4E-03	0.46	0.47
Whole Cell	1.5E-07	0.21	1.8E-15
Total Pixel Number	1.2E-93	1.3E-175	1.4E-122

Table S6: Raw APVs of fluorescent images in hypertonically treated RAW264.7 cell population

	Circularity	*Octant 1*	*Octant 2*	*Octant 3*	*Octant 4*	*Octant 5*	*Octant 6*	*Octant 7*	*Octant 8*
Nucleus									
Field 1									
1	0.14	0.29	0.37	0.07	0.30	1.76	4.73	1.36	2.83
2	0.20	0.68	0.87	0.06	0.54	2.55	2.63	0.78	3.91
3	0.96	1.85	2.26	1.76	2.46	4.16	4.05	3.47	5.52
4	1.00	0.92	0.69	0.75	0.67	6.34	4.26	5.26	5.37
5	1.00	0.18	0.21	1.13	0.61	3.38	1.69	4.17	5.70
6	0.75	0.43	0.19	0.13	0.16	6.03	4.30	2.70	3.39
7	0.63	0.55	0.28	0.27	0.12	7.17	3.26	2.51	1.20
8	0.88	2.26	0.47	0.84	1.36	6.26	2.20	2.00	4.14
9	0.97	0.29	0.33	0.66	0.58	1.47	2.40	5.24	6.11
10	0.99	0.68	0.64	1.82	2.23	1.68	1.51	3.77	5.70
11	1.00	1.88	1.13	0.53	0.97	4.59	3.84	1.95	2.58
12	0.99	3.72	1.27	1.99	2.40	5.97	2.83	5.24	6.04
13	0.88	1.11	2.23	0.78	0.89	3.38	5.61	3.05	3.39
14	0.94	1.01	1.35	1.51	1.32	2.81	4.44	4.39	2.88
15	0.96	1.08	3.24	1.10	1.30	1.88	4.07	3.45	1.86
16	0.98	4.55	2.43	1.10	4.00	7.80	2.99	1.16	4.52
17	0.99	1.83	1.11	0.47	1.04	4.78	1.79	2.84	8.33
18	1.01	0.61	0.79	2.59	1.46	1.18	1.99	7.55	6.46
19	1.00	3.69	2.44	2.55	1.59	5.77	2.80	4.40	1.53
20	0.99	0.79	0.06	0.29	1.30	3.18	1.42	3.83	6.22
Field 2									
1	0.96	5.06	4.40	7.12	7.55	2.24	1.40	2.19	4.02
2	0.97	1.55	1.44	7.54	5.02	1.02	1.12	5.59	3.21
3	0.98	3.17	11.18	8.43	3.43	1.32	4.63	5.00	1.80
4	0.93	3.00	2.41	1.94	1.54	3.00	5.05	5.02	1.88
5	0.97	2.88	1.72	1.98	2.48	5.32	1.68	3.73	7.70
6	1.00	2.13	1.97	1.48	1.80	7.36	6.30	3.47	1.82
7	0.99	2.80	8.03	4.68	3.50	2.78	4.37	6.08	6.19
8	1.01	5.83	6.43	5.40	9.64	3.31	4.06	5.87	6.97
9	0.92	2.93	3.04	2.96	5.65	5.54	3.48	2.11	6.78
10	0.71	2.94	2.31	1.56	1.74	4.97	2.71	3.68	3.90
11	0.98	5.36	7.74	6.07	3.70	2.88	5.01	4.71	2.33
12	0.60	3.45	1.98	1.44	2.16	6.59	2.31	1.53	5.05
13	1.01	1.25	3.22	4.29	1.39	1.76	6.29	8.02	3.35

14	0.97	6.37	1.70	5.28	10.66	3.19	0.86	3.75	5.97
15	0.97	7.59	9.79	3.50	1.62	7.23	6.14	2.77	1.77
16	1.01	6.62	9.36	12.44	7.03	1.78	1.74	5.19	4.02
17	0.91	5.20	2.02	1.72	3.75	5.36	2.04	1.43	6.48
18	0.40	1.84	1.03	1.74	2.07	3.32	1.55	2.16	4.41
19	0.77	1.97	4.02	1.38	0.60	2.64	5.02	2.71	1.32
20	0.94	2.90	5.87	3.54	0.82	3.16	5.93	3.87	1.04
21	0.96	1.45	6.67	6.61	0.85	0.66	5.60	4.90	0.52
22	0.65	1.63	1.72	1.77	3.02	1.21	1.19	2.54	5.52
23	0.64	0.94	1.47	1.01	1.51	1.96	2.10	2.30	4.65
24	0.54	2.90	1.42	0.15	0.56	3.45	1.20	0.68	1.98
25	0.95	8.09	8.57	3.89	4.56	3.84	2.93	1.37	2.28
26	0.92	0.96	1.40	6.26	6.79	0.81	1.33	3.35	3.63
27	0.98	1.74	1.70	3.77	5.56	0.93	0.91	4.29	4.97
Field 3									
1	0.89	5.56	7.04	10.25	5.76	3.80	7.11	9.26	3.67
2	0.97	4.74	1.06	8.18	8.07	5.18	1.67	9.43	9.62
3	0.97	3.46	3.60	6.36	6.13	10.62	8.66	11.10	10.50
4	1.00	2.76	1.96	2.72	6.10	8.05	8.53	7.93	11.36
5	0.86	7.89	6.73	1.62	1.66	12.04	8.15	2.94	5.46
6	0.77	4.31	2.12	4.62	4.63	7.45	2.29	5.21	8.90
7	0.97	3.95	3.27	3.41	3.54	7.82	7.21	8.52	6.31
8	0.93	3.02	2.94	8.69	6.70	4.04	3.00	10.64	8.93
9	0.95	6.38	2.60	4.03	7.75	7.02	3.90	6.58	11.44
10	0.94	2.28	3.81	3.89	1.47	3.45	7.53	8.87	5.31
11	0.90	3.32	1.61	4.95	10.59	8.99	1.41	3.13	12.84
12	0.99	2.35	8.06	9.91	2.24	4.22	10.29	12.39	5.21
13	0.82	3.83	3.76	4.04	7.93	3.88	2.27	3.82	12.49
14	1.00	10.88	3.51	10.29	17.19	5.49	1.92	5.33	12.06
15	0.78	7.07	5.61	16.15	14.62	8.83	6.66	7.86	8.73
16	0.99	13.17	9.81	5.16	8.11	13.88	7.72	1.97	3.81
17	0.97	3.79	3.93	5.30	7.79	2.80	1.77	5.40	9.80
18	1.00	3.23	2.57	3.44	5.09	6.41	6.29	5.94	7.30
19	0.94	3.21	3.97	5.68	4.22	5.12	6.10	5.49	6.28
20	0.83	4.85	5.95	4.23	6.29	6.65	12.89	4.83	5.52
21	0.68	3.84	3.69	6.65	5.78	5.14	2.94	4.52	7.95
22	0.84	2.16	4.92	3.22	2.43	2.80	9.61	8.54	2.08
23	0.92	3.65	8.24	5.61	1.82	5.44	9.31	5.43	1.98
24	0.75	1.11	4.30	4.03	3.66	1.11	4.93	9.47	5.58
25	0.60	0.82	2.87	3.25	2.15	1.58	3.27	6.76	4.80
26	0.45	0.95	4.24	1.65	0.87	1.55	8.30	3.07	2.07
27	0.95	2.55	5.07	6.09	2.30	3.50	6.55	6.60	3.77
28	1.00	6.87	11.09	5.11	2.35	4.65	10.66	5.92	1.73

29	0.98	3.87	5.79	3.68	1.37	3.34	5.80	4.20	2.28
30	0.98	7.36	7.80	10.57	6.19	3.60	5.62	4.26	2.20
31	0.98	3.77	6.34	3.25	1.62	5.10	7.91	6.12	3.07
32	1.02	6.81	1.94	6.49	11.02	7.95	2.25	5.09	7.89
33	0.93	2.25	2.43	1.83	1.82	6.89	10.11	3.00	4.97
34	0.91	1.83	6.51	3.96	2.45	3.50	7.98	9.18	7.26
35	0.91	3.85	3.12	2.43	3.58	5.72	7.23	3.17	5.12
36	0.94	2.49	2.13	3.45	2.98	3.43	4.98	10.72	7.47
37	0.96	4.20	6.82	6.36	2.60	5.92	10.08	6.76	2.38
38	0.96	7.39	7.28	6.14	5.34	5.48	2.58	2.27	3.84
39	0.33	2.02	2.37	3.51	2.97	1.61	1.90	7.02	7.22
40	0.96	7.50	6.92	2.63	2.94	8.72	5.74	6.18	7.59
41	0.58	3.62	2.11	2.85	2.74	6.97	2.12	4.49	6.44
42	0.51	1.06	3.14	2.19	0.76	3.17	7.63	12.61	1.24
Field 4									
1	0.62	0.13	0.13	1.42	1.22	0.80	1.15	3.73	4.16
2	1.00	0.77	0.67	0.79	1.70	5.10	1.91	5.24	9.69
3	0.96	1.04	2.06	2.14	2.78	2.36	5.78	6.30	6.36
4	0.99	1.91	3.59	4.25	3.17	2.88	3.25	5.23	5.55
5	1.00	3.95	2.78	2.52	3.15	6.71	4.45	2.27	5.88
6	0.95	0.71	0.52	0.94	0.67	4.76	5.01	7.05	4.04
7	0.97	1.00	1.09	1.96	2.03	4.82	5.81	6.17	6.29
8	1.01	4.11	1.78	1.16	2.02	8.43	9.46	6.65	3.14
9	0.97	0.53	1.93	2.45	1.07	0.98	6.37	7.31	2.53
10	0.96	1.92	0.82	1.65	3.22	8.75	0.95	4.11	8.39
11	0.97	5.10	3.32	3.11	3.73	7.65	2.32	5.88	7.30
12	1.01	2.48	1.30	1.98	2.74	7.83	6.70	5.04	5.06
13	0.95	4.14	7.61	5.22	3.94	2.42	4.81	2.25	2.21
14	1.01	4.18	2.81	2.39	0.87	3.47	2.10	4.82	2.15
15	0.74	2.97	1.75	1.21	2.67	5.11	4.25	2.13	3.21
16	1.02	2.53	3.36	3.79	2.71	2.65	3.24	4.63	4.39
17	0.99	1.58	2.29	1.90	1.75	3.42	6.30	5.42	2.17
18	0.40	0.30	1.77	0.64	0.47	2.48	6.05	3.62	3.40
19	0.95	1.60	1.26	1.35	1.36	7.39	5.17	5.46	4.52
20	0.95	2.00	1.26	2.27	1.89	7.17	2.50	3.86	6.71
Cell Membrane									
Field 1									
1	0.27	5.07	5.65	5.96	7.35	5.84	5.72	6.91	6.46
2	0.91	13.05	15.73	11.58	11.76	9.32	9.46	8.96	7.64
3	0.19	5.11	7.74	6.54	7.77	7.06	7.44	5.92	9.28
4	0.52	15.56	8.27	6.87	12.41	8.84	6.74	6.40	6.91
5	0.21	3.71	13.70	12.86	2.82	7.89	7.13	7.21	7.00
6	0.97	9.09	12.13	10.89	11.90	6.46	7.18	7.08	6.78

7	0.77	13.40	7.23	9.42	6.97	9.99	8.53	10.07	8.02
8	1.00	16.44	10.72	12.51	10.88	13.07	9.92	9.83	8.86
9	0.97	13.43	20.50	20.64	11.89	13.18	14.86	13.41	10.47
Field 2									
1	1.02	6.26	5.87	6.78	6.23	6.72	5.33	6.54	6.44
2	0.99	8.67	9.49	10.56	10.82	7.76	9.36	9.51	8.87
3	1.01	10.83	9.49	7.63	8.72	8.62	7.34	6.46	7.23
4	0.95	8.78	8.43	10.45	10.59	7.21	7.80	9.96	9.07
5	0.74	4.89	3.69	4.16	5.08	5.53	5.26	6.68	5.84
6	1.01	5.70	6.25	5.46	5.59	6.20	7.60	6.77	7.03
7	1.02	4.50	4.61	7.08	4.88	6.34	6.26	7.84	7.96
8	0.89	7.68	6.72	5.20	5.99	7.12	7.99	8.60	7.50
9	0.34	2.95	4.10	3.10	2.46	5.66	7.53	6.62	5.47
10	0.30	4.60	2.78	2.20	2.08	6.75	4.90	4.24	3.57
11	0.99	3.15	4.58	4.72	4.06	5.81	6.80	4.65	6.12
12	0.71	5.96	6.57	3.09	3.50	7.97	8.66	5.22	7.15
13	0.96	2.87	3.35	3.60	3.52	5.70	6.00	5.74	6.56
14	0.92	5.10	4.52	3.46	4.79	7.74	7.30	6.84	7.27
15	0.96	3.92	7.24	10.38	4.15	8.55	7.15	9.50	7.64
16	0.99	6.66	4.88	7.89	6.13	7.12	7.86	7.27	7.02
17	0.98	6.27	5.20	4.57	5.03	6.19	5.96	7.57	9.07
18	1.01	5.63	4.98	5.08	5.66	5.63	4.61	4.66	5.50
19	1.00	6.07	5.60	5.84	6.68	5.08	5.21	5.41	6.39
20	0.99	6.15	5.23	4.88	5.75	5.45	4.80	4.78	4.99
21	0.62	12.69	5.02	3.61	5.94	7.68	5.69	5.72	6.91
22	0.97	6.73	6.84	5.66	6.64	5.71	6.43	5.20	4.71
23	0.99	5.31	5.12	5.23	5.70	5.99	5.83	6.99	7.00
24	0.94	5.20	4.51	4.79	5.96	6.74	5.04	5.26	5.84
25	0.95	6.33	5.20	7.74	5.67	6.18	5.92	7.39	5.86
26	0.96	6.26	4.40	6.46	5.75	6.91	5.42	7.57	6.48
27	0.85	4.38	2.86	3.07	4.18	6.54	6.21	5.89	6.20
28	0.97	3.81	4.72	4.01	5.44	5.29	5.42	5.29	6.44
29	0.95	5.45	5.93	5.31	3.80	5.67	6.00	5.02	4.86
30	0.98	5.96	4.53	4.79	5.53	7.13	7.11	7.27	5.98
31	0.91	4.46	4.45	4.28	4.77	6.51	6.99	5.86	6.03
Field 3									
1	0.83	6.21	6.62	5.27	6.43	10.02	12.43	7.65	8.19
2	0.97	5.21	4.71	3.20	5.03	6.54	7.36	6.75	10.86
3	0.93	4.17	5.10	4.29	5.17	6.81	7.97	6.47	7.56
4	0.74	3.68	7.58	8.80	4.86	7.49	7.66	8.55	7.94
5	0.99	6.83	8.98	10.05	9.25	6.13	7.34	8.07	7.39
6	0.79	5.19	5.08	4.12	5.76	6.81	6.09	4.91	7.40
7	0.97	8.58	8.21	6.03	7.02	6.11	5.90	6.34	6.37

8	0.91	6.49	10.49	7.49	7.25	5.27	7.11	5.73	5.30
9	0.92	8.85	12.24	13.13	11.16	7.57	7.61	6.00	7.49
10	0.98	14.12	13.56	12.85	9.69	8.05	8.43	9.46	7.66
11	0.70	3.96	3.90	4.70	8.05	5.28	8.66	7.56	6.64
12	0.77	6.22	5.19	3.97	5.46	7.31	9.11	7.38	7.38
13	0.98	6.28	7.19	7.26	6.97	6.44	7.10	5.87	7.53
14	0.97	6.59	5.30	6.59	8.00	7.15	5.87	6.85	6.75
15	0.95	5.44	6.84	7.89	5.03	8.81	9.69	10.89	7.23
16	0.71	6.68	3.89	3.87	3.85	8.58	9.26	7.31	8.13
17	0.98	5.85	5.96	6.64	5.06	8.62	11.19	21.89	9.08
18	0.97	5.10	4.29	6.29	6.89	13.30	6.52	10.42	17.19
19	0.63	8.16	11.31	11.22	3.61	7.61	8.96	11.49	6.00
20	0.98	4.47	6.55	9.93	5.60	5.63	7.99	8.16	7.14
21	0.93	12.30	11.15	8.52	7.68	10.50	7.61	8.17	9.91
22	0.94	3.37	6.03	11.63	4.04	7.65	9.24	8.78	8.93
23	0.28	2.57	2.64	2.27	5.61	8.46	8.59	9.21	26.96
24	0.62	3.72	3.85	2.21	3.75	6.83	7.80	7.97	7.68
25	1.00	5.46	4.50	4.40	4.36	7.07	5.71	6.79	7.58
26	0.99	3.99	4.08	3.23	3.59	7.00	7.11	7.98	7.37
27	0.76	2.91	4.24	3.51	3.98	7.33	7.42	8.79	7.65
28	0.97	4.32	4.79	4.53	5.38	8.60	10.72	11.10	9.98
29	0.99	6.72	6.75	5.52	5.24	10.08	10.54	8.42	7.56
30	1.02	4.03	4.52	3.92	5.75	6.61	8.08	5.87	6.96
31	0.97	10.93	9.73	8.32	13.28	12.39	11.94	9.99	10.09
32	0.93	3.30	4.39	7.73	3.26	6.83	7.09	7.52	6.34
33	0.97	7.09	6.46	3.24	3.79	13.29	11.41	8.93	10.43
34	0.64	3.58	2.40	2.87	4.97	10.75	8.35	9.79	12.02
35	0.98	4.77	3.95	4.01	5.21	8.71	10.09	7.43	10.36
36	0.45	4.29	4.53	3.90	6.48	6.33	6.95	8.93	9.18
Field 4									
1	1.02	10.52	12.23	9.24	11.19	17.20	24.64	16.29	13.21
2	1.02	9.19	9.43	6.56	7.56	13.16	13.28	16.48	19.91
3	0.98	6.66	6.17	7.73	4.76	10.65	10.30	8.66	6.59
4	1.01	8.60	10.51	9.99	8.61	8.27	9.79	11.36	9.64
5	0.78	11.93	14.77	8.48	6.18	18.86	15.23	14.85	12.57
6	0.98	20.14	14.70	18.22	10.76	14.09	18.78	21.91	11.67
7	0.44	13.03	5.30	2.76	8.74	19.99	12.77	9.28	14.39
8	0.99	11.76	12.25	17.43	12.30	15.07	13.33	12.65	11.70
9	0.98	15.43	12.61	8.59	11.48	12.46	12.48	11.25	13.78
10	0.46	3.18	3.40	5.82	4.00	9.27	8.12	8.48	9.71
11	1.00	18.57	32.79	21.09	11.90	15.31	18.00	13.41	16.42
12	0.96	23.30	18.33	21.17	18.68	14.38	14.24	15.15	17.60
13	0.95	20.05	32.56	19.52	10.51	17.86	16.68	13.22	15.42

14	0.59	3.63	8.06	17.52	2.05	9.79	9.37	15.80	5.89
15	0.88	16.21	19.32	14.63	22.74	15.25	12.11	17.52	23.06
16	0.91	8.56	7.50	8.07	16.15	13.73	8.42	9.47	17.84
17	0.42	5.30	2.37	5.66	18.18	15.29	10.72	10.79	18.27
18	0.28	7.22	13.23	10.89	10.33	7.03	8.79	7.88	9.65
19	0.92	10.53	10.16	7.70	6.37	15.73	13.83	11.18	10.06
Field 5									
1	0.98	17.96	23.80	24.26	34.19	12.60	15.70	17.87	20.38
2	0.52	13.42	5.38	38.90	32.36	11.87	10.80	15.59	21.84
3	1.00	62.01	43.67	42.79	44.27	35.90	28.56	24.25	26.72
4	0.44	24.51	15.50	28.92	23.10	13.70	14.45	11.44	18.48
5	0.75	31.73	39.76	17.27	11.89	13.05	22.58	16.21	13.43
6	0.49	12.24	14.24	7.98	10.08	25.56	16.02	14.67	29.99
7	0.98	29.35	29.62	22.26	25.70	25.06	21.06	21.33	23.58
8	1.01	17.18	22.81	18.39	24.16	16.46	15.23	13.26	15.44
9	0.79	12.05	11.47	7.09	9.75	17.87	12.25	13.78	19.00
10	0.67	21.93	19.81	33.92	34.46	27.10	30.49	31.15	32.70
11	0.43	32.81	19.35	35.84	14.52	25.34	23.41	18.80	14.93
Mitochondria									
Field 1									
1	0.14	1.23	1.28	0.24	1.49	17.66	26.06	13.53	34.26
2	0.20	1.20	0.99	0.10	1.08	11.08	14.24	8.32	19.10
3	0.96	7.80	9.68	8.59	7.25	25.37	20.40	30.67	26.27
4	1.00	9.83	3.89	2.91	6.40	34.64	21.68	21.98	35.69
5	1.00	0.28	0.34	1.49	0.23	15.53	27.79	23.83	11.42
6	0.75	1.45	0.84	0.79	0.85	27.28	25.77	32.60	24.97
7	0.63	0.92	0.19	0.27	0.19	32.09	13.74	20.01	21.97
8	0.88	5.19	0.93	4.53	5.32	22.50	10.11	34.15	34.71
9	0.97	5.03	2.95	3.27	3.71	33.30	22.51	10.28	18.23
10	0.99	3.26	1.54	4.59	6.48	18.73	11.64	11.20	15.83
11	1.00	5.10	3.81	5.81	6.72	11.37	7.75	20.77	18.77
12	0.99	14.94	12.74	8.21	8.09	26.35	35.39	23.59	18.48
13	0.88	6.40	5.50	4.94	12.24	20.04	17.34	17.11	35.87
14	0.94	4.37	3.52	7.88	8.17	17.09	9.41	23.84	23.71
15	0.96	9.84	12.61	10.76	8.32	7.83	7.60	7.53	6.35
16	0.98	8.04	15.99	22.99	14.77	16.04	24.99	28.57	28.25
17	0.99	5.76	3.41	2.79	3.03	17.91	21.07	26.40	12.76
18	1.01	6.34	8.57	9.52	6.90	28.75	22.62	12.22	17.57
19	1.00	18.16	12.93	10.63	20.02	18.20	21.34	18.98	28.53
20	0.99	6.22	0.48	1.97	16.95	7.38	6.81	17.93	11.77
Field 2									
1	0.96	11.45	20.54	15.27	11.20	8.48	15.57	11.81	7.18
2	0.97	10.76	9.41	7.78	7.30	4.45	2.78	2.84	1.84

3	0.98	7.24	4.36	6.43	6.57	3.34	5.55	1.70	1.40
4	0.93	4.03	2.37	3.27	3.26	4.71	3.24	3.44	4.81
5	0.97	3.31	3.38	4.02	3.38	18.04	30.29	27.97	16.87
6	1.00	2.63	1.77	1.61	2.45	26.70	17.67	24.65	37.74
7	0.99	4.22	16.17	11.17	2.62	22.81	27.95	19.93	11.97
8	1.01	30.46	14.98	7.93	23.38	20.69	12.54	7.98	13.73
9	0.92	1.71	2.59	4.29	6.29	13.83	16.09	27.49	20.89
10	0.71	2.30	2.74	0.77	0.37	20.23	26.25	13.71	9.51
11	0.98	5.32	5.73	4.66	3.67	16.32	17.62	11.45	11.94
12	0.60	3.35	2.46	0.77	1.47	10.90	17.47	11.33	9.43
13	1.01	7.71	10.27	10.92	6.85	15.37	13.29	12.04	10.76
14	0.97	18.65	12.80	8.85	12.69	15.66	13.08	9.82	8.59
15	0.97	25.56	13.58	19.20	24.53	14.16	10.34	10.75	12.21
16	1.01	22.33	19.51	16.41	12.98	20.03	20.35	15.61	10.89
17	0.91	6.61	11.52	10.81	5.51	12.58	21.57	21.78	12.95
18	0.40	6.79	0.29	1.00	4.47	20.65	6.96	9.24	15.97
19	0.77	3.32	3.96	1.98	3.69	12.63	8.89	9.22	10.94
20	0.94	11.75	7.21	6.71	8.79	8.30	5.85	7.54	8.10
21	0.96	12.17	18.24	17.30	7.46	15.88	14.50	9.48	10.32
22	0.65	2.40	2.55	1.37	1.59	11.05	11.78	4.72	4.14
23	0.64	2.73	2.84	3.21	4.94	17.03	15.46	16.35	16.33
24	0.54	6.30	1.82	0.08	2.24	10.04	6.39	3.03	9.79
25	0.95	16.55	21.65	19.32	15.58	7.34	12.79	11.02	8.09
26	0.92	2.04	2.89	1.82	1.39	1.06	0.68	0.43	1.04
27	0.98	1.15	2.11	3.53	1.64	0.61	1.61	2.30	1.24
Field 3									
1	0.89	13.58	10.91	22.30	19.22	22.69	14.67	17.24	25.97
2	0.97	24.12	20.94	22.70	22.83	20.53	22.45	18.02	16.71
3	0.97	33.60	35.50	35.56	38.54	22.89	24.36	23.48	21.41
4	1.00	17.79	19.50	18.12	22.21	11.82	18.07	15.28	12.30
5	0.86	13.31	12.90	17.30	19.57	11.89	13.59	21.00	19.24
6	0.77	2.46	14.09	2.33	2.65	6.54	13.25	7.53	5.16
7	0.97	11.26	13.26	12.96	5.11	5.35	11.60	8.16	3.58
8	0.93	12.59	13.97	12.39	7.99	11.09	14.08	9.05	8.02
9	0.95	16.55	19.35	14.30	11.37	9.37	12.52	7.63	7.19
10	0.94	4.54	5.12	5.44	8.36	9.46	5.57	6.06	11.10
11	0.90	6.64	4.01	7.82	11.35	14.08	16.99	14.77	14.89
12	0.99	7.46	6.21	4.96	7.49	3.44	1.24	1.76	2.74
13	0.82	2.10	10.36	6.24	2.93	4.40	12.78	10.22	4.38
14	1.00	14.61	22.79	11.67	14.57	13.60	16.12	6.89	8.70
15	0.78	15.15	9.73	17.40	25.47	19.20	9.89	20.74	26.97
16	0.99	4.74	7.18	8.93	6.96	1.86	2.98	4.95	5.38
17	0.97	11.01	7.84	5.88	6.81	10.37	7.52	3.68	4.49

18	1.00	9.29	19.18	11.30	11.69	5.05	9.58	7.19	6.57
19	0.94	3.20	6.26	9.43	9.54	7.81	6.97	11.69	11.16
20	0.83	7.06	5.79	5.22	12.56	13.62	7.25	11.50	17.88
21	0.68	4.10	7.65	8.78	4.13	8.19	12.21	16.39	10.70
22	0.84	1.06	1.30	4.66	4.19	4.52	10.15	11.03	16.66
23	0.92	16.58	8.05	6.10	6.97	22.45	13.18	19.52	23.76
24	0.75	1.39	3.08	3.31	2.80	8.52	15.01	6.02	9.57
25	0.60	1.42	3.71	7.77	5.33	6.81	14.16	12.33	11.00
26	0.45	1.91	4.21	1.27	3.56	5.67	6.18	8.28	14.35
27	0.95	11.34	5.10	9.98	20.27	8.41	3.25	8.05	13.65
28	1.00	2.79	0.85	3.01	3.19	1.20	1.08	0.94	0.68
29	0.98	16.14	10.33	11.40	13.17	10.84	8.22	10.75	13.23
30	0.98	14.77	8.82	9.97	16.13	12.91	11.99	17.21	18.33
31	0.98	9.82	8.41	16.96	16.13	4.24	2.75	9.99	11.24
32	1.02	10.25	11.12	7.99	9.19	6.52	10.08	4.20	5.25
33	0.93	1.97	2.75	8.20	4.01	5.47	7.31	13.65	9.08
34	0.91	8.57	10.38	11.25	6.86	16.89	13.57	13.79	15.46
35	0.91	2.57	4.94	5.76	5.47	4.29	6.66	11.38	11.90
36	0.94	4.88	6.20	5.84	6.89	13.12	11.54	9.58	9.51
37	0.96	1.92	3.14	7.69	3.81	7.19	6.67	15.40	16.58
38	0.96	6.33	6.57	7.39	5.90	1.80	3.14	1.79	1.53
39	0.33	2.81	2.36	8.03	8.08	4.34	10.40	14.42	12.76
40	0.96	6.74	4.80	6.40	5.91	14.24	15.28	12.86	15.05
41	0.58	5.09	4.49	4.08	1.83	15.30	18.41	16.82	7.72
42	0.51	0.17	9.30	2.39	0.02	19.98	31.30	20.19	5.93
Field 4									
1	0.62	5.19	8.34	9.61	11.82	19.72	25.61	28.65	31.40
2	1.00	6.36	6.50	8.12	7.68	25.00	30.86	28.15	27.61
3	0.96	6.82	5.68	4.53	8.13	27.99	21.09	17.11	21.11
4	0.99	5.40	7.14	10.75	9.60	23.81	23.80	24.62	23.95
5	1.00	9.19	5.10	2.55	4.35	7.05	8.62	13.47	8.88
6	0.95	3.77	5.71	2.62	1.78	7.41	13.78	5.22	3.79
7	0.97	2.08	3.04	3.81	2.59	6.31	4.28	5.01	4.65
8	1.01	5.68	1.03	0.83	3.15	8.34	4.11	7.28	11.19
9	0.97	4.31	2.36	2.72	6.23	8.43	4.34	4.48	9.80
10	0.96	4.34	8.07	5.36	9.45	7.06	14.61	10.25	5.39
11	0.97	5.66	7.15	1.53	3.08	6.66	9.55	4.14	4.36
12	1.01	10.26	17.52	20.25	13.32	6.85	8.91	9.19	6.53
13	0.95	11.51	13.52	14.51	12.80	19.81	22.44	20.37	18.31
14	1.01	3.30	3.57	5.43	4.67	3.73	3.04	3.41	3.65
15	0.74	2.52	1.67	2.39	6.52	3.49	2.89	4.15	5.47
16	1.02	6.04	3.59	8.01	7.72	12.19	8.61	8.99	14.26
17	0.99	2.52	3.52	3.80	3.67	10.17	9.67	12.68	13.61

18	0.40	0.61	5.62	1.43	1.08	2.59	4.97	4.23	5.72
19	0.95	5.19	5.62	3.89	3.01	8.19	9.22	7.89	3.20
20	0.95	1.94	3.43	3.53	1.73	7.84	15.88	14.75	3.81
Endoplasmic Reticulum									
Field 1									
1	0.27	1.68	2.58	1.34	1.53	3.08	4.40	2.59	2.15
2	0.91	8.15	7.14	4.96	7.50	3.28	3.44	2.66	2.95
3	0.19	1.22	2.48	2.56	2.44	2.14	2.96	3.20	3.04
4	0.52	4.93	4.25	2.44	4.87	4.10	3.54	2.23	2.42
5	0.21	1.01	6.40	5.75	0.21	3.84	4.59	5.16	1.76
6	0.97	5.48	5.10	5.80	7.25	3.52	3.47	4.04	4.12
7	0.77	5.38	3.47	3.57	3.70	4.82	3.50	4.00	4.55
8	1.00	7.64	5.43	3.80	5.13	5.89	3.66	2.59	3.20
9	0.97	4.13	8.53	8.40	4.45	4.23	5.42	5.55	3.79
Field 2									
1	1.02	2.10	1.77	1.93	2.13	2.83	2.17	2.49	2.67
2	0.99	3.45	3.16	2.66	2.30	3.16	2.84	2.65	2.34
3	1.01	3.20	2.39	1.89	2.49	3.20	2.74	2.07	2.57
4	0.95	3.63	2.96	2.60	3.48	2.70	2.64	2.49	2.88
5	0.74	1.12	1.08	1.42	1.48	1.81	1.92	2.31	2.17
6	1.01	2.01	2.73	2.11	1.76	2.63	3.65	2.94	2.72
7	1.02	1.57	2.06	2.40	1.59	2.47	3.48	3.66	2.64
8	0.89	3.43	2.67	1.84	2.27	3.36	2.81	2.85	3.05
9	0.34	0.96	1.31	1.07	0.60	2.33	3.05	2.76	1.88
10	0.30	1.90	1.03	0.56	0.49	3.36	2.19	1.75	1.39
11	0.99	1.32	1.85	1.91	1.68	2.81	3.04	3.02	3.06
12	0.71	2.13	2.46	1.10	1.25	4.07	3.50	2.31	3.04
13	0.96	1.25	1.43	1.17	1.28	3.55	3.38	2.43	2.86
14	0.92	1.68	1.59	1.15	1.55	2.97	3.04	2.50	3.35
15	0.96	1.14	1.71	2.34	1.32	2.66	2.23	3.70	2.76
16	0.99	2.48	1.74	1.88	2.26	3.75	3.34	2.50	3.26
17	0.98	5.16	3.45	2.12	2.75	2.36	2.28	2.29	2.79
18	1.01	2.84	1.91	2.16	2.94	3.16	2.23	2.43	3.13
19	1.00	2.65	2.04	1.81	2.52	2.67	2.76	2.22	2.99
20	0.99	3.29	2.28	2.39	2.76	2.85	2.72	2.66	2.55
21	0.62	2.60	1.53	1.48	1.88	3.32	2.54	2.14	2.49
22	0.97	2.61	2.12	2.14	3.21	2.41	2.58	2.55	3.43
23	0.99	3.24	2.33	2.05	2.84	3.66	2.63	2.53	3.22
24	0.94	2.11	2.30	2.39	1.86	2.73	3.03	3.11	2.13
25	0.95	2.83	2.39	3.30	2.34	2.92	2.82	3.73	2.54
26	0.96	3.66	2.47	2.40	3.02	3.73	2.78	3.13	2.98
27	0.85	1.61	0.95	1.02	1.48	2.52	2.24	2.50	2.88
28	0.97	1.60	1.59	1.30	1.64	2.57	2.59	2.97	2.99

29	0.95	2.25	2.47	1.62	1.40	2.75	2.70	2.07	2.13
30	0.98	2.33	2.03	2.66	3.24	2.84	3.28	3.36	3.64
31	0.91	2.00	1.64	1.59	2.11	3.43	3.07	2.63	3.49
Field 3									
1	0.83	1.65	1.39	1.44	1.88	2.81	3.00	3.58	3.28
2	0.97	1.49	1.23	1.12	1.71	2.60	2.82	3.21	3.81
3	0.93	1.19	2.08	1.36	1.25	2.38	4.09	2.97	2.00
4	0.74	0.71	2.10	2.52	1.73	1.83	2.57	4.26	3.25
5	0.99	2.24	2.22	2.64	3.04	2.60	2.42	2.81	3.15
6	0.79	1.76	1.96	0.87	1.15	2.63	3.37	2.31	2.27
7	0.97	4.95	4.49	2.38	2.99	2.46	2.75	1.85	1.92
8	0.91	2.24	3.40	3.47	4.29	2.29	2.59	3.14	4.30
9	0.92	2.62	3.97	3.38	3.83	2.53	3.88	2.81	3.20
10	0.98	4.22	3.35	3.24	4.12	2.78	2.43	2.70	2.62
11	0.70	1.47	1.09	1.62	2.82	2.33	2.63	3.21	3.50
12	0.77	1.96	1.23	0.83	1.48	2.79	3.16	2.54	2.52
13	0.98	1.86	2.90	3.85	2.83	2.07	2.87	3.44	3.18
14	0.97	2.18	2.17	2.95	2.86	2.26	2.60	3.83	2.72
15	0.95	2.97	2.50	2.58	1.62	3.39	3.10	3.48	2.17
16	0.71	2.61	1.04	1.29	1.41	3.07	2.11	2.91	3.20
17	0.98	2.01	1.74	1.75	1.56	3.67	3.27	3.81	3.43
18	0.97	1.85	2.69	2.82	1.43	3.33	2.79	3.48	4.08
19	0.63	2.68	2.73	2.43	1.04	3.50	2.80	3.42	2.14
20	0.98	1.63	2.41	3.10	2.15	3.53	4.01	3.63	3.39
21	0.93	3.67	2.93	1.97	2.06	3.84	3.52	3.21	3.88
22	0.94	0.97	1.95	3.46	1.04	2.67	3.91	3.65	3.03
23	0.28	0.30	0.47	0.27	0.76	2.46	3.65	2.76	4.32
24	0.62	1.65	1.44	0.32	1.06	3.22	3.91	2.42	4.14
25	1.00	1.92	1.94	2.37	1.68	2.58	2.98	5.31	3.17
26	0.99	1.57	1.49	1.19	1.52	3.02	4.40	4.50	4.00
27	0.76	0.89	1.23	1.07	1.28	3.16	3.29	4.34	3.89
28	0.97	1.34	1.52	1.39	2.06	3.29	4.50	4.42	4.15
29	0.99	2.31	2.11	2.24	2.14	3.16	4.51	5.06	4.63
30	1.02	2.34	1.88	1.88	2.73	4.15	4.28	2.72	3.62
31	0.97	2.28	1.87	1.80	3.23	3.56	3.38	3.35	4.62
32	0.93	0.84	1.08	1.77	0.97	2.22	3.35	4.57	2.95
33	0.97	1.13	1.13	0.82	1.00	3.61	3.31	3.37	3.62
34	0.64	1.10	0.55	0.54	1.05	3.15	2.66	3.32	3.40
35	0.98	1.40	1.59	1.27	1.73	3.08	3.51	2.87	3.57
36	0.45	1.65	1.70	1.22	1.86	3.03	3.70	2.87	3.16
Field 4									
1	1.02	3.03	3.34	3.32	2.75	5.98	8.05	7.28	4.45
2	1.02	2.46	2.37	2.30	3.05	6.03	4.51	6.55	7.70

3	0.98	2.66	2.35	2.92	1.71	7.48	6.62	5.81	4.36
4	1.01	4.30	4.52	5.01	4.31	5.99	3.86	5.94	5.57
5	0.78	4.12	4.94	2.33	1.93	6.79	5.32	5.55	4.71
6	0.98	4.27	4.84	5.84	2.08	4.92	8.06	6.86	3.53
7	0.44	4.58	1.04	0.51	1.66	7.26	4.09	2.98	4.35
8	0.99	4.58	5.29	7.69	4.83	6.36	6.20	6.14	5.28
9	0.98	6.10	5.95	3.50	4.70	4.48	5.67	6.14	7.30
10	0.46	0.76	0.96	1.80	1.16	3.67	4.73	5.27	5.53
11	1.00	7.04	9.78	4.54	2.47	9.03	7.93	5.47	4.72
12	0.96	9.68	5.68	6.78	6.61	6.91	4.38	6.54	8.01
13	0.95	7.35	9.82	4.10	2.14	8.93	8.06	5.46	4.35
14	0.59	1.38	3.32	5.53	0.34	4.73	6.34	7.90	3.15
15	0.88	4.44	5.97	3.14	3.37	6.58	7.94	7.94	8.39
16	0.91	5.05	2.45	3.22	8.27	7.41	3.90	4.88	9.51
17	0.42	2.45	0.51	1.97	3.80	6.16	2.92	6.06	6.38
18	0.28	1.53	2.83	1.80	2.00	3.20	4.95	4.74	4.57
19	0.92	7.07	5.74	5.89	6.28	5.87	3.96	4.52	6.58
Field 5									
1	0.98	1.69	2.58	2.42	1.73	1.47	1.86	1.98	1.70
2	0.52	1.80	0.51	2.37	2.52	1.62	1.27	2.02	2.06
3	1.00	3.37	3.04	2.72	3.03	1.90	2.20	2.08	1.75
4	0.44	2.14	1.44	1.82	1.63	1.73	1.52	1.59	1.62
5	0.75	2.36	1.72	1.60	1.00	1.46	1.72	2.15	1.82
6	0.49	1.69	2.25	1.30	1.33	2.44	2.13	2.25	2.56
7	0.98	3.46	3.56	2.42	2.61	3.01	2.85	2.78	3.05
8	1.01	2.04	1.59	1.87	2.42	2.29	1.98	2.01	2.12
9	0.79	1.65	0.96	0.81	1.33	2.17	1.62	1.60	2.13
10	0.67	1.56	1.44	1.93	1.88	2.22	2.05	2.21	2.30
11	0.43	2.72	1.37	2.76	2.01	2.40	1.70	2.14	1.84
Lysosome									
Field 1									
1	0.95	3.08	0.44	0.44	4.66	7.30	4.15	4.83	9.67
2	0.96	4.32	4.68	7.75	6.58	9.79	5.82	5.60	6.68
3	0.78	2.92	3.67	1.29	2.77	6.73	5.55	0.59	4.22
4	0.99	5.66	6.89	2.44	1.32	6.19	9.53	1.70	0.49
5	0.98	5.55	5.99	8.69	13.70	5.02	1.26	10.24	16.90
6	0.40	0.22	0.04	0.04	0.01	1.90	0.83	1.54	0.98
7	0.78	1.35	0.16	1.16	1.58	4.75	2.57	3.92	4.95
8	0.35	0.90	1.42	0.37	0.75	2.39	3.09	2.05	2.59
9	0.80	1.61	1.68	1.34	0.17	8.80	5.59	6.37	1.38
10	0.86	0.59	1.74	0.75	0.47	1.04	4.33	1.31	0.41
11	0.66	0.41	0.29	1.75	2.82	0.37	2.01	4.16	2.05
12	0.78	0.81	3.02	2.34	0.59	1.55	3.68	4.54	1.70

13	0.72	0.28	0.09	0.84	0.58	1.99	0.46	0.92	2.81
14	1.00	2.06	1.92	1.39	1.69	5.06	3.74	1.43	1.21
15	1.00	1.53	0.29	1.90	4.85	4.16	0.31	2.39	9.84
16	0.55	0.45	0.04	0.09	0.18	2.88	1.22	2.83	1.39
17	0.85	0.43	0.02	0.08	1.76	0.91	0.01	0.01	3.56
18	0.98	1.06	3.94	10.41	4.67	0.35	2.13	7.82	6.01
19	0.98	13.53	19.81	3.81	1.73	5.75	6.97	0.83	0.82
20	0.37	3.86	4.26	6.45	5.40	4.52	5.11	0.94	1.45
21	0.26	0.06	0.00	0.00	0.70	0.79	0.94	0.10	2.59
22	0.37	0.03	0.20	0.55	0.03	0.27	0.69	4.39	0.76
23	0.35	0.20	0.42	0.28	0.14	1.53	5.62	1.28	1.90
24	0.54	1.61	0.69	2.53	0.92	4.52	3.33	5.71	4.13
25	0.31	2.06	0.21	0.35	0.99	5.85	2.83	1.04	1.98
26	0.96	1.07	2.12	2.46	3.01	1.84	0.58	1.18	4.91
Field 2									
1	1.01	1.56	2.36	0.36	0.36	5.34	7.65	5.57	3.01
2	1.01	0.36	0.91	0.64	0.05	1.44	14.88	18.11	1.98
3	0.99	2.61	0.36	1.51	1.15	13.47	1.63	3.06	16.72
4	0.87	2.45	0.40	1.13	2.61	12.08	1.92	1.05	6.32
5	0.57	0.14	0.00	0.06	0.73	8.52	1.17	1.37	13.82
6	0.48	0.19	0.23	0.01	0.08	2.36	9.49	2.04	1.99
7	0.54	0.36	0.00	0.00	0.78	2.02	0.80	1.20	4.30
8	0.46	0.00	0.00	0.00	0.01	2.54	3.12	1.34	4.30
9	0.90	0.33	0.47	0.22	0.36	5.31	4.94	0.74	0.83
10	0.95	0.08	0.16	0.10	0.18	0.95	7.66	5.64	0.86
11	0.97	0.66	0.23	2.99	4.15	1.19	0.22	7.64	10.81
12	0.98	3.31	0.84	0.01	0.09	10.89	6.62	0.04	0.58
13	1.00	2.68	1.77	4.39	4.11	5.79	2.98	5.50	14.19
14	0.88	1.44	0.60	0.13	0.27	1.01	4.52	3.82	2.81
15	0.51	0.62	0.05	1.12	0.63	2.41	0.25	1.23	1.36
16	0.91	0.18	0.41	0.39	0.20	0.24	1.67	1.81	0.77
17	0.92	2.17	2.83	1.67	0.76	2.24	4.17	7.46	4.43
18	0.97	3.93	5.73	8.32	2.59	0.64	2.38	2.19	0.23
19	0.56	0.00	0.01	0.13	0.06	3.55	6.47	1.63	2.89
20	0.43	0.00	0.01	0.01	0.06	0.53	0.74	2.34	3.91
21	0.98	3.48	1.09	0.59	0.57	4.15	3.51	3.36	0.80
22	0.59	0.00	0.00	0.00	0.00	4.14	5.92	1.36	12.78
Field 3									
1	0.26	5.69	0.52	0.00	1.33	8.09	11.94	0.64	1.71
2	0.96	7.11	5.23	3.83	4.69	2.62	0.61	3.05	3.49
3	0.95	18.94	19.08	8.46	3.42	8.92	10.31	2.55	0.92
4	0.99	8.19	10.16	4.24	4.15	16.08	13.93	11.04	10.47
5	0.88	0.02	0.02	0.02	0.01	7.53	6.83	2.36	8.30

6	0.88	0.05	0.02	0.00	0.00	9.07	8.39	1.58	7.32
7	0.40	2.21	4.43	1.36	6.30	11.61	4.15	3.48	25.01
8	0.98	3.33	10.60	4.90	0.43	14.28	6.57	1.61	4.92
9	0.93	8.61	3.39	3.99	4.38	2.71	14.01	17.04	4.40
10	0.96	0.50	7.37	11.31	5.70	0.27	6.69	14.60	5.66
11	0.69	1.50	2.39	2.07	5.46	2.04	1.65	1.14	8.40
12	0.96	4.23	3.24	1.74	2.18	11.10	11.46	7.39	3.82
13	0.42	0.00	0.06	1.14	0.01	0.06	3.20	7.68	0.06
14	0.47	0.17	0.01	0.01	0.27	4.65	0.41	0.61	8.85
15	1.01	2.28	2.44	1.07	1.15	3.11	4.56	1.53	0.43
16	0.79	0.25	0.00	0.00	0.00	5.59	0.60	0.73	2.76
17	0.92	1.56	0.79	0.90	1.90	5.94	1.46	0.84	8.13
18	0.83	0.27	0.42	1.34	0.76	5.52	1.36	2.64	5.82
19	0.38	0.03	0.41	0.36	0.12	5.31	4.33	9.02	8.42
20	0.99	0.39	0.21	0.98	1.57	6.82	7.82	8.44	13.66
21	0.69	1.31	0.09	0.60	0.57	3.20	4.71	12.07	7.41
22	0.86	1.81	2.09	2.19	2.19	11.65	16.96	15.06	3.30
23	0.48	3.20	5.08	0.60	1.27	9.54	12.36	2.42	2.98
24	0.44	3.57	2.98	2.25	6.84	13.08	17.04	11.38	11.76
25	0.86	4.86	0.26	0.51	0.64	12.52	3.26	9.70	6.81
26	1.00	2.06	2.71	0.55	0.82	10.45	12.99	5.25	8.79
27	0.96	1.73	4.90	2.24	1.12	5.51	9.61	5.33	1.11
28	0.89	3.06	4.63	1.15	3.43	10.99	6.01	1.37	4.19
29	0.69	19.17	7.30	0.83	11.86	29.84	11.73	1.12	13.32
30	0.89	5.33	0.04	0.06	3.34	24.31	2.37	0.46	17.03
31	0.90	0.29	0.36	0.20	1.20	11.83	10.02	2.45	21.06
32	0.28	5.24	0.30	2.46	4.22	26.47	4.17	29.39	11.99
33	1.02	13.19	9.66	6.04	7.70	12.44	10.92	2.58	4.01
34	0.99	0.99	1.09	0.05	0.29	11.60	6.28	0.61	7.86
35	1.02	1.06	0.87	0.74	1.72	6.69	4.26	8.29	7.77
36	1.01	0.51	1.34	1.68	1.07	8.36	3.55	7.02	15.59
Field 4									
1	1.00	6.77	7.49	4.82	8.98	6.10	10.35	9.76	3.32
2	0.85	0.32	4.84	2.66	0.04	2.50	4.96	2.56	0.06
3	1.01	4.97	10.84	2.21	2.79	4.89	2.76	0.81	1.08
4	1.02	0.12	0.55	1.20	0.22	0.41	2.57	1.06	1.78
5	1.00	1.69	0.66	0.56	0.22	0.48	0.82	0.40	0.47
6	0.98	1.35	0.71	7.61	6.69	1.07	2.59	9.33	9.48
7	0.98	2.70	5.80	1.06	1.79	4.50	9.56	2.79	0.57
8	1.00	1.32	1.82	2.12	1.17	0.24	0.07	0.22	0.56
9	0.97	0.90	0.83	1.24	0.77	0.08	0.18	0.55	0.33
10	0.51	0.11	0.16	0.04	0.09	1.00	1.33	1.46	0.15
11	0.95	0.06	0.36	0.75	0.01	0.14	0.97	1.94	0.30

12	1.00	1.26	0.63	0.68	3.63	6.08	0.14	0.06	4.54
13	0.82	0.93	2.20	2.13	2.58	2.29	0.87	4.09	3.15
14	0.97	5.23	8.91	9.03	1.58	4.07	7.65	12.53	2.57
15	1.00	0.26	0.06	0.00	0.05	0.30	0.01	0.00	0.04
16	0.99	0.83	1.93	2.39	1.61	0.11	1.11	2.13	0.56
17	0.95	0.46	2.07	3.38	1.64	0.07	0.81	1.49	0.38
18	0.96	0.57	2.51	4.26	1.13	5.03	6.08	10.23	2.95
19	1.01	0.17	1.03	0.97	2.51	0.18	0.01	1.39	2.22
20	0.99	3.43	5.60	2.09	4.29	1.38	0.10	0.30	0.89
21	1.04	2.35	10.17	1.38	2.62	0.28	0.23	0.00	0.02
22	0.94	3.27	2.77	7.83	2.87	0.43	5.20	5.85	0.35
23	1.03	0.94	2.83	0.84	1.04	0.08	0.64	0.72	0.03
24	1.01	0.19	0.19	0.53	1.11	0.72	0.19	0.79	1.33
25	0.79	0.75	0.81	0.08	0.11	2.52	2.93	0.03	0.03
26	0.96	0.39	1.29	0.31	0.04	0.49	1.32	0.11	0.02
27	0.84	0.10	0.08	0.09	0.05	0.32	0.84	1.05	0.15
28	0.98	4.03	0.41	5.40	14.12	5.08	0.04	5.55	15.66
Field 5									
1	0.93	4.22	3.07	0.51	1.63	15.04	7.45	3.06	8.84
2	0.90	0.22	0.05	1.42	0.33	7.84	6.81	2.81	1.82
3	0.89	1.65	1.84	2.16	3.51	1.74	5.30	11.56	10.97
4	0.31	0.32	1.10	2.65	3.31	3.39	15.81	3.28	4.52
5	0.94	2.08	6.71	3.01	1.58	6.41	12.26	4.82	4.21
6	1.00	1.19	0.87	2.70	2.52	3.25	7.84	6.59	5.63
7	0.51	0.65	0.74	1.49	1.08	5.58	3.92	9.00	8.83
8	0.96	4.79	2.49	4.08	4.87	3.73	1.32	4.33	2.64
9	1.02	5.14	8.94	3.75	2.08	3.28	4.00	1.78	0.24
10	1.02	1.41	5.44	1.73	0.35	0.73	5.94	2.60	0.38
11	0.69	2.00	1.09	1.04	2.29	1.99	0.62	1.47	3.65
12	0.99	1.43	1.96	0.62	0.84	0.92	0.58	0.08	0.16
13	0.40	0.02	0.15	1.14	0.67	0.96	1.03	1.26	0.64
14	0.29	0.00	0.00	2.12	2.89	0.05	0.01	3.77	2.08
15	0.97	3.15	5.52	3.79	2.69	5.88	3.54	2.79	3.39
16	0.97	10.77	1.84	0.73	3.70	3.58	1.98	0.56	1.79
17	0.98	4.81	2.29	1.22	2.73	2.51	0.41	0.16	0.65
18	0.99	0.35	1.30	5.48	2.42	0.29	0.67	2.43	1.20
19	0.97	4.40	0.98	0.38	1.20	3.21	1.00	0.48	0.60
20	0.99	0.33	0.80	6.07	4.24	0.06	0.80	3.13	1.62
Actin									
Field 1									
1	1.00	7.09	7.32	8.65	6.47	7.06	9.90	9.16	6.17
2	0.98	7.14	5.90	7.10	5.90	9.02	10.46	10.55	7.00
3	0.98	18.10	16.42	17.57	16.71	24.14	25.88	26.56	28.17

4	0.92	6.55	9.33	8.49	11.69	13.95	12.77	8.51	12.28
5	1.02	3.06	3.70	0.68	0.89	3.65	2.72	0.43	1.03
6	0.98	11.07	31.43	18.52	13.85	7.20	13.90	12.57	8.66
7	1.00	17.13	12.53	11.08	18.16	13.67	10.88	14.57	15.77
8	0.66	4.22	7.29	6.02	2.84	7.41	8.46	7.45	7.50
9	0.76	3.63	5.12	10.33	8.35	8.48	10.61	6.00	8.32
10	0.93	10.57	7.52	11.10	5.21	11.10	10.69	8.36	8.39
11	0.96	10.62	8.27	14.67	23.54	13.43	9.74	8.16	10.27
12	1.02	33.01	32.51	33.58	39.69	23.46	26.05	29.15	25.85
13	0.98	6.26	9.37	14.10	6.92	8.51	14.21	16.85	10.16
14	0.97	22.75	20.10	16.52	30.71	13.81	13.12	9.75	12.35
15	0.89	20.19	9.27	7.45	15.55	15.68	9.73	10.26	14.01
16	0.96	12.27	20.13	12.64	13.48	8.47	13.55	10.58	8.21
17	0.61	3.53	4.71	3.67	4.88	7.24	7.61	10.37	8.00
18	0.91	3.57	5.22	10.28	9.74	8.57	10.60	9.68	10.22
19	1.02	8.08	10.98	5.08	4.85	8.10	10.65	6.79	6.05
20	0.82	6.32	5.52	6.44	4.80	8.73	8.73	9.78	6.26
21	0.97	7.30	5.58	6.82	3.00	7.70	6.71	6.18	5.97
22	0.82	4.89	4.63	3.13	3.55	8.13	8.42	7.09	6.24
23	1.02	5.53	5.65	7.22	7.17	5.93	6.65	7.19	7.05
24	0.98	8.08	6.93	7.26	5.42	8.76	6.92	7.04	5.86
25	0.89	10.35	4.88	6.10	7.22	10.37	4.77	6.50	12.28
26	0.97	8.56	5.67	6.18	6.92	8.64	8.10	6.18	4.80
27	1.00	13.94	12.87	9.05	11.21	7.92	8.68	7.13	7.56
28	0.34	1.08	5.49	3.73	3.10	6.48	8.94	7.92	4.76
29	1.00	4.19	3.36	3.87	6.72	6.72	6.69	5.68	9.11
30	0.31	5.42	2.63	2.33	3.84	8.19	11.50	7.62	8.92
31	0.57	1.30	2.09	2.70	1.78	8.09	9.65	12.79	11.30
32	0.97	4.23	2.23	2.49	4.28	11.29	8.63	11.79	13.30
Field 2									
1	0.99	2.89	1.96	2.11	5.38	16.94	17.52	14.72	9.53
2	0.35	1.08	1.77	1.35	3.04	14.12	12.81	11.77	15.91
3	0.98	6.53	4.01	4.02	6.82	15.76	16.28	8.71	10.30
4	0.71	2.35	2.23	2.67	3.33	7.99	10.72	11.09	15.93
5	1.01	14.59	11.49	9.19	12.05	21.47	17.25	9.95	15.66
6	0.77	2.42	3.88	4.94	1.98	11.26	9.96	18.78	11.98
7	0.78	9.61	7.16	5.61	7.66	13.50	12.94	12.80	17.57
8	0.74	10.97	4.98	3.18	5.66	14.72	14.02	12.03	14.51
9	0.99	16.15	14.67	21.67	12.90	14.55	24.30	15.84	17.74
10	1.01	3.43	2.09	9.31	10.99	11.48	9.94	22.58	17.07
11	1.00	14.91	14.47	8.09	11.99	14.94	15.44	12.52	22.08
12	1.02	2.33	2.55	1.90	1.76	17.61	2.69	3.53	14.25
13	0.74	4.61	9.66	14.86	8.10	15.98	17.81	16.54	20.30

14	0.63	10.28	15.31	6.07	9.75	14.87	22.13	19.75	21.22
15	0.95	23.91	10.66	9.39	11.01	20.31	21.05	24.55	17.65
16	0.91	15.23	16.73	14.55	17.14	18.01	16.04	20.18	16.27
17	0.97	21.56	9.70	11.87	9.50	25.16	9.10	8.23	11.13
18	0.92	13.41	9.37	7.22	23.37	15.95	9.31	8.25	29.47
19	0.52	16.21	9.92	5.74	7.20	11.84	10.57	15.22	12.43
20	0.96	9.94	14.24	13.74	8.47	16.56	21.87	21.21	17.77
21	1.00	13.08	9.48	7.72	9.86	14.35	24.86	19.69	14.73
22	0.98	14.13	15.54	16.55	10.89	14.67	15.68	12.26	15.10
23	0.93	6.35	12.53	11.61	9.67	10.62	16.80	19.93	16.27
24	0.97	16.76	14.64	14.60	20.33	19.24	16.46	20.32	19.09
25	0.72	5.90	8.62	5.82	6.00	14.63	17.54	12.96	23.40
26	0.94	16.71	6.18	7.98	14.39	12.95	15.13	9.60	14.36
27	0.51	9.70	6.94	1.05	6.49	11.84	10.08	8.85	14.56
28	0.84	18.00	18.35	11.87	14.31	18.68	17.62	9.69	13.27
29	0.98	13.02	15.70	17.84	6.15	10.89	14.03	14.92	10.12
30	0.37	0.32	7.47	11.34	16.10	5.66	12.51	11.38	16.67
31	0.15	1.47	2.78	3.19	4.12	9.74	6.40	9.25	12.24
Field 3									
1	0.50	11.30	14.00	7.11	10.72	12.66	22.67	18.33	11.26
2	1.00	14.00	12.04	16.28	15.63	11.93	10.38	14.63	11.70
3	0.88	13.12	11.06	14.91	17.55	8.29	9.56	10.16	12.95
4	0.98	16.97	18.07	27.50	17.39	17.66	11.03	11.29	12.70
5	0.97	26.40	16.38	12.19	12.88	10.80	9.62	19.26	11.27
6	0.91	11.13	12.72	13.30	20.66	12.27	13.70	13.77	17.06
7	0.96	12.82	19.66	11.76	10.16	14.47	22.66	13.30	11.85
8	0.92	17.60	15.55	12.94	14.05	9.49	6.44	8.11	9.88
9	0.96	22.86	17.17	15.48	12.45	11.28	12.53	9.89	7.89
10	0.99	22.27	14.66	18.44	20.91	9.57	9.64	9.99	12.21
11	0.88	18.25	11.95	13.63	11.49	11.41	6.03	7.90	7.87
12	1.01	26.98	22.64	20.02	33.65	13.89	11.38	11.46	15.15
13	0.97	12.99	7.97	8.38	9.49	7.29	7.20	9.31	8.04
14	0.97	8.62	10.36	17.26	10.78	5.94	8.08	8.79	6.55
15	0.96	6.32	6.41	4.74	5.70	11.97	9.25	8.85	8.06
16	0.69	10.10	8.47	10.82	11.55	12.85	12.69	13.84	10.71
17	0.84	8.67	12.61	3.93	8.21	15.40	7.08	9.66	16.19
18	0.78	11.12	13.01	11.35	10.18	9.80	16.07	12.63	9.78
19	0.93	14.24	10.46	10.31	18.18	11.36	10.27	10.20	14.22
20	0.91	9.51	8.78	8.06	13.32	12.73	11.25	14.25	16.73
21	0.91	8.96	5.34	6.47	7.93	8.44	14.04	13.08	8.02
22	0.98	8.17	10.38	8.06	9.62	10.25	12.74	15.37	15.25
23	0.94	12.45	5.41	9.70	13.10	8.45	10.74	16.00	13.71
24	0.99	29.30	27.99	26.13	42.96	38.72	31.74	30.88	43.78

25	0.51	11.79	15.45	12.10	12.06	13.95	8.63	10.02	17.36
26	0.93	8.94	9.73	16.54	17.32	9.57	8.33	16.13	16.80
27	0.97	14.00	18.91	23.18	18.26	15.97	17.22	22.30	16.34
28	0.93	18.48	28.10	25.74	13.01	15.77	23.38	17.75	10.88
29	0.80	14.82	13.76	10.02	6.11	16.40	11.75	6.55	10.91
30	0.94	18.33	6.28	16.02	22.08	12.73	8.08	19.33	20.01
Field 4									
1	1.01	12.74	11.82	14.49	22.97	14.09	26.30	20.42	20.16
2	0.92	16.48	17.70	16.76	15.18	14.49	21.91	14.89	18.29
3	0.95	16.12	14.94	18.23	14.88	12.09	11.57	11.11	12.13
4	0.74	17.18	17.92	18.92	20.63	12.49	10.38	12.41	13.28
5	0.99	14.19	19.67	16.14	12.89	12.40	12.36	11.18	9.61
6	1.02	36.81	17.78	23.48	36.53	16.83	18.21	17.38	16.65
7	1.01	18.29	35.19	23.99	21.72	12.87	20.97	15.69	12.67
8	1.00	23.96	17.82	19.90	28.50	18.87	17.74	14.57	17.75
9	1.00	16.19	15.56	20.65	26.17	16.64	14.31	12.86	16.46
10	0.41	8.41	12.00	13.11	7.95	19.23	16.36	27.52	23.52
11	0.22	9.80	3.47	12.08	9.36	18.38	11.25	13.80	12.46
12	0.70	5.72	5.76	7.83	8.13	12.97	14.17	13.48	18.13
13	0.52	19.29	14.90	15.70	15.12	11.94	19.92	12.94	11.19
14	0.77	25.03	13.82	20.41	16.70	30.58	11.10	24.71	33.11
15	0.39	13.34	10.70	18.18	7.19	13.84	10.07	14.31	14.51
16	0.72	13.20	12.85	7.78	14.64	14.47	8.46	9.50	11.16
17	0.81	8.37	5.92	8.99	12.37	19.27	11.18	11.88	21.88
18	0.77	18.81	16.71	14.99	24.89	12.12	12.99	10.98	13.91
19	0.92	22.28	23.31	18.20	21.76	15.18	10.72	7.40	11.16
20	1.02	19.35	23.23	28.18	20.34	12.87	20.82	30.09	15.23
21	0.97	30.69	23.22	18.91	20.07	20.86	17.92	18.41	19.41
22	0.97	14.22	21.05	18.70	16.82	10.67	18.06	14.08	9.16
23	0.98	13.51	14.09	13.47	16.67	10.61	11.85	12.60	12.31
24	0.97	19.54	15.30	16.83	17.06	12.26	11.79	11.51	17.93
25	0.83	8.73	17.80	21.05	9.66	11.31	12.07	8.71	10.72
Tubulin									
Field 1									
1	0.99	4.58	4.66	4.69	4.64	4.18	4.19	3.53	3.72
2	0.92	3.87	4.07	4.46	4.55	5.80	5.28	5.12	6.50
3	0.64	4.00	3.89	3.34	3.88	4.72	5.94	3.74	5.66
4	0.98	3.45	3.57	4.55	3.57	3.71	4.04	4.44	4.11
5	0.92	4.05	4.85	4.19	4.32	6.06	6.02	7.82	6.92
6	1.00	3.72	4.29	5.77	5.09	5.75	5.83	7.73	9.29
7	0.96	4.18	6.99	3.66	3.51	4.53	8.23	4.65	3.35
8	0.92	3.73	3.43	3.62	3.72	5.38	3.43	4.68	5.83
9	0.80	4.63	4.40	6.95	5.59	4.28	3.63	4.08	3.88

10	0.98	4.43	3.78	4.58	4.15	5.16	4.37	3.49	4.13
11	0.97	4.17	3.82	4.79	3.90	4.40	3.71	4.44	4.69
12	0.98	3.41	4.14	4.92	3.77	3.12	4.02	4.33	3.25
13	0.95	3.44	3.78	3.75	3.97	3.90	5.06	4.76	4.58
14	0.96	3.46	3.30	3.34	3.23	4.09	3.40	3.78	3.95
15	0.82	3.86	4.07	15.14	10.08	5.24	4.56	9.37	6.29
16	0.99	9.17	5.43	4.41	3.82	7.04	6.04	4.82	5.15
17	0.96	3.62	3.75	3.87	3.63	4.67	4.32	4.25	5.21
18	0.90	4.83	3.62	3.46	4.47	6.96	3.76	3.71	5.48
19	0.63	3.73	4.80	4.57	5.11	4.90	4.80	4.20	5.35
20	0.73	3.87	3.33	3.88	3.92	4.94	3.90	4.89	4.96
21	0.97	5.16	3.62	4.41	5.16	6.78	3.51	4.32	6.81
22	0.98	5.17	7.32	5.42	3.99	6.31	9.83	7.60	4.30
23	0.91	3.66	3.56	3.47	3.53	5.12	4.23	4.32	4.33
Field 2									
1	0.72	1.96	1.98	2.58	7.73	4.03	3.48	3.56	4.27
2	1.00	3.14	4.20	2.12	11.67	4.39	4.39	2.46	11.93
3	0.88	2.03	2.53	2.69	3.22	3.99	2.96	3.30	3.14
4	0.99	8.61	2.66	3.18	3.38	5.33	3.80	3.35	5.05
5	0.92	1.93	4.64	2.06	3.43	4.51	4.91	2.36	3.04
6	0.95	1.83	2.84	1.94	2.91	3.08	2.37	3.57	6.19
7	0.95	1.82	5.16	1.78	1.74	3.00	6.74	2.65	1.74
8	1.00	3.16	3.70	3.17	2.95	3.45	2.33	3.65	3.09
9	0.90	2.80	3.05	3.89	2.23	3.15	3.55	5.06	2.31
10	0.36	1.82	1.46	2.74	3.08	2.66	2.31	6.37	4.38
Field 3									
1	0.34	2.15	3.18	17.31	42.89	2.66	2.90	12.42	30.97
2	0.92	7.69	35.98	40.43	2.86	10.17	45.82	40.09	4.11
3	0.98	4.85	3.30	2.81	6.11	17.43	4.80	2.58	5.67
4	0.99	5.15	5.52	3.66	4.09	6.00	2.33	3.41	6.36
5	0.79	5.64	2.77	2.64	2.78	2.28	2.52	1.99	1.74
6	0.93	6.13	8.47	34.86	7.32	5.16	3.21	14.31	14.25
7	0.99	4.72	3.80	3.09	4.35	2.17	3.23	2.56	2.49
8	1.00	3.30	4.38	5.08	2.82	2.99	4.60	7.60	3.02
9	0.88	3.41	4.34	3.48	3.74	8.35	11.24	6.67	6.50
10	0.98	3.86	4.03	4.05	4.63	5.20	4.16	3.65	6.04
11	0.90	2.47	2.55	5.07	3.24	2.53	2.49	3.65	4.54
12	0.97	3.61	3.54	5.87	3.40	4.45	6.52	5.46	3.50
Field 4									
1	0.95	3.06	2.85	4.10	3.70	4.92	4.85	15.19	7.34
2	0.84	3.96	7.44	9.34	3.78	4.28	10.28	10.47	3.77
3	0.98	5.74	4.46	5.41	15.49	5.60	5.75	5.68	4.61
4	0.99	2.47	2.36	2.75	2.32	4.99	2.73	4.14	5.54

5	0.97	3.40	3.16	2.62	4.05	10.84	3.88	2.73	3.52
6	0.97	4.12	3.62	4.89	3.67	4.62	5.02	10.69	4.30
7	0.87	5.11	5.38	2.99	4.58	4.32	3.62	4.74	3.45
8	0.98	5.84	3.71	5.09	3.95	4.99	4.05	5.62	5.78
9	0.97	4.76	5.44	4.18	4.52	3.91	3.06	4.57	3.76
10	0.73	4.57	18.22	4.51	4.46	3.67	11.79	5.30	2.78
11	0.98	4.36	5.71	4.80	3.08	4.41	5.14	4.90	1.95
12	0.94	4.75	7.73	10.41	4.99	3.27	8.04	15.17	3.33
13	0.99	3.90	3.14	4.13	3.84	5.73	4.31	3.37	3.89
14	0.97	3.10	2.61	4.70	16.76	2.23	2.27	4.22	9.60
Field 5									
1	0.92	17.32	1.35	1.23	6.51	55.38	2.40	3.92	55.72
2	0.76	3.84	29.77	6.79	1.47	2.82	25.51	8.60	3.01
3	0.94	6.61	6.54	3.54	1.11	8.01	7.09	20.93	5.12
4	0.89	0.59	5.05	0.33	0.21	2.77	4.08	1.60	0.88
5	0.97	0.96	1.88	0.97	0.82	3.92	4.08	2.67	3.54
6	0.94	4.64	1.26	1.73	2.11	3.81	2.98	4.00	4.63
7	0.98	1.70	2.61	1.51	1.64	2.78	2.89	1.89	2.44
8	1.03	2.58	0.79	1.05	19.83	14.95	2.85	1.74	16.66
9	0.97	3.88	9.20	1.56	1.33	8.55	53.33	19.23	8.32
10	0.80	4.34	8.32	4.33	2.64	4.94	6.88	12.88	4.42
11	0.97	5.90	10.69	0.74	1.84	30.99	25.85	1.28	5.15
12	0.97	0.89	0.86	0.48	0.87	2.79	4.49	1.50	2.06
13	0.97	3.79	2.46	4.85	3.70	4.16	2.51	4.38	3.13
14	0.98	0.79	0.81	1.79	1.07	3.73	2.70	4.09	3.26
15	1.00	1.58	1.93	1.07	1.11	3.30	4.22	3.08	2.94
16	1.01	2.40	0.88	2.23	2.44	3.78	1.39	5.86	5.97
17	0.81	3.13	3.61	5.91	4.87	1.32	2.58	3.30	1.97
18	1.00	1.53	1.85	1.93	6.98	4.72	3.45	1.55	2.02
19	0.83	2.77	1.65	1.11	1.47	6.72	2.40	2.00	2.83
Field 6									
1	0.97	1.12	1.33	4.80	1.51	3.54	3.55	10.33	2.73
2	1.00	2.28	5.17	25.54	4.46	7.44	9.22	27.17	5.18
3	1.02	12.30	5.41	1.64	2.50	19.81	6.92	3.19	3.00
4	0.95	2.45	1.52	1.39	4.80	9.34	2.66	1.83	3.73
5	0.93	7.61	11.49	3.84	7.05	7.05	11.64	5.52	9.13
6	0.89	7.83	4.07	10.22	28.44	12.79	2.78	14.14	48.12
7	0.37	2.56	7.89	5.61	3.27	1.32	5.43	2.23	2.01
8	0.94	3.61	4.33	2.33	2.49	5.03	4.06	2.60	4.07
9	0.83	1.02	0.70	2.34	1.25	3.30	1.50	1.53	3.40
10	0.84	2.03	1.49	0.79	2.09	5.13	3.71	2.37	3.16
11	0.63	2.00	1.84	3.74	5.74	1.98	1.61	2.20	3.96
12	0.96	4.10	1.91	2.56	5.95	2.85	1.79	3.70	3.89

13	1.00	3.41	3.90	3.81	2.90	4.07	4.03	4.13	3.84
14	0.97	3.41	1.73	1.65	2.11	3.57	8.02	12.58	4.29
15	1.01	2.76	2.34	2.56	2.61	4.25	1.14	1.66	2.84
16	1.00	2.87	3.32	4.06	2.43	1.67	2.67	3.86	1.00
17	1.00	1.96	3.38	4.09	1.74	4.63	5.97	6.56	3.29
18	0.92	1.82	1.84	1.80	1.70	2.06	1.69	4.41	3.88
19	1.00	1.65	1.94	1.55	1.93	2.44	2.40	1.79	2.90
20	0.87	1.50	1.44	1.44	1.56	1.28	1.17	2.07	5.36
21	0.93	3.40	2.47	2.16	2.64	9.35	4.03	3.59	4.31
22	0.75	2.57	2.62	4.61	8.89	8.74	8.00	15.28	68.43
Field 7									
1	0.88	3.52	2.33	2.06	3.96	2.71	2.16	1.09	4.98
2	0.98	1.91	2.45	1.64	1.61	1.79	3.25	1.68	0.81
3	0.92	5.10	30.75	4.31	2.65	7.99	34.10	2.58	1.00
4	0.98	2.22	2.23	2.04	1.99	0.86	1.51	1.66	0.61
5	1.00	1.12	2.12	2.05	1.07	1.43	7.65	1.56	0.89
6	0.97	3.35	1.78	6.56	3.32	1.86	0.75	2.67	3.81
7	0.98	1.79	1.22	1.38	3.09	2.62	0.72	1.00	3.77
8	0.89	8.45	0.79	1.16	9.05	13.12	2.50	2.59	6.98
9	0.95	1.11	4.57	1.84	1.16	1.68	6.18	4.15	3.95
10	0.99	3.22	2.70	15.63	7.26	2.37	2.79	12.84	6.29

Table S7: Raw APVs of DIC images corresponding to fluorescent images of organelles in hypertonically treated RAW264.7 cell population

	Octant 1	*Octant 2*	*Octant 3*	*Octant 4*	*Octant 5*	*Octant 6*	*Octant 7*	*Octant 8*
Field 1-Nucleus/Mitochondria								
1	130.96	134.89	139.70	126.56	130.65	137.73	141.49	120.66
2	141.08	131.25	145.79	134.61	142.40	138.03	144.32	132.63
3	150.07	134.93	136.78	141.70	130.49	130.50	128.20	127.01
4	143.35	132.69	133.01	140.83	131.15	129.14	126.90	122.45
5	136.53	133.19	135.44	140.73	134.93	125.12	133.61	131.46
6	138.68	139.05	123.40	133.98	129.92	127.24	118.27	133.56
7	137.74	118.64	102.65	125.84	125.42	119.63	109.25	124.97
8	131.57	118.89	130.07	135.18	127.15	127.19	117.16	121.77
9	139.67	124.64	131.02	141.46	127.72	126.12	125.81	129.38
10	125.46	121.34	120.89	131.56	125.66	125.33	121.39	126.13
11	122.14	114.92	120.55	124.58	118.86	120.63	112.30	118.47
12	138.48	130.55	149.56	148.93	133.84	132.13	125.83	128.26
13	123.01	123.78	124.47	131.02	122.74	124.56	124.68	120.85
14	130.61	128.22	135.22	145.64	130.56	129.71	126.21	133.36
15	119.73	130.05	136.95	123.91	114.23	114.78	124.48	122.49

16	109.89	112.05	119.31	117.62	99.95	105.00	102.89	101.49
17	109.93	124.54	135.74	126.26	110.86	109.64	118.07	114.61
18	114.52	116.67	112.40	114.49	96.76	109.63	116.44	103.02
19	105.47	127.14	129.85	114.92	102.15	109.59	111.55	104.21
20	90.19	86.80	104.42	108.65	93.51	94.29	90.52	97.60
Field 2								
1	129.86	148.93	137.71	130.80	129.03	124.66	124.50	113.36
2	135.19	142.96	147.26	134.61	134.19	126.63	132.37	127.95
3	131.60	148.96	143.73	131.17	113.09	132.05	129.93	114.66
4	142.36	148.38	147.93	135.45	142.20	139.73	135.67	142.80
5	144.69	141.41	141.48	148.55	131.51	142.73	132.93	129.85
6	147.08	140.51	133.38	140.64	137.11	131.81	125.49	133.92
7	149.78	149.46	139.29	141.17	136.00	130.72	141.93	136.54
8	131.19	124.60	121.81	136.57	119.33	118.53	117.86	122.43
9	139.52	124.54	126.48	134.21	128.04	118.07	112.80	121.89
10	135.27	124.56	119.74	125.61	127.05	116.17	117.07	128.64
11	137.95	133.48	124.87	133.11	124.74	117.09	117.99	130.99
12	146.18	144.27	144.22	144.02	139.13	133.74	134.77	137.65
13	141.56	145.94	151.77	139.35	125.01	125.75	125.27	127.34
14	137.43	130.20	131.07	129.69	120.22	123.92	129.76	136.64
15	138.69	135.66	127.73	133.58	134.49	124.64	121.79	122.34
16	138.63	135.81	140.59	139.29	120.35	129.79	132.61	132.27
17	133.97	138.99	144.26	144.64	128.91	128.46	129.22	140.32
18	143.17	127.90	129.33	135.52	128.79	134.70	130.07	130.03
19	135.31	135.57	142.28	145.06	132.06	134.29	137.23	130.57
20	127.42	140.53	141.98	131.10	131.67	117.43	125.75	123.76
21	127.76	142.51	131.74	122.04	108.98	116.26	119.40	118.88
22	130.97	135.48	137.79	130.53	125.32	131.93	134.56	128.80
23	124.89	126.15	129.65	123.29	121.90	126.46	128.97	122.15
24	99.62	103.44	103.97	100.33	91.19	105.70	103.03	98.22
25	113.32	121.48	126.85	118.95	106.86	107.68	115.23	112.24
26	123.11	125.50	125.59	125.01	123.99	120.69	124.22	124.77
27	122.70	118.80	119.00	123.21	124.37	117.89	120.46	121.77
Field 3								
1	155.14	147.40	145.52	156.62	137.14	151.23	145.67	142.18
2	147.93	152.61	153.79	144.85	144.22	144.10	136.43	148.69
3	147.64	145.76	139.83	138.49	142.45	145.53	131.09	131.42
4	132.71	137.34	129.48	133.98	129.62	135.63	120.65	121.59
5	136.88	141.69	138.35	128.98	141.70	139.40	125.04	130.19
6	141.02	138.53	133.58	136.07	139.00	141.64	128.30	136.80
7	133.42	147.23	143.57	133.26	131.95	138.68	136.95	137.42
8	128.11	136.38	144.33	131.33	127.82	138.85	138.44	122.83
9	135.54	137.27	137.56	137.76	132.26	137.59	137.71	135.42

10	134.78	133.12	139.01	137.16	138.57	133.62	134.99	136.78
11	134.26	137.89	143.54	140.04	137.50	141.57	143.19	141.99
12	135.34	143.45	144.09	142.10	138.87	130.01	134.71	132.31
13	133.67	145.92	141.54	148.42	140.72	137.95	139.85	137.37
14	142.22	150.89	146.50	156.70	135.87	125.66	132.05	137.54
15	138.95	137.58	154.30	148.88	131.88	138.44	131.18	134.33
16	142.90	140.65	144.91	147.65	135.53	130.89	136.56	137.47
17	138.17	133.36	135.53	134.10	125.93	139.14	136.29	140.29
18	126.21	130.28	133.64	128.98	125.47	130.31	133.32	136.90
19	117.94	126.61	135.72	127.87	131.53	128.11	135.41	128.32
20	127.56	135.96	131.78	134.11	123.30	122.11	132.35	121.74
21	118.99	136.69	133.23	126.39	127.57	134.44	133.76	127.10
22	118.58	127.04	128.12	114.69	117.28	132.62	134.22	126.65
23	106.16	122.38	123.58	123.01	108.37	120.08	125.61	117.06
24	119.58	130.30	129.71	117.37	122.50	119.67	130.20	119.67
25	115.26	116.16	129.77	116.60	114.84	111.98	119.78	110.92
26	105.57	120.17	119.17	102.57	111.64	111.33	118.87	105.95
27	101.16	108.88	119.32	107.17	96.69	110.20	111.81	100.17
28	125.42	133.09	129.65	125.36	125.82	132.55	132.55	125.75
29	118.90	133.81	134.50	123.61	128.78	134.56	133.47	126.22
30	116.30	129.51	130.60	118.33	122.25	129.60	123.03	119.42
31	116.00	123.99	122.90	117.05	118.26	124.55	125.33	115.77
32	122.07	115.86	120.26	119.70	113.93	124.35	122.04	109.82
33	131.76	131.88	130.39	130.68	133.31	128.81	129.26	134.86
34	129.98	129.09	131.01	138.14	130.11	126.62	125.83	129.39
35	126.96	119.16	129.32	138.17	127.03	124.74	125.77	127.94
36	114.32	105.90	113.14	115.62	111.88	103.86	112.19	118.34
37	102.24	100.12	115.23	119.38	104.04	99.54	103.60	115.25
38	137.94	134.82	134.23	138.42	135.07	130.81	132.92	133.79
39	134.32	136.45	133.76	136.10	138.69	135.58	129.23	138.96
40	137.95	129.35	126.03	142.22	134.55	126.79	121.53	134.41
41	126.06	115.10	112.79	128.59	125.38	113.63	116.14	126.63
42	119.35	127.07	125.07	127.02	129.65	116.29	118.69	127.50
Field 4								
1	144.70	148.33	152.61	141.19	141.11	149.94	145.43	135.61
2	151.90	155.28	149.98	153.97	147.05	145.02	138.77	147.80
3	155.69	151.54	148.96	148.13	149.41	149.63	147.17	138.45
4	155.68	149.95	147.60	149.08	148.52	140.23	135.46	144.10
5	147.50	144.98	157.22	152.21	145.92	139.89	137.84	151.66
6	143.79	148.21	158.59	144.21	152.61	147.53	136.31	155.02
7	135.43	145.80	144.17	143.50	152.91	152.62	143.83	141.73
8	142.06	144.64	145.15	151.56	140.11	142.32	138.54	141.09
9	141.21	149.67	150.36	143.58	125.64	146.00	155.40	132.46

10	147.06	151.24	145.86	144.26	126.53	138.59	150.59	147.03
11	136.70	141.46	148.01	139.78	137.43	137.34	143.77	134.69
12	129.57	141.79	133.54	127.54	116.19	124.38	133.93	125.50
13	125.58	133.90	137.10	130.13	117.04	117.16	127.25	121.89
14	99.19	107.16	113.83	103.60	94.81	106.15	109.56	102.02
15	109.64	112.74	120.02	109.38	103.32	114.18	118.31	96.14
16	123.56	111.81	121.70	138.66	112.38	112.16	114.38	114.75
17	118.81	109.49	113.04	123.90	113.79	103.96	106.56	116.80
18	149.02	144.31	139.27	139.07	150.15	131.96	138.33	145.65
19	127.37	108.14	114.55	129.49	125.76	107.85	119.13	122.25
20	127.21	116.76	120.39	129.08	123.70	107.00	115.44	127.77
Field 1-Cell Membrane/Endoplasmic Reticulum								
1	77.48	76.31	75.35	73.62	81.60	82.96	78.40	81.61
2	68.73	73.50	72.61	68.13	67.88	67.58	71.39	68.43
3	73.06	72.48	75.50	72.50	75.35	78.15	78.19	76.53
4	74.95	74.20	79.03	76.32	80.21	81.62	83.35	80.61
5	71.84	66.85	68.00	74.00	76.93	63.72	61.60	74.49
6	76.09	73.02	74.56	79.09	78.54	78.38	73.62	73.38
7	80.91	73.78	76.43	77.92	72.17	78.31	72.86	80.81
8	81.67	76.09	74.04	72.90	71.39	70.81	69.04	67.78
9	72.17	64.60	61.24	65.77	73.06	62.10	58.08	68.28
Field 2								
1	93.15	100.09	99.61	96.17	94.09	99.47	96.50	93.50
2	90.90	95.70	96.42	95.97	89.07	91.96	99.09	88.46
3	95.47	100.46	100.59	97.20	99.60	99.20	100.63	93.32
4	90.03	99.59	96.43	94.35	88.75	92.74	100.04	90.77
5	94.16	97.98	100.14	96.44	96.81	101.46	100.32	92.19
6	99.68	95.00	98.38	100.56	94.17	93.00	100.75	104.47
7	97.93	94.35	98.96	100.21	102.61	96.70	102.05	109.65
8	96.14	91.89	93.54	95.94	95.16	91.80	101.19	100.76
9	90.53	93.26	95.65	95.56	97.02	95.76	96.95	99.77
10	96.85	95.33	94.36	97.54	102.08	98.45	99.94	103.08
11	99.43	97.66	102.98	104.35	105.44	95.24	98.68	102.50
12	100.72	95.13	99.11	109.94	96.57	91.79	92.75	97.49
13	104.13	94.84	99.73	110.55	105.43	91.59	97.01	101.89
14	110.27	108.67	95.34	103.56	107.58	97.37	101.51	97.23
15	103.84	95.08	100.71	105.13	111.44	97.28	99.98	102.60
16	110.14	104.71	101.87	106.11	101.72	99.32	101.45	106.93
17	104.62	92.43	97.08	107.45	104.35	90.89	81.95	92.67
18	98.07	105.76	98.19	97.51	97.42	97.76	97.17	92.66
19	100.88	104.99	105.46	102.92	104.03	97.96	100.92	115.45
20	101.92	102.17	102.20	104.25	99.01	95.00	102.85	100.31
21	105.42	105.19	103.37	103.65	100.28	108.00	105.18	102.42

22	93.04	106.07	106.73	110.53	96.90	113.72	98.28	96.06
23	98.52	105.34	105.18	102.38	89.46	99.62	111.69	104.35
24	105.69	106.61	108.16	107.46	114.77	109.73	105.24	101.53
25	104.98	99.97	107.30	108.40	104.97	104.60	110.50	109.88
26	104.99	98.90	99.98	108.06	102.63	108.87	105.02	104.12
27	98.53	99.61	98.32	106.26	105.41	104.22	106.18	106.91
28	99.93	104.05	102.98	102.65	106.34	106.05	106.90	107.21
29	97.02	100.13	101.47	102.57	102.32	99.96	100.56	108.65
30	106.03	102.73	104.61	106.06	109.77	104.83	101.21	105.27
31	110.95	109.44	106.84	106.77	107.47	107.29	107.97	105.34
Field 3								
1	116.49	129.15	124.12	119.76	118.21	123.82	117.81	108.67
2	124.94	127.67	120.52	120.39	109.14	118.62	114.31	115.93
3	115.64	123.68	121.72	113.82	113.17	116.09	107.57	106.05
4	122.94	127.95	126.68	113.66	119.92	114.70	110.89	119.27
5	114.54	121.27	118.31	115.19	111.96	108.08	109.14	100.32
6	108.28	114.82	117.53	110.19	108.87	110.43	110.83	108.86
7	106.14	109.62	115.50	106.11	91.60	106.67	109.00	100.49
8	104.46	107.26	117.68	115.94	114.26	112.88	113.91	107.47
9	106.55	114.01	118.49	106.32	107.14	105.74	108.56	98.87
10	103.40	113.52	123.70	117.52	115.00	107.81	105.88	108.67
11	112.48	118.89	125.57	120.67	112.69	119.15	113.58	114.03
12	115.39	122.07	122.38	116.96	111.80	122.06	122.96	110.97
13	100.02	107.97	107.56	100.69	98.93	105.29	96.25	94.97
14	102.66	112.17	115.82	110.33	104.88	109.93	105.91	104.21
15	124.23	117.85	118.93	119.57	105.79	110.93	112.32	115.72
16	124.27	125.17	122.44	126.59	116.98	123.50	122.76	120.68
17	127.01	124.55	121.49	122.10	124.70	129.95	128.02	120.89
18	113.22	117.05	116.57	126.10	121.53	104.65	111.25	115.66
19	119.23	115.07	108.15	108.55	121.35	106.33	110.07	121.91
20	119.46	113.30	103.67	112.33	111.71	110.29	107.26	113.22
21	116.66	115.50	113.37	121.51	115.59	98.91	101.09	115.13
22	117.61	119.46	121.98	123.73	118.29	109.66	114.02	123.24
23	117.65	108.06	112.58	112.75	111.72	103.98	108.80	111.01
24	125.76	119.34	116.88	128.65	119.84	109.29	113.96	116.36
25	118.28	126.89	129.57	124.22	123.15	110.32	110.13	113.54
26	123.83	120.50	116.21	128.62	125.57	120.49	117.48	122.27
27	118.11	124.89	121.13	116.14	128.51	117.74	117.99	127.55
28	123.43	115.42	116.14	124.05	117.94	101.98	106.25	113.82
29	122.33	122.40	117.58	132.26	124.32	107.45	109.34	115.91
30	126.62	132.80	126.35	128.15	125.62	127.72	124.56	120.97
31	125.22	120.13	117.12	127.03	131.23	119.67	117.90	121.51
32	123.07	133.63	125.66	126.99	131.29	121.19	126.47	126.77

33	137.30	135.16	120.62	126.20	127.20	125.42	127.21	126.64
34	128.07	119.40	130.75	130.02	132.04	131.72	129.47	129.20
35	129.18	129.36	120.79	124.07	123.39	128.39	120.88	122.26
36	127.72	131.68	122.98	121.96	129.61	128.92	121.30	116.54
Field 4								
1	135.06	138.08	125.42	134.97	124.55	123.00	118.08	125.20
2	133.28	131.74	126.33	130.92	126.45	126.74	121.23	122.30
3	122.95	129.06	121.03	113.92	117.29	122.40	114.77	112.05
4	129.57	129.29	129.21	128.75	122.12	124.45	117.20	122.23
5	125.63	125.59	125.54	125.71	115.12	118.31	125.19	120.88
6	120.31	131.38	124.44	113.76	107.53	112.41	114.67	116.75
7	129.62	136.53	128.09	130.04	122.52	125.66	128.90	120.70
8	123.44	130.96	131.16	128.95	121.07	121.13	119.75	121.80
9	116.54	123.63	124.19	123.42	113.43	114.94	119.81	114.90
10	117.69	120.29	125.35	122.55	117.26	115.95	113.92	116.49
11	124.85	123.40	128.93	123.19	115.05	121.75	118.82	121.78
12	122.92	124.72	126.04	126.00	124.31	121.31	120.59	122.10
13	122.12	123.47	128.53	122.42	115.28	122.36	121.11	124.00
14	120.00	121.98	121.11	126.95	120.66	119.07	122.66	126.19
15	126.79	130.92	126.37	133.76	126.10	125.89	125.94	126.59
16	126.33	124.90	123.02	128.67	126.12	124.21	120.14	126.02
17	128.91	137.91	127.46	135.94	128.80	134.94	128.56	128.68
18	129.27	134.33	133.62	130.11	129.38	133.72	132.44	130.34
19	120.04	114.52	108.21	123.64	117.88	110.40	106.97	117.60
Field 5								
1	146.21	142.17	132.91	135.55	129.93	127.84	122.73	126.99
2	141.39	148.38	148.05	148.88	136.98	139.58	133.44	136.79
3	142.28	144.04	141.76	143.83	131.64	138.38	136.45	136.22
4	127.55	143.40	136.48	136.59	125.01	135.38	124.60	129.08
5	125.33	138.45	143.41	135.11	123.61	131.73	129.81	131.37
6	129.87	132.44	130.91	127.19	124.92	122.44	126.79	125.68
7	121.49	116.75	126.71	128.80	113.25	115.79	123.22	122.69
8	142.02	137.83	140.66	152.80	130.47	135.12	130.96	136.16
9	147.63	144.90	142.55	144.87	135.45	139.60	138.44	139.31
10	138.45	136.71	124.91	138.94	135.50	128.45	120.50	125.97
11	132.70	122.16	116.47	131.12	121.83	115.38	113.07	124.90
Field 1-Lysosome								
1	58.89	61.58	60.87	60.19	56.90	58.33	59.80	57.80
2	61.06	62.63	61.02	59.74	60.90	60.94	57.75	60.65
3	58.48	57.96	59.36	56.76	54.93	57.64	57.39	56.22
4	59.07	62.92	59.73	59.06	54.18	57.04	60.62	59.12
5	56.85	57.98	59.02	59.33	55.53	55.80	50.03	53.91
6	57.02	56.31	56.59	53.94	54.29	57.11	55.20	55.57

7	54.34	54.72	54.57	54.27	50.11	53.46	54.39	48.66
8	54.30	57.71	58.11	55.18	54.37	56.44	57.27	53.78
9	58.16	58.41	58.85	57.42	54.75	55.49	55.61	55.76
10	55.73	57.15	56.69	55.15	56.67	56.13	57.13	54.04
11	58.11	55.22	56.55	58.14	56.20	57.86	56.11	55.49
12	54.81	58.19	56.08	58.70	53.02	53.96	57.31	50.85
13	57.72	61.64	59.69	61.51	57.90	57.96	59.43	59.18
14	59.24	60.55	61.71	61.29	58.01	58.00	60.42	57.85
15	60.45	60.54	60.80	61.00	56.37	57.19	59.84	56.67
16	62.98	60.25	61.93	61.42	58.33	59.98	60.85	59.17
17	59.73	62.61	62.24	62.77	61.17	61.91	58.73	58.98
18	61.53	60.25	59.99	63.53	56.68	55.39	54.67	56.08
19	56.68	59.59	58.43	60.68	53.32	54.57	57.47	57.99
20	62.97	61.42	60.98	62.77	63.51	63.08	57.42	55.01
21	62.60	56.02	61.17	61.59	60.11	61.69	59.93	61.27
22	60.85	62.10	61.54	64.46	60.74	61.44	57.96	61.24
23	61.10	62.83	59.29	60.03	58.48	59.96	59.77	59.03
24	58.40	62.34	63.19	60.45	60.74	58.92	61.00	60.32
25	62.44	64.33	62.36	63.39	62.70	62.14	59.47	60.48
26	63.13	58.95	54.99	57.72	50.84	55.41	53.68	52.24
Field 2								
1	65.38	66.38	64.72	62.43	61.19	57.52	56.67	58.26
2	66.73	68.97	65.43	63.64	62.59	59.51	56.44	59.68
3	70.88	64.99	64.06	61.82	60.60	62.03	57.82	56.88
4	65.16	68.00	66.05	65.83	62.10	60.90	57.62	56.54
5	61.08	64.94	61.57	64.61	61.69	63.05	60.09	59.93
6	64.30	62.75	61.16	63.25	60.82	62.71	60.77	60.23
7	64.58	60.84	62.58	65.06	57.89	60.25	61.42	57.57
8	63.64	64.76	62.14	65.20	63.11	63.12	61.29	62.46
9	58.54	62.71	61.80	57.59	55.63	54.54	56.43	51.09
10	60.30	61.70	62.27	57.78	57.59	58.98	59.55	56.95
11	57.73	58.73	58.72	55.64	49.94	53.10	51.82	50.09
12	56.52	59.80	58.07	58.07	52.30	56.39	53.60	54.04
13	60.37	61.95	64.07	63.27	60.68	59.05	56.60	58.72
14	62.70	61.15	61.61	61.57	57.61	59.64	60.99	59.86
15	60.16	60.27	57.66	59.33	50.88	59.81	54.10	54.69
16	57.21	58.95	60.20	59.44	55.05	55.48	55.77	55.58
17	53.69	57.15	59.25	58.17	57.55	54.15	56.30	57.25
18	57.34	59.63	60.91	61.16	53.68	55.58	56.24	55.40
19	60.76	61.52	61.51	62.50	60.70	61.09	58.74	60.01
20	57.74	58.89	60.81	57.92	58.64	58.94	58.40	59.90
21	60.56	61.41	61.10	61.50	57.25	57.78	58.44	58.47
22	57.50	58.49	56.67	56.61	56.21	56.15	54.47	56.84

Field 3								
1	58.16	61.13	59.47	58.24	55.14	59.69	60.62	57.64
2	57.24	57.78	56.55	53.41	50.13	51.83	53.28	50.87
3	55.63	60.71	59.21	56.05	51.62	54.78	53.07	50.98
4	56.81	60.75	62.12	57.63	53.70	59.31	58.22	54.40
5	62.91	60.40	65.79	60.11	61.17	60.67	60.40	59.86
6	59.95	62.96	61.85	57.85	58.83	59.01	60.44	60.14
7	63.10	66.89	63.47	66.05	61.82	58.15	63.67	59.99
8	63.07	62.35	64.20	60.62	59.65	56.49	60.85	62.35
9	61.16	60.58	63.44	60.19	59.30	60.17	59.70	61.47
10	55.52	57.48	59.95	56.24	54.30	55.29	55.17	52.48
11	52.92	55.89	58.97	53.89	54.37	55.79	58.02	56.22
12	55.75	58.17	56.22	56.14	54.46	51.60	55.75	53.92
13	55.60	57.19	62.44	54.27	56.42	57.48	55.18	53.03
14	49.93	53.26	53.40	50.10	48.75	53.44	52.91	48.35
15	56.40	54.67	57.78	61.64	54.15	54.01	57.09	57.93
16	59.53	58.30	58.44	59.80	58.55	57.83	57.38	59.35
17	57.62	59.55	56.21	60.85	56.55	55.35	57.08	58.55
18	60.20	62.62	57.89	56.60	57.77	51.64	53.62	55.25
19	62.98	64.84	63.24	59.64	60.38	56.78	60.87	62.27
20	59.79	60.88	65.24	65.92	60.39	60.81	60.35	59.02
21	59.17	58.37	60.88	63.24	57.29	58.37	58.02	59.36
22	59.06	58.92	61.10	58.65	57.20	58.04	59.11	57.37
23	61.12	58.56	61.34	61.62	58.35	56.86	58.88	59.66
24	63.35	58.55	61.62	63.97	62.03	61.38	60.65	58.83
25	62.81	63.10	63.07	59.92	59.72	62.48	62.16	59.24
26	61.56	62.12	60.90	59.70	57.61	60.47	59.54	58.00
27	63.29	66.95	63.70	60.89	61.18	60.82	59.94	59.44
28	58.18	60.55	61.82	59.24	56.26	60.17	60.73	52.70
29	61.78	61.93	62.41	59.60	59.22	63.87	60.35	59.24
30	65.82	61.80	62.70	64.04	60.21	61.29	63.48	59.30
31	61.27	58.52	61.08	62.06	59.79	60.04	59.45	59.70
32	62.15	62.87	61.27	63.44	62.78	62.17	59.23	60.88
33	61.02	58.76	58.23	60.30	58.87	56.72	53.22	53.10
34	64.88	62.31	60.94	65.53	58.00	58.27	60.56	57.10
35	63.85	67.70	58.66	64.65	58.51	57.88	57.97	58.18
36	61.53	64.77	64.45	69.07	61.78	60.38	58.11	57.42
Field 4								
1	62.39	64.61	63.24	61.47	57.32	61.12	60.27	57.92
2	61.94	63.11	61.81	60.13	60.54	61.97	59.94	62.22
3	62.92	64.08	64.35	60.84	62.53	57.86	61.73	59.77
4	66.71	63.81	68.79	62.88	63.16	65.80	59.47	57.24
5	65.12	70.02	68.88	62.00	61.99	55.85	56.74	58.94

6	61.20	64.28	60.13	60.16	63.26	59.57	56.66	60.86
7	64.78	62.84	61.85	60.66	60.83	62.23	57.26	60.74
8	62.90	60.85	62.92	60.82	59.02	57.29	53.65	56.96
9	63.66	62.41	61.07	64.90	59.84	55.57	60.49	53.06
10	62.26	66.15	63.95	61.70	60.47	61.00	58.25	61.33
11	64.33	64.22	62.82	59.30	61.94	61.26	59.19	57.89
12	65.60	62.03	60.82	63.78	59.61	59.26	57.09	58.33
13	65.62	61.55	60.73	64.17	59.77	55.44	51.65	54.65
14	60.53	62.61	64.09	62.29	57.82	60.03	61.13	59.99
15	59.70	61.46	61.84	59.71	55.88	59.66	57.01	56.47
16	59.04	59.06	60.09	57.42	55.44	58.51	58.56	51.01
17	60.67	61.55	61.67	60.10	56.73	55.51	53.31	53.30
18	60.09	60.68	61.76	55.14	53.22	57.93	52.88	59.45
19	56.14	63.67	60.81	57.02	57.93	57.83	53.02	54.07
20	56.87	57.13	59.66	54.49	53.34	52.50	53.46	54.35
21	53.60	57.10	57.82	55.96	46.33	54.65	55.26	50.11
22	52.41	56.28	54.14	53.17	48.00	50.78	52.27	51.71
23	51.46	56.65	57.41	52.08	45.45	50.51	54.24	47.74
24	49.95	55.08	54.62	53.41	47.43	48.77	51.62	49.98
25	54.63	58.26	60.50	53.91	54.93	56.64	57.17	56.46
26	61.64	62.88	65.87	62.38	53.44	58.20	54.52	55.67
27	61.45	61.79	61.97	65.03	63.75	62.04	59.74	60.68
28	59.65	58.26	58.85	61.44	56.76	56.06	55.92	54.74
Field 5								
1	63.43	62.42	59.43	59.07	57.82	57.04	58.61	55.11
2	61.48	60.75	58.72	58.20	58.36	56.88	53.31	53.36
3	67.29	65.73	59.49	63.52	60.15	60.22	58.32	55.22
4	65.55	66.56	62.19	64.16	62.09	60.38	60.94	58.94
5	64.10	69.61	67.40	62.08	63.15	61.23	58.01	63.20
6	63.90	66.85	63.00	58.95	63.28	64.65	62.21	60.92
7	58.33	63.63	61.90	59.73	58.98	59.49	61.14	56.65
8	57.30	60.11	61.59	59.79	55.55	51.95	57.05	55.26
9	58.55	59.34	59.69	58.27	56.91	56.96	53.48	54.35
10	58.89	67.39	63.74	64.50	57.36	55.56	55.49	56.02
11	57.16	59.48	61.13	56.36	56.34	55.89	56.95	54.37
12	53.02	56.67	57.69	55.35	46.70	55.18	55.72	51.01
13	63.35	58.40	62.23	59.54	62.62	59.44	59.29	58.25
14	64.23	63.23	63.28	63.94	64.24	61.91	56.69	56.26
15	65.28	63.23	61.88	64.19	60.47	60.12	57.91	61.34
16	62.81	64.27	63.17	63.06	59.57	63.30	59.75	63.07
17	64.71	59.54	63.93	66.58	61.23	66.05	62.20	61.58
18	62.11	66.46	65.40	63.28	59.36	61.71	60.68	57.59
19	65.91	63.59	65.72	63.68	60.40	64.01	59.06	58.64

20	63.83	63.57	63.94	63.96	60.17	56.50	59.81	57.15
Field 1-Actin								
1	147.97	154.22	148.48	147.13	148.45	148.12	145.39	141.60
2	153.00	155.78	151.12	147.45	147.70	154.70	152.56	145.31
3	154.07	157.16	156.08	151.42	141.21	147.54	143.40	140.14
4	146.26	148.42	149.06	145.78	144.35	145.02	146.48	135.75
5	134.65	143.12	145.30	145.15	129.54	148.52	142.86	126.71
6	133.56	138.64	138.38	137.51	131.60	124.84	135.60	125.35
7	137.82	144.00	146.08	137.81	139.26	145.30	139.89	137.10
8	158.56	155.71	158.88	159.04	154.14	160.22	160.35	159.21
9	160.68	163.64	161.62	163.71	156.92	160.52	160.43	155.74
10	162.50	165.20	164.61	158.14	162.19	159.64	155.80	157.89
11	162.82	160.25	157.97	152.30	147.29	157.47	151.96	156.43
12	150.45	151.56	154.84	148.68	147.41	151.72	151.49	145.56
13	156.09	157.85	152.75	153.49	158.46	155.64	157.71	156.33
14	146.03	152.85	146.82	152.83	156.28	154.83	162.53	149.72
15	151.63	156.40	156.77	157.35	161.20	154.57	159.41	154.04
16	155.31	153.15	153.33	156.04	151.31	146.16	149.95	149.84
17	161.36	160.56	162.06	162.84	157.87	158.42	159.92	154.48
18	157.45	157.66	160.53	156.06	157.81	156.22	152.99	156.31
19	159.59	156.50	159.38	161.99	145.10	153.96	155.76	151.02
20	152.13	155.48	156.81	155.04	161.72	156.09	151.80	152.40
21	153.74	155.32	159.43	155.42	155.55	154.39	149.29	154.07
22	152.54	159.75	157.34	152.77	156.90	150.64	153.80	156.65
23	156.74	153.53	151.80	153.69	150.56	150.56	153.06	154.01
24	151.58	150.22	153.02	155.44	152.32	152.24	150.17	151.32
25	149.21	147.37	150.21	145.68	142.92	148.13	142.54	144.01
26	144.11	152.07	147.24	154.27	146.61	138.89	141.61	131.06
27	149.67	145.33	145.72	148.34	145.37	141.38	145.65	148.55
28	158.74	154.22	155.12	154.53	157.55	155.29	152.45	157.82
29	156.24	150.08	148.21	154.41	152.68	151.85	149.90	150.48
30	157.62	161.18	163.93	160.76	156.57	159.25	160.28	151.66
31	155.94	158.27	155.40	158.34	160.17	153.83	156.14	158.30
32	153.42	153.63	154.16	156.98	154.93	153.70	152.00	156.55
Field 2								
1	154.64	150.21	148.98	156.25	154.54	149.38	150.36	148.65
2	160.59	152.33	155.10	158.59	158.59	153.64	153.48	152.37
3	159.23	153.38	152.30	155.76	157.37	153.02	152.43	156.48
4	164.94	155.43	148.14	158.89	160.52	154.95	153.02	154.86
5	152.38	147.99	147.38	151.09	150.06	148.21	145.40	149.93
6	164.52	165.28	157.35	164.35	166.36	161.72	157.34	165.01
7	168.71	168.74	160.26	166.65	169.01	162.87	163.05	164.53
8	164.58	163.29	160.23	165.54	162.42	162.46	162.83	161.59

9	163.91	160.95	163.13	163.74	162.45	163.56	160.92	162.50
10	161.76	159.71	158.85	165.79	164.69	161.93	160.97	159.57
11	155.20	164.56	160.92	159.50	157.58	162.45	161.43	159.63
12	160.39	162.79	166.78	152.76	163.50	152.06	150.16	158.43
13	155.89	158.82	162.65	155.76	158.40	160.37	163.46	158.14
14	158.63	162.03	163.76	163.76	160.06	162.59	164.17	161.52
15	160.71	160.62	161.58	159.27	157.45	163.23	161.23	156.91
16	155.57	158.95	156.86	160.46	155.34	160.44	157.94	153.18
17	156.17	153.71	157.22	153.47	153.18	158.29	154.77	154.43
18	156.92	157.77	161.12	153.15	154.68	158.95	158.25	156.41
19	146.33	151.01	150.62	148.02	146.42	149.87	148.33	145.67
20	165.25	165.12	163.65	165.34	166.59	165.32	162.85	165.69
21	165.81	164.13	159.91	161.31	165.77	165.15	162.11	162.57
22	152.37	156.05	161.00	152.57	150.06	153.73	151.87	147.66
23	151.27	156.53	161.25	152.67	153.02	160.44	158.32	152.27
24	161.58	156.53	161.05	158.94	158.96	154.86	155.12	156.10
25	159.17	158.19	156.90	158.19	159.13	157.24	157.75	157.67
26	153.46	153.78	151.32	152.20	151.24	154.53	151.78	153.99
27	152.00	146.76	151.10	149.69	148.95	152.96	151.30	151.17
28	144.66	150.29	153.10	146.36	144.40	143.55	148.69	146.82
29	142.38	145.33	145.45	145.17	142.90	143.87	148.59	144.80
30	151.91	153.79	151.89	147.21	152.96	147.80	149.24	144.74
31	162.83	160.52	157.67	157.05	162.49	161.26	157.51	160.75
Field 3								
1	153.73	157.89	158.02	156.40	145.51	158.55	155.98	147.83
2	156.97	158.68	162.00	155.56	155.54	163.23	156.81	151.82
3	155.34	161.09	163.21	157.88	155.70	159.30	160.21	151.44
4	159.17	158.14	159.40	154.50	150.89	157.22	153.79	149.46
5	161.32	159.34	161.24	157.53	151.30	155.01	154.69	150.90
6	164.30	164.20	162.67	159.72	158.94	166.07	155.69	156.28
7	161.14	160.14	158.95	159.36	162.53	144.51	159.72	158.25
8	148.85	148.83	148.61	144.71	141.30	150.92	148.47	141.69
9	144.52	145.05	144.50	140.92	139.02	143.74	143.05	142.90
10	152.87	162.85	156.36	154.33	145.70	156.30	148.12	146.42
11	152.90	159.67	161.75	154.84	151.02	160.69	152.11	153.68
12	146.81	153.32	152.58	147.58	145.87	152.35	151.96	135.99
13	157.32	159.01	159.22	159.77	160.01	155.27	154.25	159.98
14	155.35	158.63	159.12	165.09	164.01	158.86	155.44	158.88
15	167.71	164.36	164.89	167.72	168.16	169.57	163.24	167.37
16	168.03	168.28	165.63	162.52	167.55	169.08	160.66	165.89
17	168.04	170.95	166.38	168.53	167.67	165.38	170.56	168.85
18	166.50	169.94	167.06	167.87	171.55	167.59	164.63	164.57
19	173.34	167.86	164.26	170.57	160.65	169.19	165.03	158.14

20	172.10	167.85	171.85	168.41	167.71	172.97	161.68	164.70
21	173.00	165.63	168.05	166.37	166.37	168.22	167.61	164.36
22	168.24	163.41	162.68	165.89	162.12	165.71	164.10	163.04
23	163.85	167.46	163.70	166.70	159.64	167.36	158.10	159.51
24	162.88	160.63	156.30	162.43	156.49	156.94	157.43	158.71
25	163.94	156.70	157.08	162.34	164.68	158.22	155.14	159.59
26	165.85	157.70	154.24	160.03	157.48	157.17	153.05	158.97
27	163.84	161.10	155.64	158.73	168.03	160.74	157.78	162.03
28	156.29	152.68	145.88	154.31	157.87	142.87	149.70	150.93
29	153.98	145.71	150.42	160.78	145.38	149.69	147.97	155.53
30	156.27	153.90	153.27	156.58	162.84	159.43	154.19	160.48
Field 4								
1	172.57	168.29	167.87	169.48	166.18	171.52	167.10	166.42
2	169.51	167.68	166.31	166.88	172.02	168.29	166.55	165.48
3	165.23	167.52	163.75	163.65	160.82	163.15	162.79	154.94
4	167.33	171.53	171.25	166.28	165.74	164.96	171.79	164.76
5	166.13	166.35	169.60	163.61	168.17	167.81	162.49	163.42
6	160.12	165.97	165.14	162.62	161.17	164.88	164.28	160.81
7	157.55	162.40	167.03	161.86	157.79	161.73	161.44	159.81
8	162.37	167.45	165.23	161.42	160.52	168.83	166.59	165.20
9	170.53	164.70	165.91	168.32	171.78	167.55	164.12	167.91
10	159.89	157.73	157.94	161.13	160.68	157.36	157.42	161.72
11	170.08	173.26	168.48	170.55	174.27	171.15	166.85	169.69
12	173.85	171.69	174.12	174.77	174.79	174.36	168.19	170.15
13	172.85	173.80	174.70	174.53	171.55	173.80	170.74	170.24
14	172.83	173.49	172.56	172.87	167.22	171.29	169.14	172.30
15	162.76	166.12	168.40	168.83	163.46	162.01	164.43	168.20
16	166.93	168.26	169.26	164.41	162.36	168.44	168.15	163.98
17	156.34	164.67	162.11	158.55	158.46	161.57	161.88	157.53
18	146.14	152.99	153.39	148.09	146.90	153.26	153.24	146.67
19	144.48	146.73	148.23	145.37	137.81	144.30	147.30	142.62
20	158.65	161.29	158.88	159.13	160.43	155.98	157.44	160.61
21	156.58	155.00	155.47	156.96	153.14	153.62	154.12	156.80
22	156.96	156.00	161.28	164.16	156.70	156.58	155.76	157.26
23	160.22	159.41	162.72	160.18	161.55	161.95	159.30	156.91
24	161.45	158.54	163.06	162.82	159.81	156.23	156.81	161.29
25	165.25	161.56	165.31	163.99	165.71	158.69	160.22	164.62
Field 1-Tubulin								
1	102.23	101.40	102.35	108.50	99.58	105.01	93.84	95.04
2	107.03	109.41	109.23	108.65	108.76	104.59	99.62	103.39
3	117.65	96.86	104.88	99.12	100.89	97.21	107.92	97.79
4	109.42	110.10	109.46	116.69	106.43	107.72	103.25	102.09
5	101.85	112.39	110.55	101.97	94.48	97.01	99.71	100.63

6	110.89	106.76	110.57	111.15	110.06	110.68	104.67	103.75
7	103.62	106.08	110.26	115.33	98.89	103.89	105.17	100.68
8	106.10	114.96	108.69	103.12	98.39	99.92	106.87	104.35
9	95.08	100.98	107.31	99.58	91.50	99.71	100.28	89.56
10	89.68	91.79	96.64	88.32	81.43	87.64	82.53	77.17
11	87.67	86.54	103.03	95.46	84.22	89.68	72.22	79.31
12	86.33	88.73	93.41	89.34	74.56	87.00	80.20	78.94
13	89.27	98.48	100.18	98.77	87.71	89.17	94.97	96.28
14	113.36	107.76	112.66	107.88	107.56	109.34	111.02	115.17
15	102.63	105.49	101.81	112.54	103.51	104.49	108.36	104.28
16	102.07	98.73	101.60	107.52	98.10	96.11	97.86	101.44
17	105.31	98.19	101.90	102.13	94.67	87.83	95.97	99.46
18	95.34	89.25	95.65	109.12	94.07	89.98	93.48	96.19
19	111.98	101.37	109.55	114.92	105.19	102.02	110.68	102.60
20	112.88	90.25	112.53	101.57	103.24	101.47	104.08	107.43
21	107.62	93.45	108.89	103.47	96.12	91.43	90.76	97.40
22	107.95	95.81	97.65	106.86	96.97	99.15	102.48	100.26
23	112.05	93.37	102.78	105.28	105.22	92.96	97.29	96.72
Field 2								
1	121.26	114.50	114.60	114.91	120.24	112.29	109.13	111.80
2	123.47	117.03	117.59	122.23	124.82	111.06	116.90	120.91
3	128.94	127.85	120.80	117.06	131.74	121.20	116.87	113.81
4	115.49	117.79	123.36	117.91	116.51	120.92	118.32	118.21
5	104.94	121.40	134.59	121.74	107.35	117.20	117.49	111.39
6	112.33	123.14	122.05	110.69	111.06	110.29	119.79	115.92
7	108.87	99.80	122.88	103.42	102.73	94.76	107.03	105.39
8	116.68	114.17	122.75	122.70	118.31	115.32	115.48	118.94
9	111.03	104.56	115.42	118.32	111.77	105.59	104.96	119.75
10	123.87	126.62	125.11	129.49	128.73	128.59	125.79	129.88
Field 3								
1	129.27	137.34	130.34	117.61	120.80	122.16	130.07	125.11
2	120.06	123.35	125.93	121.37	118.37	125.35	125.41	120.03
3	116.13	121.16	124.80	121.10	112.24	123.10	126.19	114.65
4	118.42	129.29	125.03	119.83	110.81	116.45	122.42	112.64
5	116.03	124.60	124.73	120.61	112.51	124.97	124.98	115.93
6	105.06	118.09	113.94	106.18	101.50	112.09	107.86	102.02
7	112.31	122.22	123.22	110.36	110.32	110.01	119.81	114.64
8	130.53	129.06	129.44	133.87	129.25	123.32	127.55	132.15
9	131.67	129.30	128.72	132.24	133.49	128.41	129.00	133.20
10	116.66	117.04	114.42	115.83	115.40	106.55	109.49	109.11
11	125.99	128.77	127.64	119.28	128.51	128.16	128.13	114.38
12	130.30	126.92	124.26	126.15	128.79	127.50	121.18	127.69
Field 4								

1	136.25	143.46	136.40	135.18	134.31	137.10	134.29	128.98
2	137.26	134.10	122.70	132.98	132.99	130.30	119.27	129.97
3	138.65	137.07	136.86	141.75	133.06	135.09	131.95	130.61
4	133.62	125.74	124.09	128.98	129.05	124.63	119.18	125.44
5	137.00	137.59	134.18	136.34	134.89	130.97	132.98	131.10
6	134.92	137.62	134.79	137.81	129.65	135.03	135.58	132.18
7	132.38	135.38	132.40	131.84	127.74	133.20	132.66	126.22
8	135.96	135.48	137.08	141.01	134.42	133.02	134.97	132.73
9	137.76	132.06	133.19	138.54	132.80	127.15	130.22	130.19
10	107.00	114.79	114.52	111.03	105.45	113.07	112.83	106.40
11	116.75	122.59	119.18	118.47	114.37	119.40	114.09	114.04
12	130.40	131.49	130.92	132.22	129.33	128.90	129.82	127.46
13	120.58	122.95	123.44	121.08	117.03	116.92	118.10	119.25
14	118.19	108.35	112.48	120.16	113.28	106.73	107.16	115.20
Field 5								
1	134.29	135.59	131.08	128.18	132.20	131.48	123.45	130.27
2	135.17	130.68	124.17	134.44	136.37	131.85	125.81	132.15
3	131.95	132.16	121.90	131.77	133.10	121.46	125.77	127.12
4	133.13	147.44	130.00	135.24	140.17	131.16	132.04	137.34
5	135.64	138.79	139.90	134.01	137.12	129.11	128.30	134.03
6	136.45	131.39	121.11	141.96	134.23	128.46	120.42	127.90
7	136.48	120.14	123.27	125.35	130.75	119.83	119.09	123.88
8	144.64	137.41	141.98	145.56	139.71	135.81	138.76	140.44
9	143.24	137.61	134.65	141.69	137.07	139.06	132.19	134.57
10	133.69	137.05	140.80	132.74	131.61	138.14	139.71	126.05
11	119.85	124.93	127.72	125.02	115.03	127.69	127.95	117.75
12	119.11	125.64	123.26	117.02	113.98	123.55	122.04	112.91
13	106.45	111.40	117.75	109.11	93.19	111.63	108.69	106.20
14	129.42	136.51	131.60	132.81	126.67	128.41	131.89	131.23
15	131.03	129.55	133.10	137.52	127.89	124.52	134.15	132.85
16	134.70	128.91	134.06	136.57	130.31	125.56	119.12	134.28
17	130.87	126.77	125.16	136.98	119.41	111.59	122.09	128.37
18	119.88	117.18	125.34	122.65	117.98	116.54	114.14	110.94
19	119.02	127.94	136.40	126.55	115.11	120.27	128.18	116.23
Field 6								
1	115.08	123.42	129.00	123.73	117.00	119.69	117.90	115.11
2	131.20	129.96	134.62	133.76	128.64	128.94	128.76	131.88
3	132.43	132.82	133.28	137.55	127.75	127.49	130.46	132.60
4	128.42	131.10	133.56	135.20	132.19	128.09	132.16	124.17
5	139.87	147.88	145.45	142.43	132.10	136.34	133.28	137.52
6	141.77	136.22	131.33	131.13	135.75	133.16	133.74	135.92
7	130.39	116.67	128.85	128.71	128.00	116.88	123.58	125.87
8	135.57	123.25	120.69	126.54	122.74	112.93	116.21	129.46

9	119.17	133.32	125.24	134.35	112.86	123.84	126.63	113.43
10	123.81	122.10	125.95	109.81	113.57	122.72	124.98	112.04
11	125.20	124.72	125.40	125.77	118.65	130.59	119.27	124.51
12	125.35	125.35	138.72	130.26	127.71	123.22	128.44	121.58
13	141.15	150.44	132.43	136.58	134.33	134.98	134.47	134.69
14	150.96	144.26	145.07	143.01	135.23	134.85	141.87	138.06
15	138.37	135.15	137.12	139.04	136.36	137.62	139.22	135.42
16	128.45	122.93	141.42	139.35	125.26	119.17	133.91	134.80
17	152.38	147.74	139.17	140.91	135.93	135.68	131.54	134.60
18	143.21	132.26	134.13	136.83	138.44	135.96	127.32	132.37
19	144.25	134.79	127.51	134.67	140.38	130.41	126.38	134.12
20	144.11	135.85	126.67	136.01	138.90	134.20	124.51	129.10
21	127.61	127.18	127.01	135.27	129.72	125.02	123.64	126.36
22	138.55	136.38	113.94	131.35	132.07	127.46	127.72	126.60
Field 7								
1	142.51	140.79	145.13	139.83	139.94	141.32	138.84	134.42
2	141.52	144.06	138.37	137.13	140.86	135.66	130.65	131.31
3	140.97	139.09	143.94	140.04	141.81	140.40	132.58	131.83
4	127.87	140.62	150.43	133.62	128.91	128.01	129.39	121.85
5	132.25	138.04	142.04	132.11	128.69	131.38	127.50	125.59
6	141.35	129.66	125.78	134.93	130.68	123.24	118.42	121.35
7	134.08	122.05	132.10	134.74	132.80	116.05	121.20	129.97
8	145.18	142.15	131.34	140.04	137.58	127.96	123.30	131.92
9	142.72	136.83	133.00	133.46	134.41	133.41	128.85	129.06
10	136.48	126.73	135.41	135.30	135.63	126.17	132.92	134.17

Table S8: Raw APVs of fluorescent images of isotonically treated RAW264.7 population

	Circularity	*Octant 1*	*Octant 2*	*Octant 3*	*Octant 4*	*Octant 5*	*Octant 6*	*Octant 7*	*Octant 8*
Nucleus									
Field 1									
1	0.45	4.71	1.46	1.98	5.71	3.99	1.23	2.61	5.85
2	0.19	1.51	3.87	2.59	4.50	1.71	3.58	2.59	4.83
3	0.90	3.64	4.78	9.44	4.10	4.20	3.43	3.73	3.58
4	0.39	0.56	0.61	3.96	4.20	1.27	1.46	3.34	3.98
5	0.30	2.03	1.11	1.42	0.77	2.16	1.86	2.60	1.32
6	0.29	2.43	3.86	1.46	1.67	2.50	3.24	1.09	1.59
7	0.65	2.28	3.74	3.75	3.88	2.21	2.96	3.07	3.09
8	0.24	0.07	0.15	4.49	4.53	0.49	0.85	2.90	3.43
9	0.29	1.62	7.67	1.08	4.59	1.71	7.69	1.23	3.50
10	0.53	4.23	1.22	3.74	1.94	5.90	1.92	5.57	2.87

11	0.40	0.72	8.18	3.83	0.11	1.16	8.11	3.74	0.35
12	0.98	4.13	1.68	3.29	7.89	3.26	0.87	2.46	10.08
13	0.91	5.72	3.87	2.95	6.83	9.06	2.60	1.46	9.72
14	0.61	5.76	6.90	4.62	6.28	4.89	4.57	2.39	4.86
15	0.45	4.53	4.09	4.24	1.46	5.73	4.65	5.62	1.24
16	0.34	6.95	1.14	2.13	4.60	6.91	0.47	1.60	6.30
17	1.01	7.60	7.04	9.23	9.77	4.63	4.20	6.23	6.28
18	0.98	1.76	1.94	8.67	7.38	1.10	1.26	8.71	6.06
19	0.65	4.03	2.55	3.50	5.02	5.08	2.71	5.00	7.56
Field 2									
1	0.96	5.38	1.58	2.10	6.66	4.15	0.55	1.16	5.10
2	0.99	4.35	7.69	5.69	2.47	2.19	4.86	3.26	1.54
3	0.67	5.86	3.60	1.49	1.92	5.05	3.14	0.77	1.12
4	1.01	4.46	2.89	4.00	6.03	2.39	1.99	2.08	3.51
5	0.60	1.65	7.57	4.80	1.49	1.30	3.75	2.76	1.42
6	0.56	1.84	1.65	2.14	5.78	0.79	0.98	1.23	3.77
7	0.96	4.79	3.39	1.68	7.15	3.14	1.53	0.92	4.28
8	1.00	7.04	10.53	6.40	3.57	1.40	2.64	2.35	1.15
9	0.95	4.98	7.56	9.47	2.95	2.05	3.66	3.61	1.16
10	1.00	4.28	4.15	3.88	5.45	2.46	1.23	1.99	3.47
11	0.82	0.60	2.91	4.75	3.36	0.60	3.37	5.18	2.92
12	0.98	8.56	6.30	0.96	3.13	4.14	3.70	0.84	1.68
13	0.72	0.70	8.85	7.89	0.99	0.69	3.78	4.06	0.89
14	0.96	6.08	8.64	4.94	5.54	2.44	3.25	1.44	1.68
15	0.63	6.57	0.80	2.42	4.47	4.87	0.65	1.59	3.26
16	0.80	2.02	5.73	3.56	3.89	2.30	4.00	2.43	3.09
17	0.32	0.55	2.59	5.31	2.07	0.45	1.15	3.57	1.22
18	0.53	1.20	4.72	4.06	2.59	1.55	3.39	2.76	2.27
19	1.01	6.29	4.98	5.62	7.43	3.45	4.02	3.77	2.37
Field 3									
1	1.00	3.24	2.76	0.36	1.57	3.90	2.19	0.24	1.07
2	0.98	0.54	0.58	3.37	3.64	0.32	0.48	3.12	3.68
3	0.42	0.32	1.52	0.49	1.29	1.14	3.68	1.04	4.24
4	0.51	0.40	1.12	0.51	0.46	1.06	2.11	1.42	1.88
5	1.00	2.66	1.45	1.42	3.69	2.60	1.46	1.49	2.79
6	0.99	1.37	2.35	1.67	1.13	1.46	3.04	1.54	1.05
7	0.45	0.84	0.24	0.67	0.99	1.38	0.61	1.07	0.99
8	0.95	1.78	3.62	2.15	0.96	1.10	3.37	2.13	0.61
9	0.98	2.16	1.94	3.03	3.75	1.73	1.48	2.96	3.88
10	0.63	0.32	2.07	3.93	1.03	1.04	2.56	1.91	1.60
11	0.90	1.19	1.54	2.73	1.98	1.29	1.85	3.28	2.57
12	0.89	2.19	0.93	1.32	1.75	2.82	1.12	1.19	2.56
13	1.02	2.05	3.78	5.94	5.07	1.00	2.07	1.82	1.49

14	1.02	5.09	8.62	4.31	4.53	1.55	1.58	2.85	2.31
15	0.92	2.48	1.83	3.20	3.20	2.92	1.34	1.91	3.00
16	0.62	1.71	0.61	1.58	1.60	1.01	0.65	2.09	2.46
17	0.97	1.57	2.61	2.56	0.90	1.75	2.40	2.39	0.88
18	0.72	4.21	5.08	1.95	2.05	2.95	5.13	2.26	1.42
19	1.00	2.39	2.38	3.03	2.22	2.22	1.56	2.27	2.76
20	0.99	2.82	5.21	4.06	2.34	1.55	2.46	2.70	1.20
21	0.98	5.03	5.17	5.74	3.10	1.97	1.81	3.12	1.34
22	0.96	4.82	3.98	2.04	1.59	2.83	2.49	0.88	0.96
23	0.99	3.20	2.27	0.57	2.86	3.30	1.75	0.59	2.24
24	0.88	3.19	4.66	2.26	1.32	3.18	2.51	1.93	1.24
25	0.96	5.33	1.27	0.76	4.32	3.10	0.81	0.52	2.83
26	1.00	4.46	2.40	1.72	3.45	3.00	1.37	1.59	3.39
27	0.76	0.42	1.00	2.02	0.85	1.14	2.04	3.18	1.50
28	0.97	4.76	4.23	1.36	1.90	2.12	2.80	0.72	0.78
29	1.02	5.50	3.69	2.39	4.36	2.51	1.41	1.32	1.82
30	1.00	1.10	2.81	3.61	1.12	0.85	2.66	4.06	1.62
31	0.98	3.34	1.29	2.02	2.08	1.94	0.70	1.16	2.46
32	0.98	1.86	1.88	1.87	0.49	2.20	2.32	1.33	0.99
33	0.98	3.26	2.92	1.53	4.03	2.09	2.83	1.88	2.71
34	0.96	0.75	1.06	1.03	1.41	1.74	1.38	1.54	2.94
35	1.01	0.72	1.46	0.48	0.17	2.51	3.82	2.06	0.55
36	0.86	0.51	1.08	1.08	0.59	2.98	2.73	2.27	1.90
37	0.99	1.47	2.10	1.73	1.14	2.10	2.86	2.38	2.18
38	1.01	2.04	2.74	3.44	1.16	1.70	2.57	3.43	1.28
39	0.99	4.50	3.33	0.56	1.38	4.73	2.87	0.37	1.30
40	0.97	3.75	5.47	5.32	1.40	1.55	3.47	3.27	0.92
41	0.92	2.68	2.81	2.46	1.25	2.26	2.75	2.33	0.91
42	0.99	3.72	1.75	2.28	3.70	2.82	2.17	2.85	2.77
43	1.01	6.03	0.46	0.65	6.48	4.07	0.51	0.77	4.74
44	0.98	1.32	3.73	3.97	2.78	0.99	2.48	3.51	2.30
45	0.91	3.75	3.22	2.89	3.90	1.10	2.02	1.99	1.37
46	0.96	3.92	3.56	3.76	2.38	2.55	1.74	2.46	1.95
Field 4									
1	0.98	2.20	1.41	3.16	4.37	1.55	1.11	1.97	2.64
2	0.99	0.69	1.23	1.17	0.96	1.42	1.31	1.67	1.77
3	0.45	0.97	0.25	0.61	1.32	1.76	0.72	1.34	1.90
4	0.65	0.30	0.65	2.88	0.78	0.70	0.90	3.41	1.29
5	1.00	2.64	1.19	2.50	3.57	2.37	1.14	1.94	2.30
6	0.67	3.26	4.33	2.23	7.28	2.26	3.57	1.73	3.39
7	0.70	4.71	5.36	3.26	7.32	2.54	1.30	0.59	2.77
8	1.03	3.60	5.03	2.86	2.49	2.25	2.69	2.13	1.59
9	0.99	7.75	4.62	4.10	4.33	2.65	1.63	1.25	1.65

10	0.97	4.86	3.60	2.98	3.35	2.01	1.01	0.99	1.41
11	0.87	4.84	3.51	4.41	3.25	2.17	1.97	2.01	1.50
12	0.97	3.04	3.54	4.67	2.75	2.20	2.76	3.06	1.49
13	0.98	2.04	1.55	3.68	5.48	1.33	1.12	2.00	2.81
14	0.99	2.08	1.75	2.17	3.28	1.98	1.43	1.70	2.39
15	0.99	2.01	1.41	1.59	2.32	2.60	1.77	1.61	2.25
16	0.94	2.33	0.72	2.71	3.80	2.62	0.75	1.97	4.30
17	0.91	3.86	1.73	1.52	3.05	3.52	1.25	0.84	1.96
18	1.00	0.82	1.16	3.12	2.81	0.43	0.57	1.90	1.71
19	0.74	1.54	1.20	3.07	1.61	1.70	1.16	2.47	1.78
20	1.01	2.45	3.72	0.58	0.48	1.23	2.12	0.47	0.36
21	0.98	3.09	1.12	1.55	3.09	1.54	0.68	0.75	1.86
22	0.64	1.91	1.72	2.74	2.66	0.93	1.50	1.94	1.13
23	0.46	1.02	1.62	3.04	0.44	0.38	0.67	2.26	0.43
24	0.80	2.58	0.68	1.91	2.73	1.81	0.60	1.42	1.95
25	0.99	2.37	3.94	4.13	2.05	1.11	2.48	1.63	1.10
26	0.90	3.04	1.76	2.06	3.60	1.17	0.82	1.04	1.42
27	1.02	1.55	3.19	5.40	4.14	0.54	0.91	1.81	1.34
28	1.02	1.41	5.88	6.93	3.22	0.36	0.66	0.73	0.50
29	0.95	5.64	4.00	1.39	0.91	2.20	2.23	1.13	0.82
30	1.00	3.73	2.53	1.88	4.35	2.05	1.38	0.90	2.13
31	0.96	4.06	3.26	5.71	3.23	1.70	0.98	2.32	1.91
32	0.99	6.47	5.08	2.78	5.46	1.00	0.85	0.83	1.00
33	0.98	5.75	4.23	2.47	2.39	1.94	0.83	0.61	0.76
34	0.97	5.39	3.28	3.72	5.44	1.86	0.81	1.58	2.97
35	0.94	3.52	3.29	5.13	5.36	0.92	0.70	1.50	2.65
36	1.01	4.29	9.21	8.85	4.19	1.03	2.30	1.83	1.11
37	0.92	5.33	2.51	1.70	4.71	0.92	0.47	0.72	1.32
Cell Membrane									
Field 1									
1	0.99	6.38	6.59	6.62	8.44	12.79	10.08	16.16	18.37
2	0.96	13.62	7.16	8.04	9.54	15.88	14.82	18.12	11.27
3	1.02	7.50	4.60	10.21	13.51	13.91	8.97	13.89	14.18
4	0.77	15.61	9.35	7.31	8.63	21.57	13.64	12.80	16.97
5	1.01	11.44	15.86	13.29	9.89	15.43	22.13	15.69	11.72
6	1.00	12.05	14.85	11.48	11.99	18.30	21.05	17.83	21.28
7	1.00	25.73	27.43	32.34	18.24	13.60	9.09	16.09	12.64
8	0.97	23.98	21.32	29.26	14.34	26.50	25.32	19.81	14.38
9	0.94	24.85	14.86	14.36	25.53	15.01	14.07	11.31	15.18
10	0.90	7.32	4.10	19.51	21.89	7.71	7.80	20.07	17.45
11	0.60	22.91	15.33	8.00	4.53	23.43	16.94	11.58	11.93
12	0.64	6.69	6.01	11.61	9.37	15.87	12.66	25.01	22.68
13	0.98	7.10	16.97	6.69	6.51	10.17	20.41	13.92	10.03

14	0.94	15.39	18.79	20.07	19.41	29.69	22.73	18.53	25.03
15	0.63	7.69	5.98	13.31	10.85	18.04	15.16	25.20	21.17
16	0.43	5.98	7.38	7.34	7.85	13.34	19.48	12.76	16.06
17	0.45	4.11	4.87	3.46	4.82	11.99	13.17	8.98	11.47
18	1.02	7.99	9.45	15.95	16.10	12.73	10.24	16.56	17.44
Field 2									
1	0.44	5.14	7.01	21.28	4.79	14.84	11.91	27.35	13.37
2	0.92	11.09	10.47	10.15	9.87	18.49	20.31	27.91	11.53
3	0.37	10.73	16.27	11.03	10.11	18.69	20.79	17.38	16.20
4	0.65	4.50	6.18	4.39	7.92	11.73	12.21	9.54	14.05
5	0.99	15.34	20.54	21.88	11.92	13.34	21.91	18.82	12.53
6	0.30	2.83	5.36	9.47	7.37	7.70	15.65	13.40	14.89
7	0.64	12.91	9.81	7.95	14.00	24.89	16.24	16.40	11.59
8	0.99	15.40	20.77	22.14	19.86	15.45	20.41	17.35	17.16
9	0.32	8.39	20.07	14.87	22.85	18.62	31.17	21.26	15.20
10	1.00	14.91	10.55	9.35	10.86	41.22	14.42	20.51	23.27
11	0.88	7.82	9.30	14.90	10.89	26.49	26.64	33.89	28.21
12	0.67	11.62	14.68	9.10	7.11	15.77	12.18	15.54	14.56
13	0.97	14.79	9.46	15.46	19.64	15.88	15.69	20.45	12.45
14	0.59	10.85	5.60	14.21	11.21	24.66	18.82	22.56	15.30
15	0.95	13.68	16.47	13.77	12.34	24.25	17.36	17.92	19.19
16	0.59	13.11	12.19	9.71	19.18	15.93	18.46	21.23	30.60
17	0.99	19.74	12.34	9.03	9.73	15.56	15.79	11.41	14.56
18	0.62	5.62	10.42	17.17	8.68	13.37	18.08	13.87	12.54
19	0.82	11.25	13.40	14.66	17.39	12.64	16.09	17.59	13.96
20	0.98	17.72	9.83	8.48	20.88	15.17	9.74	9.68	11.87
21	0.92	12.03	14.95	11.33	6.42	14.55	11.26	14.87	14.94
22	0.95	17.18	9.74	17.96	14.43	17.32	11.77	13.41	18.78
23	0.93	12.03	8.24	8.02	8.79	12.77	14.20	14.19	10.37
24	0.83	4.60	4.29	6.14	6.87	16.89	16.11	17.26	21.04
25	0.94	24.71	40.65	37.52	29.06	26.89	28.43	30.44	25.69
26	0.94	9.13	5.41	4.01	7.25	9.73	9.46	14.44	10.97
27	0.68	14.43	8.93	8.39	9.82	31.58	34.14	14.81	14.73
28	0.28	14.39	2.80	2.59	6.89	23.40	11.25	11.48	14.10
29	0.62	5.81	3.79	2.71	3.53	22.66	14.73	11.07	13.32
30	0.65	4.96	9.64	7.94	6.08	12.24	14.31	14.02	14.02
Field 3									
1	0.39	5.16	5.01	8.05	4.29	14.26	17.26	13.89	12.31
2	0.85	18.95	19.48	20.83	13.42	25.89	20.29	17.56	24.74
3	0.98	30.51	21.90	31.39	36.99	27.29	22.46	29.84	38.60
4	0.99	18.47	14.44	9.25	13.31	19.15	15.62	18.20	22.92
5	1.02	13.40	9.27	8.21	15.48	18.74	14.31	12.26	20.42
6	1.00	18.78	12.68	19.46	18.54	15.15	15.43	18.59	12.84

7	0.98	19.32	17.39	13.30	9.95	10.70	11.82	13.53	7.78
8	0.99	14.50	12.06	14.06	10.28	15.68	14.05	17.67	11.51
9	0.47	5.28	7.18	8.46	8.34	15.31	9.74	17.41	14.55
10	0.80	11.67	16.08	13.83	13.63	27.41	30.46	27.52	27.99
11	0.26	15.66	5.76	9.55	5.16	28.85	10.43	22.78	20.50
12	0.32	4.96	8.25	4.56	4.57	12.10	18.62	9.46	13.61
13	0.54	6.58	5.38	5.49	9.80	11.25	8.08	9.86	22.75
14	0.97	17.17	21.63	16.09	15.21	18.91	23.36	21.91	15.41
15	0.99	12.74	11.58	10.25	7.06	12.33	7.54	9.09	9.94
16	0.99	5.43	3.66	6.16	6.27	5.91	6.88	8.50	8.27
17	1.01	7.84	8.80	8.72	10.72	13.09	14.21	10.68	11.75
18	0.53	12.54	5.16	9.47	13.96	15.84	13.23	23.86	18.51
19	0.60	6.90	10.04	3.29	4.42	10.02	15.13	11.28	8.13
20	0.96	15.00	17.48	13.38	17.13	21.44	24.43	18.13	17.59
21	0.94	19.86	21.76	13.18	16.65	21.96	14.97	20.89	24.92
22	0.89	4.01	6.29	3.11	4.16	8.84	15.16	7.33	9.06
23	0.82	5.91	6.94	4.64	4.33	11.18	12.20	11.46	8.43
24	0.41	7.85	21.92	4.98	5.91	17.35	23.25	14.58	15.45
25	1.00	20.28	22.24	22.21	23.11	24.36	23.36	27.40	21.60
26	0.19	4.76	6.66	8.45	9.98	13.59	18.60	15.94	15.22
27	0.21	3.19	3.54	5.29	2.05	20.48	11.66	13.90	7.92
28	0.46	5.65	8.34	15.08	9.13	19.84	24.15	24.07	17.94
29	1.02	14.60	13.64	16.38	14.92	16.23	19.10	20.67	24.99
30	0.34	10.77	10.61	4.69	5.28	11.93	24.39	15.48	14.67
31	0.97	15.85	9.49	11.55	13.46	33.10	21.78	13.34	15.58
Field 4									
1	0.63	27.43	31.55	9.87	33.31	28.99	30.71	16.60	22.69
2	0.98	16.99	24.97	26.73	27.21	23.24	28.85	23.50	17.16
3	0.82	7.86	12.36	21.27	17.70	13.15	19.15	34.06	25.03
4	0.20	7.50	6.43	14.46	12.42	26.88	17.22	25.42	23.58
5	0.89	25.95	14.57	15.26	22.79	18.43	9.71	10.96	19.72
6	0.96	22.20	29.08	20.66	19.77	20.43	26.21	19.61	19.90
7	1.01	19.22	15.45	16.32	22.97	26.95	17.68	19.35	18.55
8	0.90	16.36	20.57	14.47	14.97	18.35	22.96	16.46	10.74
9	0.40	3.50	3.13	10.29	4.46	27.11	21.23	29.18	17.43
10	0.98	22.70	20.14	22.39	24.57	23.54	11.93	14.27	24.98
11	0.90	25.30	30.25	37.56	32.58	26.20	20.28	24.01	18.46
12	0.99	19.72	29.99	14.21	11.80	13.00	13.35	12.83	11.08
13	0.73	17.17	32.59	27.93	13.92	16.66	17.38	14.66	19.31
14	0.49	4.35	2.63	4.24	2.95	15.04	12.53	19.70	12.81
15	0.49	4.73	6.23	4.82	5.45	14.54	17.81	22.04	16.48
16	0.25	23.38	4.21	6.95	18.80	33.16	19.43	23.41	25.52
17	0.69	3.69	6.58	9.94	2.69	17.21	21.69	29.41	14.41

18	0.86	7.74	13.19	5.45	3.55	26.34	26.32	20.08	21.40
19	0.56	14.20	18.46	23.00	8.12	22.67	20.94	26.43	26.73
20	0.94	12.02	12.75	10.85	12.44	17.90	24.17	15.90	25.35
Mitochondria									
Field 1									
1	0.45	6.78	6.36	5.82	3.87	5.22	6.89	6.05	3.89
2	0.19	5.29	3.99	5.26	16.06	4.72	9.69	8.00	10.04
3	0.90	15.02	43.94	27.52	9.45	22.93	45.73	39.41	20.57
4	0.39	0.67	0.72	1.59	2.43	5.99	5.34	9.04	8.55
5	0.30	1.57	1.55	0.32	0.53	5.17	4.54	1.47	4.59
6	0.29	9.19	12.23	8.82	10.82	13.49	18.37	18.67	14.27
7	0.65	2.91	8.58	5.70	5.50	4.43	8.47	8.48	5.44
8	0.24	0.20	0.16	2.99	7.08	1.53	0.84	4.35	4.23
9	0.29	5.06	14.53	4.16	9.06	14.69	22.82	14.59	18.78
10	0.53	2.85	2.16	2.72	1.75	2.89	3.55	2.86	3.44
11	0.40	7.31	10.83	11.10	3.78	13.08	7.88	9.41	5.86
12	0.98	3.83	2.63	3.27	7.85	4.60	4.42	4.65	1.22
13	0.91	7.99	9.70	4.88	2.27	2.96	12.46	6.95	3.14
14	0.61	5.00	7.81	11.32	7.24	4.08	7.14	13.02	8.54
15	0.45	6.47	1.81	5.62	2.22	5.50	3.48	4.42	4.72
16	0.34	10.07	0.12	1.45	6.28	12.13	1.00	6.92	6.59
17	1.01	10.34	8.11	6.85	8.87	15.52	13.87	11.33	9.25
18	0.98	7.35	5.08	4.97	9.68	7.38	6.16	3.71	7.12
19	0.65	5.76	8.06	14.25	10.56	3.63	6.32	9.46	3.65
Field 2									
1	0.96	2.10	3.70	3.80	2.47	1.43	3.72	2.57	1.70
2	0.99	9.56	6.59	7.44	11.06	4.03	2.83	4.73	4.47
3	0.67	23.22	15.67	19.04	21.27	22.26	19.50	17.14	27.99
4	1.01	14.81	18.06	19.72	12.30	9.85	10.36	14.29	8.21
5	0.60	5.94	5.42	3.12	3.72	4.78	2.28	1.40	2.20
6	0.56	9.52	10.70	4.81	9.43	5.19	4.29	4.02	3.24
7	0.96	3.39	11.20	8.08	1.92	1.56	4.54	4.60	0.52
8	1.00	5.81	3.04	7.33	8.34	5.41	4.85	4.19	4.10
9	0.95	7.98	2.85	5.94	11.48	3.25	1.36	4.33	4.83
10	1.00	4.21	5.03	7.89	8.59	1.54	3.98	4.63	2.92
11	0.82	4.99	3.85	2.97	6.51	11.53	7.79	8.01	13.21
12	0.98	14.86	17.75	16.82	12.38	5.96	7.58	7.47	6.08
13	0.72	15.55	13.31	9.60	13.62	10.15	7.35	5.76	10.05
14	0.96	7.31	6.18	4.49	4.06	3.46	2.87	3.28	2.51
15	0.63	7.66	1.47	6.05	9.90	4.83	2.38	7.37	7.14
16	0.80	3.96	8.10	7.13	6.59	4.20	6.59	7.39	6.92
17	0.32	3.47	6.94	11.71	6.98	5.26	7.04	8.41	6.05
18	0.53	2.53	6.14	7.47	5.16	2.96	5.10	7.28	5.83

19	1.01	7.07	7.56	9.78	10.24	6.52	3.10	4.64	9.84
Field 3									
1	1.00	0.74	1.51	3.98	1.35	0.11	1.42	2.53	1.74
2	0.98	3.30	4.06	8.38	8.74	2.66	2.04	2.64	1.80
3	0.42	0.25	1.21	0.58	0.75	4.57	3.70	5.44	4.06
4	0.51	1.29	4.39	1.83	1.10	3.57	6.99	6.64	3.79
5	1.00	3.20	4.97	4.91	4.93	1.30	3.02	4.82	1.47
6	0.99	1.01	1.06	3.19	2.33	0.21	0.78	3.53	3.75
7	0.45	0.80	0.25	0.54	1.43	3.86	2.71	5.21	5.09
8	0.95	3.12	1.54	2.10	3.31	1.72	0.58	1.53	2.55
9	0.98	5.60	3.72	0.61	3.13	2.55	3.41	0.17	0.41
10	0.63	1.19	5.69	10.83	3.03	6.27	4.34	6.78	7.45
11	0.90	2.43	5.10	8.95	2.69	1.70	2.24	1.16	1.04
12	0.89	2.47	3.79	3.85	0.56	1.39	4.82	2.94	0.08
13	1.02	5.59	4.31	1.48	3.26	3.39	2.19	2.01	4.78
14	1.02	6.23	6.02	3.62	0.90	4.63	4.45	1.19	1.07
15	0.92	0.98	1.75	1.51	2.94	0.47	2.70	2.78	1.62
16	0.62	2.73	5.93	1.48	0.44	6.18	6.56	5.44	1.99
17	0.97	1.93	1.66	5.19	3.78	2.28	1.90	2.90	3.09
18	0.72	7.26	1.95	1.10	6.77	3.70	2.00	2.14	6.17
19	1.00	0.87	4.46	1.60	0.59	0.59	3.68	1.89	0.36
20	0.99	3.19	1.86	1.34	2.11	2.70	1.15	0.22	1.45
21	0.98	1.84	0.96	1.05	2.05	2.18	0.64	0.58	1.20
22	0.96	0.82	3.30	0.47	0.98	0.81	0.95	1.96	1.91
23	0.99	1.03	4.57	8.87	4.82	0.43	2.59	8.25	2.97
24	0.88	3.94	3.23	3.23	1.60	1.03	1.08	1.99	1.29
25	0.96	3.33	9.92	11.87	4.81	0.97	8.84	7.21	2.98
26	1.00	2.99	8.14	6.65	8.39	1.01	2.11	2.89	1.66
27	0.76	2.71	0.40	1.32	3.51	3.19	0.53	0.51	3.57
28	0.97	2.16	2.24	5.56	4.90	4.13	2.28	6.08	4.33
29	1.02	0.14	2.00	2.49	0.67	0.16	0.49	1.23	0.73
30	1.00	4.53	1.92	1.11	1.65	2.27	1.21	0.43	1.43
31	0.98	2.22	4.16	6.14	4.91	2.48	3.07	4.42	3.29
32	0.98	0.79	1.00	0.98	1.08	1.36	1.52	1.70	1.09
33	0.98	7.66	2.67	4.24	6.96	4.02	1.72	2.26	5.30
34	0.96	2.50	1.35	0.67	0.87	2.24	2.65	3.09	1.60
35	1.01	0.53	2.03	0.51	0.49	0.67	0.52	1.14	3.05
36	0.86	0.48	0.68	3.36	1.50	1.09	0.68	2.00	2.15
37	0.99	5.30	1.97	0.33	1.28	7.04	1.40	0.38	4.18
38	1.01	6.99	7.37	7.03	7.53	5.88	3.84	5.89	6.90
39	0.99	4.15	3.61	3.88	2.78	0.21	1.49	4.12	4.17
40	0.97	5.62	2.68	3.99	7.14	4.08	0.43	0.49	4.50
41	0.92	8.60	4.38	4.15	5.55	3.49	0.96	3.81	8.38

42	0.99	8.78	5.71	5.59	2.89	4.06	5.47	4.37	2.80
43	1.01	5.66	7.96	8.39	3.85	3.95	6.39	8.05	2.98
44	0.98	6.83	3.40	6.03	7.42	4.46	1.98	3.20	3.84
45	0.91	5.42	2.61	2.80	2.54	4.12	3.46	3.96	4.50
46	0.96	2.06	2.28	11.04	4.70	1.00	2.19	4.50	3.51
Field 4									
1	0.98	8.17	10.24	7.57	6.97	3.03	2.36	2.15	2.43
2	0.99	8.95	3.32	2.19	3.60	5.07	1.45	0.77	1.52
3	0.45	1.18	1.94	4.16	2.01	1.19	3.04	3.97	1.03
4	0.65	2.03	4.51	1.04	4.36	3.69	4.46	0.86	3.32
5	1.00	3.47	3.38	4.13	3.36	2.98	3.60	4.34	4.05
6	0.67	7.67	6.44	2.88	4.43	7.10	2.71	4.70	7.59
7	0.70	6.38	7.03	4.85	4.76	4.57	9.11	7.01	4.32
8	1.03	6.17	9.18	4.75	3.31	3.31	2.29	1.86	2.94
9	0.99	1.97	11.35	12.17	2.50	0.79	4.95	5.36	1.91
10	0.97	2.84	6.67	5.03	4.63	0.72	3.06	2.65	2.40
11	0.87	6.09	10.44	6.31	8.58	3.63	3.09	2.54	1.98
12	0.97	8.97	5.70	5.19	5.29	0.62	0.70	0.08	0.12
13	0.98	3.87	10.21	5.48	1.93	1.52	1.71	1.81	0.61
14	0.99	1.62	1.51	1.27	4.28	0.40	0.74	0.55	0.74
15	0.99	1.48	4.46	6.79	5.02	0.65	1.86	3.15	3.15
16	0.94	3.23	3.41	1.86	1.68	1.71	3.84	1.49	1.97
17	0.91	5.68	5.04	8.32	6.61	0.68	2.47	4.84	2.99
18	1.00	12.76	13.79	11.53	12.24	5.50	4.53	2.08	3.27
19	0.74	0.20	3.77	0.54	0.14	0.47	3.32	1.65	0.55
20	1.01	1.50	1.11	4.29	3.84	1.87	0.86	2.23	3.36
21	0.98	1.31	3.91	5.88	3.04	1.17	2.87	2.31	0.26
22	0.64	4.24	2.06	0.84	2.75	1.81	1.34	0.86	2.91
23	0.46	4.11	3.24	2.90	0.81	7.65	6.50	2.39	1.98
24	0.80	5.13	5.10	6.02	0.56	4.27	5.61	6.97	1.84
25	0.99	7.32	4.47	7.77	12.96	5.30	1.34	3.88	5.38
26	0.90	7.48	5.98	3.98	3.59	5.97	4.32	4.89	4.30
27	1.02	15.98	8.73	10.45	11.37	6.64	6.01	4.96	3.90
28	1.02	8.26	7.98	8.80	9.29	5.79	8.57	3.18	3.55
29	0.95	7.24	4.22	10.07	12.34	1.83	0.90	2.70	3.15
30	1.00	7.08	13.09	12.71	3.48	1.65	5.73	5.77	3.43
31	0.96	2.74	3.05	4.03	6.55	0.38	1.16	0.54	1.14
32	0.99	2.04	6.07	7.29	4.90	0.92	1.64	1.78	1.38
33	0.98	1.51	2.84	2.70	1.60	0.66	2.29	1.93	1.54
34	0.97	6.74	8.51	3.84	5.41	4.24	6.91	5.01	1.67
35	0.94	4.54	7.36	9.89	7.18	4.80	6.04	5.91	2.40
36	1.01	4.18	3.76	4.91	6.78	5.09	3.23	4.80	4.73
37	0.92	5.66	5.71	3.34	2.86	2.87	2.96	1.49	2.91

Endoplasmic Reticulum									
Field 1									
1	0.99	5.41	4.96	5.41	5.88	5.60	4.45	4.87	5.35
2	0.96	6.70	5.93	6.48	6.37	6.29	6.77	5.64	4.09
3	1.02	5.98	4.79	6.82	8.86	4.06	3.37	5.35	6.18
4	0.77	9.71	6.67	7.22	7.44	7.38	4.01	6.03	6.84
5	1.01	7.38	8.95	7.01	6.37	7.61	7.43	4.41	4.75
6	1.00	6.54	7.16	7.36	7.99	5.48	7.19	5.98	6.56
7	1.00	10.44	8.82	10.58	6.58	7.48	4.11	5.92	4.69
8	0.97	9.12	9.05	10.25	6.30	6.76	8.75	6.83	5.42
9	0.94	10.51	7.54	7.15	7.76	6.44	6.44	7.22	5.81
10	0.90	5.71	5.54	9.55	8.74	3.45	2.94	7.38	7.20
11	0.60	11.65	8.63	6.23	5.10	6.81	7.93	3.69	3.03
12	0.64	5.85	6.12	8.29	8.22	6.40	3.70	5.64	8.63
13	0.98	4.99	6.97	4.92	5.06	6.71	6.62	5.45	6.96
14	0.94	4.37	5.37	4.70	6.47	6.93	6.63	5.56	8.12
15	0.63	2.70	2.26	4.34	3.90	6.26	5.42	6.51	5.99
16	0.43	2.24	2.89	2.48	2.92	4.44	6.33	4.65	5.11
17	0.45	2.31	2.43	1.51	2.00	4.27	5.14	3.60	4.32
18	1.02	3.33	3.48	4.03	4.53	5.69	4.26	4.71	5.61
Field 2									
1	0.44	4.44	5.04	10.13	4.68	4.27	4.52	7.72	4.48
2	0.92	7.81	5.37	5.58	7.04	5.32	5.41	7.71	4.95
3	0.37	5.21	7.55	5.65	5.76	5.22	6.03	6.22	4.55
4	0.65	3.12	3.89	3.26	4.09	5.14	4.76	3.42	6.37
5	0.99	6.77	10.24	9.79	6.38	5.26	8.00	6.93	3.57
6	0.30	3.71	4.70	5.91	4.22	1.84	5.06	6.03	4.53
7	0.64	6.91	7.13	6.03	7.56	8.03	7.84	7.31	5.08
8	0.99	6.10	8.73	9.23	7.45	4.69	6.18	7.99	8.11
9	0.32	5.45	7.08	6.21	7.64	6.49	7.07	5.54	4.84
10	1.00	7.79	5.97	6.33	6.57	9.86	4.63	6.02	6.70
11	0.88	5.51	6.02	7.21	6.85	7.85	6.42	9.67	7.88
12	0.67	6.71	8.77	5.92	4.77	5.17	4.39	5.81	4.81
13	0.97	6.77	5.79	6.95	7.06	6.35	5.90	6.20	5.31
14	0.59	6.29	5.51	8.48	6.54	7.28	4.58	6.99	5.29
15	0.95	7.75	7.82	7.67	7.07	9.42	7.02	5.47	7.61
16	0.59	8.18	6.77	7.06	9.78	6.09	5.84	6.26	8.84
17	0.99	7.84	5.13	4.27	5.08	5.97	5.29	5.05	6.20
18	0.62	4.31	4.86	6.30	4.73	4.57	6.53	4.66	5.25
19	0.82	5.60	6.50	5.94	7.24	5.46	7.68	7.20	5.56
20	0.98	11.56	5.50	5.11	9.36	8.07	4.93	3.96	5.81
21	0.92	7.15	6.35	4.46	3.63	6.64	4.71	6.16	6.83
22	0.95	6.64	4.08	5.18	4.45	6.73	5.47	5.84	6.01

23	0.93	5.52	5.31	4.63	4.96	5.58	5.71	6.68	6.45
24	0.83	2.67	2.81	3.39	2.76	6.29	5.42	5.22	6.97
25	0.94	6.62	11.95	9.33	6.58	5.67	7.57	6.16	5.47
26	0.94	5.23	3.92	3.41	4.23	4.71	5.34	5.76	6.93
27	0.68	8.07	6.05	5.39	6.06	10.45	7.68	4.16	5.26
28	0.28	5.62	2.95	2.67	3.20	6.55	2.82	2.45	6.56
29	0.62	2.28	2.10	1.79	1.73	5.95	4.63	3.93	3.59
30	0.65	5.18	8.25	6.91	5.57	5.11	5.49	5.80	5.57
Field 3									
1	0.39	1.27	1.32	1.72	1.01	3.53	4.04	3.70	3.33
2	0.85	4.14	5.06	4.99	2.80	7.29	4.50	4.77	5.89
3	0.98	7.48	6.64	5.26	4.09	4.50	5.87	7.35	5.82
4	0.99	4.90	3.75	3.74	3.67	5.16	5.25	8.20	7.78
5	1.02	4.33	3.58	3.44	4.45	6.46	6.65	6.27	5.47
6	1.00	9.05	5.27	5.70	7.22	7.85	5.53	4.57	4.37
7	0.98	4.55	4.32	4.76	3.85	3.76	4.75	4.64	3.12
8	0.99	4.62	4.26	6.39	4.78	4.89	4.29	6.19	3.85
9	0.47	2.98	4.10	3.72	5.33	5.55	5.25	4.86	5.29
10	0.80	5.83	7.56	7.58	5.95	6.79	7.67	6.43	5.34
11	0.26	7.26	5.07	5.45	3.23	6.29	2.09	5.44	4.74
12	0.32	2.44	3.69	2.03	2.10	3.79	6.69	2.84	4.80
13	0.54	3.50	2.67	2.15	3.79	4.85	3.86	5.59	7.14
14	0.97	5.69	7.69	4.94	4.27	5.08	8.00	7.47	3.80
15	0.99	5.25	3.29	3.84	2.56	7.71	4.33	3.84	4.63
16	0.99	1.21	1.43	2.76	2.69	2.88	3.28	4.98	6.57
17	1.01	3.09	4.29	3.91	3.68	5.33	7.19	4.89	4.16
18	0.53	5.03	1.76	2.32	4.10	5.71	4.39	7.63	5.28
19	0.60	2.82	3.29	1.06	1.44	3.98	6.33	4.02	4.20
20	0.96	4.17	6.21	3.85	4.01	7.78	7.16	5.05	7.24
21	0.94	4.47	6.52	3.36	3.11	7.17	5.08	5.78	6.99
22	0.89	1.18	1.99	1.25	1.73	3.27	5.09	4.17	5.91
23	0.82	2.63	3.03	1.61	2.19	5.05	7.35	4.92	3.67
24	0.41	3.22	7.14	2.67	3.71	6.56	7.38	6.45	6.28
25	1.00	6.48	7.14	8.99	8.41	5.23	6.80	11.48	7.21
26	0.19	1.73	2.20	3.26	3.19	3.37	3.76	5.80	4.29
27	0.21	2.07	2.99	2.77	1.61	4.56	3.76	5.08	2.41
28	0.46	3.34	4.19	5.97	4.61	6.53	7.02	5.99	7.53
29	1.02	5.72	6.92	6.75	5.86	3.85	7.97	7.48	5.89
30	0.34	5.00	4.86	2.17	2.90	5.15	7.23	3.17	4.54
31	0.97	4.80	4.51	6.06	6.49	7.28	6.39	5.49	6.07
Field 4									
1	0.63	9.02	9.51	5.20	10.60	7.17	7.20	5.38	5.54
2	0.98	6.43	9.32	10.63	9.47	4.97	8.00	7.21	5.41

3	0.82	5.32	7.02	8.09	6.37	4.61	5.46	9.29	6.94
4	0.20	2.96	3.02	3.79	3.81	5.17	3.72	6.55	5.80
5	0.89	9.61	6.50	6.74	10.15	8.00	3.95	4.77	8.17
6	0.96	9.78	13.48	13.76	10.18	8.04	10.15	8.30	7.44
7	1.01	8.13	9.94	8.61	8.80	9.44	9.22	9.08	6.94
8	0.90	7.38	7.67	7.59	7.88	8.76	9.37	6.45	5.08
9	0.40	3.31	3.18	6.44	3.67	5.53	3.66	7.24	5.18
10	0.98	8.32	7.49	8.49	10.63	6.98	5.51	6.45	7.70
11	0.90	11.31	11.76	11.14	10.91	7.71	7.54	6.42	6.75
12	0.99	10.15	10.98	11.28	7.53	5.41	5.12	6.30	5.23
13	0.73	9.52	12.34	8.22	7.54	7.59	6.81	5.50	6.34
14	0.49	3.15	3.26	3.86	3.00	5.50	3.57	6.73	2.99
15	0.49	3.27	5.37	3.46	3.81	4.86	6.46	6.54	7.76
16	0.25	5.77	3.93	4.12	5.04	6.57	5.68	8.80	6.61
17	0.69	3.95	5.78	6.97	3.58	7.43	7.93	8.69	5.45
18	0.86	5.96	7.75	4.78	4.00	9.04	8.93	8.92	7.18
19	0.56	6.91	7.05	8.89	6.35	7.11	6.41	6.46	7.90
20	0.94	6.86	8.59	5.27	6.22	5.18	7.60	4.68	9.98
Lysosome									
Field 1									
1	1.01	11.61	14.41	12.04	8.48	3.85	8.51	5.23	2.99
2	0.53	1.33	0.95	1.32	0.00	2.56	3.54	0.44	1.74
3	0.19	0.15	0.84	1.31	0.53	4.02	2.12	3.00	2.66
4	1.01	8.16	12.29	5.49	3.59	7.01	11.30	5.81	1.55
5	0.42	0.02	0.78	5.96	2.39	0.11	0.57	9.05	10.42
6	0.97	3.81	5.76	4.23	1.82	1.90	0.17	2.85	3.09
7	1.00	3.29	1.17	11.65	8.51	2.51	1.07	3.00	4.90
8	0.86	0.88	2.30	2.22	0.84	0.77	5.06	0.63	0.35
9	1.01	0.54	4.74	2.78	0.08	1.81	9.34	6.72	0.47
10	0.28	5.43	0.85	3.52	2.33	10.56	1.91	10.37	5.13
11	0.46	0.89	2.13	0.51	0.07	1.81	8.10	3.01	0.57
12	1.01	2.55	4.32	3.44	1.54	0.32	0.82	3.06	0.83
13	0.71	2.00	1.37	2.89	1.82	0.53	1.33	1.31	1.63
14	0.63	2.16	1.03	0.78	2.08	1.38	1.32	1.26	4.16
15	0.43	3.69	1.28	8.87	5.77	1.80	4.52	7.13	3.84
16	0.30	0.51	0.94	4.18	2.02	1.65	3.50	1.70	3.28
17	0.31	0.75	1.05	0.77	1.92	3.04	1.18	1.12	3.54
18	0.99	2.50	1.09	1.07	2.89	2.65	1.01	0.34	2.97
19	0.54	0.16	1.26	2.62	1.69	3.86	1.19	5.27	2.13
20	0.31	0.77	0.65	0.00	0.00	5.87	2.29	0.72	1.34
21	0.95	3.30	4.53	8.59	0.68	7.57	12.93	2.40	1.05
22	1.00	4.12	2.90	1.20	2.70	12.97	4.97	1.24	9.58
23	0.99	0.53	2.84	4.59	1.95	1.92	1.66	1.29	0.73

24	0.60	1.36	1.94	1.26	3.99	1.07	2.49	5.54	5.98
25	0.91	15.97	6.06	5.51	2.79	2.67	3.92	0.56	1.73
26	1.01	2.88	5.89	1.20	0.61	2.93	10.03	2.86	0.16
Field 2									
1	0.99	2.86	1.02	1.05	6.82	15.53	2.79	8.96	14.66
2	0.34	0.24	0.42	1.27	0.07	2.39	3.50	10.63	5.22
3	0.96	0.80	0.26	1.06	0.73	8.48	2.58	5.32	3.33
4	0.98	3.54	4.09	1.58	5.88	4.99	7.08	6.85	6.71
5	0.97	2.44	3.46	3.77	3.95	2.25	9.14	13.50	6.33
6	0.52	1.51	2.05	4.91	1.98	10.20	6.78	7.70	8.58
7	0.98	7.10	6.55	2.84	2.95	8.80	2.26	7.68	6.83
8	0.62	0.30	1.52	1.25	0.74	2.95	7.67	6.77	2.82
9	0.91	3.51	1.89	3.13	7.23	19.71	11.80	5.92	8.74
10	0.98	3.32	8.01	5.69	1.78	5.63	10.14	9.50	2.15
11	1.00	2.87	4.76	4.75	5.01	2.45	7.96	11.17	7.56
12	0.23	0.22	1.77	1.24	2.21	2.91	6.72	6.65	4.31
13	0.88	7.17	0.73	0.20	0.63	11.97	2.22	1.08	1.24
14	1.02	5.74	9.81	7.45	6.87	3.77	4.64	5.45	7.51
15	0.43	1.45	2.17	1.07	2.44	4.42	6.16	5.76	8.03
16	0.99	0.92	3.72	3.23	0.96	8.02	15.15	11.00	5.18
17	0.74	0.76	1.30	2.01	10.99	12.93	2.45	6.48	18.46
18	0.51	0.44	0.18	0.04	0.50	7.75	1.53	3.14	6.67
19	0.48	0.32	1.53	1.10	0.75	6.97	4.76	8.02	6.75
20	0.97	7.59	11.39	10.60	5.63	6.45	15.22	9.24	1.56
21	0.99	8.20	4.12	8.17	12.94	3.74	0.95	6.81	12.37
22	0.98	8.45	5.22	0.49	2.52	30.51	18.65	4.57	17.71
23	0.76	0.18	0.02	0.01	0.04	4.29	8.47	5.94	2.42
24	0.66	2.14	0.28	0.22	0.74	3.68	7.02	4.83	7.82
25	0.52	1.43	0.97	3.07	0.27	11.02	12.57	11.62	1.69
26	0.99	2.45	7.29	8.89	3.87	10.83	7.59	17.89	26.75
27	0.91	10.86	3.99	0.16	1.99	28.95	15.65	9.36	11.49
28	0.93	0.66	4.50	3.75	3.73	15.78	10.39	4.58	21.83
29	0.98	4.99	11.30	20.49	7.34	10.22	13.89	17.83	1.53
30	0.92	3.65	7.03	5.62	3.48	2.81	9.65	13.49	6.15
Field 3									
1	1.00	7.03	3.33	4.89	17.49	23.98	2.86	6.29	23.90
2	0.85	4.87	6.70	6.58	1.09	7.30	12.05	3.43	0.34
3	0.98	4.76	0.76	1.37	8.72	6.79	0.95	0.42	8.07
4	0.38	0.20	1.17	2.07	1.01	3.17	10.23	7.08	2.98
5	0.97	1.11	0.19	0.00	0.27	7.52	4.03	0.68	0.60
6	1.00	1.21	1.80	1.52	1.55	2.47	4.23	4.89	2.85
7	0.75	0.09	0.07	0.15	0.00	0.74	3.82	0.14	0.67
8	1.02	0.33	0.86	0.48	0.34	3.55	1.68	1.39	0.93

9	0.90	0.08	0.02	0.10	0.04	2.93	0.72	1.18	0.64
10	0.99	2.86	6.82	6.15	2.93	13.41	15.02	21.50	2.68
11	0.80	0.25	0.11	0.03	0.22	2.03	0.40	2.57	3.17
12	0.36	0.16	0.06	0.17	0.30	3.25	0.61	2.15	1.85
13	0.97	2.30	3.56	2.68	2.52	1.43	3.69	4.29	0.95
14	0.43	0.90	2.36	1.35	2.75	1.83	3.25	5.58	2.45
15	1.00	4.82	1.47	2.51	4.86	3.74	4.10	1.37	3.90
16	0.99	2.85	1.70	1.03	5.54	4.23	1.04	1.00	7.91
17	0.17	0.00	0.00	0.01	0.00	0.21	0.00	1.88	2.11
18	0.97	9.85	3.18	6.20	14.92	4.27	0.79	5.93	9.32
19	0.99	3.28	5.34	11.31	13.37	3.11	1.49	5.38	18.07
20	0.57	11.03	14.09	0.73	0.43	13.27	11.25	0.74	2.90
21	0.99	3.64	24.04	28.92	15.48	6.47	8.37	22.59	10.17
22	0.94	2.64	1.47	2.31	4.80	4.58	2.13	0.11	3.17
23	0.94	2.64	3.37	3.80	5.16	6.41	6.83	4.19	4.71
24	0.24	0.00	0.00	0.00	0.44	0.50	0.11	0.01	3.63
25	1.00	4.32	3.73	2.38	11.31	10.53	3.06	4.78	8.72
26	0.94	2.45	1.50	2.17	5.70	3.69	1.76	6.58	6.24
27	0.79	0.64	3.76	0.74	0.64	0.79	10.36	6.12	7.88
28	0.88	0.14	3.14	6.58	1.22	0.09	9.39	16.94	2.43
29	0.60	0.14	4.88	0.87	0.42	1.79	2.44	2.15	1.53
30	0.79	0.32	3.74	1.16	0.20	5.32	4.21	2.13	5.00
31	1.02	3.85	10.30	11.66	6.02	10.65	10.81	8.50	0.90
32	0.89	2.81	0.83	0.45	3.98	7.09	1.82	0.30	5.81
Field 4									
1	0.93	0.53	0.04	0.65	2.42	1.86	0.19	1.86	5.58
2	0.99	2.15	0.59	2.47	3.61	0.92	4.63	6.93	6.92
3	1.00	4.49	4.55	1.32	3.90	3.72	2.41	0.80	6.92
4	1.00	10.75	4.66	13.35	19.31	0.47	0.58	0.56	1.56
5	0.97	2.43	1.78	0.86	1.78	3.01	4.77	2.68	2.03
6	1.03	2.49	3.97	2.92	2.11	3.25	4.11	6.73	7.88
7	0.99	4.03	2.09	4.64	5.64	7.82	3.09	6.43	7.19
8	1.00	7.43	2.36	2.05	5.54	3.31	10.67	7.88	10.26
9	0.84	1.97	1.74	1.29	2.99	5.25	6.36	1.93	3.76
10	0.96	2.30	4.16	2.78	3.15	4.92	1.93	1.42	6.74
11	0.82	1.24	2.40	5.53	1.61	5.67	4.71	5.69	2.49
12	0.29	0.04	2.25	2.35	0.48	1.92	6.17	2.80	7.13
13	0.40	0.57	0.22	1.30	1.20	1.68	2.23	11.34	12.68
14	0.96	2.96	1.92	0.83	2.38	3.47	2.88	1.17	0.82
15	1.02	0.79	1.35	2.26	1.07	1.63	0.59	0.75	2.67
16	0.40	2.85	4.10	1.18	0.43	4.11	2.10	2.85	2.43
17	1.00	0.99	1.01	0.18	0.40	5.88	2.51	2.77	4.23
18	0.96	0.58	2.66	5.94	5.30	0.22	2.18	7.62	6.99

19	0.49	1.71	0.85	1.50	1.20	2.42	3.19	6.62	3.63
20	0.94	1.42	0.67	0.82	1.39	4.32	9.45	10.76	6.04
21	0.56	1.90	0.84	0.30	0.11	3.52	4.54	2.21	1.67
22	0.99	7.61	7.28	10.84	0.50	9.50	4.07	0.39	1.72
23	0.97	0.25	0.50	0.15	0.04	3.92	10.55	3.84	0.57
24	0.77	0.08	0.28	0.35	0.28	3.55	4.60	6.71	2.57
25	0.94	0.00	0.00	0.00	0.01	1.59	3.73	1.97	1.56
26	0.98	3.01	3.01	4.90	5.60	0.89	0.33	1.76	3.08
27	1.00	1.77	4.20	2.16	0.78	5.38	5.96	3.35	5.59
28	0.98	1.20	0.68	2.19	5.02	1.45	3.53	15.85	21.51
29	0.97	4.04	2.35	2.12	5.67	4.78	1.76	3.01	5.71
30	0.97	4.71	7.08	3.95	1.46	7.52	13.25	6.31	2.47
31	0.97	0.09	0.82	4.05	0.49	8.51	8.85	4.22	3.64
32	1.02	0.66	1.71	0.57	1.16	7.27	5.04	4.66	6.47
33	1.01	2.53	1.74	1.43	1.72	3.07	2.60	8.24	9.58
34	0.87	0.00	0.00	0.00	0.00	0.84	4.49	5.84	0.20
Actin									
Field 1									
1	0.44	6.64	10.94	6.98	10.14	14.99	16.93	18.36	15.12
2	0.65	1.23	1.74	1.72	1.66	12.84	14.54	16.27	15.46
3	0.52	2.27	2.29	1.41	3.44	12.94	15.88	12.41	13.51
4	0.69	1.87	1.19	8.41	5.28	13.94	19.02	13.61	16.81
5	0.91	12.67	10.40	28.20	21.58	18.60	15.56	28.44	35.71
6	0.97	18.81	11.26	11.25	17.16	17.74	17.55	16.95	14.01
7	0.95	8.20	10.10	7.54	7.92	14.97	13.70	16.16	16.28
8	0.97	3.09	4.09	4.80	4.97	10.09	9.77	10.35	16.94
9	0.95	13.04	11.00	16.04	13.32	18.39	18.78	31.74	26.07
10	0.94	5.20	6.87	16.37	7.09	12.88	22.12	21.53	14.37
11	0.93	18.13	20.92	14.17	12.50	20.79	24.47	15.54	14.66
12	0.98	17.72	19.76	13.91	19.18	24.75	19.38	17.99	20.89
13	0.98	14.32	15.63	9.96	12.27	13.04	19.88	18.34	20.05
14	0.96	13.03	10.63	8.61	11.89	16.22	19.80	18.84	18.47
15	0.90	10.48	14.14	7.58	7.83	12.60	20.47	12.57	10.34
16	0.96	15.03	16.09	14.44	7.37	8.88	9.42	19.44	14.34
17	0.94	22.59	10.04	18.02	19.54	17.09	17.54	10.21	12.88
18	0.91	9.19	13.96	22.28	12.00	13.33	10.11	17.97	12.25
19	1.00	26.05	15.37	11.90	21.05	17.99	13.67	14.52	17.89
20	0.96	20.89	21.11	14.41	12.86	16.30	21.12	13.13	10.86
21	0.94	10.29	9.45	10.34	17.48	16.15	12.85	10.94	13.18
22	1.01	7.62	19.05	15.91	11.38	11.28	20.74	11.64	12.59
23	0.87	6.03	7.13	3.73	5.74	14.20	13.72	16.76	14.66
24	0.94	8.11	8.77	13.21	10.90	15.52	16.01	28.62	22.78
25	0.94	14.20	11.32	7.41	8.52	31.20	25.51	13.29	17.36

26	0.69	3.25	6.82	2.88	2.78	11.53	12.39	10.44	20.51
Field 2									
1	0.94	7.82	6.49	10.31	17.61	27.21	26.64	30.44	34.58
2	0.95	10.50	15.54	10.72	7.96	38.98	38.58	25.73	34.55
3	0.39	5.00	12.11	6.62	4.20	22.59	37.45	31.37	19.57
4	0.33	10.64	16.86	3.59	18.50	33.05	33.42	20.11	44.48
5	0.18	4.23	0.86	0.73	2.16	14.55	5.10	5.13	12.08
6	0.98	26.47	20.42	21.19	26.89	20.90	16.28	14.12	21.83
7	0.94	31.08	27.62	17.98	21.73	28.81	21.51	17.34	23.76
8	0.87	8.83	18.48	28.24	5.42	16.36	22.43	20.59	13.75
9	0.98	7.15	10.83	13.79	10.77	11.59	12.68	21.24	17.47
10	0.92	22.31	14.99	22.47	13.24	20.96	20.31	24.65	14.24
11	0.98	22.18	14.83	18.32	19.28	23.02	15.22	19.84	12.79
12	0.98	21.79	26.42	22.09	19.04	20.40	27.93	18.40	16.37
13	0.94	12.12	19.09	18.62	3.54	25.15	18.06	20.41	10.07
14	1.00	8.84	5.00	21.25	10.64	31.18	20.00	25.35	23.89
15	0.24	1.59	5.78	5.14	1.38	9.44	12.75	24.90	4.26
16	0.50	2.84	5.14	2.34	2.75	16.84	15.49	17.32	16.66
17	0.93	6.15	7.64	4.66	3.57	27.39	33.64	30.33	17.99
18	0.79	1.70	2.43	3.81	2.69	14.15	19.12	15.25	14.88
Field 3									
1	0.91	7.20	8.49	6.74	9.14	25.81	32.57	19.54	19.54
2	0.96	11.21	14.82	11.06	10.08	22.98	27.51	20.94	25.02
3	0.95	15.97	10.34	10.26	13.84	32.69	21.65	18.73	29.67
4	0.76	7.53	12.98	10.57	15.01	17.12	13.12	28.68	22.83
5	1.01	26.75	12.05	20.67	17.20	15.72	21.95	28.41	21.26
6	0.41	14.79	11.61	15.26	7.17	15.89	18.01	22.04	16.48
7	0.81	10.68	15.32	16.08	11.46	21.51	19.54	25.10	22.21
8	0.96	13.03	10.69	13.28	16.20	25.50	16.97	18.58	19.91
9	0.98	16.57	11.16	11.48	20.02	18.94	16.17	19.07	21.57
10	0.99	19.99	18.16	19.45	32.23	37.56	24.36	18.08	33.66
11	0.44	7.90	8.95	5.69	10.28	16.21	17.51	19.96	16.62
12	0.97	7.57	13.82	19.45	12.80	19.27	17.32	20.33	18.65
13	0.92	9.59	8.24	4.64	6.76	19.97	22.09	18.15	18.21
14	0.97	18.98	18.39	14.86	13.41	20.09	25.52	22.87	13.84
15	0.93	14.28	15.25	12.49	11.96	19.99	24.28	16.49	14.50
16	0.65	20.45	15.43	11.13	7.69	20.94	12.68	18.21	14.96
17	0.90	13.55	8.64	9.40	17.72	16.65	23.55	27.22	18.11
18	0.96	28.35	36.23	38.63	36.28	43.06	64.93	57.57	46.35
19	1.00	27.08	11.02	8.68	26.78	33.08	16.92	9.44	32.96
20	0.94	15.83	13.36	10.53	8.39	20.08	18.92	22.51	19.60
21	0.98	14.26	11.72	17.92	25.49	14.50	23.50	22.08	28.14
22	1.01	16.91	9.10	8.24	11.64	20.28	19.26	25.60	18.48

23	0.95	19.73	12.73	18.18	12.93	31.34	23.64	24.54	12.45
24	0.85	12.10	10.46	22.60	13.71	17.21	25.51	40.18	23.27
25	0.94	7.93	4.91	13.06	10.05	17.44	14.84	23.95	20.78
Field 4									
1	0.97	15.10	11.17	14.65	18.33	20.17	16.33	19.24	28.53
2	0.98	20.28	20.80	14.39	18.87	27.55	23.38	16.54	15.42
3	0.97	15.04	13.82	15.54	13.91	23.38	17.13	18.32	13.91
4	0.99	15.97	17.98	23.68	17.65	23.96	31.04	18.60	11.77
5	0.97	26.07	16.23	17.26	26.45	17.20	14.70	10.87	14.26
6	0.83	19.05	19.05	17.06	13.14	18.39	19.52	17.46	18.87
7	0.97	23.93	20.60	23.47	32.07	17.20	15.73	23.00	21.12
8	0.98	21.67	25.66	21.79	23.07	19.07	19.96	21.31	19.92
9	0.92	22.29	17.54	18.62	24.00	19.75	18.70	15.25	17.31
10	0.30	11.42	12.36	15.69	20.07	18.00	20.78	21.97	24.46
11	0.87	17.78	23.71	24.63	19.56	15.79	22.60	19.61	18.10
12	0.89	29.18	36.03	17.70	15.68	25.29	28.88	22.10	19.56
13	0.93	15.32	13.38	31.10	32.51	16.15	13.98	17.63	22.56
14	1.00	17.85	18.82	18.44	25.66	15.85	24.65	16.84	16.41
15	0.81	16.65	17.39	15.74	15.93	18.31	14.03	26.63	14.12
16	0.97	8.81	12.90	10.03	13.86	17.84	31.43	17.27	17.78
17	1.01	20.01	16.44	17.78	21.05	17.00	21.32	22.33	19.29
18	0.96	11.95	14.51	13.62	12.37	17.91	19.16	23.46	17.85
19	0.92	10.87	15.40	18.95	13.54	9.19	12.01	14.63	21.69
20	0.53	16.46	7.35	21.63	19.02	20.11	13.94	24.90	15.66
21	0.92	23.12	30.28	35.24	23.13	25.19	34.21	28.62	21.64
22	0.96	41.51	26.65	21.44	25.37	34.66	18.15	19.93	19.53
23	1.01	18.11	17.31	24.60	15.87	19.78	12.94	19.00	16.29
24	0.96	20.49	20.23	26.57	19.09	26.16	21.78	20.48	18.58
25	1.00	25.44	15.81	14.31	16.54	20.15	14.67	12.80	13.12
26	0.92	12.53	20.77	20.91	16.21	13.07	19.35	20.50	12.73
27	1.00	12.77	12.45	16.48	11.83	20.31	25.13	25.08	24.31
28	1.01	3.21	1.97	1.93	5.85	4.05	2.26	4.41	6.47
29	0.62	14.68	14.54	18.81	11.29	13.24	15.54	18.62	22.11
30	0.98	8.01	5.24	2.28	2.95	9.00	6.25	2.81	3.61
31	1.01	22.27	24.39	23.75	24.97	20.41	11.63	17.68	22.08
32	0.61	12.13	19.72	21.66	22.99	21.85	19.49	17.41	17.64
33	0.99	17.38	15.35	15.95	19.28	14.51	13.95	15.51	15.94
34	1.00	15.45	14.98	26.55	13.64	14.77	9.58	16.23	12.48
35	0.97	25.02	15.60	15.94	22.30	15.50	14.09	12.48	14.61
36	0.77	13.25	15.56	21.16	12.10	7.59	10.28	25.02	8.98
Tubulin									
Field 1									
1	0.72	0.58	0.70	1.02	0.69	0.97	1.49	1.44	1.27

2	0.75	1.22	1.77	0.71	0.80	1.63	3.11	1.23	0.93
3	0.88	0.97	3.58	2.43	1.14	1.83	1.27	2.61	1.24
4	0.92	3.26	4.73	3.16	2.81	1.90	2.77	3.73	4.64
5	0.40	0.71	0.52	0.75	1.76	1.40	1.32	1.27	1.60
6	1.01	1.12	1.48	1.01	0.69	2.07	1.57	1.32	0.89
7	0.99	1.01	0.64	0.77	1.51	1.34	1.03	0.95	1.21
8	0.98	0.82	1.26	1.08	0.58	1.60	1.76	1.82	0.90
9	0.70	0.55	0.73	0.62	0.59	1.04	1.73	1.36	1.21
10	0.81	0.84	0.91	1.18	1.12	0.91	1.80	1.94	1.75
11	0.53	0.63	0.81	0.61	0.65	1.53	1.32	1.45	1.52
12	0.66	0.67	0.55	0.54	0.58	1.73	1.15	0.96	0.96
13	0.56	0.62	0.55	0.67	0.73	1.08	1.00	1.89	2.11
14	0.27	3.49	0.55	0.52	0.52	2.53	1.59	1.37	1.27
15	0.41	0.79	0.57	0.54	0.60	1.32	0.88	1.08	1.05
16	0.43	0.63	0.78	0.54	0.64	1.40	1.18	1.34	0.87
17	1.00	1.66	0.87	1.17	1.13	1.43	1.47	1.00	0.91
18	0.24	0.99	0.51	0.68	0.71	1.43	0.91	1.08	1.11
19	0.18	2.37	0.76	0.54	1.52	1.09	1.24	1.08	1.24
20	0.27	0.46	0.51	0.59	0.60	0.85	1.03	1.04	1.10
21	0.88	0.83	0.97	0.84	1.21	1.10	1.00	1.05	1.08
Field 2									
1	0.96	2.88	3.44	3.16	5.08	1.96	2.02	1.95	2.33
2	0.99	3.85	5.43	2.85	1.61	1.68	2.32	1.99	1.23
3	0.97	11.97	14.43	15.49	18.92	5.03	5.14	6.71	4.43
4	1.02	2.90	2.35	1.98	3.70	1.66	1.20	1.16	1.85
5	0.28	4.29	1.77	2.80	1.39	4.69	1.12	1.66	1.26
6	0.92	1.50	3.38	3.67	1.59	1.46	1.86	1.66	1.32
7	0.92	1.47	6.34	1.79	1.87	1.47	3.24	1.95	1.88
8	0.93	5.63	1.86	2.28	2.56	4.96	1.28	1.64	2.00
9	0.98	2.05	2.29	1.26	1.13	1.86	1.82	1.31	1.35
10	0.65	1.88	2.48	2.17	1.78	1.03	1.22	1.40	1.07
11	0.96	4.58	3.26	2.91	2.95	1.43	1.33	1.62	1.28
12	0.79	2.69	3.94	6.57	3.82	1.87	1.71	2.03	2.49
13	0.90	4.44	4.59	3.34	4.10	2.07	2.31	1.93	1.74
14	0.93	2.75	3.28	2.91	1.99	1.74	1.83	2.16	1.62
15	0.82	18.36	18.26	8.49	15.36	7.06	11.96	13.77	7.14
16	0.88	3.53	3.37	4.33	3.99	1.90	1.13	1.86	1.51
17	0.96	3.13	3.50	10.05	2.67	1.78	2.05	2.53	1.48
18	0.72	6.56	2.56	2.31	2.72	1.97	1.18	1.24	2.58
19	1.01	3.42	3.50	23.23	18.18	1.53	2.64	14.44	4.52
20	0.99	6.12	3.18	2.66	4.36	2.38	1.38	1.57	2.78
21	1.00	20.84	89.69	39.52	28.84	21.39	59.52	26.43	17.49
22	0.97	9.87	6.73	6.46	6.92	7.78	4.71	6.83	5.49

23	0.98	1.60	2.79	4.75	2.90	1.41	2.37	3.13	1.64
24	0.93	5.45	3.78	2.31	3.88	2.38	2.68	0.94	1.42
Field 3									
1	0.96	2.48	2.06	2.04	2.02	2.41	1.99	1.53	4.05
2	0.99	2.58	1.71	2.85	2.31	1.69	1.28	2.05	1.77
3	0.92	2.49	2.67	1.99	2.78	1.51	1.55	1.40	1.36
4	0.89	5.89	2.11	5.18	3.82	1.07	1.67	2.61	2.70
5	0.40	2.06	2.09	2.33	4.13	2.10	2.16	1.79	2.78
6	0.97	1.89	2.11	2.50	2.11	1.50	1.57	1.98	1.40
7	0.81	5.26	2.02	3.36	2.48	2.33	1.31	2.17	2.27
8	0.87	2.23	2.46	2.12	2.34	1.32	1.45	1.45	1.48
9	0.97	3.33	2.61	2.64	5.50	1.77	1.28	1.70	2.06
10	0.97	2.85	2.50	3.55	2.33	1.64	1.46	3.33	1.66
11	0.94	2.54	2.00	2.12	2.26	2.86	1.14	1.47	1.96
12	1.00	1.36	1.80	3.02	1.66	1.07	1.17	2.06	1.67
13	1.00	1.72	2.43	1.57	1.07	1.44	1.86	1.23	0.86
14	0.42	4.27	2.25	2.09	2.73	7.08	2.08	1.33	4.52
15	1.00	2.97	4.57	3.94	2.20	1.11	1.10	1.25	1.27
16	0.85	3.04	2.24	1.86	1.71	2.00	1.14	1.77	2.07
17	0.98	2.41	2.11	2.89	2.97	2.16	1.71	2.67	2.28
18	0.99	2.81	1.58	2.87	4.96	1.49	1.23	1.20	2.58
Field 4									
1	0.26	0.12	0.66	0.59	0.12	0.13	4.48	2.94	0.13
2	0.95	6.53	3.22	11.37	19.50	26.43	17.82	43.48	60.86
3	0.84	0.90	1.24	2.57	17.22	1.94	5.19	3.91	14.34
4	0.95	2.70	3.50	5.80	1.88	6.23	3.66	5.32	5.73
5	0.57	0.25	0.19	1.03	0.33	1.04	0.57	10.06	2.52
6	1.01	7.15	5.70	0.65	5.30	7.96	5.09	1.52	2.73
7	0.97	0.78	0.20	0.28	1.82	1.84	1.13	1.03	2.32
8	0.91	0.44	1.64	1.36	0.54	1.26	2.39	3.42	1.55
9	0.96	0.86	0.88	0.88	0.67	0.85	1.20	1.09	0.57
10	0.83	1.40	1.33	1.52	0.55	0.42	1.06	1.49	0.54
11	1.01	1.53	4.62	13.44	2.96	0.80	3.99	5.35	1.69
12	0.96	7.00	3.80	1.35	2.33	4.05	2.43	1.48	1.99
13	0.93	1.48	4.30	1.20	2.53	1.86	3.65	1.80	2.24
14	0.63	0.62	1.75	1.86	1.70	1.02	1.27	1.45	1.75
15	0.92	0.71	1.12	0.62	0.96	1.80	5.13	2.19	1.71
16	0.86	19.80	1.02	2.03	2.48	11.02	2.79	1.66	3.97
17	0.95	0.19	0.30	0.47	0.38	1.38	1.32	5.42	6.36
18	1.00	13.43	0.27	0.15	0.26	4.86	1.24	0.47	1.00
19	0.29	0.97	2.34	16.98	0.28	10.75	3.53	11.05	2.42
20	0.27	0.13	0.15	0.72	0.16	0.37	0.65	4.73	0.85
Field 5									

1	0.96	1.66	1.13	0.72	0.45	0.66	1.92	1.81	0.52
2	0.98	1.17	1.23	1.48	4.44	1.02	1.17	0.63	2.08
3	1.01	2.08	2.45	0.98	1.76	1.83	0.71	1.14	1.00
4	0.87	1.29	0.55	1.15	0.84	1.29	1.29	1.40	0.79
5	0.82	0.23	0.34	0.45	0.36	0.26	1.95	2.19	0.60
6	0.67	0.60	0.90	3.49	7.22	0.77	0.78	7.93	10.76
7	0.67	0.58	0.34	0.32	0.24	1.59	0.56	0.79	0.90
8	0.91	0.48	0.91	0.70	0.44	0.61	1.00	1.73	0.36
9	0.98	0.59	1.40	5.84	0.69	0.26	0.64	0.92	0.55
10	0.97	6.53	1.10	1.66	1.38	0.97	2.30	1.17	1.01
11	0.44	0.98	1.67	1.12	0.97	1.03	0.96	0.82	0.93
12	0.83	9.10	6.06	11.34	10.89	9.18	6.27	10.65	11.26
13	0.84	12.30	5.18	7.59	5.11	13.63	4.61	2.75	5.68
14	0.84	1.76	1.45	1.96	1.06	2.02	1.28	5.73	1.12
15	0.90	7.09	8.74	2.58	1.93	3.11	8.63	2.04	1.20
16	0.94	3.26	1.27	1.75	2.94	5.46	1.65	1.73	2.87
17	0.95	3.95	2.06	9.27	7.38	3.21	2.02	5.38	3.73
18	0.98	1.16	0.95	1.21	1.30	1.69	1.27	1.47	1.16

Table S9: Raw APVs of DIC images corresponding to fluorescent images of organelles in isotonically treated RAW264.7 cell population

	Octant 1	*Octant 2*	*Octant 3*	*Octant 4*	*Octant 5*	*Octant 6*	*Octant 7*	*Octant 8*
Field 1-Nucleus/Mitochondria								
1	73.32	74.83	71.62	71.80	71.60	73.34	70.35	69.74
2	70.84	70.72	69.11	69.08	70.63	70.74	68.99	68.59
3	67.79	68.81	71.68	69.95	70.10	67.78	68.08	69.05
4	69.15	69.70	71.39	67.60	69.09	69.92	69.22	66.34
5	67.44	68.13	69.67	69.88	67.65	68.85	69.66	69.21
6	68.67	68.49	71.95	68.48	68.50	70.35	71.01	67.71
7	70.21	72.52	71.42	69.65	69.87	73.11	71.51	70.44
8	70.04	70.39	72.05	68.06	70.40	71.84	71.23	67.19
9	66.54	67.54	67.48	64.93	65.75	66.66	67.73	66.49
10	69.68	71.93	70.05	69.55	70.88	71.40	70.68	68.54
11	71.00	73.20	71.58	72.87	69.28	71.22	73.36	72.38
12	70.07	67.14	69.09	70.20	70.08	67.85	68.88	70.68
13	69.71	65.90	66.91	69.11	67.48	65.12	65.23	66.10
14	75.43	74.05	73.40	75.56	75.05	73.49	72.53	74.13
15	73.00	73.32	72.31	74.71	73.47	72.99	72.14	75.06
16	77.29	73.29	73.16	74.61	74.55	73.51	72.51	74.29
17	71.74	74.84	75.71	70.92	71.51	72.92	73.55	72.52
18	72.64	72.89	74.50	71.23	73.20	73.62	72.96	71.54

19	71.54	71.53	69.27	70.26	71.79	71.43	69.01	70.65
Field 2								
1	76.77	74.74	75.01	76.82	76.44	75.07	76.07	76.01
2	78.60	77.98	75.16	76.86	76.07	76.25	74.96	74.60
3	73.93	75.94	71.31	72.79	71.33	73.58	71.81	70.27
4	74.76	74.33	74.75	71.91	70.79	72.82	71.59	73.13
5	72.61	73.37	74.73	74.61	75.60	72.74	74.01	74.40
6	74.44	75.15	75.74	73.57	72.25	75.77	73.44	69.63
7	73.55	73.57	73.98	73.41	74.34	74.51	71.74	71.19
8	75.15	77.82	74.01	72.60	69.64	71.32	72.63	72.51
9	75.65	76.68	75.97	73.77	74.78	78.10	76.44	73.63
10	77.80	78.31	77.44	78.55	77.13	76.06	72.35	74.79
11	79.75	76.46	78.79	78.41	77.85	77.16	75.76	75.19
12	73.47	75.79	76.13	74.78	74.29	75.15	75.22	76.38
13	74.60	75.01	75.29	71.49	70.32	72.10	75.75	72.54
14	75.78	75.95	78.43	76.05	71.88	73.49	74.29	76.05
15	77.71	75.15	77.14	77.73	71.46	75.47	73.46	73.14
16	73.91	73.73	74.22	74.80	73.55	69.80	72.04	72.04
17	71.87	71.29	71.54	71.75	71.60	70.69	69.05	69.40
18	74.26	72.36	74.78	74.03	74.14	72.72	71.50	73.17
19	76.72	74.75	74.77	76.02	73.28	70.02	75.55	75.17
Field 3								
1	77.47	73.87	71.17	73.84	72.63	68.25	66.96	68.13
2	79.08	75.09	73.62	78.38	75.33	75.02	64.73	69.37
3	73.20	72.30	71.78	71.09	71.83	71.63	68.95	68.59
4	71.20	68.86	67.86	68.58	69.86	66.38	65.85	69.79
5	73.64	74.87	74.46	72.09	63.73	67.68	66.24	69.14
6	73.42	76.56	77.72	73.63	72.42	75.80	68.29	68.34
7	76.37	76.42	74.90	74.64	73.40	76.61	73.97	72.90
8	76.75	75.58	75.36	73.05	68.66	71.11	70.12	63.37
9	73.54	78.71	75.53	73.26	65.60	69.03	67.89	62.05
10	75.87	76.13	75.11	73.17	68.43	70.24	65.76	70.13
11	71.85	74.97	73.67	73.30	69.64	69.07	65.99	68.70
12	72.02	75.36	77.05	73.52	68.44	70.82	71.77	72.57
13	66.50	69.48	70.85	68.27	60.37	68.17	66.44	63.18
14	66.82	68.99	71.70	68.42	64.57	61.47	64.15	66.48
15	69.02	72.24	74.80	70.14	62.24	64.12	67.00	70.26
16	63.94	66.40	67.11	66.07	61.64	63.18	61.20	64.62
17	72.59	73.84	73.58	73.58	71.43	73.23	73.90	71.86
18	75.43	78.51	77.61	76.37	70.11	71.02	75.98	71.97
19	71.24	74.76	73.64	74.19	69.20	73.78	75.56	68.88
20	74.05	76.46	77.97	75.12	74.47	70.47	69.98	63.29
21	76.36	73.93	74.73	75.04	67.59	66.24	68.20	65.41

22	77.35	76.23	78.01	77.61	76.03	74.77	72.40	72.76
23	76.60	75.06	76.10	79.13	69.91	75.92	69.91	72.08
24	74.74	75.67	77.48	76.30	74.88	69.74	76.45	71.28
25	77.36	75.05	77.32	77.69	71.96	71.83	72.03	72.73
26	75.31	81.00	77.31	73.42	71.10	71.30	70.18	64.55
27	78.05	78.63	78.01	79.22	77.37	76.12	76.87	77.69
28	74.31	73.07	75.06	73.74	67.20	65.49	69.87	67.73
29	69.78	71.47	72.95	69.20	59.74	59.43	64.19	59.60
30	74.98	71.78	74.45	74.08	67.95	69.16	66.80	67.59
31	74.79	73.18	74.87	77.72	72.29	72.56	69.80	73.61
32	73.34	71.94	71.63	74.40	72.76	73.53	70.81	72.24
33	72.32	71.92	73.40	74.55	65.46	69.45	68.59	65.54
34	74.84	71.95	73.13	74.92	72.52	68.56	70.82	71.41
35	76.01	74.98	74.34	76.73	72.97	70.40	71.63	75.00
36	75.52	76.39	72.49	74.37	72.20	71.66	74.24	75.13
37	72.55	72.26	72.02	72.43	70.56	67.49	69.15	70.82
38	72.99	74.02	75.65	76.12	70.33	69.85	64.88	68.96
39	79.75	76.79	76.06	76.70	70.72	70.20	67.68	71.96
40	75.91	76.53	75.61	75.65	73.62	75.85	73.21	69.16
41	76.56	80.00	77.35	80.71	78.11	76.96	75.22	68.98
42	82.33	77.09	75.99	77.81	76.19	78.48	75.06	72.28
43	80.12	79.16	76.18	77.55	75.84	76.82	72.07	77.24
44	79.56	79.33	77.39	78.24	75.04	72.57	66.70	71.06
45	79.56	80.02	78.88	81.95	73.54	76.28	74.26	73.56
46	75.91	80.01	77.38	78.70	72.07	76.70	67.70	72.17
Field 4								
1	75.33	74.74	73.23	73.96	66.12	69.50	68.60	63.77
2	74.15	71.74	71.91	74.42	70.87	69.84	69.72	72.48
3	78.48	75.27	74.02	77.73	78.51	74.47	66.32	76.40
4	80.58	75.21	78.10	76.51	76.56	74.66	75.17	77.04
5	79.89	78.11	75.76	75.71	77.30	78.28	74.97	74.43
6	77.21	77.59	77.93	78.80	77.48	77.72	76.64	76.27
7	77.40	75.92	77.47	75.88	73.79	74.33	71.31	66.89
8	78.76	78.45	75.91	78.42	72.05	74.47	72.73	70.25
9	78.36	75.93	74.62	76.57	74.39	71.43	68.25	73.31
10	75.80	76.76	75.90	75.26	66.07	75.22	72.42	64.04
11	79.29	74.74	74.10	76.06	77.59	82.07	75.36	77.11
12	79.79	79.74	75.70	75.44	61.37	71.49	72.52	68.79
13	77.00	76.50	76.00	76.24	76.92	74.47	74.16	76.72
14	78.44	78.21	78.08	78.84	75.64	81.45	76.09	76.80
15	79.43	74.76	76.15	76.99	76.86	74.95	67.69	69.12
16	78.79	77.65	71.87	78.08	69.14	72.57	69.06	65.68
17	79.17	75.91	73.36	77.27	71.74	73.54	68.12	68.46

18	70.25	72.37	72.51	70.01	68.91	71.11	68.29	64.88
19	73.59	76.85	75.99	75.28	74.45	73.35	72.52	73.70
20	70.69	74.12	73.91	70.61	69.63	72.31	70.82	68.96
21	70.99	75.08	71.66	72.27	67.64	70.98	70.70	66.55
22	71.13	72.44	73.61	71.95	69.48	72.64	71.12	67.57
23	71.24	72.91	71.54	70.91	66.69	69.92	70.96	69.51
24	71.25	71.88	74.44	71.46	70.68	73.68	70.93	68.94
25	71.91	75.29	74.24	71.90	64.93	65.70	69.18	69.27
26	73.74	74.00	77.03	74.01	71.84	72.83	72.20	64.61
27	66.21	67.44	69.70	66.32	56.33	61.64	56.67	56.92
28	74.12	72.81	71.67	73.52	67.06	64.87	58.79	60.86
29	64.20	66.28	66.77	63.38	58.90	62.04	58.88	56.92
30	74.43	75.15	77.83	76.79	67.88	67.47	69.04	67.81
31	73.22	74.49	76.45	74.73	73.55	67.80	68.16	64.92
32	74.46	72.93	74.37	74.15	70.01	71.04	69.92	67.69
33	74.13	71.41	72.54	72.36	69.30	66.24	64.57	69.34
34	69.97	72.92	74.85	73.12	71.69	66.16	65.28	65.82
35	69.04	72.23	72.48	71.99	66.90	67.96	69.31	66.90
36	68.97	71.09	72.28	69.35	62.14	63.28	64.10	60.73
37	69.04	69.27	71.98	77.28	63.04	64.56	69.14	68.97
Field 1-Cell Membrane/Endoplasmic Reticulum								
1	132.11	121.60	120.91	123.24	124.63	121.16	115.66	122.11
2	133.97	125.77	128.26	130.56	123.90	120.35	120.99	118.40
3	130.90	127.77	121.80	127.87	126.04	124.30	116.42	122.60
4	128.04	130.59	122.24	131.82	129.24	126.68	121.15	130.09
5	138.06	129.98	129.97	130.94	128.20	130.40	127.14	124.27
6	137.63	143.22	132.96	139.51	132.47	133.12	128.93	131.93
7	139.38	139.32	140.76	136.25	136.11	137.39	137.40	134.22
8	131.07	136.33	140.36	133.22	128.44	133.81	134.21	126.44
9	139.18	136.00	136.14	137.49	136.02	133.64	131.66	132.84
10	133.46	136.04	128.04	140.25	135.11	131.65	129.12	132.36
11	136.66	133.35	121.59	134.05	130.45	121.05	125.52	133.64
12	125.25	123.92	121.61	123.58	122.05	119.40	118.24	121.08
13	131.43	124.70	123.60	122.03	118.89	121.86	120.08	118.97
14	116.94	122.02	122.57	116.61	114.20	118.46	116.71	110.02
15	119.99	128.31	124.30	118.50	122.77	122.59	124.49	117.55
16	132.49	127.43	138.54	134.95	128.37	131.09	132.10	129.89
17	119.27	121.69	123.71	120.27	116.80	122.23	122.26	117.44
18	122.43	125.22	126.03	122.88	118.78	120.57	119.92	113.38
Field 2								
1	131.03	141.20	136.04	131.64	131.10	134.03	128.54	129.30
2	138.91	134.68	140.78	135.46	133.10	134.19	132.38	126.08
3	136.78	136.60	134.26	134.80	134.36	133.19	131.04	133.33

4	144.18	149.13	138.33	139.82	137.89	140.50	138.12	138.39
5	136.22	135.56	135.09	138.84	136.00	131.57	130.92	135.57
6	141.67	139.10	135.19	123.59	138.77	135.37	129.97	134.11
7	128.53	136.21	135.21	136.61	133.36	128.94	132.19	129.35
8	130.20	124.80	124.70	127.95	127.32	122.78	121.20	123.22
9	125.35	126.02	130.54	128.45	125.95	121.59	126.07	128.21
10	123.37	120.74	123.82	125.14	123.65	118.96	116.77	122.04
11	135.23	130.88	120.24	123.25	128.85	122.08	120.54	122.10
12	131.81	124.80	127.79	132.91	128.84	122.42	123.55	130.75
13	123.98	132.76	126.26	125.35	123.92	124.55	122.17	121.69
14	121.08	117.53	104.76	119.38	119.73	117.77	104.02	115.69
15	139.51	135.22	128.32	134.69	136.74	127.29	124.42	130.54
16	129.32	123.11	117.75	127.02	127.63	118.08	113.04	123.32
17	128.41	134.43	133.89	128.22	122.83	128.75	127.77	120.77
18	127.35	129.27	132.37	120.37	123.02	129.22	124.58	120.35
19	134.40	136.00	139.55	134.78	132.32	132.66	134.62	133.14
20	135.01	138.79	137.37	138.05	128.83	134.91	131.84	127.73
21	130.16	135.92	133.38	132.89	125.84	130.68	131.56	129.53
22	133.60	134.75	133.40	129.95	127.97	136.01	137.06	129.15
23	134.07	140.43	133.28	136.51	133.90	131.78	131.79	129.91
24	140.45	139.47	135.24	142.37	139.36	135.70	137.25	139.38
25	137.26	137.48	131.43	134.22	130.66	121.85	125.90	130.24
26	140.95	141.93	140.53	141.72	134.00	136.44	137.87	134.98
27	126.99	127.31	127.78	141.89	130.28	126.73	126.14	130.85
28	129.99	130.09	129.88	132.84	127.49	128.32	129.86	129.34
29	124.65	125.26	126.86	124.87	121.87	123.12	124.68	124.27
30	124.84	120.46	121.05	122.04	123.56	116.20	118.02	124.72
Field 3								
1	124.34	141.34	138.06	133.40	132.90	138.63	138.90	133.00
2	146.17	147.18	142.72	138.39	142.12	150.39	139.64	138.16
3	142.71	146.06	142.38	142.82	144.43	147.90	137.18	141.79
4	146.33	151.66	150.12	142.64	150.87	146.24	143.03	144.36
5	147.33	152.64	142.71	143.59	150.65	148.31	143.28	146.29
6	152.97	154.70	143.13	147.26	143.81	148.65	147.87	145.24
7	148.69	146.83	141.00	144.05	149.29	148.25	150.98	147.67
8	147.27	145.60	143.12	147.33	145.70	143.80	138.92	140.88
9	150.13	160.86	143.64	144.38	149.13	144.50	140.93	141.32
10	158.52	151.53	137.34	139.41	145.72	136.77	136.73	141.86
11	142.24	139.27	134.47	146.63	142.85	138.60	129.30	146.61
12	146.66	148.17	149.03	147.95	147.62	146.66	148.16	149.00
13	154.65	150.27	146.78	148.88	147.75	144.82	146.17	147.25
14	146.87	155.09	146.27	152.17	153.30	146.00	147.57	147.21
15	146.95	148.57	146.95	136.94	134.00	136.09	135.82	136.86

16	143.23	139.77	138.03	142.44	143.65	143.50	143.04	140.38
17	134.54	147.10	147.60	142.49	142.08	145.29	145.30	142.65
18	146.54	152.45	151.36	145.69	142.26	149.32	150.09	146.04
19	141.93	140.14	146.85	142.58	138.45	143.58	144.03	139.39
20	140.78	142.17	147.80	139.35	147.31	146.53	143.96	142.87
21	133.80	142.88	137.85	135.40	128.63	140.81	144.77	135.63
22	136.63	139.24	137.82	137.82	132.77	141.08	143.07	133.41
23	128.08	135.32	137.37	128.84	127.78	134.58	136.06	129.58
24	149.96	145.19	149.49	147.12	151.33	142.78	146.80	144.88
25	147.57	147.54	138.90	147.03	141.06	137.99	140.93	141.27
26	150.85	147.05	143.83	149.10	150.49	144.76	139.88	146.74
27	121.88	135.15	132.10	140.15	139.13	131.89	130.56	140.14
28	133.60	129.01	122.97	136.89	132.27	124.94	124.59	133.63
29	127.46	122.26	140.12	131.39	120.18	111.54	121.83	127.58
30	148.18	138.19	137.98	139.88	144.74	135.14	140.05	140.14
31	142.72	138.74	134.84	147.19	142.83	136.98	131.54	138.46
Field 4								
1	149.14	160.82	156.31	143.38	138.62	142.39	152.23	128.32
2	135.36	141.82	144.01	139.41	133.72	134.25	132.33	121.69
3	136.85	148.55	146.51	139.60	134.26	138.09	138.91	134.23
4	143.21	148.22	146.73	138.33	137.02	146.83	142.92	138.54
5	147.13	153.73	140.18	147.14	126.07	124.17	129.34	126.84
6	165.21	150.21	157.36	156.51	136.81	131.78	115.05	119.32
7	160.30	169.69	156.10	148.95	137.18	130.41	109.00	136.76
8	155.68	157.82	147.65	155.75	149.61	140.63	135.88	148.86
9	159.96	151.08	149.29	154.72	157.46	150.35	153.95	154.74
10	146.74	161.15	152.91	147.26	140.15	140.73	134.75	144.78
11	151.34	161.37	155.21	152.84	153.12	138.37	128.74	116.95
12	154.08	156.14	171.05	169.92	134.41	136.07	112.80	147.37
13	146.04	157.50	156.89	153.03	146.34	128.02	127.83	138.61
14	154.79	146.91	155.81	143.99	152.12	149.27	149.83	151.81
15	154.55	155.48	148.72	166.22	149.98	150.47	145.12	145.78
16	155.40	158.79	167.65	143.77	147.22	151.19	154.13	139.38
17	156.59	148.39	140.15	151.68	149.77	143.42	138.38	152.05
18	147.60	150.78	145.42	146.88	143.49	134.81	138.85	147.81
19	140.64	137.65	136.49	139.33	140.98	133.14	132.56	141.86
20	139.26	140.45	141.50	149.61	141.23	138.42	141.89	145.61
Field 1-Lysosome								
1	68.39	71.55	71.30	67.91	65.66	67.76	66.25	63.31
2	68.67	69.73	69.44	72.37	62.15	63.53	61.77	65.43
3	73.71	72.48	65.28	70.81	68.22	67.67	61.42	68.74
4	72.99	74.39	73.38	71.18	70.35	69.66	69.67	69.67
5	71.93	74.50	72.84	72.17	70.53	73.00	71.75	67.98

6	71.55	75.85	75.01	73.49	71.49	71.38	72.75	71.67
7	70.60	77.38	70.91	68.24	64.06	66.34	65.81	60.63
8	69.37	74.70	73.84	73.74	71.79	72.84	73.26	72.04
9	74.48	76.73	73.26	74.74	73.98	74.61	71.34	73.29
10	71.99	73.86	74.53	70.96	65.53	69.31	69.30	67.87
11	68.85	73.60	73.27	70.13	67.83	70.43	72.85	70.10
12	61.96	65.46	64.54	62.52	61.86	64.77	63.81	60.30
13	71.42	74.39	74.32	73.67	70.66	73.82	72.73	70.71
14	75.67	75.16	77.52	76.50	72.57	73.77	73.43	74.16
15	69.51	71.93	71.59	71.15	69.43	71.48	69.93	68.57
16	71.40	73.71	75.03	72.35	70.44	70.93	66.46	69.69
17	74.51	71.54	74.43	73.28	71.42	73.02	72.21	70.32
18	75.79	73.64	75.46	75.28	67.77	67.48	70.49	68.14
19	69.26	68.41	71.13	70.32	70.10	66.49	67.45	69.24
20	75.71	72.90	75.36	68.00	73.79	74.08	73.31	72.84
21	72.13	67.82	66.39	73.29	65.62	62.18	58.49	67.57
22	73.70	73.61	68.12	74.44	72.08	66.83	66.00	70.85
23	77.53	78.71	74.27	75.21	75.56	67.83	70.36	71.48
24	71.02	68.50	63.96	64.10	64.73	62.01	61.69	61.66
25	74.43	74.61	69.82	70.40	70.60	71.65	62.09	62.77
26	68.94	67.80	59.33	66.15	65.41	60.89	56.42	60.71
Field 2								
1	65.36	67.85	69.57	65.75	60.55	64.75	64.42	60.79
2	68.07	71.01	69.16	67.72	66.90	68.62	67.53	67.24
3	72.47	74.39	73.28	70.92	71.53	72.46	71.79	71.05
4	69.65	72.55	72.80	72.59	69.55	70.00	67.16	67.00
5	74.13	75.07	71.76	68.75	67.78	69.59	71.34	69.83
6	71.30	68.94	71.29	68.74	69.33	70.02	68.08	64.41
7	71.22	74.10	73.20	70.46	70.23	69.97	70.57	69.73
8	68.86	67.89	73.08	71.55	67.27	68.96	69.39	66.54
9	68.77	71.92	71.69	70.01	67.79	71.03	68.90	68.53
10	68.02	69.75	69.86	67.95	66.92	68.29	65.89	65.90
11	71.78	71.58	72.37	70.63	70.04	70.79	71.16	70.53
12	73.26	70.55	72.64	67.83	72.20	70.09	72.01	68.24
13	71.68	76.39	75.19	72.58	67.69	72.40	73.97	71.24
14	76.69	79.10	77.84	76.55	71.91	72.15	70.64	71.88
15	69.92	70.90	68.70	69.84	67.91	67.02	67.32	66.59
16	72.91	70.49	70.12	70.54	69.34	71.18	69.17	66.73
17	69.07	73.36	75.41	75.38	70.19	71.38	67.55	67.18
18	77.56	75.55	74.82	70.39	74.47	74.38	73.20	73.10
19	77.10	75.80	72.41	73.01	72.52	73.00	70.66	72.30
20	71.98	76.06	70.38	78.85	70.89	71.44	70.13	70.65
21	74.96	75.94	79.06	76.33	70.43	71.83	70.00	70.46

22	71.88	73.26	73.15	72.24	69.20	68.21	70.68	69.90
23	72.45	70.64	69.35	74.01	69.60	68.91	69.53	71.86
24	71.15	70.12	72.82	75.12	68.27	70.42	71.57	72.30
25	71.08	73.23	77.54	76.35	72.99	72.35	71.54	73.81
26	70.42	73.79	72.73	74.02	67.63	65.80	65.29	70.17
27	71.11	67.43	69.78	78.00	67.20	64.59	66.87	70.27
28	70.93	67.22	70.58	75.12	69.22	66.28	68.07	71.20
29	72.66	68.91	64.64	69.03	66.72	65.46	64.12	66.37
30	73.54	73.29	64.67	72.01	71.07	68.59	64.06	68.46
Field 3								
1	75.30	76.92	76.34	73.43	71.37	70.42	71.49	72.40
2	74.33	74.59	74.01	73.11	69.18	72.09	70.67	68.57
3	74.33	79.56	73.10	70.26	70.83	76.17	68.21	61.88
4	73.59	72.52	72.40	72.66	73.46	71.87	69.44	70.01
5	71.32	68.14	66.56	73.06	65.04	66.25	67.21	66.92
6	71.74	77.82	75.77	71.81	69.09	71.93	69.77	68.26
7	70.00	74.34	72.49	72.21	66.98	70.41	70.34	69.09
8	72.26	71.46	72.62	71.55	68.56	68.90	70.10	69.27
9	74.55	71.71	73.69	69.86	66.82	73.43	73.12	69.07
10	69.00	70.55	70.31	69.09	66.33	67.03	70.12	65.24
11	74.32	77.05	72.25	70.86	69.25	71.69	72.00	68.84
12	71.04	69.24	69.95	70.79	68.92	65.77	69.40	66.29
13	73.92	72.79	70.78	70.65	71.04	70.40	68.23	68.96
14	74.12	80.57	72.80	73.43	73.84	69.49	69.84	71.37
15	75.39	71.69	69.94	71.88	67.39	70.64	67.68	65.84
16	72.30	73.37	73.86	76.44	69.97	69.58	71.69	67.96
17	75.86	0.00	77.55	78.51	74.55	0.00	72.78	74.86
18	75.87	74.38	74.68	70.22	66.14	67.02	69.12	68.36
19	69.63	71.48	72.03	71.90	65.74	69.15	67.29	67.45
20	66.37	70.21	66.24	65.73	61.52	64.47	68.14	66.76
21	62.01	60.88	67.06	64.22	56.46	53.08	59.26	57.46
22	73.27	74.80	75.35	72.33	68.33	68.09	68.59	66.86
23	74.52	73.03	69.79	72.51	66.36	64.21	68.23	69.90
24	70.09	72.72	69.66	74.10	72.00	71.83	71.22	73.01
25	72.60	71.50	70.50	73.88	66.07	66.96	68.75	64.37
26	73.26	70.39	72.05	72.56	68.45	67.75	69.29	67.64
27	70.77	69.73	72.33	70.11	67.94	65.75	71.12	70.51
28	67.53	68.27	71.34	71.92	68.24	63.38	68.76	63.82
29	74.30	72.99	74.02	75.70	72.57	68.32	70.25	74.42
30	74.64	74.40	72.44	71.98	73.02	65.97	68.28	72.84
31	77.52	74.17	69.20	76.84	73.77	70.05	63.00	68.71
32	73.41	69.23	67.12	71.62	68.04	67.85	66.15	66.21
Field 4								

1	69.44	72.93	72.36	67.09	64.01	66.10	65.40	60.72
2	69.26	75.26	72.90	71.71	64.18	68.79	64.13	62.95
3	69.50	74.09	73.23	66.90	64.23	64.15	65.97	61.96
4	71.16	76.79	68.91	66.62	57.34	64.89	57.63	59.01
5	72.76	77.29	74.01	71.89	69.62	72.40	68.35	65.88
6	77.69	74.98	72.71	71.63	70.85	72.21	68.41	68.63
7	74.67	77.08	76.42	72.01	72.98	70.01	67.40	66.12
8	77.84	77.39	75.67	80.19	68.89	67.92	66.23	66.57
9	72.44	75.88	73.59	77.27	72.87	71.58	75.85	67.36
10	71.91	73.16	71.18	72.36	68.77	72.31	72.24	67.39
11	79.61	79.89	71.93	70.04	67.16	71.21	71.72	70.79
12	73.29	75.07	76.82	72.86	73.59	73.08	74.95	72.43
13	75.63	76.52	74.42	71.95	69.85	74.98	73.05	70.01
14	67.87	68.80	69.97	64.25	55.57	61.85	63.66	56.49
15	64.93	68.76	72.54	67.83	60.23	62.66	57.84	57.01
16	64.96	70.46	69.28	65.78	60.93	63.29	65.12	63.10
17	69.67	72.82	74.70	70.64	65.18	69.56	64.40	65.87
18	68.90	71.62	73.94	68.15	67.07	70.86	67.47	64.32
19	73.77	74.90	73.24	73.84	69.28	71.56	71.99	70.52
20	76.27	75.69	77.02	76.41	67.91	70.59	72.58	70.20
21	72.70	76.58	73.81	72.12	69.47	71.95	74.44	74.16
22	74.32	76.40	73.49	70.46	63.41	67.39	62.53	69.04
23	70.86	77.71	74.98	78.68	74.76	68.42	70.38	74.63
24	71.26	74.89	79.80	76.30	68.22	72.42	70.98	71.45
25	73.52	75.89	73.47	76.10	70.88	71.64	71.76	71.65
26	69.94	73.43	79.62	79.85	68.41	69.56	64.12	65.34
27	77.31	81.06	76.32	73.96	69.16	65.51	68.74	73.91
28	78.19	74.41	71.88	74.59	68.08	69.10	68.89	65.97
29	75.92	72.08	68.80	77.82	67.42	66.31	66.54	66.20
30	85.15	75.36	73.37	74.42	67.58	68.81	64.78	68.68
31	81.46	74.99	76.56	73.66	73.14	71.80	68.38	73.81
32	77.13	74.28	74.84	75.49	71.43	75.05	69.83	69.47
33	76.45	73.38	77.59	75.29	70.52	72.56	67.23	69.39
34	72.00	73.09	77.65	76.30	76.93	73.01	70.91	74.18
Field 1-Actin								
1	137.13	139.53	136.89	134.78	140.49	140.92	139.08	134.35
2	135.40	129.44	130.96	132.94	137.11	133.23	132.79	136.05
3	134.29	136.68	136.14	139.23	135.14	135.46	137.37	135.95
4	134.79	135.81	133.41	135.83	138.09	138.98	138.56	137.21
5	134.41	138.03	136.06	135.45	141.66	140.00	137.51	139.42
6	135.35	139.51	137.33	138.24	140.26	139.13	139.10	143.76
7	136.96	138.17	141.24	138.86	142.38	143.30	138.45	141.64
8	143.70	146.62	146.24	145.54	142.39	146.01	144.14	143.93

9	145.08	141.87	139.45	138.76	141.80	139.78	143.12	142.41
10	147.52	143.92	140.50	144.76	146.08	148.44	146.42	143.09
11	141.09	141.63	141.30	140.41	143.23	144.23	141.16	144.12
12	140.04	139.76	138.07	139.34	141.20	140.97	141.05	141.89
13	140.51	139.04	137.56	141.04	141.50	136.85	142.45	141.61
14	138.03	133.08	133.59	135.89	140.23	135.35	137.14	138.90
15	145.16	140.95	141.63	141.79	143.28	140.70	144.65	145.60
16	144.72	144.69	141.01	143.01	143.09	142.87	145.02	145.28
17	128.74	134.28	132.45	131.39	131.06	136.48	133.47	130.28
18	123.06	123.64	124.34	122.20	124.55	126.24	127.75	124.10
19	127.73	133.22	132.61	128.45	129.09	133.98	131.25	129.45
20	124.79	123.96	131.45	125.18	127.25	127.48	129.00	126.81
21	128.28	131.46	128.12	127.41	132.87	135.19	131.24	128.15
22	120.20	126.88	124.73	120.07	125.11	127.39	122.01	119.50
23	134.56	129.46	131.12	128.98	136.49	132.17	133.08	137.45
24	132.00	131.22	128.19	129.79	137.79	132.94	129.33	132.93
25	124.77	121.49	123.78	126.22	129.55	125.12	125.33	129.37
26	131.61	137.57	133.47	132.52	137.38	136.43	136.21	136.70
Field 2								
1	153.16	157.91	160.63	157.78	150.35	156.01	159.21	155.93
2	152.34	158.64	162.06	157.24	150.69	157.38	161.47	154.36
3	157.81	141.91	138.39	138.11	154.07	141.57	136.25	138.56
4	152.14	150.85	154.23	150.31	150.55	145.04	152.30	144.13
5	147.19	152.29	160.71	148.25	143.73	152.81	160.85	148.46
6	148.47	147.83	144.95	148.65	147.69	145.93	142.29	148.60
7	145.23	147.90	145.25	141.95	147.25	147.45	142.26	140.34
8	138.98	140.82	145.67	140.23	135.27	142.99	143.39	137.06
9	132.48	136.51	135.17	130.28	131.51	135.56	133.59	128.95
10	142.59	143.73	145.68	147.10	139.30	143.26	143.24	144.85
11	146.04	150.54	150.16	145.53	145.94	151.24	149.93	143.43
12	140.93	143.88	143.25	141.47	139.61	141.71	141.44	137.46
13	149.74	150.45	148.87	149.97	151.20	149.84	147.58	147.74
14	154.49	149.65	149.04	154.43	153.79	147.93	146.71	153.01
15	143.11	141.50	136.94	141.55	141.47	133.88	135.97	142.50
16	150.59	152.79	145.13	141.66	149.57	151.47	144.37	141.19
17	148.39	151.78	147.43	144.78	146.66	149.56	145.62	138.81
18	138.73	140.31	144.09	143.89	137.14	138.16	139.94	141.59
Field 3								
1	159.29	154.89	158.08	152.19	158.06	159.58	154.28	154.41
2	147.54	151.98	149.03	148.61	149.02	149.53	148.71	145.38
3	149.96	152.41	156.50	153.36	150.71	155.53	150.36	151.31
4	156.58	152.66	157.94	153.94	155.33	155.03	156.24	152.15
5	163.73	165.24	163.58	164.55	161.00	164.49	161.62	161.07

6	156.87	163.91	151.36	151.00	158.98	162.61	146.96	150.41
7	145.62	149.75	149.43	146.15	145.76	146.23	148.66	146.74
8	147.02	148.96	152.83	146.08	146.03	149.07	149.97	148.89
9	143.05	149.39	145.74	141.96	142.13	142.72	145.03	139.90
10	157.01	151.22	147.76	152.70	155.92	152.16	146.42	152.40
11	153.48	154.63	161.04	154.74	153.41	158.04	159.20	153.49
12	147.29	151.10	150.93	144.49	146.32	150.34	146.74	144.81
13	144.68	144.98	145.28	145.81	143.10	144.90	146.62	145.94
14	158.14	158.06	158.80	157.76	156.75	154.82	160.45	157.75
15	159.18	154.95	157.48	160.19	157.18	153.63	157.12	158.27
16	147.72	147.94	142.08	145.48	147.64	146.98	143.13	146.41
17	150.61	150.70	151.74	149.99	150.16	155.38	153.33	150.66
18	145.77	148.97	151.64	152.40	144.45	144.58	148.72	146.98
19	145.87	146.90	157.61	151.16	143.07	147.91	153.62	150.72
20	163.85	160.36	161.11	165.15	164.68	160.41	161.27	167.19
21	153.49	156.05	157.84	160.42	153.14	152.11	157.76	156.54
22	151.83	151.99	148.59	155.14	153.03	148.91	149.92	155.41
23	157.23	151.51	155.31	154.07	155.47	153.22	151.66	156.35
24	155.08	150.91	157.61	156.75	151.85	154.05	151.29	157.12
25	155.64	148.36	159.92	158.68	154.06	152.61	153.22	157.52
Field 4								
1	153.72	153.89	154.71	152.12	153.53	154.82	152.75	147.90
2	148.85	152.82	151.82	152.04	144.63	150.58	148.96	140.29
3	140.27	144.41	145.44	139.70	137.27	141.15	139.26	137.39
4	145.20	147.58	150.66	146.52	142.30	147.84	147.64	143.56
5	149.51	149.20	150.68	148.33	142.97	148.42	148.76	145.77
6	152.16	150.86	149.38	152.14	146.61	147.00	146.40	149.23
7	145.17	155.17	153.74	144.19	143.98	150.23	150.85	142.75
8	156.37	163.14	158.23	159.46	157.45	158.14	157.80	154.80
9	159.95	161.76	158.07	158.55	153.37	157.98	154.59	153.51
10	154.11	134.60	144.52	147.74	153.01	133.89	141.19	144.18
11	149.35	148.59	143.20	142.33	137.98	140.47	139.62	136.97
12	145.82	148.55	151.11	146.34	146.87	149.90	148.57	144.80
13	150.99	149.06	154.44	154.41	150.84	155.31	149.32	148.42
14	152.05	146.50	147.23	154.17	152.76	146.41	145.96	150.42
15	147.18	144.88	149.96	149.09	144.04	143.91	146.42	147.91
16	137.12	144.08	145.91	138.90	137.77	142.96	141.79	139.91
17	153.08	152.94	150.97	154.78	148.57	149.23	146.58	150.64
18	158.97	153.35	157.15	163.31	157.38	149.36	152.78	158.44
19	161.58	157.09	156.42	159.03	160.31	156.65	158.05	155.65
20	155.95	152.20	157.50	159.59	156.41	151.44	150.73	153.94
21	138.14	134.19	142.17	142.53	137.52	132.45	135.40	141.58
22	134.43	127.22	131.33	138.19	129.93	127.47	129.67	135.18

23	147.80	149.32	149.13	147.03	147.92	150.98	142.72	142.29
24	145.61	150.73	153.74	147.86	149.26	154.51	145.46	141.29
25	155.97	149.87	156.39	157.81	152.37	150.30	157.16	153.74
26	157.08	146.70	149.45	153.05	153.52	143.17	143.39	153.36
27	142.47	140.21	148.31	147.51	140.55	137.57	140.80	146.58
28	155.89	153.28	155.57	160.83	149.36	153.88	142.48	144.19
29	154.31	157.15	152.27	153.86	147.89	155.06	151.14	151.85
30	148.64	152.85	152.15	153.77	133.74	136.78	152.99	148.97
31	142.34	145.05	150.20	143.70	138.82	143.25	147.38	143.38
32	141.95	139.42	140.45	137.51	136.96	137.02	138.74	134.60
33	136.91	138.43	137.85	134.97	135.20	135.86	135.61	131.75
34	135.41	136.63	136.13	139.25	135.53	136.06	137.38	138.24
35	132.75	132.34	133.34	133.97	134.76	128.57	131.52	132.83
36	139.24	132.21	132.26	134.38	126.70	133.97	132.46	133.91
Field 1-Tubulin								
1	136.41	125.05	125.53	131.34	133.85	126.89	124.29	128.28
2	130.84	121.34	129.14	128.24	124.73	120.88	116.29	123.81
3	133.54	125.80	123.97	132.94	127.25	128.01	118.19	123.87
4	124.08	126.53	123.60	122.74	128.49	126.49	122.20	126.47
5	133.32	135.57	135.66	132.23	127.94	134.74	129.08	135.05
6	124.81	130.76	131.52	133.32	127.29	125.72	126.41	124.39
7	132.14	136.06	132.74	124.57	121.15	129.32	123.43	123.27
8	123.77	125.27	121.36	125.64	120.46	120.21	124.98	121.18
9	124.85	121.14	118.52	127.77	120.82	121.75	125.56	121.94
10	136.75	137.95	132.09	137.79	134.19	131.65	130.13	132.08
11	136.52	127.27	127.89	129.99	132.25	129.02	123.83	128.84
12	134.07	122.73	114.29	136.16	118.94	120.72	119.74	124.87
13	111.04	105.00	99.55	112.15	108.33	103.21	105.37	113.09
14	132.18	124.68	128.82	141.40	130.56	124.36	125.65	128.81
15	131.55	112.07	124.49	123.72	126.59	121.49	122.82	129.46
16	129.07	133.61	123.92	124.83	126.58	112.76	120.62	121.75
17	119.49	116.72	121.06	122.26	117.94	114.11	115.00	123.77
18	111.45	125.92	121.46	121.25	105.55	126.98	121.41	115.92
19	124.01	128.99	131.63	122.94	119.28	124.53	125.94	113.80
20	116.28	118.12	121.79	113.20	116.60	120.70	121.01	115.47
21	100.38	105.51	107.96	99.33	106.06	108.12	105.67	89.91
Field 2								
1	124.61	131.72	132.76	128.45	118.55	127.29	126.68	126.04
2	123.53	127.93	129.63	123.83	118.01	123.10	123.05	120.10
3	124.02	127.34	128.91	123.58	121.82	123.06	125.38	120.54
4	126.18	131.43	131.75	126.13	120.48	124.11	125.38	119.49
5	125.18	132.32	131.46	125.03	118.87	128.66	125.10	118.85
6	120.04	125.25	128.18	118.48	114.70	119.85	118.45	109.26

7	123.32	128.11	129.82	123.10	119.44	124.74	125.86	118.57
8	127.09	131.46	132.31	128.81	120.82	129.42	129.59	125.67
9	131.72	133.62	135.60	131.88	125.83	128.09	132.55	123.41
10	137.15	137.37	141.43	138.29	132.62	130.63	128.27	127.81
11	134.06	129.27	134.04	134.58	127.18	121.20	123.62	124.85
12	130.52	135.93	136.03	129.30	127.62	133.31	133.69	124.35
13	137.41	135.11	136.17	137.53	133.45	129.65	127.30	132.67
14	137.65	137.43	136.80	135.73	128.91	131.78	135.58	134.92
15	134.55	134.59	133.47	131.12	130.04	131.33	133.32	126.51
16	136.29	123.97	123.41	132.44	127.29	122.39	119.78	124.36
17	129.40	123.19	121.55	130.50	122.14	121.19	121.34	122.99
18	124.37	124.10	119.35	128.49	126.01	115.75	117.45	122.32
19	130.67	126.71	127.99	130.64	127.54	122.88	124.47	126.62
20	126.18	119.17	121.36	126.68	120.23	114.81	113.98	120.87
21	126.26	124.41	124.39	129.23	125.97	113.86	120.66	123.55
22	125.28	121.28	121.65	121.72	122.19	118.58	116.63	118.45
23	123.54	116.74	117.52	125.38	117.72	110.25	114.44	117.51
24	120.54	114.62	117.05	121.98	115.08	111.55	110.57	116.71
Field 3								
1	139.31	142.18	136.82	134.39	132.52	135.07	136.25	129.88
2	141.64	142.38	141.13	140.04	140.46	141.51	136.92	139.05
3	123.62	127.21	126.66	122.97	120.72	127.03	125.06	119.16
4	126.38	131.92	131.93	124.88	125.15	127.97	124.40	124.35
5	127.19	131.10	129.53	126.16	115.18	127.42	120.55	120.01
6	130.04	134.86	137.86	131.57	128.63	133.83	129.13	127.58
7	130.78	131.54	136.17	130.54	133.22	133.96	130.69	131.57
8	133.16	136.50	139.58	134.96	133.09	135.43	136.39	135.51
9	130.17	137.22	141.45	137.64	130.57	136.03	135.19	133.59
10	131.04	135.09	137.75	141.59	131.66	136.71	134.22	129.13
11	132.16	131.41	133.52	133.09	134.07	131.61	132.00	128.94
12	112.49	118.44	117.96	112.08	109.70	116.40	113.50	110.93
13	107.96	113.53	112.97	108.65	102.87	106.26	107.62	105.25
14	129.24	127.67	124.63	130.31	130.70	125.14	127.43	128.95
15	137.48	137.67	134.68	137.46	134.88	133.10	131.99	132.87
16	141.23	139.26	142.84	138.21	132.87	130.60	134.90	136.14
17	135.49	135.76	135.78	137.00	135.90	131.77	132.98	135.41
18	143.38	137.90	135.65	141.05	137.28	127.96	131.13	132.63
Field 4								
1	138.59	133.71	137.15	136.97	136.08	132.92	130.81	134.91
2	134.87	127.65	117.53	130.84	129.68	127.60	118.81	124.00
3	145.94	149.55	147.19	135.83	140.38	140.21	145.78	133.13
4	134.15	137.13	142.23	138.09	128.69	126.17	125.97	131.17
5	133.39	144.66	136.89	138.04	129.06	137.77	136.35	133.45

6	125.84	147.63	155.43	140.28	132.17	136.64	136.95	130.79
7	126.15	131.91	131.47	132.17	122.25	129.83	129.73	124.55
8	126.73	122.84	124.62	124.69	119.81	120.68	123.81	121.36
9	127.90	134.15	133.71	127.05	118.66	132.11	133.10	125.22
10	127.77	130.07	138.20	132.43	118.75	128.17	129.19	125.28
11	134.30	134.17	133.16	136.57	124.31	130.77	127.22	124.68
12	133.73	132.98	140.49	131.22	126.97	124.62	135.27	129.33
13	137.84	137.79	144.74	142.45	138.92	132.41	137.74	142.53
14	144.47	146.34	145.01	141.72	142.06	140.18	139.94	142.73
15	139.36	134.87	136.73	153.24	138.46	135.69	131.13	139.13
16	133.32	128.49	132.42	132.26	127.05	126.09	121.59	131.89
17	126.30	125.57	119.97	119.04	127.17	122.56	112.90	116.69
18	123.99	122.83	126.60	130.72	119.70	119.69	122.45	131.22
19	128.12	125.17	132.32	134.51	123.87	122.84	125.84	132.60
20	123.30	116.84	122.82	129.60	123.38	115.60	122.05	128.09
Field 5								
1	125.04	136.48	132.46	125.62	123.92	133.39	132.89	122.25
2	133.91	145.85	149.75	136.61	131.79	140.12	139.66	136.24
3	135.12	143.19	144.20	131.15	130.59	140.85	139.74	130.63
4	142.07	139.69	140.32	136.80	133.72	142.92	140.11	131.64
5	125.21	132.33	135.44	130.01	123.65	130.27	132.54	122.59
6	139.62	150.51	141.32	134.34	138.99	143.29	138.49	130.60
7	114.38	123.86	123.36	118.28	108.71	118.23	122.63	116.90
8	132.20	124.79	122.07	136.48	123.12	122.05	121.72	128.93
9	125.19	122.66	126.39	132.45	120.93	117.06	121.11	121.73
10	120.18	119.18	127.73	129.04	118.37	115.89	115.08	120.94
11	134.88	134.59	133.25	144.34	135.20	128.88	128.60	138.06
12	117.98	117.01	122.31	124.99	117.81	115.77	118.93	125.82
13	131.75	133.80	129.63	136.81	131.89	130.91	129.82	135.74
14	149.68	141.48	139.45	143.27	141.03	138.57	135.16	139.49
15	134.33	125.18	128.89	134.44	132.39	127.50	126.55	131.77
16	134.13	124.51	127.78	131.79	134.18	129.58	126.05	128.19
17	129.37	128.02	124.91	134.53	127.76	127.98	125.84	125.73
18	134.29	128.11	126.89	128.69	132.43	126.36	122.42	126.82

Table S10: Raw APVs of fluorescent images of organelles in hypotonically treated RAW264.7 cell population

	Circularity	*Octant 1*	*Octant 2*	*Octant 3*	*Octant 4*	*Octant 5*	*Octant 6*	*Octant 7*	*Octant 8*
Nucleus									
Field 1									

1	0.83	6.54	5.33	0.75	2.54	8.22	8.58	1.54	2.73
2	0.97	3.23	5.57	4.54	5.65	5.80	8.52	5.58	6.82
3	0.97	1.68	4.29	10.92	3.77	4.10	4.45	11.81	7.99
4	1.02	9.04	6.44	5.03	2.13	9.42	8.19	7.30	2.46
5	1.00	7.74	5.08	1.46	1.03	9.84	6.41	2.53	2.33
6	0.96	3.55	3.64	2.82	3.09	3.25	5.08	4.62	4.35
7	0.69	3.77	6.65	1.16	3.48	4.43	7.35	1.27	3.04
8	1.01	10.16	3.80	5.29	9.66	3.42	3.20	3.34	2.90
9	1.01	6.31	8.52	4.91	1.75	2.70	4.75	3.59	1.57
10	0.91	5.01	5.60	3.54	4.49	1.70	3.46	2.22	1.18
11	0.91	7.74	8.49	1.51	4.26	7.41	6.19	1.63	3.31
12	0.76	2.40	7.18	3.75	0.49	2.05	5.65	2.51	0.95
13	0.97	1.34	1.25	3.80	1.09	1.12	1.13	4.59	1.15
14	0.90	2.30	12.38	2.40	2.25	2.12	9.99	3.28	2.23
15	0.76	2.93	1.79	3.20	3.26	2.72	1.30	2.63	2.26
16	0.93	2.59	3.17	4.08	5.59	1.86	2.47	4.31	5.34
17	0.79	5.24	6.54	6.04	2.10	4.05	5.44	6.00	2.48
18	1.00	8.39	9.21	5.26	4.56	2.39	4.25	2.00	1.53
19	1.03	11.47	8.25	1.71	2.02	10.39	10.54	1.90	2.21
20	0.99	5.33	7.75	7.91	4.24	5.96	6.53	4.36	2.96
21	0.97	8.60	9.25	7.43	6.95	2.81	4.66	3.29	2.49
22	0.99	4.11	2.45	8.12	8.69	5.42	3.91	6.51	6.91
23	1.00	3.89	5.78	6.96	5.80	3.56	7.96	10.29	4.72
24	0.97	4.71	5.70	5.55	7.47	4.55	5.71	6.38	7.67
25	0.97	7.73	7.60	7.84	4.52	3.19	5.70	8.84	5.04
26	0.81	3.80	2.42	4.14	3.55	4.46	3.40	4.87	3.77
27	1.00	5.83	6.56	4.38	4.81	6.09	5.14	4.61	4.91
28	0.99	3.78	3.20	7.07	4.47	4.63	3.95	8.02	6.53
29	0.96	2.07	6.24	4.16	2.99	3.36	8.05	5.49	4.76
Field 2									
1	0.92	3.70	4.07	3.63	3.68	9.12	7.70	3.77	6.76
2	0.65	2.99	3.99	1.14	2.50	5.79	10.51	2.06	3.84
3	0.90	1.22	2.23	6.88	4.76	1.98	4.06	9.58	9.01
4	0.39	3.16	4.87	1.44	2.70	3.70	7.35	1.66	2.78
5	0.67	12.90	12.04	11.85	10.68	0.56	0.68	0.51	0.45
6	1.00	8.71	2.58	7.13	14.22	4.56	1.68	2.90	6.66
7	0.92	4.33	5.93	3.35	8.32	3.37	4.27	5.06	6.31
8	0.99	5.92	7.57	5.27	8.25	3.22	3.16	3.94	4.90
9	0.95	6.22	8.82	3.33	1.73	1.63	7.51	4.10	1.30
10	0.98	9.31	6.52	6.81	8.83	3.16	1.42	1.71	4.04
11	0.98	8.71	7.95	5.26	4.18	3.33	5.14	1.95	1.39
12	0.87	2.64	4.56	2.26	4.06	1.76	1.90	1.29	2.36
13	0.71	2.86	3.32	2.60	1.32	3.65	4.31	3.02	1.42

14	0.86	1.87	1.05	1.74	3.04	2.32	0.78	3.27	6.68
15	0.96	10.97	7.63	4.14	5.71	7.25	3.55	1.95	4.14
16	1.01	4.71	9.18	8.20	2.08	3.22	7.22	6.95	2.07
17	0.80	3.27	4.64	3.30	1.60	3.13	4.10	3.45	2.00
18	0.56	3.64	3.55	5.40	3.23	3.35	3.35	7.89	4.32
19	0.64	4.51	7.16	3.81	4.07	3.93	6.04	3.76	4.87
Field 3									
1	1.01	0.71	0.93	1.46	1.14	3.19	4.11	8.66	9.24
2	0.96	1.65	2.33	1.47	0.91	9.27	7.86	3.31	3.74
3	0.99	3.06	1.75	0.38	0.46	12.64	6.79	1.31	1.55
4	0.98	1.34	0.43	1.39	2.98	5.21	1.06	6.57	12.33
5	0.53	1.99	0.45	2.13	2.29	5.42	2.33	6.45	4.02
6	0.64	1.14	0.63	0.48	1.29	4.09	2.38	1.78	4.52
7	0.99	3.24	1.60	1.23	2.77	6.07	3.37	2.58	6.14
8	0.99	3.64	2.68	2.94	3.75	5.96	2.81	3.97	6.96
9	0.63	1.30	1.14	1.87	0.24	7.73	4.87	8.28	2.85
10	0.39	1.34	1.13	0.35	0.93	8.30	5.69	2.98	8.43
11	0.98	1.07	0.66	0.56	0.67	7.55	4.30	5.22	6.69
12	0.83	0.70	0.96	0.13	0.05	7.05	6.97	2.42	0.72
13	0.77	0.99	0.62	0.92	0.61	7.20	2.97	5.31	5.27
14	0.97	2.80	11.01	4.99	2.11	1.96	12.22	10.55	2.73
15	0.97	6.27	5.40	2.73	2.11	5.03	6.37	8.72	3.81
16	0.50	3.62	1.39	0.03	0.38	7.53	3.75	0.32	1.13
17	0.41	0.29	0.05	0.05	0.16	4.35	0.66	0.82	3.37
18	0.43	0.29	0.02	0.16	0.56	3.23	0.08	0.71	6.27
19	0.96	2.43	0.48	0.47	1.52	9.57	2.08	1.42	6.80
20	0.80	0.88	0.13	0.32	1.23	5.95	1.35	2.20	8.04
21	0.17	0.03	0.69	0.32	0.45	0.50	5.87	3.14	6.35
Field 4									
1	0.74	0.58	0.32	0.14	0.06	3.15	2.20	0.99	0.61
2	0.21	1.62	1.80	0.07	1.46	2.33	3.20	0.71	3.29
3	0.59	1.69	1.55	0.62	0.25	5.09	9.42	2.41	0.75
4	0.95	8.02	6.51	4.33	3.16	4.26	6.67	3.26	1.46
5	0.56	1.85	3.79	2.47	3.60	3.99	3.79	3.78	6.72
6	0.99	2.78	3.94	3.82	2.85	3.64	2.99	4.56	4.43
7	0.71	1.92	2.15	2.87	3.58	4.85	4.03	4.19	7.63
8	0.91	2.07	0.69	2.11	3.90	3.51	2.87	7.45	7.36
9	0.91	5.76	4.83	0.76	1.71	7.94	6.23	1.05	1.95
10	1.00	5.13	4.14	3.89	5.53	3.32	2.95	3.93	5.50
11	0.99	2.96	7.73	2.69	1.55	1.37	3.60	2.62	0.95
12	0.94	5.81	5.25	5.11	5.77	4.85	3.98	3.22	4.21
13	0.86	0.22	1.00	4.21	2.12	1.23	2.37	7.16	5.84
14	0.96	3.30	6.79	3.60	1.83	3.55	5.75	3.70	2.33

15	0.95	1.66	1.30	3.58	3.95	1.86	1.23	3.83	4.90
16	1.00	1.86	5.27	6.43	2.50	1.91	3.72	6.34	2.98
17	0.94	2.07	3.15	1.20	0.30	2.85	2.95	2.46	2.19
18	1.00	5.79	6.41	5.11	5.59	5.53	6.96	4.17	4.78
19	0.35	4.40	3.89	0.81	1.24	4.73	3.65	0.96	1.44
Field 5									
1	0.99	3.88	3.24	3.54	4.45	2.96	4.91	5.50	5.26
2	1.00	4.86	1.83	2.44	5.54	8.61	3.01	3.74	9.37
3	0.56	0.09	1.10	3.76	5.93	0.66	5.40	6.60	8.42
4	1.02	5.99	9.85	2.93	1.32	6.14	11.69	3.56	1.96
5	0.84	6.06	10.12	7.17	2.58	4.69	11.27	9.09	2.96
6	0.92	7.86	5.55	4.62	3.95	6.99	2.66	3.92	4.21
7	0.99	7.56	4.48	6.89	10.92	5.41	4.04	5.83	8.38
8	0.90	2.79	1.05	2.29	1.45	8.04	2.47	7.33	4.87
9	0.95	5.46	4.74	2.40	1.41	7.96	5.31	4.19	5.53
10	1.03	3.32	0.90	4.29	10.32	6.46	1.87	7.46	14.48
11	0.99	5.96	10.86	3.87	3.13	8.75	10.82	3.23	2.92
12	0.64	2.10	1.14	1.55	0.69	8.43	3.61	4.47	2.51
13	0.69	3.06	2.42	0.16	0.43	8.49	5.15	1.14	2.22
14	0.96	8.98	3.12	1.69	4.48	5.36	2.03	1.17	3.13
15	0.28	10.35	4.24	2.23	1.12	10.47	3.38	5.32	2.40
16	0.98	3.04	6.16	10.16	6.71	2.59	4.41	13.76	10.80
17	0.95	4.64	1.75	6.38	4.96	5.72	2.01	3.80	5.57
18	1.02	7.47	7.19	5.02	6.33	6.00	2.97	2.72	4.51
19	1.02	9.86	11.78	1.36	1.38	7.62	8.90	1.24	1.64
20	0.98	11.69	3.64	1.42	9.76	11.43	1.72	1.58	7.79
21	0.36	5.36	0.48	3.25	3.39	4.25	0.86	3.94	2.99
Cell Membrane									
Field 1									
1	0.88	4.95	5.45	4.73	3.74	9.46	8.17	6.52	6.50
2	0.22	3.90	4.45	4.21	2.94	7.04	5.74	4.84	9.21
3	0.29	2.52	4.04	2.83	2.10	6.16	10.89	9.30	5.76
4	1.00	4.72	4.81	4.56	3.74	8.45	7.37	5.99	4.89
5	0.99	8.50	7.24	7.60	9.39	14.73	13.53	11.01	13.10
6	0.27	6.87	1.83	2.36	5.43	9.03	5.37	6.64	8.48
7	0.21	3.20	4.53	2.78	3.94	5.77	5.22	5.64	5.99
8	1.01	8.46	9.83	13.04	9.94	9.68	10.87	10.95	7.54
9	0.99	7.12	6.22	6.81	6.64	10.99	13.00	14.16	14.13
10	0.17	4.11	1.90	1.74	2.37	6.11	5.23	4.40	5.07
11	0.26	2.81	2.40	3.08	2.11	9.13	7.40	7.15	6.39
12	0.37	4.89	2.72	3.93	2.69	8.33	4.67	7.10	6.98
13	0.30	4.46	8.80	3.89	6.73	7.38	11.21	8.54	11.25
14	0.78	2.75	2.73	2.52	3.04	6.18	5.84	5.16	8.23

15	0.96	7.07	3.72	5.03	6.69	7.84	5.89	5.59	6.02
Field 2									
1	1.01	10.93	8.87	15.68	14.05	7.09	7.16	8.56	8.08
2	1.01	14.37	14.66	7.33	5.39	7.71	7.33	7.38	6.19
3	0.98	7.14	7.77	6.62	6.29	5.46	5.65	4.74	3.94
4	0.80	7.82	8.80	6.79	5.55	8.72	8.14	6.16	5.93
5	0.80	7.54	6.77	5.40	6.14	5.99	8.25	7.01	5.54
6	0.97	8.75	8.34	12.48	10.26	7.23	5.77	9.58	7.49
7	0.97	7.83	14.72	6.11	4.18	6.55	9.51	6.79	5.08
8	0.99	9.22	17.50	13.55	7.19	8.03	9.51	11.12	10.29
9	0.98	8.14	12.71	15.91	18.84	6.46	8.37	7.66	9.55
10	0.84	7.03	8.48	8.39	9.47	6.20	8.02	7.83	6.31
11	0.97	4.61	3.47	6.38	6.76	9.12	6.88	9.37	8.40
12	0.98	3.62	7.83	5.83	3.07	5.04	8.25	8.93	5.70
13	0.97	3.89	6.21	10.33	7.71	4.61	5.40	9.57	5.62
14	0.63	4.54	4.36	2.57	3.72	9.09	8.68	7.02	7.72
15	0.26	3.39	1.92	3.57	5.05	9.51	9.97	7.00	8.35
16	0.96	2.16	3.37	4.61	3.86	3.18	5.65	8.39	6.24
17	0.89	4.04	3.46	3.06	4.06	7.95	5.61	3.72	6.04
18	0.90	6.40	9.11	11.68	6.57	7.31	8.36	10.64	7.35
19	0.90	15.49	8.27	5.88	11.13	10.45	7.63	7.63	9.75
20	0.78	2.38	7.11	6.32	2.42	4.62	10.53	13.21	4.86
21	0.75	7.18	4.91	6.19	7.07	10.53	12.77	10.22	13.03
22	0.33	3.03	4.39	3.08	3.13	10.71	14.83	10.37	9.06
23	0.84	6.33	6.93	3.13	4.52	14.21	14.95	9.92	11.53
24	0.56	2.42	1.91	4.00	4.83	12.83	9.52	13.44	15.83
25	0.98	22.52	14.98	5.95	11.33	7.27	6.00	4.79	7.73
26	0.99	8.15	11.03	3.67	7.12	10.15	13.35	8.81	10.96
Field 3									
1	0.88	9.93	5.02	5.99	9.43	11.99	7.20	7.27	9.14
2	0.97	9.48	15.10	13.36	11.63	14.76	17.33	12.45	9.96
3	0.92	9.91	22.06	11.52	6.98	8.02	15.13	10.55	8.17
4	0.91	21.70	18.73	16.26	11.08	11.69	12.29	10.64	9.72
5	1.00	12.03	11.20	11.31	13.06	12.38	12.12	12.00	12.53
6	0.95	26.28	16.08	13.47	21.21	17.20	12.19	9.97	17.38
7	0.98	15.34	32.56	25.18	19.12	10.17	18.20	17.54	11.84
8	0.97	10.42	16.76	11.51	10.31	21.07	15.12	10.92	16.60
9	0.95	5.96	4.92	3.09	3.78	14.26	10.82	5.94	10.12
10	1.00	10.10	8.81	10.14	6.95	14.23	10.18	12.87	9.55
11	0.75	7.17	5.29	5.97	5.21	9.46	13.34	6.58	8.03
12	0.82	3.94	5.84	5.82	3.05	7.98	11.07	10.49	9.19
13	0.51	9.37	3.82	10.32	6.40	19.70	18.37	18.40	20.01
14	0.96	15.74	13.40	17.41	10.72	25.84	37.63	28.86	17.70

15	0.45	7.39	6.22	9.36	4.43	20.53	23.14	22.21	17.40
16	0.72	10.27	7.77	13.20	10.54	15.70	14.45	22.83	17.26
17	1.01	4.93	12.48	6.48	5.05	8.99	13.15	8.87	7.49
18	0.95	14.36	10.35	11.81	18.86	17.49	11.34	12.32	14.82
19	0.89	8.62	17.93	21.94	14.54	7.31	9.09	12.58	6.91
20	0.37	14.77	12.95	5.23	10.06	10.91	9.45	6.60	11.78
21	0.85	5.19	5.71	6.10	6.03	11.05	9.80	10.76	12.48
22	0.88	6.12	9.17	6.40	5.76	12.03	14.36	9.58	13.09
23	0.98	9.33	6.45	4.51	4.63	18.13	11.89	11.66	15.98
24	0.99	6.27	14.37	10.64	6.13	7.63	12.73	16.11	10.60
Field 4									
1	0.95	4.87	6.45	11.09	6.19	8.35	9.52	9.92	9.16
2	0.93	11.01	7.10	5.59	5.35	11.45	8.75	6.35	8.45
3	0.98	6.39	14.44	13.48	7.43	6.32	14.29	13.55	8.18
4	0.96	12.14	9.41	7.25	7.84	11.83	10.67	6.28	7.48
5	1.00	5.36	5.01	5.01	6.63	6.56	7.64	8.66	8.78
6	0.92	6.78	5.09	9.05	7.16	6.79	7.13	10.57	10.03
7	0.97	10.27	9.90	9.03	10.37	10.35	10.13	9.65	10.33
8	0.93	9.27	12.92	9.29	6.39	6.44	9.69	11.71	5.79
9	0.87	3.06	4.20	4.21	3.75	7.08	8.88	8.32	5.93
10	0.55	3.03	3.73	3.80	3.73	8.75	7.80	7.90	7.63
11	0.99	12.78	11.45	15.61	17.58	11.61	10.94	13.00	13.19
12	0.91	9.79	5.83	7.70	8.25	10.93	6.93	6.19	5.60
13	0.98	3.18	5.92	8.34	7.03	4.50	8.98	10.27	8.54
14	0.97	2.77	5.64	6.46	4.39	4.34	7.99	10.03	7.98
15	0.98	8.98	5.87	7.96	10.73	5.44	5.80	7.12	8.09
16	0.59	15.59	7.34	9.70	15.28	11.25	8.73	9.58	10.17
Field 5									
1	0.96	10.38	13.15	6.92	7.58	17.93	14.07	9.13	15.14
2	0.71	2.07	4.22	4.50	2.81	7.83	9.63	13.09	6.53
3	0.83	9.29	4.28	3.93	5.81	10.43	8.26	9.43	10.67
4	0.94	12.00	4.69	4.97	7.83	11.86	6.94	7.53	9.35
5	0.87	4.82	8.11	7.44	3.42	9.76	9.27	10.54	9.80
6	0.93	12.85	13.34	10.13	8.13	9.44	11.32	13.72	13.56
7	0.97	4.07	6.75	5.75	6.55	7.64	7.77	9.57	10.94
8	0.99	10.71	10.17	9.25	8.90	11.41	12.93	11.11	11.58
9	0.99	13.15	13.25	13.40	19.93	10.02	9.10	10.95	12.18
10	0.98	9.80	9.46	8.49	9.84	8.24	12.20	8.63	6.90
11	1.01	12.54	14.10	17.20	11.95	13.76	14.04	11.27	10.97
12	0.98	15.49	14.64	17.59	10.50	14.18	13.78	10.27	11.69
13	0.85	8.41	7.62	4.37	7.18	10.79	10.10	9.91	11.49
14	0.67	3.94	3.92	6.07	4.40	13.52	7.66	10.57	8.79
15	0.97	4.60	3.15	7.48	9.33	8.57	5.50	10.41	14.94

16	0.45	4.58	8.48	7.45	10.84	16.09	14.46	12.67	21.25
17	0.43	8.87	4.95	21.20	14.27	21.02	12.34	22.39	17.31
18	0.78	2.72	3.51	4.09	4.70	7.81	10.36	13.02	13.65
19	0.61	3.79	2.93	5.57	9.12	12.82	11.23	13.54	18.69
20	0.89	3.29	2.44	6.76	8.66	11.89	10.37	11.72	10.10
21	0.90	5.83	3.41	2.10	3.11	15.94	11.45	8.91	12.40
22	0.44	1.18	1.61	2.66	2.09	6.02	9.37	11.55	13.22
23	0.35	1.60	1.45	2.39	3.40	7.66	4.94	9.83	8.17
Mitochondria									
Field 1									
1	0.83	3.32	3.93	1.09	2.83	5.94	5.14	3.78	6.55
2	0.97	5.13	5.26	8.32	4.73	5.49	4.29	8.27	7.20
3	0.97	9.65	9.33	2.84	5.09	7.71	9.73	2.26	4.14
4	1.02	6.34	7.83	7.41	9.16	6.70	4.36	3.89	9.47
5	1.00	5.46	3.29	3.51	6.21	4.51	2.11	3.61	6.37
6	0.96	5.51	1.93	2.59	4.21	9.13	3.84	2.97	6.05
7	0.69	1.38	3.10	5.01	2.59	1.20	2.95	7.60	5.12
8	1.01	4.30	3.15	2.66	6.59	5.72	2.84	3.35	10.20
9	1.01	6.40	3.46	4.80	6.78	2.21	2.00	1.07	2.15
10	0.91	5.32	7.63	5.18	2.90	3.63	2.19	1.60	1.53
11	0.91	7.28	4.34	10.13	3.96	3.23	0.69	3.08	0.80
12	0.76	2.90	5.35	5.19	1.77	5.20	6.48	8.95	4.02
13	0.97	4.53	4.49	2.75	2.53	3.62	3.77	1.85	2.40
14	0.90	7.51	3.10	6.42	6.50	4.59	1.69	4.66	5.41
15	0.76	2.57	2.70	2.86	3.50	2.52	3.27	3.32	4.19
16	0.93	2.32	0.58	1.47	1.80	4.25	2.30	4.24	3.83
17	0.79	4.44	2.63	3.07	2.53	4.65	3.15	3.32	3.00
18	1.00	7.03	4.53	6.04	8.06	6.58	4.18	5.66	7.68
19	1.03	3.81	5.84	5.11	4.55	3.54	4.28	6.32	4.14
20	0.99	7.80	7.03	8.50	6.26	3.48	3.48	4.59	3.50
21	0.97	6.64	3.91	2.63	3.21	6.26	5.51	5.23	6.35
22	0.99	7.09	7.48	3.85	4.80	3.13	4.88	2.33	4.15
23	1.00	7.86	7.35	2.28	4.00	5.94	5.41	2.30	6.99
24	0.97	6.63	3.57	1.71	1.14	5.37	2.08	1.39	0.70
25	0.97	4.75	4.81	3.45	2.66	5.02	5.82	2.91	1.28
26	0.81	1.32	1.47	2.91	1.95	2.58	1.72	4.49	4.79
27	1.00	2.61	3.30	3.36	3.13	4.54	5.58	5.27	5.43
28	0.99	8.93	9.32	2.64	1.79	7.93	8.76	2.36	2.45
29	0.96	5.76	2.10	2.99	6.22	7.38	1.62	2.51	5.14
Field 2									
1	0.92	0.37	0.92	1.02	0.81	1.69	4.95	8.08	5.10
2	0.65	1.29	1.03	0.51	0.49	2.94	2.58	3.58	4.57
3	0.90	4.13	6.07	6.01	8.18	7.27	10.24	6.52	7.12

4	0.39	0.90	0.90	0.57	0.64	3.39	2.81	3.27	5.32
5	0.67	4.34	2.82	2.17	2.22	0.00	0.00	0.01	0.00
6	1.00	4.33	5.75	6.36	1.82	1.27	1.92	3.22	1.88
7	0.92	5.34	4.54	3.25	5.84	1.35	3.64	1.48	2.28
8	0.99	9.39	7.68	3.40	6.67	3.57	4.11	2.36	1.98
9	0.95	4.93	4.32	6.75	6.93	3.81	1.88	3.16	4.72
10	0.98	7.35	10.75	4.74	3.92	5.64	8.60	5.24	4.01
11	0.98	7.98	8.36	8.64	13.03	4.22	2.75	4.59	7.12
12	0.87	3.16	5.73	6.06	7.16	1.82	2.53	2.75	2.91
13	0.71	0.69	0.65	1.32	1.59	3.21	2.96	4.83	8.07
14	0.86	0.44	0.24	0.36	1.07	5.38	4.72	4.76	5.20
15	0.96	8.50	5.52	6.98	11.24	7.89	8.29	7.83	10.08
16	1.01	8.77	9.74	8.78	7.19	6.98	6.77	6.11	6.83
17	0.80	3.57	4.90	2.08	1.35	8.51	10.64	4.78	4.87
18	0.56	1.38	1.42	1.22	0.89	8.31	9.30	8.00	6.83
19	0.64	1.55	2.42	2.29	2.03	8.05	8.40	9.80	8.41
Field 3									
1	1.01	2.19	2.78	1.43	0.79	14.42	13.30	6.58	7.87
2	0.96	0.09	1.19	2.20	0.62	5.81	8.57	13.21	10.28
3	0.99	3.60	1.05	1.94	2.07	11.15	8.32	14.34	15.17
4	0.98	1.33	0.87	1.05	2.24	14.46	11.45	7.43	11.16
5	0.53	2.08	0.18	1.11	1.30	7.67	2.12	5.03	6.43
6	0.64	0.63	1.58	1.11	0.81	2.33	4.78	5.42	3.12
7	0.99	2.38	3.83	3.21	2.56	6.06	5.96	9.92	8.23
8	0.99	1.85	4.47	3.54	2.45	6.07	10.44	8.83	7.07
9	0.63	0.33	0.34	1.59	0.18	13.79	7.98	15.50	7.59
10	0.39	0.36	0.35	0.11	0.21	11.49	8.27	4.13	12.57
11	0.98	0.64	0.28	0.14	0.30	8.75	6.33	5.55	6.31
12	0.83	0.02	0.10	0.00	0.01	1.97	2.38	1.47	1.62
13	0.77	0.34	0.04	0.01	0.00	5.15	3.09	1.97	0.95
14	0.97	5.00	1.24	1.91	2.76	5.30	3.00	5.50	5.31
15	0.97	3.32	4.09	2.78	5.89	1.53	5.40	4.66	2.05
16	0.50	1.96	1.08	0.04	0.76	4.49	4.17	1.38	6.11
17	0.41	0.02	0.00	0.00	0.01	9.09	1.71	1.76	5.75
18	0.43	0.07	0.00	0.00	0.17	9.68	0.25	1.34	9.01
19	0.96	0.83	0.13	0.28	0.44	3.17	1.68	3.64	4.30
20	0.80	0.43	0.47	0.58	1.06	13.39	10.01	11.75	13.13
21	0.17	0.01	0.15	0.06	0.08	1.77	14.10	4.33	10.38
Field 4									
1	0.74	0.06	0.17	0.09	0.03	2.77	1.94	1.19	1.12
2	0.21	0.58	0.45	0.11	1.37	2.95	1.68	0.74	5.34
3	0.59	2.20	0.62	0.69	0.76	1.64	0.75	3.38	2.63
4	0.95	3.82	3.87	4.11	4.40	5.31	2.67	3.84	5.40

5	0.56	0.50	1.67	0.52	0.57	2.01	4.22	3.12	1.23
6	0.99	1.15	2.50	1.12	0.98	2.62	7.11	4.08	1.72
7	0.71	0.68	2.46	3.98	0.89	1.77	7.31	6.73	0.69
8	0.91	0.89	0.17	0.38	1.79	4.63	1.18	0.80	1.79
9	0.91	0.96	1.58	1.00	1.42	1.11	0.67	2.25	3.33
10	1.00	2.33	2.23	2.55	1.54	3.50	2.91	1.75	2.55
11	0.99	3.85	3.22	3.27	3.60	1.45	1.65	1.16	0.90
12	0.94	4.32	4.65	3.63	3.67	2.28	2.29	7.10	4.82
13	0.86	0.24	0.12	1.53	1.16	2.52	1.56	1.58	1.88
14	0.96	2.62	1.71	1.50	2.13	0.93	0.92	2.26	2.76
15	0.95	1.63	1.45	1.29	1.18	3.43	4.04	2.02	0.62
16	1.00	3.81	2.90	1.10	4.22	2.34	0.86	0.63	3.22
17	0.94	0.58	0.93	0.50	0.42	1.14	2.07	0.83	0.66
18	1.00	1.94	1.10	1.94	1.40	6.39	3.88	5.47	6.52
19	0.35	1.72	3.71	1.97	0.53	1.02	3.37	2.29	0.41
Field 5									
1	0.99	12.28	8.26	7.03	8.59	14.51	7.64	7.52	9.51
2	1.00	3.46	4.22	3.76	2.60	4.59	10.10	8.15	3.01
3	0.56	0.02	0.09	1.16	1.51	1.91	4.34	5.60	10.18
4	1.02	4.26	3.10	2.01	4.77	5.85	2.14	5.53	9.30
5	0.84	6.20	6.85	5.43	4.72	13.57	8.20	8.38	10.60
6	0.92	4.64	6.11	2.35	3.00	4.00	6.12	1.14	3.78
7	0.99	5.93	4.70	5.56	4.19	3.54	5.16	5.93	3.98
8	0.90	2.05	1.17	1.17	2.38	5.73	8.39	3.89	7.74
9	0.95	1.89	1.91	2.30	1.68	3.69	3.55	6.47	7.54
10	1.03	7.83	4.40	5.36	6.80	10.03	8.93	7.57	4.50
11	0.99	5.41	7.86	5.89	6.42	3.98	5.11	12.15	8.97
12	0.64	0.26	0.15	0.41	0.22	2.90	3.44	4.92	3.97
13	0.69	2.64	1.98	0.28	0.67	5.71	7.90	2.78	7.38
14	0.96	2.96	4.45	3.77	4.33	0.97	1.98	1.45	1.34
15	0.28	3.89	2.72	1.20	0.48	5.19	8.93	4.22	0.91
16	0.98	10.42	5.02	3.52	3.08	11.99	9.08	3.14	2.87
17	0.95	4.99	4.92	3.29	3.40	4.54	4.28	3.21	3.57
18	1.02	7.41	5.64	4.39	3.89	7.14	7.24	5.51	4.33
19	1.02	14.56	17.65	15.90	10.10	8.96	9.15	7.24	8.51
20	0.98	9.88	17.47	12.64	10.80	6.24	12.14	11.04	7.12
21	0.36	1.91	0.29	1.60	1.68	5.55	1.59	3.87	3.17
Endoplasmic Reticulum									
Field 1									
1	0.88	2.19	2.48	2.49	2.18	3.92	4.07	3.74	3.65
2	0.22	1.72	1.80	1.81	1.21	3.50	3.44	3.54	3.23
3	0.29	1.23	1.70	1.21	0.89	3.34	5.06	4.23	2.69
4	1.00	2.96	3.40	4.32	2.34	3.90	4.04	4.82	2.81

5	0.99	3.70	3.30	3.28	3.76	5.61	5.41	4.63	4.68
6	0.27	2.31	0.63	0.96	2.19	3.78	1.93	3.41	4.43
7	0.21	1.38	2.46	1.13	1.57	2.82	4.05	2.98	3.07
8	1.01	4.68	5.46	6.30	4.48	4.62	6.63	6.55	3.76
9	0.99	2.71	2.21	1.97	1.99	7.19	5.06	5.15	6.23
10	0.17	2.63	0.69	0.57	1.18	4.60	2.14	1.61	2.62
11	0.26	1.36	0.92	1.33	0.88	4.04	3.60	4.58	4.16
12	0.37	1.68	1.01	1.20	1.12	4.95	2.47	3.90	3.90
13	0.30	1.93	2.98	1.38	2.87	3.50	4.15	3.41	6.23
14	0.78	1.07	1.07	1.04	1.30	3.72	2.91	2.46	4.64
15	0.96	3.57	2.21	2.59	4.15	5.41	5.25	4.10	4.38
Field 2									
1	1.01	4.47	3.98	5.12	5.66	3.54	2.68	2.87	3.44
2	1.01	5.99	5.63	3.45	3.08	3.14	3.61	3.64	3.22
3	0.98	3.96	6.59	4.08	3.73	2.51	3.55	2.78	2.01
4	0.80	4.21	4.15	3.98	3.06	3.17	3.12	3.19	2.87
5	0.80	3.27	3.30	3.05	3.51	3.29	3.82	4.12	3.81
6	0.97	6.83	6.71	6.79	4.78	2.60	2.65	3.75	2.44
7	0.97	5.51	5.71	3.64	3.09	5.04	4.30	3.14	3.17
8	0.99	4.32	9.28	6.94	3.91	3.44	4.20	5.22	4.92
9	0.98	4.87	7.10	8.05	8.11	3.93	4.38	3.37	4.17
10	0.84	3.88	5.05	5.18	5.49	3.30	3.61	4.92	4.70
11	0.97	2.62	3.20	4.55	4.51	4.27	5.56	5.95	4.90
12	0.98	2.48	4.02	3.01	2.00	3.40	4.01	4.84	4.32
13	0.97	3.17	4.35	6.16	5.01	3.45	3.21	4.43	3.22
14	0.63	2.50	1.99	1.13	1.99	4.14	4.21	3.99	4.05
15	0.26	1.07	0.39	1.41	2.09	4.26	2.76	4.29	4.71
16	0.96	1.41	2.79	2.85	2.78	2.35	4.09	4.98	4.23
17	0.89	2.55	2.49	1.97	2.13	4.16	4.48	3.29	2.98
18	0.90	4.14	4.38	5.97	4.02	4.84	4.31	4.07	4.62
19	0.90	7.35	4.25	2.96	6.63	4.96	4.82	4.24	4.35
20	0.78	1.22	2.31	2.43	1.56	3.10	4.23	4.73	3.50
21	0.75	2.79	2.12	2.75	3.08	4.37	4.81	4.97	5.87
22	0.33	0.85	1.31	0.80	0.71	5.26	5.37	3.75	4.31
23	0.84	1.73	2.37	1.45	2.07	5.56	6.94	5.63	5.93
24	0.56	0.60	0.36	1.40	1.61	5.18	3.40	5.50	5.88
25	0.98	9.38	7.08	2.36	5.12	4.98	6.07	2.57	3.83
26	0.99	4.40	4.49	1.47	3.40	6.02	6.63	4.48	6.60
Field 3									
1	0.88	4.33	2.17	3.17	5.10	5.50	3.16	6.16	5.33
2	0.97	5.06	6.38	7.66	8.90	5.62	5.26	5.65	7.32
3	0.92	6.24	11.53	7.60	5.01	5.10	6.49	4.78	5.48
4	0.91	9.07	10.19	7.62	6.16	6.21	6.17	6.02	8.07

5	1.00	7.50	7.45	6.30	6.78	6.46	6.53	5.13	5.96
6	0.95	9.63	10.54	10.60	8.42	5.43	4.84	4.97	6.31
7	0.98	4.15	10.74	7.95	4.34	3.79	7.71	8.93	5.17
8	0.97	5.45	7.59	4.43	3.52	8.24	7.91	5.21	6.27
9	0.95	5.60	3.38	2.28	2.94	11.73	7.22	4.57	9.27
10	1.00	7.96	5.52	8.17	5.75	8.99	7.05	8.82	8.91
11	0.75	4.23	3.35	3.63	3.11	6.99	6.75	5.89	5.57
12	0.82	1.93	2.96	2.55	1.48	5.57	7.46	6.75	6.10
13	0.51	3.42	1.00	4.51	2.84	7.45	4.83	13.16	10.05
14	0.96	3.67	3.59	4.64	3.75	9.50	8.81	10.94	10.00
15	0.45	2.22	1.64	4.13	2.09	7.17	6.10	9.95	7.79
16	0.72	4.51	3.11	4.52	4.00	8.08	5.60	5.69	6.41
17	1.01	3.09	7.37	3.88	4.33	4.88	7.00	4.82	7.32
18	0.95	5.98	6.22	5.95	8.22	7.08	6.76	9.15	7.98
19	0.89	5.13	8.75	8.15	5.52	5.38	6.72	6.95	4.26
20	0.37	7.19	7.35	1.60	6.11	7.97	7.81	2.76	7.80
21	0.85	3.27	2.95	3.11	3.56	7.69	6.62	6.20	6.74
22	0.88	2.98	3.39	2.27	2.55	6.80	7.43	7.13	10.82
23	0.98	5.55	2.82	3.52	2.97	9.66	6.89	10.77	9.18
24	0.99	4.66	5.79	5.33	5.51	6.28	6.47	8.22	9.70
Field 4									
1	0.95	2.69	3.39	6.15	3.35	6.29	5.93	5.62	5.96
2	0.93	7.13	4.57	4.07	3.51	7.72	5.21	4.17	4.52
3	0.98	5.25	8.24	10.05	8.62	5.28	9.16	9.77	7.12
4	0.96	9.30	7.92	4.82	7.94	10.03	7.84	4.93	6.86
5	1.00	4.64	4.10	4.69	5.94	6.08	6.62	8.13	6.39
6	0.92	4.64	4.33	8.33	4.86	7.39	5.05	8.40	8.40
7	0.97	8.32	9.26	6.72	8.02	8.52	9.04	5.65	6.73
8	0.93	8.02	10.19	8.28	6.60	6.05	6.61	7.77	4.86
9	0.87	2.67	3.56	3.25	3.47	7.44	7.59	6.72	6.56
10	0.55	2.60	3.09	2.93	3.25	7.76	6.55	6.63	8.22
11	0.99	9.72	8.31	10.53	12.24	8.83	6.44	9.48	10.89
12	0.91	6.89	6.54	7.25	5.26	7.01	9.30	6.70	4.85
13	0.98	3.30	5.09	7.63	7.12	4.10	7.55	9.12	8.85
14	0.97	3.03	6.22	5.72	3.88	5.03	7.78	9.51	7.61
15	0.98	6.56	4.45	6.11	6.98	4.61	4.18	6.67	7.28
16	0.59	9.25	3.60	4.76	9.58	7.55	4.39	4.76	7.80
Field 5									
1	0.96	5.68	7.00	4.50	4.71	13.10	10.18	7.97	13.02
2	0.71	1.46	3.22	3.11	2.66	5.99	8.37	11.16	9.69
3	0.83	6.31	3.11	3.51	5.12	10.66	7.28	9.32	9.74
4	0.94	8.00	4.69	5.56	5.56	8.44	7.42	12.54	8.78
5	0.87	3.65	6.78	7.34	2.14	8.88	7.84	10.35	7.74

6	0.93	9.52	14.15	9.79	7.82	8.99	12.49	14.58	11.46
7	0.97	4.55	7.72	5.58	6.34	8.16	8.35	10.70	11.02
8	0.99	15.67	14.11	12.67	12.32	11.52	11.75	10.56	9.06
9	0.99	13.29	12.47	17.81	18.75	12.27	9.12	13.55	13.56
10	0.98	14.78	11.53	11.80	16.89	8.11	12.50	10.05	8.93
11	1.01	10.11	13.10	16.72	10.63	11.93	11.71	10.37	10.40
12	0.98	12.93	11.63	11.65	10.33	12.87	9.43	7.84	11.57
13	0.85	10.59	9.30	3.88	6.96	10.87	10.37	9.26	11.70
14	0.67	4.24	3.77	6.49	4.98	11.10	6.86	11.45	11.23
15	0.97	4.54	2.81	5.86	7.08	8.23	5.51	9.89	12.95
16	0.45	2.87	5.95	4.73	7.13	9.65	7.89	8.77	16.30
17	0.43	5.32	2.37	13.57	11.36	14.07	6.06	15.91	13.61
18	0.78	2.24	2.73	2.95	3.97	8.81	8.39	12.90	13.89
19	0.61	2.36	2.04	3.99	7.93	8.86	9.22	12.32	14.84
20	0.89	2.28	1.48	6.71	7.86	10.67	8.45	12.27	8.55
21	0.90	4.85	2.52	1.23	2.99	14.21	9.13	6.74	11.70
22	0.44	0.20	0.91	2.58	1.59	4.30	7.37	12.73	13.96
23	0.35	0.76	0.52	1.65	3.08	8.08	4.13	9.84	11.12
Lysosome									
Field 1									
1	0.46	0.02	0.19	0.14	0.19	0.71	2.14	1.61	1.11
2	1.00	1.08	2.39	1.43	0.77	7.77	15.54	3.91	1.70
3	0.98	0.47	0.50	0.48	0.16	4.57	5.11	5.09	2.94
4	1.02	2.15	1.00	0.81	1.69	7.77	4.51	3.22	3.26
5	0.92	0.33	0.51	0.10	0.03	3.18	4.20	3.01	0.49
6	0.98	1.23	1.22	0.43	0.58	5.10	2.70	1.92	2.24
7	0.99	0.21	0.54	1.36	0.57	1.61	5.98	5.02	2.86
8	0.69	0.13	0.17	0.27	0.96	7.36	7.87	1.88	9.12
9	0.43	0.04	0.07	0.00	0.00	1.78	6.12	3.28	4.30
10	0.20	0.00	0.00	0.00	0.00	2.27	3.79	1.36	1.82
11	0.99	1.70	1.87	1.14	0.73	0.60	1.47	3.81	1.12
12	0.95	0.63	1.44	0.79	1.08	0.79	2.73	0.54	0.92
13	0.98	1.77	4.85	2.15	0.56	2.84	3.70	6.61	5.16
14	0.94	2.28	0.76	4.14	1.83	1.38	2.89	3.90	1.73
15	0.96	0.09	0.04	0.11	0.89	4.24	1.55	2.43	4.14
16	0.96	0.11	0.94	0.09	0.07	3.40	3.22	4.42	3.44
17	0.91	2.04	0.23	0.09	2.30	7.60	1.77	1.82	3.12
18	1.01	5.52	4.84	3.26	5.62	0.85	1.30	0.89	3.47
19	1.00	4.08	4.21	4.77	6.93	3.32	2.24	3.06	4.92
20	1.01	0.85	2.35	1.12	1.20	1.87	4.97	3.24	3.56
Field 2									
1	0.36	0.48	1.48	0.69	0.50	1.81	3.54	3.55	1.33
2	0.45	0.91	1.53	1.20	2.45	3.52	5.07	6.58	9.39

3	0.96	2.11	0.62	3.90	4.70	2.82	0.07	5.30	8.29
4	0.98	1.25	1.42	0.54	0.48	1.83	2.16	0.50	1.42
5	0.93	5.63	5.65	2.96	6.02	6.32	5.99	2.28	3.95
6	1.00	4.54	5.07	1.82	0.70	10.12	6.28	4.43	1.02
7	0.99	1.20	2.84	1.81	0.73	2.54	1.77	1.73	2.72
8	0.97	3.11	1.76	2.86	2.85	1.98	3.55	3.92	1.87
9	0.90	7.27	4.02	1.33	3.77	2.52	2.36	0.14	1.88
10	0.65	0.96	0.58	0.01	0.14	2.57	1.83	0.59	1.29
11	0.71	1.66	1.08	0.48	0.05	1.11	0.84	0.61	0.16
12	0.53	2.11	3.87	3.41	1.16	5.00	7.17	9.09	3.12
13	0.83	3.01	3.45	4.01	6.62	0.91	3.22	7.95	11.13
14	0.77	1.36	2.33	0.81	5.51	1.90	4.96	1.06	5.16
Field 3									
1	0.99	0.00	0.06	0.09	0.02	0.73	2.03	0.92	0.38
2	0.99	0.34	0.30	0.54	0.28	0.81	1.17	2.43	0.58
3	1.00	1.62	0.75	0.01	0.73	3.69	2.90	0.11	2.38
4	0.51	0.03	0.31	0.29	0.16	0.19	0.65	0.66	1.86
5	0.96	1.72	0.78	0.91	1.60	5.36	5.97	7.21	8.37
6	1.00	0.00	0.29	0.10	0.01	0.16	0.71	0.47	0.34
7	0.98	0.66	0.74	0.61	2.14	0.75	3.29	2.92	4.84
8	0.96	0.40	0.42	0.20	0.47	2.73	3.78	3.70	2.20
9	0.46	0.01	0.03	0.03	0.13	1.00	0.41	1.00	0.37
10	1.00	1.54	1.47	0.83	0.78	1.76	1.88	1.58	0.68
11	0.95	0.00	0.00	0.00	0.00	0.37	0.38	0.26	0.09
12	0.46	0.00	0.00	0.00	0.00	0.02	0.09	0.11	1.67
13	0.54	0.09	0.05	0.02	0.07	3.95	3.83	2.52	5.63
14	1.00	0.08	0.17	0.45	0.70	0.99	0.48	0.47	1.68
15	0.97	0.38	0.07	0.87	0.89	2.30	0.42	2.06	1.71
16	0.73	0.00	0.01	0.01	0.04	0.28	0.15	0.19	0.37
17	0.45	0.00	0.00	0.00	0.00	0.51	0.30	0.08	0.03
18	0.88	0.00	0.00	0.49	0.11	1.08	1.41	5.01	2.06
19	0.97	0.00	0.03	0.01	0.00	0.37	3.36	3.81	1.14
20	0.76	0.23	0.11	0.02	0.13	3.30	1.53	0.36	0.49
21	1.02	0.60	1.49	3.49	1.67	1.43	1.54	5.60	5.47
Field 4									
1	0.56	1.52	2.09	5.61	3.25	5.52	4.78	4.72	6.20
2	0.94	4.61	1.15	3.87	12.46	2.15	0.18	2.54	3.80
3	1.02	8.80	4.88	3.35	9.93	9.77	5.51	4.78	11.67
4	0.99	2.98	2.22	4.73	5.96	2.04	1.54	1.30	2.03
5	0.67	0.60	0.26	0.10	0.55	3.43	3.39	2.20	10.26
6	0.60	0.28	0.37	0.46	0.21	2.99	1.84	1.67	1.88
7	0.37	2.16	0.75	1.36	3.85	3.22	1.50	2.50	3.37
8	0.95	3.31	1.43	5.21	5.55	4.49	1.65	1.58	6.74

9	1.01	2.07	2.04	3.69	2.19	4.39	4.87	5.16	5.25
10	1.00	8.90	12.23	4.19	4.21	0.56	3.29	0.28	0.24
11	0.97	7.81	2.41	3.50	8.75	1.26	2.12	2.31	0.66
12	1.00	8.60	3.61	5.85	9.09	6.89	2.40	2.48	7.30
13	1.00	4.73	4.31	3.89	6.65	2.38	2.25	3.89	3.89
14	0.45	0.19	0.06	1.43	1.02	3.15	5.14	4.10	4.50
15	0.67	6.13	7.88	7.31	1.78	6.34	11.28	6.27	2.40
16	0.99	2.68	4.81	12.97	10.65	2.37	2.58	13.88	13.44
17	0.32	4.03	2.07	4.99	3.60	13.98	6.20	12.41	11.13
18	0.79	8.79	2.85	2.94	6.23	6.11	3.44	1.87	14.17
19	0.99	5.47	2.41	4.38	8.70	4.90	1.99	1.64	6.70
20	0.27	5.36	1.25	1.20	6.26	10.45	3.33	3.96	12.84
21	0.57	6.10	6.86	7.47	2.15	4.27	8.87	4.12	5.89
22	1.01	8.71	7.62	6.88	5.47	4.21	4.01	5.22	1.13
23	1.01	4.24	6.20	7.82	4.85	0.49	2.41	7.08	1.71
24	0.57	1.46	1.45	1.39	1.24	2.99	3.74	3.27	2.56
25	0.56	3.74	2.32	0.90	1.31	2.94	3.77	0.93	1.04
26	0.35	0.13	0.42	0.30	0.23	1.26	1.99	3.02	3.69
27	0.96	3.89	2.47	1.21	2.50	6.60	2.87	3.32	6.21
28	0.78	1.23	1.28	2.85	2.07	1.77	1.80	2.00	2.14
29	1.03	5.16	1.80	3.56	6.02	1.25	1.05	1.73	1.89
30	0.95	9.22	1.18	0.43	10.20	5.24	2.65	2.46	5.11
31	0.98	4.13	16.40	10.57	6.13	0.74	2.39	3.74	2.22
32	1.01	5.17	6.88	13.47	12.52	3.93	3.52	2.87	2.31
33	0.57	0.39	1.23	0.03	0.04	4.96	8.89	3.19	1.13
34	1.03	3.29	7.53	4.87	2.67	2.37	2.51	2.94	1.81
35	0.99	6.76	1.79	0.40	6.15	8.10	3.05	0.97	4.80
36	0.43	5.34	1.92	0.00	0.31	2.95	1.18	0.08	2.43
Actin									
Field 1									
1	0.92	12.17	8.25	9.00	8.71	9.44	15.95	22.84	10.95
2	0.98	18.88	11.10	19.85	44.36	22.30	18.37	19.25	32.68
3	1.00	8.34	7.85	5.55	7.05	12.38	12.29	15.18	16.18
4	0.45	4.46	3.52	3.82	2.54	10.71	18.08	14.82	9.08
5	0.44	3.18	5.32	4.59	9.18	10.40	9.65	10.28	10.93
6	0.71	13.52	11.30	6.22	4.55	18.74	12.69	16.85	9.93
7	0.93	5.77	9.25	5.43	3.89	8.81	17.13	7.03	9.84
8	0.94	14.91	3.97	3.10	5.60	25.24	11.16	8.51	8.50
9	0.36	7.02	1.92	5.52	9.77	11.89	11.37	13.04	14.84
10	1.01	6.29	3.46	4.22	9.54	26.35	17.00	12.02	21.83
11	0.49	1.40	1.68	2.61	3.60	8.54	12.80	16.12	20.45
12	0.80	11.03	5.61	5.95	9.41	20.51	14.27	15.53	14.53
13	0.47	3.86	2.74	2.74	1.69	15.52	17.50	13.16	15.04

14	1.02	12.54	16.13	16.07	10.35	20.85	25.93	23.76	16.34
15	1.00	12.35	16.35	7.60	14.45	14.97	12.58	10.41	16.54
16	0.99	13.87	15.12	9.41	15.82	16.61	14.81	13.50	15.06
17	0.96	5.87	5.07	7.88	12.58	10.88	11.17	11.30	8.60
Field 2									
1	0.78	3.59	6.86	7.90	8.25	19.99	20.56	13.85	13.22
2	0.49	7.14	6.81	2.44	4.95	15.04	10.60	13.07	22.26
3	0.66	5.10	7.12	6.72	8.65	10.45	17.80	17.31	13.16
4	0.70	7.19	8.32	7.46	9.66	14.77	17.95	17.73	19.57
5	0.93	7.61	8.37	12.84	12.45	14.98	14.30	15.32	14.84
6	0.98	13.41	19.51	12.19	17.98	12.21	12.70	13.00	13.20
7	0.93	10.53	7.17	8.01	15.27	16.71	11.83	12.41	17.73
8	0.97	17.87	14.21	12.50	12.41	10.95	13.97	10.52	9.68
9	0.92	10.06	10.25	15.51	8.61	13.33	16.26	16.04	11.05
10	0.96	14.26	13.34	17.27	12.36	15.17	10.61	11.97	11.96
11	0.97	21.20	16.63	16.77	20.09	10.21	12.08	20.18	17.24
12	1.01	14.36	16.60	14.27	11.71	21.76	27.95	18.46	12.26
13	0.83	9.49	14.10	12.44	9.09	21.56	18.46	19.29	21.54
14	0.96	17.57	12.58	14.59	15.83	14.79	16.91	15.54	12.50
15	0.98	11.62	13.79	12.79	22.01	13.99	15.85	15.07	19.01
16	0.99	7.65	9.80	8.75	6.76	17.99	13.39	10.94	19.34
17	0.94	12.33	7.09	5.45	9.01	13.21	17.01	14.62	10.70
18	1.01	6.52	12.46	16.43	13.39	11.09	12.78	11.08	14.42
19	0.95	14.42	8.59	13.57	14.21	8.66	15.16	20.18	10.22
20	0.93	3.85	4.48	14.49	6.18	9.43	9.27	20.50	18.05
21	0.96	14.08	8.90	11.83	18.63	21.60	14.53	12.01	12.13
22	0.98	3.29	2.37	2.51	5.60	9.04	9.43	4.63	8.00
23	0.78	15.59	22.35	6.89	7.94	13.27	16.55	11.50	14.57
24	0.99	35.20	39.60	27.99	25.18	27.28	29.83	31.17	24.10
25	0.81	2.81	5.42	5.61	2.76	6.71	8.22	13.54	9.91
26	0.38	1.59	2.20	3.33	3.65	7.36	8.70	14.09	15.31
27	0.29	1.41	3.57	1.72	4.37	10.33	15.43	13.38	12.80
28	0.29	1.04	4.34	3.12	3.12	7.81	15.94	11.29	15.27
29	0.97	6.18	9.65	11.94	9.89	18.64	17.10	15.34	19.72
30	0.90	14.11	9.89	8.34	8.03	15.05	19.68	15.60	17.25
Field 3									
1	1.00	19.69	17.22	11.70	18.63	18.02	19.81	16.04	12.75
2	0.44	5.87	13.31	10.42	11.93	12.05	14.74	16.16	18.79
3	0.87	13.10	12.30	16.69	18.08	23.19	25.53	35.04	29.61
4	0.89	16.77	12.45	16.14	14.76	14.39	22.87	29.65	21.21
5	0.82	22.33	19.52	19.76	12.04	13.14	8.84	12.66	19.18
6	0.48	12.91	13.62	13.97	14.73	11.65	14.28	12.76	11.32
7	0.53	12.79	17.80	11.15	12.74	14.28	17.22	14.13	8.09

8	0.92	4.62	6.25	6.67	5.26	11.74	25.30	34.32	22.75
9	0.97	5.37	4.10	2.60	4.83	26.39	9.33	12.92	25.27
10	0.30	7.32	12.68	9.40	15.61	15.36	22.04	12.67	21.53
11	0.73	16.84	8.44	5.83	13.07	26.48	16.10	8.70	10.11
12	0.97	13.25	11.79	10.84	14.67	16.04	22.56	14.48	14.50
13	0.92	8.43	11.55	6.47	7.72	11.69	29.79	16.50	19.08
14	0.70	5.85	9.40	9.18	7.72	13.24	19.86	18.30	23.04
15	0.98	15.05	17.74	30.31	25.62	15.15	15.82	18.25	18.04
16	0.97	20.53	16.89	23.60	23.75	10.73	10.94	9.73	10.09
17	0.19	3.34	4.41	5.85	16.76	16.15	12.13	13.58	18.52
18	0.90	17.65	23.67	21.84	23.52	20.42	22.72	17.98	23.61
19	0.97	12.92	16.56	15.90	13.84	34.55	12.77	11.68	30.09
20	0.99	23.88	25.18	28.34	20.07	9.63	9.18	9.94	10.24
21	0.82	14.69	18.00	28.36	29.07	13.82	23.03	13.15	10.19
22	0.23	2.61	2.73	7.46	5.12	7.98	11.84	10.54	18.19
23	0.61	9.34	13.29	12.46	8.34	12.90	16.28	8.02	5.90
Field 4									
1	0.62	5.44	10.27	9.29	10.14	15.69	12.69	14.18	21.09
2	0.35	4.32	3.95	0.68	1.25	18.38	11.22	9.53	12.18
3	0.74	6.02	6.54	4.14	8.56	22.38	13.54	12.23	17.89
4	0.90	10.92	9.99	8.37	14.00	18.30	17.28	20.53	18.47
5	0.99	30.78	17.05	19.73	32.74	23.60	13.07	13.76	24.44
6	0.75	28.13	28.10	15.35	31.74	19.31	19.06	13.68	22.01
7	1.02	21.77	20.43	21.54	17.67	29.05	30.28	29.22	27.21
8	1.01	23.97	29.24	32.83	23.89	21.26	20.93	26.31	25.24
9	0.94	25.50	19.51	20.41	11.58	20.19	22.58	13.90	11.07
10	0.95	23.24	29.73	32.01	18.35	16.55	17.74	23.82	16.20
11	0.99	34.54	26.07	5.63	9.97	11.10	12.16	20.22	12.59
12	0.98	19.08	18.40	33.01	29.68	14.38	17.23	18.61	16.98
Field 5									
1	0.96	4.24	3.35	4.48	5.86	16.36	9.15	8.71	9.59
2	0.58	8.06	5.75	4.47	9.82	14.72	5.10	9.57	18.33
3	0.99	20.70	18.92	19.78	22.63	10.27	9.98	13.47	13.51
4	0.92	14.40	11.90	14.19	12.00	12.22	7.15	10.71	7.91
5	0.97	11.02	7.22	8.34	9.07	8.56	7.10	4.67	5.50
6	0.99	12.87	13.14	17.09	12.60	6.68	6.06	13.97	5.81
7	0.89	18.44	11.04	10.29	9.38	17.49	7.84	7.27	6.86
8	1.01	13.09	11.17	10.64	11.94	9.60	7.56	6.74	7.47
9	1.01	16.38	10.17	11.08	15.26	8.78	5.82	6.37	10.60
10	0.96	10.08	11.36	18.46	15.15	6.06	8.49	10.21	8.21
11	0.95	11.13	16.43	11.48	11.46	6.78	7.97	7.69	7.53
12	0.99	14.04	16.19	15.59	15.25	7.11	7.01	8.87	5.62
13	0.77	7.42	13.10	12.33	7.62	8.15	9.36	9.73	8.99

14	0.99	10.09	10.13	16.72	8.72	7.36	7.07	6.16	4.82
15	0.91	5.73	9.43	11.45	9.10	4.59	12.87	12.03	5.48
16	0.95	15.51	15.20	24.83	12.97	17.72	5.59	7.60	9.04
17	0.95	13.02	9.00	8.95	12.44	9.90	9.15	11.66	9.62
18	0.76	9.30	14.33	8.34	6.10	6.43	12.18	8.84	6.71
19	0.89	9.09	6.40	6.73	9.58	8.10	9.32	14.58	10.19
20	0.86	8.81	7.87	5.78	8.81	14.23	10.60	13.76	7.97
21	0.96	10.64	9.00	9.23	13.66	7.62	5.05	8.85	6.45
22	0.79	9.80	11.47	8.33	10.43	5.77	5.87	5.29	6.55
Tubulin									
Field 1									
1	1.01	2.74	3.18	5.04	3.77	3.27	4.78	4.80	3.72
2	0.42	2.47	3.71	2.05	1.97	4.75	5.25	2.50	2.57
3	0.99	2.87	3.64	2.63	2.55	4.44	2.66	2.82	3.50
4	0.68	2.26	2.18	2.59	2.08	3.53	3.40	2.51	5.45
5	0.92	2.13	2.01	2.44	2.58	2.82	2.95	2.47	2.02
6	1.00	2.02	2.21	2.00	2.07	3.76	2.59	1.86	3.21
7	0.98	1.88	1.86	1.93	1.94	2.76	2.19	2.01	3.16
8	1.01	1.95	2.49	2.12	2.12	2.76	3.71	3.36	2.58
9	0.94	1.42	1.78	2.05	1.98	1.46	2.34	2.71	2.41
10	0.94	2.14	2.24	2.96	2.31	2.02	2.03	3.18	1.90
11	0.75	2.80	1.91	2.58	1.91	3.35	4.96	10.17	2.89
12	0.35	1.41	1.79	1.69	1.77	2.03	3.11	2.43	2.76
13	0.99	2.29	2.67	6.17	3.34	2.49	4.86	5.54	3.15
14	0.99	1.86	2.74	2.33	2.14	2.51	2.75	3.50	3.57
15	1.00	1.92	1.94	2.65	2.46	2.12	2.60	2.18	2.62
16	0.40	28.83	3.08	4.11	4.37	24.37	2.64	2.90	4.25
17	0.98	2.93	3.24	4.86	4.36	2.77	3.70	5.11	3.43
18	0.34	3.07	2.78	2.24	2.01	3.81	4.36	3.19	2.30
19	0.99	3.96	3.10	2.72	4.05	3.28	3.65	3.73	4.21
20	0.85	2.37	2.87	3.81	2.86	1.86	2.82	4.03	2.31
21	0.47	2.07	1.74	1.75	3.88	3.66	1.99	1.92	4.82
22	0.95	3.47	5.08	3.21	2.26	4.52	3.87	3.66	3.49
Field 2									
1	0.91	1.79	1.58	1.66	1.71	2.11	1.84	6.32	1.96
2	0.94	1.77	2.04	2.52	2.04	2.93	3.89	2.39	1.44
3	1.00	1.39	1.85	2.75	1.85	1.37	1.57	2.53	1.67
4	0.93	1.73	2.64	3.36	3.05	1.93	1.26	1.19	1.93
5	0.99	2.80	1.66	3.11	7.07	2.39	1.44	2.35	5.68
6	0.97	5.98	2.55	2.01	3.40	2.05	1.29	1.76	2.68
7	0.70	1.57	1.52	2.00	5.23	1.60	1.32	2.07	3.12
8	0.53	2.55	2.57	2.69	1.37	3.45	1.76	2.46	2.64
9	0.99	1.96	2.26	2.13	1.80	1.69	1.51	1.55	1.31

10	0.88	2.44	2.21	1.78	2.20	2.88	2.66	1.60	1.77
11	1.01	2.42	2.91	3.14	1.94	1.88	2.62	2.77	1.50
12	0.64	1.83	1.75	1.41	2.94	2.66	4.26	4.49	4.75
13	0.52	4.45	1.84	1.22	3.76	3.50	3.30	1.29	1.58
14	0.94	1.73	1.52	1.57	1.20	2.14	2.91	2.15	1.56
15	0.99	1.39	2.12	1.82	1.63	2.36	3.43	2.07	1.56
16	0.77	1.15	2.24	3.15	1.69	1.79	2.00	2.59	2.83
17	0.56	8.61	15.67	1.01	1.13	3.26	4.17	1.43	1.51
18	0.95	1.97	2.24	3.38	4.13	2.34	2.81	1.78	4.24
19	0.60	1.38	4.98	1.36	1.63	1.46	3.96	1.76	2.15
20	0.97	1.44	3.72	2.28	1.23	2.11	3.37	2.69	1.36
21	0.56	2.62	3.18	3.81	2.69	2.05	1.72	2.45	2.13
22	0.94	2.70	2.05	1.53	1.67	2.37	4.48	1.71	1.79
23	0.87	1.46	2.19	5.04	2.82	2.87	1.98	2.17	4.05
24	0.77	1.45	1.60	1.62	1.42	1.24	1.14	1.46	1.56
25	0.61	2.30	2.36	26.24	8.21	2.59	1.19	3.27	3.62
26	0.97	17.47	11.57	2.13	2.54	14.19	7.39	1.31	1.39
27	0.93	1.59	4.41	3.74	1.92	1.45	2.67	1.92	1.41
Field 3									
1	0.95	3.79	4.75	4.87	3.82	3.83	5.02	6.82	6.59
2	0.84	3.85	7.89	36.18	7.38	4.37	6.02	28.86	6.17
3	0.98	9.29	32.48	8.69	10.33	10.92	13.70	4.78	6.78
4	0.71	3.01	8.50	5.86	4.75	2.82	5.57	3.67	4.20
5	0.98	5.18	4.97	2.76	2.28	4.25	4.35	2.80	2.17
6	0.91	2.64	4.60	2.25	3.76	2.21	3.31	1.79	3.78
7	0.76	1.83	4.35	32.13	1.96	2.42	3.73	9.22	2.02
8	0.35	1.04	2.65	2.37	1.22	1.53	3.86	2.52	2.39
9	0.74	4.50	2.20	2.30	3.60	3.55	2.40	2.20	3.43
10	1.01	2.50	7.80	6.67	2.19	2.73	5.28	3.73	1.85
11	1.00	3.19	3.48	7.74	6.01	1.92	1.46	2.71	3.05
12	0.29	6.17	4.57	6.91	3.57	6.54	4.16	5.90	8.21
13	0.98	5.31	14.02	5.20	3.85	5.43	8.24	5.61	4.54
14	1.02	45.29	64.59	68.88	57.08	59.29	56.80	89.16	65.37
15	0.95	2.49	2.88	6.89	4.84	2.45	2.63	8.20	6.46
16	0.96	19.19	2.89	2.56	4.22	10.61	2.51	1.72	3.05
17	0.69	4.05	9.48	5.41	5.38	2.67	7.03	4.46	1.98
18	0.59	1.28	1.41	23.62	21.64	2.33	2.00	7.73	8.01
19	0.95	11.72	3.98	2.43	3.19	9.65	2.56	3.39	7.21
20	0.77	1.90	2.72	3.34	2.58	3.21	2.34	1.94	2.64
21	0.99	61.59	53.88	47.54	39.40	94.17	69.59	63.91	66.42
22	0.98	8.62	3.13	4.22	9.12	5.36	3.03	3.99	6.34
23	0.95	2.12	2.74	2.65	3.05	3.51	5.05	3.98	3.01
24	0.76	2.00	2.10	1.64	1.99	4.28	8.32	2.35	3.11

25	0.49	1.72	1.76	2.28	1.57	2.93	2.51	5.12	3.30
26	0.73	2.40	47.46	22.68	3.12	4.03	46.08	20.99	7.42
27	0.80	1.41	3.30	1.58	1.46	2.10	7.47	2.07	2.94
Field 4									
1	0.97	0.49	0.37	0.41	1.35	2.11	1.11	3.57	4.74
2	0.66	6.68	0.68	0.55	12.85	28.23	2.96	1.62	7.81
3	0.85	0.67	2.63	0.90	0.72	0.75	1.19	2.09	1.63
4	1.03	0.47	0.55	0.62	0.37	1.15	1.62	2.03	0.75
5	1.02	0.47	1.15	5.08	0.89	0.98	1.52	1.94	3.13
6	1.01	0.59	0.94	0.82	0.54	1.78	1.84	2.59	1.36
7	0.97	1.60	0.89	0.82	0.59	3.75	1.39	1.14	0.68
8	0.66	0.44	0.28	0.47	0.98	1.26	0.72	1.92	1.80
9	0.39	1.26	0.29	0.44	3.28	7.01	0.74	1.08	10.92
10	0.99	0.52	1.31	1.08	0.49	0.76	3.56	2.32	1.24
11	0.63	0.41	0.81	1.11	1.30	1.00	0.90	3.20	2.28
12	0.62	1.85	0.35	0.28	4.30	4.25	1.11	1.06	8.83
13	0.34	0.25	0.29	0.35	21.95	0.89	1.31	5.12	22.84
14	0.38	1.98	0.32	4.50	3.35	5.02	0.89	3.62	2.89
15	0.97	2.19	2.24	2.22	1.96	1.87	1.93	3.18	2.79
16	0.98	0.87	0.70	1.97	9.29	8.88	1.43	2.43	3.45
17	0.84	0.48	6.80	6.27	0.67	0.68	6.89	13.59	1.41
18	0.92	0.89	1.29	4.58	15.02	2.14	1.58	7.18	8.81
19	1.03	0.60	0.48	0.52	1.62	0.97	1.64	1.06	3.05
20	0.52	0.51	0.75	0.99	0.32	1.61	1.18	2.00	0.97
21	1.01	0.36	0.70	4.16	1.30	0.54	1.38	2.85	2.25
22	1.00	0.97	0.78	0.50	1.43	1.60	1.73	1.19	0.79
23	0.94	0.81	0.44	0.87	2.14	4.83	1.64	2.92	12.79
24	0.62	0.47	2.12	1.02	0.58	1.09	4.91	4.83	1.54
25	0.96	11.22	9.13	8.78	7.19	6.21	4.95	7.55	12.99
26	0.84	3.68	7.10	0.51	0.44	19.67	33.23	1.61	0.99
27	0.98	0.96	1.45	1.51	1.25	1.67	2.70	2.23	1.53
28	0.91	0.89	0.50	1.12	0.72	2.01	1.42	4.42	3.48

Table S11: Raw APVs of DIC images corresponding to fluorescent images of organelles in hypotonically treated RAW264.7 cell population

	Octant 1	*Octant 2*	*Octant 3*	*Octant 4*	*Octant 5*	*Octant 6*	*Octant 7*	*Octant 8*
Field 1-Nucleus/Mitochondria								
1	170.89	162.31	170.62	167.54	159.74	161.03	164.60	161.11
2	165.23	165.47	154.13	166.47	158.92	159.49	146.20	152.37
3	167.15	164.50	153.38	154.15	157.59	162.10	141.24	136.17
4	173.36	160.51	147.26	154.49	161.08	141.34	135.28	141.21

5	160.84	149.15	142.41	154.10	145.24	144.93	132.40	145.50
6	170.26	163.63	154.16	164.69	165.32	159.75	150.79	161.85
7	168.32	168.56	162.62	165.54	164.74	163.49	154.25	162.02
8	156.64	158.48	153.18	162.17	138.36	143.04	136.90	143.54
9	142.93	153.10	152.22	149.55	130.19	147.86	147.94	142.73
10	151.73	147.27	154.67	152.63	154.04	131.43	146.79	148.09
11	143.61	155.72	158.15	148.65	128.11	142.43	144.36	136.52
12	154.67	157.31	159.65	158.07	154.09	150.14	154.01	153.13
13	151.79	153.77	155.52	152.60	147.17	155.16	147.35	147.54
14	161.66	158.59	156.79	154.24	157.01	150.12	149.18	151.14
15	130.88	144.62	143.28	132.18	128.59	140.91	141.07	125.11
16	113.39	119.22	121.69	115.15	110.09	117.86	118.21	108.16
17	133.60	150.41	148.60	141.11	131.73	145.81	148.02	138.33
18	146.10	152.23	147.04	153.47	132.40	132.49	136.96	145.95
19	167.01	161.04	161.97	159.47	144.61	150.89	149.82	153.01
20	148.46	144.02	154.81	153.68	146.88	138.00	147.56	141.45
21	151.91	146.74	153.54	151.17	144.77	133.62	143.20	140.82
22	155.45	151.45	152.94	159.45	145.96	134.94	150.28	153.64
23	151.03	152.48	158.32	162.89	148.77	140.27	148.26	141.48
24	132.62	134.54	146.14	142.73	130.52	131.33	132.23	142.07
25	130.22	138.37	142.46	132.06	119.61	138.43	124.77	126.22
26	151.84	149.86	146.89	155.46	149.35	146.67	142.40	149.71
27	152.34	148.46	142.10	151.82	144.29	133.92	136.53	143.25
28	146.03	127.87	125.02	143.55	126.11	114.15	119.87	141.33
29	137.05	121.52	129.58	143.12	132.10	119.99	125.58	130.46
Field 2								
1	177.10	177.37	172.56	171.90	165.93	163.03	157.50	160.97
2	163.50	175.68	167.24	161.07	159.93	162.17	161.52	157.82
3	163.27	165.97	160.96	152.86	159.04	154.20	144.49	149.71
4	162.91	154.29	163.06	154.97	155.93	161.07	158.11	154.09
5	146.62	150.95	149.06	146.44	143.80	147.84	145.37	145.64
6	143.68	148.92	156.91	146.28	132.02	145.24	138.38	141.46
7	136.06	144.87	146.67	140.36	129.79	138.02	144.98	129.76
8	129.54	140.52	141.42	133.21	129.79	133.56	131.83	132.90
9	128.25	139.77	137.62	130.31	133.94	132.95	124.52	123.95
10	125.46	135.69	136.85	128.94	116.08	126.32	124.12	128.52
11	129.05	137.27	140.74	131.39	123.67	133.45	134.72	129.71
12	115.50	116.40	120.65	113.80	115.97	121.03	118.07	109.71
13	165.70	163.40	161.57	165.67	162.65	162.51	159.15	159.13
14	168.82	165.85	164.58	163.76	165.95	164.08	162.48	161.94
15	154.99	158.68	159.76	159.44	147.61	152.16	156.07	155.01
16	156.30	158.34	155.50	151.51	141.52	142.64	151.01	147.86
17	152.45	159.53	159.19	155.97	148.53	149.81	155.51	154.67

18	151.52	145.46	149.91	154.95	147.31	143.62	146.71	153.18
19	147.10	138.21	141.15	144.52	143.06	136.79	137.77	144.14
Field 3								
1	166.04	179.73	165.53	159.05	160.75	171.22	162.20	157.27
2	166.83	172.23	160.65	167.85	162.48	165.92	158.98	167.47
3	153.84	155.76	143.52	137.89	153.18	152.44	141.06	135.46
4	178.77	182.56	163.10	156.37	167.93	168.86	158.38	155.07
5	140.78	158.52	156.29	147.31	139.59	155.69	153.96	145.95
6	161.94	163.72	162.90	156.65	161.66	160.18	165.54	159.20
7	144.59	152.59	162.23	149.90	141.73	145.96	155.06	143.96
8	136.17	151.94	152.24	142.28	132.66	142.62	149.06	141.36
9	154.40	160.67	159.48	155.69	149.53	157.72	156.47	155.42
10	145.62	142.37	150.40	148.93	134.86	134.78	147.46	150.91
11	137.47	141.14	139.53	143.88	134.38	139.44	143.27	145.11
12	167.71	162.93	162.19	166.58	165.40	156.90	159.32	164.41
13	175.70	168.38	165.15	167.02	169.06	164.74	166.53	168.71
14	152.08	146.11	142.46	155.74	155.58	135.53	133.62	158.61
15	149.30	131.78	132.95	145.86	150.67	128.14	122.65	151.27
16	159.00	153.52	161.52	157.17	153.37	148.44	158.44	154.77
17	159.85	138.61	134.63	151.76	150.16	135.55	135.07	147.30
18	158.34	136.80	117.02	145.45	149.57	138.88	128.45	143.57
19	164.06	151.44	143.38	152.56	162.86	150.08	147.08	153.84
20	163.80	159.45	152.31	162.80	159.78	158.12	155.60	159.09
21	171.76	187.74	159.17	162.61	171.89	166.07	157.94	167.22
Field 4								
1	168.57	162.61	159.59	164.90	164.45	159.81	158.16	164.97
2	152.15	141.00	144.19	144.60	146.08	136.94	140.99	132.70
3	168.17	173.98	166.02	172.00	160.65	161.33	155.98	163.11
4	165.75	172.80	154.96	154.28	155.12	157.50	142.61	136.29
5	173.19	172.51	159.10	163.74	156.50	146.46	146.02	152.52
6	172.11	175.98	166.43	168.73	171.64	155.68	151.52	167.53
7	175.23	178.77	176.05	174.21	172.44	159.01	163.12	168.33
8	175.71	174.33	173.72	165.04	154.50	165.24	161.56	153.87
9	160.81	181.47	171.03	163.73	156.80	163.79	166.26	161.03
10	151.58	153.04	155.22	156.32	143.66	146.42	151.64	145.77
11	122.40	136.97	139.87	126.21	117.95	126.95	134.90	124.08
12	149.77	149.34	158.08	158.85	146.28	154.06	156.57	142.04
13	156.75	167.67	163.11	160.59	156.10	160.74	159.63	153.02
14	151.15	156.19	156.05	158.46	149.52	142.61	149.37	148.14
15	159.32	154.43	156.17	160.00	150.04	151.38	146.12	157.64
16	149.13	139.62	147.21	151.96	142.51	135.57	141.22	142.37
17	155.20	147.57	154.62	156.90	151.98	139.36	152.15	155.43
18	141.33	132.00	133.41	144.03	122.85	127.54	130.03	128.17

19	166.71	163.12	153.10	166.61	166.49	153.27	150.72	166.43
Field 5								
1	174.38	169.67	160.21	165.58	152.67	160.31	145.84	151.54
2	168.30	168.15	166.80	173.16	155.74	162.54	160.69	160.23
3	174.13	176.71	177.66	173.94	171.56	172.08	169.91	167.96
4	168.80	175.73	171.78	167.96	152.24	162.53	159.65	144.56
5	168.57	171.56	176.92	163.78	154.81	162.95	164.45	154.27
6	168.64	167.83	157.44	162.56	148.67	157.67	160.54	166.95
7	164.27	167.64	169.49	173.00	147.18	156.54	152.76	154.54
8	167.89	175.48	164.10	174.55	166.40	165.69	164.99	165.98
9	172.62	163.61	165.21	173.93	169.73	167.49	161.21	157.87
10	175.03	172.87	169.99	177.89	160.55	156.00	149.17	155.06
11	169.11	171.50	172.44	164.58	147.80	151.73	160.81	147.46
12	150.65	164.33	158.95	163.27	158.63	162.09	157.76	160.60
13	174.78	171.69	165.72	169.83	162.40	161.29	165.41	165.91
14	147.60	144.73	142.44	147.67	142.16	140.87	140.99	144.78
15	142.37	158.49	150.66	151.15	133.30	136.61	154.47	149.33
16	138.12	149.33	150.14	148.32	111.57	123.99	141.65	132.35
17	149.92	154.66	161.85	152.69	143.39	151.80	157.05	144.64
18	135.23	151.27	152.23	143.60	115.59	141.64	143.24	134.61
19	156.78	150.69	140.37	155.46	131.81	137.73	129.96	140.43
20	154.22	140.60	140.08	151.68	129.84	125.36	131.52	144.27
21	123.49	141.73	136.02	133.58	123.21	138.93	140.46	127.46
Field 1-Cell Membrane/Endoplasmic Reticulum								
1	174.31	173.86	168.28	174.79	186.58	184.03	182.34	185.39
2	180.42	178.92	175.37	183.22	191.49	182.41	172.61	187.41
3	172.86	179.96	154.25	174.74	187.88	189.30	187.97	193.05
4	158.44	171.20	176.58	168.91	152.70	168.82	174.78	171.84
5	175.38	174.53	176.65	171.21	180.40	179.56	177.32	170.38
6	136.78	160.93	147.18	146.33	157.54	174.01	171.73	154.22
7	136.80	147.96	148.72	135.84	145.96	156.03	159.87	144.74
8	146.33	145.45	148.70	158.77	150.45	150.00	160.68	155.01
9	158.60	157.64	160.78	162.18	160.64	165.98	167.19	168.88
10	162.59	155.76	151.51	165.51	165.98	169.05	164.94	177.61
11	157.79	155.84	153.28	163.89	165.48	166.47	162.08	176.18
12	158.12	157.46	161.09	162.08	178.32	169.41	171.65	177.21
13	171.79	171.38	164.91	175.08	185.66	173.37	175.83	181.69
14	164.94	159.63	158.16	165.58	180.22	172.62	171.22	178.43
15	149.40	154.00	144.26	148.62	166.76	152.28	141.45	147.72
Field 2								
1	152.00	161.66	159.88	148.18	147.34	158.07	155.97	137.77
2	158.85	165.98	163.31	162.73	149.18	159.30	158.51	149.66
3	149.36	156.32	158.05	150.60	143.94	149.69	152.38	140.61

4	142.26	149.32	149.60	143.00	134.63	143.24	140.71	138.26
5	147.85	153.34	156.03	147.94	142.59	151.07	146.23	137.95
6	132.50	141.62	141.03	136.76	117.34	132.23	133.01	129.23
7	135.04	145.86	145.69	138.10	132.69	140.24	141.82	134.07
8	135.48	145.58	146.63	141.04	127.37	135.07	131.40	131.23
9	140.36	145.89	153.04	137.63	132.62	142.38	138.03	132.54
10	145.20	156.18	155.98	149.91	138.65	149.73	146.13	139.33
11	154.03	158.67	163.09	156.62	154.10	151.91	151.79	148.49
12	159.10	165.42	162.75	162.16	157.98	160.92	158.87	156.67
13	158.62	167.83	166.41	158.96	157.01	158.11	157.70	154.98
14	158.23	161.97	165.94	159.63	156.18	158.74	163.31	155.51
15	156.25	163.20	159.76	167.32	164.51	161.99	156.29	163.66
16	170.86	168.67	167.39	165.43	165.34	165.35	163.03	162.49
17	166.51	174.24	168.29	168.17	170.73	168.38	163.13	164.81
18	158.46	166.11	163.33	149.63	153.02	158.64	153.40	149.55
19	169.72	173.28	166.88	164.01	156.38	159.75	153.55	163.95
20	170.48	175.25	169.27	167.37	164.81	157.84	159.38	161.39
21	168.99	164.82	161.00	166.52	164.86	158.43	150.68	159.19
22	152.68	144.80	146.07	157.22	151.65	144.58	140.38	152.29
23	143.77	134.82	126.94	141.06	140.25	128.16	129.69	136.86
24	143.13	139.55	137.24	147.68	142.61	139.41	137.03	142.77
25	151.63	149.02	152.91	158.30	138.78	125.68	141.21	141.89
26	167.04	166.19	156.29	158.77	156.38	141.55	152.98	158.21
Field 3								
1	161.47	168.56	166.99	166.85	158.68	170.71	163.03	159.81
2	172.70	166.56	162.14	162.69	157.79	162.34	156.68	141.64
3	135.54	142.74	138.19	141.90	135.44	137.60	138.35	127.77
4	143.51	146.50	143.48	143.93	137.40	141.64	144.40	131.43
5	147.37	155.95	148.63	144.48	140.73	147.68	150.56	143.43
6	137.07	146.47	147.63	140.28	137.89	145.60	135.38	136.84
7	133.19	137.11	148.33	133.99	134.57	135.88	134.61	132.02
8	122.05	126.27	129.84	121.96	124.81	124.90	125.57	124.99
9	152.28	155.38	160.03	157.26	153.73	150.08	146.64	154.60
10	154.99	162.17	158.12	159.92	153.32	152.30	147.65	150.41
11	164.24	168.67	167.88	165.45	167.92	168.02	171.61	165.63
12	159.91	167.87	169.33	158.25	164.30	169.99	167.84	160.75
13	184.66	175.90	189.86	177.07	165.15	172.00	161.98	170.95
14	170.25	169.40	177.50	169.56	167.58	167.33	153.46	156.35
15	173.26	165.84	163.72	171.51	169.64	165.63	160.85	169.82
16	179.50	171.95	156.82	166.55	167.02	165.07	156.04	165.44
17	171.38	170.08	171.77	166.37	172.79	170.84	168.54	166.67
18	167.19	172.21	173.52	167.22	158.28	161.30	161.46	164.38
19	167.48	169.00	169.16	162.51	158.51	158.39	152.50	153.60

20	157.55	146.39	150.29	164.01	150.97	146.23	152.10	153.87
21	153.31	146.69	145.09	155.91	155.22	148.32	145.20	155.54
22	150.66	142.57	149.00	154.56	149.52	141.72	145.89	154.38
23	150.20	152.20	148.40	146.52	144.57	142.07	135.10	145.20
24	159.36	154.87	146.53	152.16	158.70	141.45	141.30	150.90
Field 4								
1	167.13	161.48	161.58	163.41	162.08	152.44	147.26	154.76
2	150.78	153.53	154.92	153.40	153.51	148.67	142.44	156.22
3	177.94	174.77	172.43	174.62	171.12	165.40	160.93	161.66
4	167.71	174.10	168.89	169.54	164.29	172.38	165.21	156.52
5	180.24	173.27	176.19	174.75	165.74	167.44	160.02	167.62
6	176.42	177.95	172.60	176.14	163.32	175.56	176.94	166.09
7	176.29	178.52	177.40	176.13	171.70	158.17	163.66	170.06
8	181.77	177.55	181.90	178.11	165.34	170.21	163.21	163.75
9	178.94	174.54	176.58	178.79	172.59	169.59	168.83	169.95
10	177.77	167.94	174.77	179.32	165.67	164.27	162.85	164.76
11	176.37	176.78	174.85	171.06	157.85	167.18	158.58	152.03
12	170.73	173.56	180.32	176.37	157.32	160.43	158.56	157.09
13	169.38	168.09	168.62	169.74	160.99	160.65	156.22	160.42
14	165.72	166.02	168.57	165.75	160.02	165.28	159.70	160.69
15	153.44	153.73	158.54	155.55	142.68	149.84	145.72	144.28
16	167.84	174.06	172.56	166.22	156.00	171.93	165.09	155.30
Field 5								
1	175.91	174.96	182.84	178.31	172.80	174.31	170.93	173.64
2	176.28	167.44	156.35	165.49	172.41	168.23	147.93	162.14
3	159.24	156.07	169.30	171.47	150.69	154.89	165.70	163.77
4	163.03	170.52	171.43	162.13	160.42	163.84	160.72	156.46
5	154.55	168.09	166.10	163.67	155.28	162.63	157.58	158.01
6	169.73	169.18	170.68	166.75	152.57	155.05	155.59	162.99
7	135.21	143.22	144.24	140.72	135.39	138.16	140.38	138.01
8	156.20	151.38	155.70	154.56	151.42	156.29	155.38	152.82
9	156.48	148.29	156.49	154.36	152.86	144.56	149.03	155.62
10	151.64	150.67	152.32	154.65	151.53	147.73	140.03	144.43
11	163.34	165.20	169.00	172.46	160.55	155.46	154.85	160.60
12	166.94	157.08	160.02	167.83	164.45	149.73	151.61	161.80
13	172.53	165.53	169.45	169.66	160.51	162.79	164.33	164.13
14	170.45	170.04	170.83	183.62	165.04	164.24	166.96	167.36
15	170.73	174.58	172.07	171.47	170.81	169.12	164.62	169.99
16	176.76	168.55	179.12	182.61	176.50	170.43	172.17	172.20
17	184.28	177.96	184.61	182.97	175.68	175.35	167.12	169.07
18	181.46	182.70	181.97	177.87	173.85	171.47	170.52	171.33
19	177.34	173.68	168.45	174.75	174.99	173.17	168.66	164.95
20	166.33	170.80	169.97	174.11	169.88	170.65	164.05	164.76

21	173.34	167.89	169.60	174.96	170.58	171.81	170.08	168.06
22	173.96	160.97	175.69	172.47	169.02	169.20	165.64	170.31
23	169.88	163.35	160.91	167.21	167.09	165.40	151.54	159.48
Field 1-Lysosome								
1	65.37	65.02	64.21	68.93	65.90	63.48	62.56	63.77
2	64.34	66.81	64.95	64.52	61.37	60.18	56.42	59.60
3	59.29	61.30	61.49	58.26	57.01	60.76	58.33	55.29
4	65.83	68.42	68.76	64.74	59.33	62.57	61.28	60.26
5	62.73	63.77	66.98	66.13	62.75	64.42	61.87	62.11
6	65.90	66.66	66.84	69.08	62.78	63.69	60.07	59.49
7	58.45	59.40	62.38	58.78	57.39	59.01	57.43	56.39
8	61.45	63.71	62.27	59.76	60.42	60.51	60.54	59.88
9	64.39	67.27	67.98	63.94	64.55	65.34	67.10	64.15
10	67.18	67.21	67.60	67.32	66.70	66.45	66.28	67.20
11	68.30	65.22	63.99	71.63	60.03	62.80	63.92	65.54
12	67.44	67.05	62.30	65.49	61.20	62.71	60.80	60.35
13	62.76	62.72	64.50	65.51	59.44	60.45	55.64	56.82
14	63.41	61.77	67.20	66.27	55.50	59.63	58.06	61.25
15	64.25	63.03	64.44	60.77	61.83	59.58	57.77	62.08
16	66.51	63.05	62.86	66.71	61.18	60.46	57.66	61.26
17	63.94	61.53	62.28	63.03	56.23	57.05	58.26	57.34
18	59.12	59.29	60.56	61.72	52.88	58.84	55.68	57.62
19	58.87	61.08	60.41	59.41	55.08	56.28	57.59	54.43
20	61.24	61.77	62.32	61.00	59.28	59.21	61.17	60.54
Field 2								
1	65.87	68.96	65.47	65.24	62.74	61.83	61.59	59.61
2	63.55	64.75	64.13	63.21	61.02	62.98	62.99	59.89
3	61.08	64.45	57.91	64.30	60.03	58.91	57.17	58.46
4	61.75	60.62	59.45	61.12	60.17	57.62	57.19	59.37
5	63.26	59.38	61.87	60.63	60.61	58.55	55.38	57.09
6	65.36	61.13	64.06	62.46	59.99	60.16	57.42	59.49
7	62.32	63.79	66.40	66.23	61.15	59.89	61.84	60.36
8	63.17	65.70	65.48	64.76	62.72	62.72	63.49	61.37
9	65.47	64.68	64.66	66.43	61.91	62.55	62.04	62.62
10	63.15	63.19	61.80	63.02	59.67	61.62	61.28	60.05
11	59.75	60.90	59.53	59.40	58.09	59.00	59.33	57.03
12	63.77	62.69	62.34	64.66	60.99	60.32	61.65	62.53
13	65.46	61.53	62.33	64.77	62.40	60.98	61.62	61.42
14	61.42	58.04	61.27	62.11	60.13	58.26	58.37	59.87
Field 3								
1	60.04	64.51	60.20	63.78	61.28	60.41	57.31	58.84
2	63.74	62.94	64.35	63.20	57.39	60.64	57.03	58.73
3	61.66	62.06	64.17	60.74	57.65	58.43	55.71	53.93

4	62.99	63.38	63.97	62.42	59.05	59.15	61.18	60.19
5	62.45	66.19	64.57	62.36	60.65	62.43	60.61	61.28
6	63.26	63.84	61.15	65.10	59.92	53.87	63.65	61.75
7	61.82	64.03	63.34	61.40	56.37	56.68	55.24	57.46
8	66.01	65.41	65.15	63.61	60.66	63.16	63.22	59.11
9	63.24	60.46	65.32	64.38	61.06	59.03	61.74	59.65
10	62.39	62.81	65.03	63.23	61.10	62.30	61.26	59.78
11	59.91	61.58	62.27	61.96	59.51	60.50	59.99	59.65
12	59.72	57.41	60.07	65.54	58.15	58.50	58.54	57.90
13	56.02	58.78	58.09	56.00	55.23	57.61	56.70	54.86
14	59.88	59.47	62.85	57.92	56.37	57.47	55.96	56.61
15	59.65	63.55	62.84	59.47	58.03	55.68	57.75	57.71
16	63.92	61.82	65.30	61.79	62.87	61.78	60.61	59.24
17	59.27	60.26	62.70	56.23	58.87	58.25	58.48	56.36
18	61.38	59.98	60.45	64.38	60.99	59.70	57.77	58.50
19	61.50	58.83	57.56	57.94	57.83	57.57	56.36	56.76
20	58.53	56.54	57.47	61.14	58.39	57.44	56.67	55.65
21	61.47	58.88	57.31	58.20	56.03	56.01	53.26	51.25
Field 4								
1	62.15	60.84	61.11	61.20	60.71	60.41	57.22	59.15
2	61.72	61.74	60.95	60.56	58.63	58.11	58.14	58.43
3	61.22	58.64	62.80	59.09	58.00	60.95	56.84	60.01
4	57.15	60.97	59.72	59.03	54.86	56.61	56.79	54.40
5	62.84	61.19	60.00	59.73	56.06	59.46	61.26	57.73
6	62.26	61.51	63.25	60.59	58.69	59.86	58.42	58.95
7	57.99	59.23	58.71	56.86	56.25	58.41	56.32	56.31
8	54.77	57.88	57.13	55.14	53.57	54.27	53.17	54.51
9	58.95	61.49	59.95	58.54	56.46	57.49	55.56	58.39
10	55.06	57.77	58.20	58.24	52.69	54.75	53.87	50.79
11	57.90	59.20	58.65	57.06	54.49	55.52	57.15	54.23
12	61.19	63.08	59.81	60.90	56.36	56.16	58.73	55.63
13	59.89	63.79	61.00	61.29	59.10	57.32	54.78	56.96
14	61.40	60.75	58.64	60.70	60.66	59.75	59.36	58.40
15	61.60	60.45	58.27	61.30	57.91	56.92	57.06	60.56
16	61.33	58.11	58.67	61.23	58.77	57.57	56.23	54.19
17	59.80	59.74	56.84	60.45	59.00	59.75	57.88	58.25
18	62.14	59.83	60.25	59.41	56.39	59.30	57.59	55.67
19	61.07	58.73	57.35	59.24	56.27	55.84	53.97	58.02
20	59.72	59.95	59.49	61.68	59.62	58.60	58.01	57.61
21	58.44	55.74	55.90	60.46	54.64	53.79	54.88	56.90
22	55.43	53.17	53.64	56.49	52.45	53.00	54.65	54.91
23	60.60	56.12	57.74	57.56	54.90	55.69	54.93	56.79
24	58.64	57.35	57.59	60.61	57.57	56.56	57.18	58.71

25	57.87	58.31	58.89	60.91	56.58	53.72	56.02	58.60
26	60.03	61.38	62.59	59.90	59.25	61.99	62.09	61.64
27	57.92	59.84	60.39	58.65	55.30	57.19	58.81	57.58
28	58.30	60.15	59.84	60.12	58.08	58.66	58.78	58.73
29	59.87	60.52	59.96	60.19	55.48	58.21	58.12	55.32
30	61.25	62.04	61.37	60.25	56.52	57.20	60.05	58.63
31	58.56	59.59	59.53	58.84	56.87	57.74	56.38	57.94
32	58.27	60.07	58.42	59.00	55.97	56.18	56.68	54.48
33	57.33	56.41	57.46	55.20	56.48	57.78	54.79	55.46
34	57.19	56.17	56.34	56.97	56.02	52.07	51.84	52.20
35	55.70	55.09	56.09	54.99	53.54	53.68	53.45	52.96
36	61.79	62.08	61.95	57.35	59.04	56.29	61.33	58.77
Field 1-Actin								
1	156.12	159.21	157.99	154.75	149.04	158.30	158.73	150.53
2	160.77	166.69	165.05	158.79	156.34	164.53	159.05	156.58
3	157.85	165.06	160.02	157.95	160.24	160.89	159.03	153.68
4	159.40	165.04	159.81	161.38	161.46	162.14	164.02	157.45
5	156.75	155.83	158.90	154.07	153.93	162.00	156.98	153.90
6	147.84	153.95	149.45	145.57	144.47	149.53	148.66	139.54
7	149.54	153.32	152.70	147.24	146.32	147.09	151.10	146.76
8	152.49	155.57	155.11	152.26	153.04	154.41	154.73	154.50
9	157.17	164.50	159.34	161.41	157.24	162.48	162.75	158.73
10	158.08	154.96	156.53	161.74	153.04	158.53	153.07	154.50
11	154.65	155.53	151.91	149.44	152.68	151.63	150.95	147.92
12	155.82	151.56	154.05	150.70	151.04	152.52	153.60	153.44
13	168.42	162.90	159.92	167.89	163.40	159.32	163.57	166.50
14	173.34	170.98	170.89	172.34	172.44	168.15	166.00	167.91
15	175.15	168.95	174.85	168.54	171.59	171.25	165.38	170.45
16	174.75	168.36	164.44	172.03	163.77	166.18	167.13	167.73
17	168.40	166.55	164.43	174.92	170.45	168.97	164.98	159.86
Field 2								
1	162.53	163.41	164.75	160.94	162.49	162.01	158.00	161.86
2	166.11	165.17	165.21	165.40	165.75	164.47	165.55	163.41
3	169.82	165.58	164.11	167.23	171.23	164.58	161.51	163.63
4	166.54	166.07	167.16	162.25	164.82	168.71	162.96	156.78
5	163.50	159.65	161.47	162.17	155.60	159.09	158.73	163.33
6	161.31	162.48	167.17	161.35	161.20	157.80	155.72	158.39
7	152.75	154.28	159.94	154.37	153.31	158.43	149.87	145.24
8	150.74	154.64	154.35	154.30	145.69	155.35	154.66	149.65
9	147.31	154.52	151.24	149.96	148.23	148.96	147.33	145.79
10	150.89	157.36	154.15	149.65	149.85	152.51	151.79	149.41
11	167.04	167.09	168.38	162.29	159.13	167.77	166.11	162.73
12	174.21	175.62	174.86	173.28	172.61	169.29	165.12	169.76

13	175.01	172.59	169.91	174.22	168.29	169.15	170.74	169.91
14	162.32	172.11	166.51	164.00	164.89	162.69	164.90	163.01
15	170.25	169.77	166.89	166.63	166.03	167.71	165.95	163.80
16	164.15	162.79	165.71	169.29	162.96	163.94	166.27	163.77
17	165.85	167.14	168.06	163.37	164.55	165.30	166.27	165.21
18	156.03	162.18	157.32	157.12	153.80	156.33	160.73	150.83
19	154.59	160.62	162.72	153.94	153.52	156.71	155.72	153.62
20	158.98	161.41	159.14	160.55	160.18	158.87	161.31	158.13
21	160.26	162.21	163.67	163.87	156.11	152.93	160.49	155.51
22	164.91	168.65	163.54	163.03	160.80	162.42	162.23	161.91
23	158.66	158.77	158.86	159.11	155.33	157.27	159.34	159.67
24	136.33	145.25	145.63	139.98	135.23	141.71	146.61	137.70
25	152.14	152.81	152.27	152.10	152.36	148.46	151.47	152.21
26	152.96	154.82	155.12	154.20	154.97	149.98	152.12	152.41
27	175.36	167.93	170.13	173.18	173.64	170.13	168.16	171.86
28	168.88	161.47	164.30	164.39	166.50	159.34	161.47	164.34
29	160.53	155.05	159.52	161.65	159.24	153.61	153.59	161.41
30	169.08	157.18	158.38	162.98	160.58	157.38	157.21	163.44
Field 3								
1	159.80	163.10	159.93	155.96	156.40	159.81	158.14	148.60
2	163.48	163.49	164.93	164.70	164.97	167.74	162.34	153.41
3	158.17	160.87	159.27	159.49	157.62	159.53	155.40	148.59
4	157.27	160.23	158.17	155.45	148.25	156.12	154.96	152.13
5	151.85	158.34	153.60	153.16	152.95	152.05	154.43	148.74
6	166.73	166.59	164.84	166.37	166.06	168.60	161.77	157.64
7	169.26	166.93	168.64	166.94	168.08	167.42	164.88	167.36
8	163.27	160.76	161.53	165.62	164.51	160.13	160.52	158.95
9	160.69	158.36	155.89	161.60	157.48	157.61	160.25	160.00
10	169.76	171.54	169.62	169.04	167.80	165.44	168.33	168.38
11	163.03	167.49	164.42	166.09	161.57	161.15	159.70	155.72
12	162.19	163.15	164.23	161.12	155.61	161.41	163.35	157.66
13	165.25	164.55	165.75	163.81	161.25	165.30	162.17	156.02
14	165.73	169.04	170.16	166.70	166.56	169.20	162.74	164.26
15	169.37	171.63	169.24	171.50	172.23	166.28	164.20	165.59
16	167.84	170.40	168.64	166.71	165.37	166.27	165.42	159.95
17	167.99	167.57	168.91	165.24	163.95	167.83	165.55	166.90
18	163.45	161.41	165.51	164.74	161.63	160.18	161.77	161.43
19	159.93	159.17	162.89	160.39	160.03	159.52	155.25	161.22
20	157.80	158.01	158.79	163.91	151.38	155.97	159.91	156.24
21	160.63	162.88	160.50	161.44	158.01	158.21	157.79	153.50
22	162.92	170.03	161.15	166.38	166.11	164.75	165.47	162.61
23	138.63	144.16	142.47	138.55	139.55	144.14	142.92	137.92
Field 4								

1	165.66	165.76	164.36	162.47	163.88	165.87	164.49	159.34
2	163.11	167.41	167.50	162.87	163.18	164.70	163.32	164.19
3	162.71	165.13	164.04	162.37	161.31	164.22	162.56	161.62
4	169.79	167.75	168.07	166.52	166.38	169.04	167.73	165.53
5	169.66	167.79	166.97	168.11	163.46	169.69	166.24	163.65
6	161.07	163.68	164.30	162.53	160.49	158.91	162.24	156.18
7	161.31	160.32	159.76	164.99	161.96	160.00	159.78	158.91
8	153.47	152.87	155.07	154.29	152.65	149.11	148.59	154.13
9	155.67	153.27	158.03	154.26	151.98	153.67	151.07	152.67
10	152.89	153.70	150.02	154.41	151.33	153.32	152.56	152.45
11	145.08	150.59	150.91	145.82	143.28	147.58	151.20	140.56
12	145.00	148.55	153.93	142.50	142.95	150.50	146.03	138.01
Field 5								
1	161.30	164.94	162.81	159.98	160.13	162.47	155.09	151.23
2	161.07	161.25	165.21	162.89	156.25	156.77	156.29	153.15
3	155.35	160.78	156.51	155.57	157.60	155.64	156.57	151.75
4	141.58	145.59	144.51	142.72	135.90	141.41	143.51	135.52
5	144.01	148.02	147.96	142.04	139.61	147.39	145.36	142.12
6	141.62	144.60	141.46	140.88	132.19	143.47	141.41	132.24
7	142.14	146.21	148.35	142.41	139.51	141.24	141.56	138.64
8	160.49	159.38	160.27	156.02	154.08	156.96	156.24	159.15
9	155.56	157.82	162.41	159.74	150.79	152.42	149.21	156.67
10	156.00	156.33	157.32	157.27	152.80	155.02	153.02	150.68
11	153.79	154.49	156.08	154.77	151.01	154.04	150.63	152.57
12	154.69	156.69	158.08	155.94	152.96	156.28	154.02	155.14
13	149.91	153.99	158.09	152.22	151.18	153.13	147.24	148.67
14	157.00	156.32	159.09	158.23	151.74	153.62	156.16	155.72
15	150.41	151.61	149.01	149.99	147.50	148.70	150.69	143.86
16	149.88	149.81	149.24	149.44	147.78	138.60	150.78	146.57
17	165.37	160.93	162.27	165.94	158.62	160.64	160.79	153.38
18	164.87	163.97	160.15	167.02	162.92	159.94	163.99	162.29
19	165.31	160.57	153.79	162.65	166.39	159.64	152.55	155.79
20	167.72	162.78	164.91	166.65	163.62	166.79	161.98	155.22
21	158.63	153.92	158.63	158.69	155.26	155.74	150.36	149.99
22	157.15	149.36	150.39	156.17	155.80	151.15	149.49	153.29
Field 1-Tubulin								
1	130.48	130.68	124.29	127.22	125.20	124.44	114.47	118.79
2	128.59	128.35	124.65	128.49	129.20	128.15	124.55	128.46
3	120.61	124.56	118.79	124.00	113.92	116.14	113.41	112.53
4	126.31	127.26	117.08	107.25	119.41	122.38	118.81	121.45
5	122.14	122.89	119.93	121.69	123.50	125.23	118.67	122.22
6	120.25	125.51	125.09	119.88	123.08	126.05	121.50	116.92
7	116.08	125.32	128.45	126.66	119.75	124.36	120.30	116.02

8	121.87	124.21	120.83	122.42	113.87	120.77	123.42	114.79
9	116.50	115.93	119.46	115.84	116.77	121.60	121.35	116.80
10	108.83	116.02	108.14	110.86	111.29	114.40	116.29	107.57
11	109.47	118.61	115.91	111.82	110.14	117.22	117.30	110.60
12	125.62	134.25	130.76	125.09	126.37	119.79	125.51	114.59
13	115.97	120.63	126.59	123.73	119.60	123.31	121.64	120.12
14	117.81	122.99	124.11	119.43	118.34	121.83	121.78	119.60
15	109.24	113.59	118.40	114.56	110.54	110.52	103.76	111.65
16	112.98	107.16	114.34	117.89	111.04	110.92	108.65	116.81
17	121.38	111.90	113.60	125.02	121.30	108.73	108.30	111.95
18	131.45	124.68	122.76	130.22	128.90	124.59	122.81	128.32
19	122.62	116.08	111.20	121.58	120.38	113.96	111.15	115.35
20	125.14	119.26	112.80	124.52	122.26	112.59	113.89	117.35
21	117.63	104.07	101.76	105.93	116.30	106.21	104.16	109.96
22	121.89	109.64	108.80	120.07	115.99	111.21	103.83	112.72
Field 2								
1	132.35	126.14	124.60	125.40	130.32	128.30	122.48	120.29
2	129.91	129.83	126.34	130.85	131.28	130.35	123.15	127.82
3	135.01	132.07	128.44	130.42	132.61	132.74	127.01	133.37
4	136.62	133.43	131.71	121.39	135.64	134.37	128.78	130.42
5	134.24	140.40	127.10	126.01	132.19	135.01	123.89	126.11
6	134.59	131.30	134.10	124.38	128.58	130.26	128.13	125.29
7	137.78	135.89	138.81	135.90	134.29	136.43	136.23	128.51
8	128.30	133.08	127.91	129.87	125.28	127.87	130.68	129.58
9	131.24	135.33	130.78	134.87	136.39	131.84	131.21	130.30
10	129.38	133.26	136.30	133.68	129.91	135.01	130.76	136.16
11	128.64	127.79	131.83	128.36	123.44	129.18	124.43	120.27
12	129.18	131.72	127.86	125.76	129.77	132.13	129.32	128.18
13	128.25	132.51	136.12	145.27	126.41	129.12	134.94	130.57
14	122.71	126.76	132.71	123.24	123.93	127.31	130.87	123.00
15	123.36	133.15	127.47	124.44	118.31	122.59	130.08	122.50
16	116.73	126.50	124.35	118.94	115.31	123.48	123.18	116.36
17	111.06	116.51	115.36	113.98	106.38	111.01	115.66	109.77
18	103.65	112.94	114.35	99.47	102.52	107.34	106.02	103.86
19	103.53	103.27	108.76	101.90	101.74	106.13	107.38	102.35
20	110.76	115.37	121.31	114.89	112.45	114.93	122.48	112.95
21	119.27	120.31	122.25	122.84	116.26	112.60	120.61	114.39
22	125.29	123.49	129.30	127.94	126.13	126.43	125.64	126.66
23	126.11	126.64	125.30	125.65	128.52	122.19	122.84	127.44
24	117.35	119.58	122.96	128.85	120.28	116.42	118.47	124.99
25	117.56	113.28	126.73	130.07	115.01	114.69	113.80	121.51
26	111.95	108.89	114.00	118.89	116.54	105.92	111.60	117.53
27	117.00	115.66	116.80	119.56	116.83	113.85	116.49	118.01

Field 3								
1	131.75	126.70	124.76	128.49	130.05	123.83	121.80	126.73
2	135.58	138.44	130.38	131.95	131.94	127.89	129.42	128.49
3	132.99	136.82	134.14	132.43	132.83	128.73	127.63	132.85
4	126.50	129.96	126.21	125.06	121.86	130.14	128.84	119.24
5	125.09	136.39	129.83	127.12	120.23	121.61	123.40	119.07
6	123.25	122.72	126.58	125.38	115.33	123.05	121.95	113.61
7	123.15	124.74	126.59	121.85	120.93	125.22	119.34	117.99
8	115.67	121.45	120.90	116.13	116.81	120.58	119.12	113.81
9	121.75	132.13	127.62	123.62	120.83	129.38	127.62	116.49
10	108.45	111.24	116.45	115.40	99.76	106.70	101.57	98.08
11	131.05	133.61	133.60	129.23	130.17	132.63	125.67	127.39
12	137.17	138.99	134.87	136.53	133.87	135.71	132.49	134.86
13	132.54	133.70	138.00	136.07	126.57	126.93	124.40	126.85
14	126.33	123.31	139.11	131.23	127.86	124.68	127.43	121.65
15	120.83	126.37	126.59	124.10	122.18	121.27	120.55	123.55
16	115.12	116.92	125.20	124.68	112.80	114.30	115.28	106.67
17	132.17	132.09	136.81	135.67	127.45	122.90	125.72	129.72
18	127.94	133.54	130.81	135.42	129.79	132.08	131.15	131.34
19	122.63	136.16	134.79	130.18	131.28	127.31	121.65	132.15
20	118.80	117.31	117.12	125.42	118.87	116.14	113.15	120.96
21	125.97	120.47	122.33	122.66	124.95	120.68	117.46	123.56
22	120.15	115.36	114.53	120.79	119.87	109.24	104.49	118.75
23	124.54	118.17	120.58	126.95	126.30	116.01	113.69	122.70
24	132.19	126.61	125.91	132.16	131.85	125.04	123.26	129.04
25	138.26	117.73	113.42	120.60	127.27	115.20	109.70	120.34
26	143.37	123.69	121.92	131.35	134.00	120.41	122.64	129.15
27	135.57	128.74	130.47	134.64	134.32	132.55	130.31	134.23
Field 4								
1	130.03	128.90	124.61	135.08	133.67	126.65	124.89	131.43
2	126.29	128.69	132.45	129.18	128.77	123.63	123.52	129.39
3	147.61	127.84	122.75	136.41	133.44	129.04	126.79	128.56
4	131.04	132.49	133.76	132.00	132.56	123.98	121.35	126.96
5	133.19	137.44	132.47	125.53	129.55	127.75	122.43	116.44
6	132.74	134.21	131.47	132.25	129.03	132.71	128.01	126.74
7	131.35	134.12	133.29	135.90	137.64	133.98	129.15	132.39
8	133.46	138.02	136.05	129.93	137.32	139.86	135.62	133.57
9	134.87	143.27	134.65	131.24	133.84	136.58	133.04	130.84
10	124.65	131.03	130.71	126.00	124.47	129.46	130.03	121.90
11	120.84	129.94	122.41	118.70	116.66	123.08	121.65	117.91
12	123.54	132.37	127.66	118.08	119.96	125.93	126.66	120.58
13	130.07	130.29	121.56	116.32	125.02	125.91	126.33	117.85
14	121.48	128.56	129.48	123.04	117.59	127.74	125.52	119.55

15	112.56	124.25	126.23	119.59	113.07	119.13	121.23	116.08
16	127.74	122.51	124.58	135.55	129.76	120.45	122.03	126.99
17	131.50	123.76	125.24	130.14	127.20	128.69	125.40	129.06
18	122.82	117.81	117.84	124.16	120.61	110.62	113.30	116.94
19	130.02	128.94	128.20	130.79	122.96	123.43	126.35	127.67
20	126.46	130.41	138.92	127.43	123.58	122.62	123.48	126.26
21	118.78	128.80	117.35	119.60	116.88	115.70	114.65	115.88
22	117.22	118.88	119.72	124.17	114.50	112.68	121.40	118.37
23	117.96	113.47	121.57	121.06	107.76	116.89	118.96	117.88
24	107.57	114.99	109.94	113.80	102.52	107.64	111.23	105.98
25	115.48	121.37	119.72	109.94	107.73	113.85	113.60	114.60
26	107.84	114.56	117.41	112.14	105.69	110.09	114.20	106.19
27	111.99	113.72	103.64	103.65	109.84	104.47	99.15	100.58
28	115.26	116.09	106.97	118.39	113.10	105.95	110.76	119.93

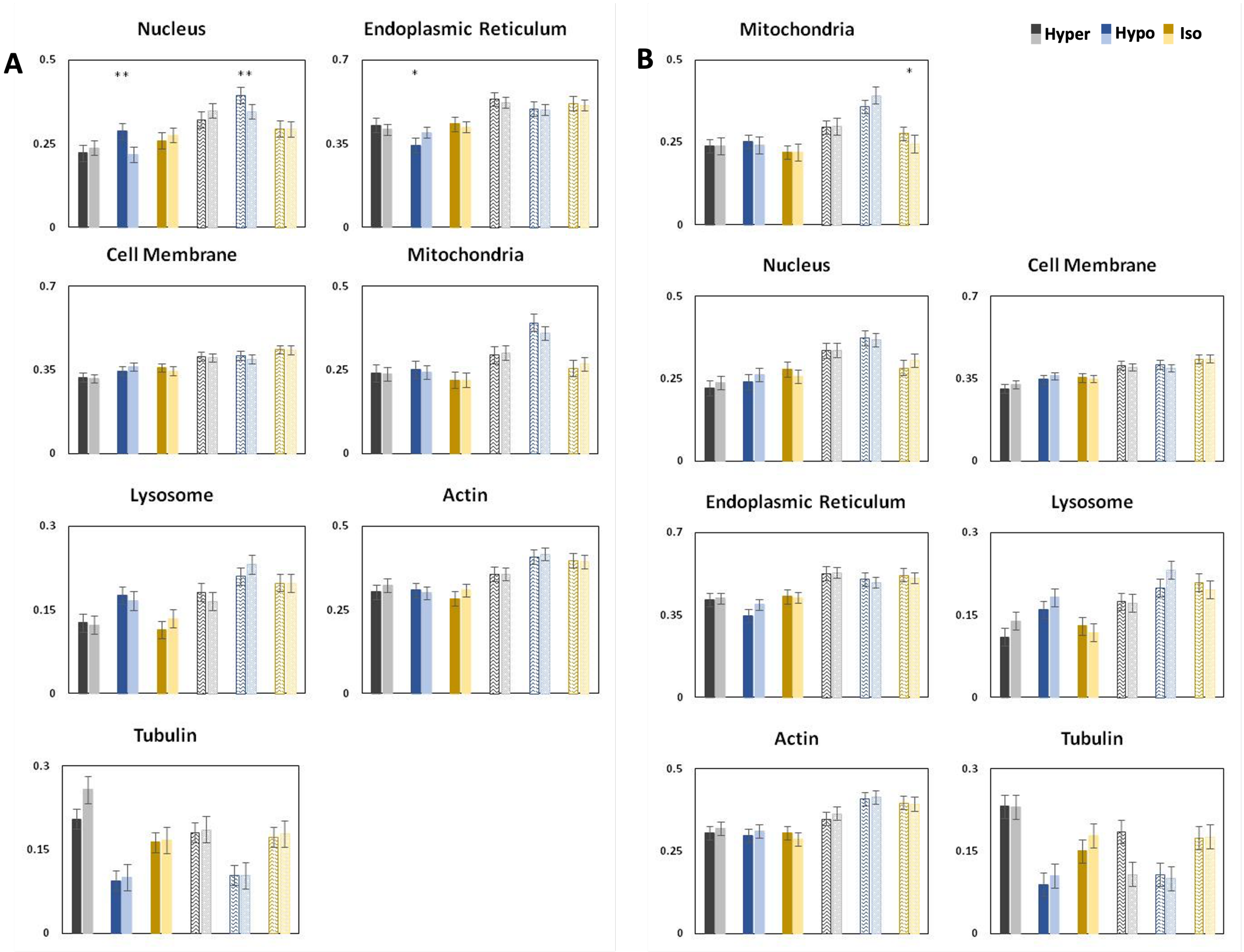

Fig. S1: A and B show bar graphs depicting average APVs (along y-axis) of fluorescent images of Nuclei, Cell Membrane, Mitochondria, Endoplasmic Reticulum, Lysosome, Actin, and Tubulin in asymmetric (solid bars) and symmetric (patterned bars) RAW264.7 cells along the y (A) and x (B) axes direction in hypertonic (black and gray bars), hypotonic (dark and light blue bars), and isotonic (dark and light-yellow bars) conditions (mean±SE). Individual n values are mentioned in Table 1 in manuscript.

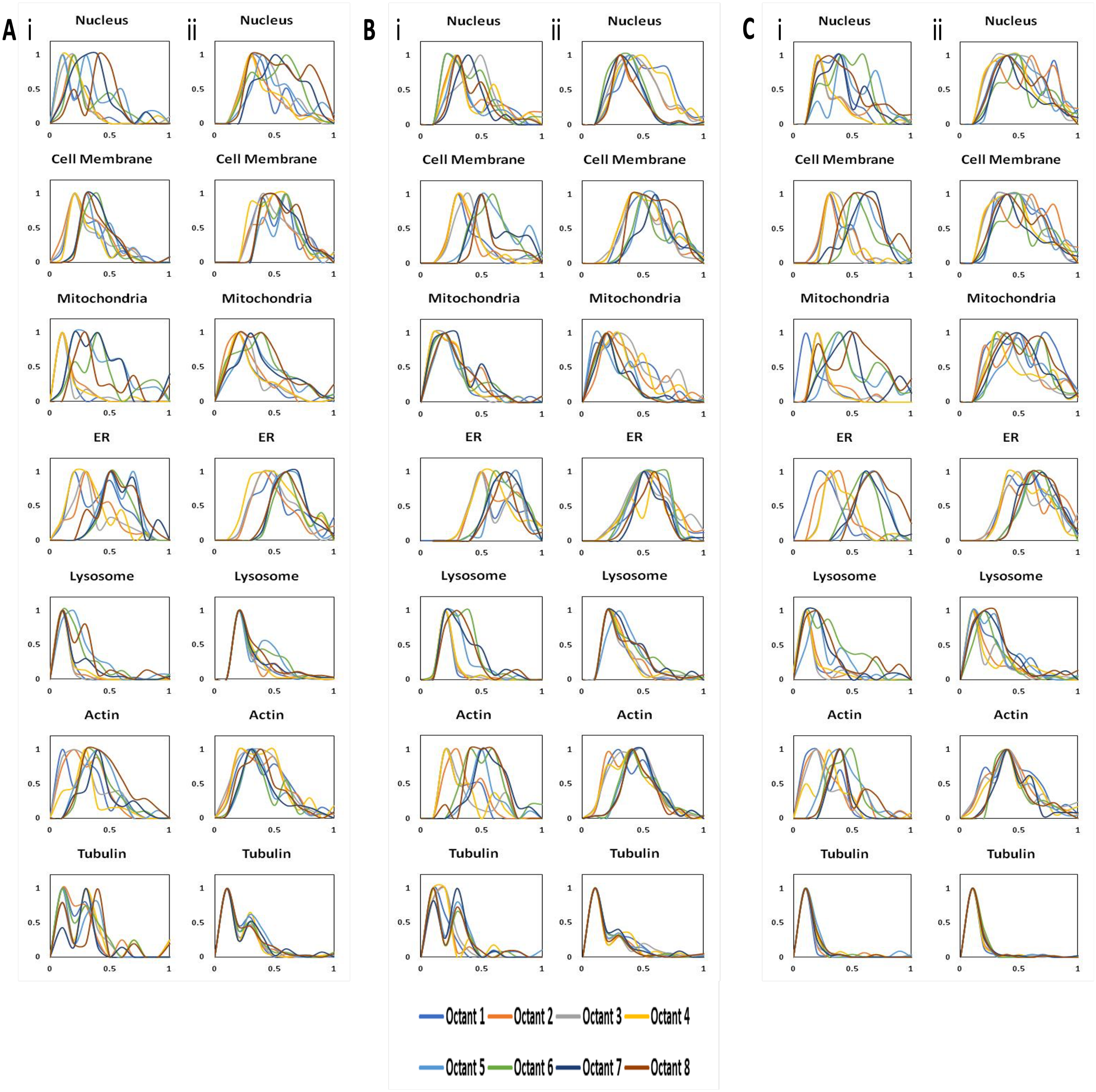

Fig. S2: A-C show the distribution normalized frequency of the number of cells along APV values for each octant (color coding below Tubulin Frequency distribution) in asymmetric (i) and symmetric (ii) RAW264.7 cells in hypertonic (A), isotonic (B) and hypotonic (C) conditions for different organelles imaged (namely Nucleus, Cell Membrane, Mitochondria, Endoplasmic Reticulum, Lysosome, Actin, and Tubulin).

METHODS

The Python code for calculating the area under the curves from Figure S2 is below:

```
import numpy as np
import matplotlib.pyplot as plt

# Define APV array
x = np.array([0, 0.1, 0.2, 0.3, 0.4, 0.5, 0.6, 0.7, 0.8, 0.9, 1.0])

# Define FREQ arrays – Different datasets
y1 = np.array([…..])
y2 = np.array([…..])
y3 = np.array([…..])
y4 = np.array([…..])
y5 = np.array([…..])
y6 = np.array([…..])
y7 = np.array([…..])
y8 = np.array([…..])

# Calculate common area using trapezoidal rule
common_area = np.trapz(np.minimum.reduce([y1, y2, y3, y4, y5, y6, y7, y8]), x)

# Plot the curves
plt.plot(x, y1, label='y1')
plt.plot(x, y2, label='y2')
plt.plot(x, y3, label='y3')
plt.plot(x, y4, label='y4')
plt.plot(x, y5, label='y5')
plt.plot(x, y6, label='y6')
plt.plot(x, y7, label='y7')
plt.plot(x, y8, label='y8')

# Shade the common area
plt.fill_between(x, np.minimum.reduce([y1, y2, y3, y4, y5, y6, y7, y8]), alpha=0.3, color='gray', label='Common Area')

# Add labels and legend
plt.xlabel('x-axis')
plt.ylabel('y-axis')
plt.legend()

# Show the plot
plt.show()

# Print the common area
print(f"Common Area: {common_area}")
```
